# Design and optimisation of meta-substituted bis(arylsulfonamido)benzene inhibitors through a molecular hybridisation strategy targeting the Keap1-Nrf2 protein-protein interaction[Author-notes AUFN1]

**DOI:** 10.1080/14756366.2026.2622777

**Published:** 2026-02-06

**Authors:** Sumi Lee, Ahmed R. Ali, Dhulfiqar Ali Abed, Longqin Hu

**Affiliations:** a Department of Medicinal Chemistry, Ernest Mario School of Pharmacy, Rutgers, The State University of New Jersey, Piscataway, NJ, USA; b School of Pharmacy, Jeonbuk National University, Jeonju, Republic of Korea; c Rutgers Cancer Institute of New Jersey, New Brunswick, NJ, USA

**Keywords:** Keap1-Nrf2 binding, PPI Inhibitor, oxidative stress, molecular hybridisation

## Abstract

Nrf2 is recognised as an attractive therapeutic target for oxidative stress-related disorders through its regulation of antioxidant gene transcription. Direct inhibition of Keap1-Nrf2 protein-protein interaction represents a promising strategy to modulate Nrf2 activity. Herein, we report the discovery of meta-substituted bis(arylsulfonamido)benzene derivatives using a molecular hybridisation strategy based onpotent inhibitors **2a** and **3a**. Among the initial hybrids, **7a** demonstrated good potency in the FP assay, making it a suitable lead for SAR optimisation. Our study found **13b** was the most potent analog, showing IC_50_ values of 183.4 nM in the FP assay and 107.5 nM in the TR-FRET assay. It also demonstrated excellent metabolic stability, with 93.9% remaining after a 30 minute-incubation in human liver microsomes. Collectively, these results highlight **13b** as a non-covalent Keap1-Nrf2PPI inhibitor, with balanced potency and metabolic stability, supporting its potential as a tractable scaffold for further optimisation to modulate the Nrf2 pathway.

## Graphical Abstract

**Figure:**
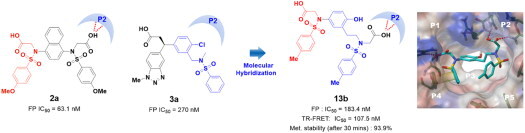

## Introduction

Nrf2 (nuclear factor erythroid 2-related factor 2) has gained attention as a valuable drug target for various diseases associated with oxidative stress^1^, including malignancies^2^, COPD (chronic obstructive pulmonary disorder)^3^, MS (multiple sclerosis)^4^, as well as a variety of neurodegenerative disorders^5^^,^^6^. Serving as a central regulator of antioxidant defense pathways, Nrf2 governs both transcription and subsequent expression of numerous antioxidant and detoxification genes through interaction with AREs (antioxidant response elements) in gene promoters^7^^,^^8^.

In the absence of stimulation, Nrf2 activity is suppressed by Keap1 (Kelch-like ECH-associated protein 1)^9^. The Keap1 interacts with the Cul3-RBX1 E3 ubiquitin ligase complex^10^^,^^11^ ,acting as a bridging adaptor. This process positions Nrf2 near the ligase system^12^, promoting its rapid proteasomal degradation^13^^,^^14^. In the presence of electrophilic or oxidative stress, Nrf2 inducers, such as electrophiles, and ROS (reactive oxygen species), covalently alter reactive cysteines located within the IVR and BTB regions of Keap1^15^. This modification disrupts the Keap1-Nrf2 binding, which suppresses Nrf2 ubiquitination through three possible mechanisms^13^: i) dissociation of Cul3 from Keap1^16^^,^^17^, ii) displacement of Nrf2’s DLG motif^18–20^, and iii) conformational disruption of Keap1^21^. Consequently, newly synthesised Nrf2 translocates into the nucleus to dimerise with small musculoaponeurotic fibrosarcoma (Maf) proteins and other transcriptional cofactors^22^. These complexes bind to AREs within the promoters of drug target genes, thereby inducing the expression of various cytoprotective proteins and detoxifying enzymes, including HO-1 (haem oxygenase-1), TRX (thioredoxin), and GCL (glutamate-cysteine ligase). This ultimately protects cells against oxidative stress^22–24^.

This well-characterised regulatory mechanism has made targeting the Keap1-Nrf2 protein-protein interaction (PPI) a major focus for developing therapeutic and preventive interventions against oxidative stress-associated disorders and inflammation^23^. Earlier approaches utilised electrophilic compounds that indirectly activated Nrf2 by covalently modifying reactive cysteine residues on the Keap1 protein, thereby preventing its interaction with Nrf2^25^. Notable examples of such Nrf2 stimulators include DMF (dimethyl fumarate), CDDO-Me (bardoxolone methyl), and omaveloxolone^26–28^. In 2013, DMF received U.S. FDA approval to manage relapsing MS^26^. Bardoxolone methyl (CDDO-Me) was investigated in a phase II clinical study targeting chronic kidney disorder among patients with type 2 diabetes. However, the subsequent phase III trial was discontinued because of cardiac adverse outcomes. Subsequent studies suggested that unintended interactions involving covalent cysteine-reactive molecules may have been responsible for the unanticipated safety risks^27^^,^^28^. In 2023, omaveloxolone, an analog of bardoxolone, gained FDA approval for treating Friedreich’s ataxia, a rare inherited neurodegenerative disease characterised by dysregulated Nrf2 signalling^29^.

**Figure 1.**
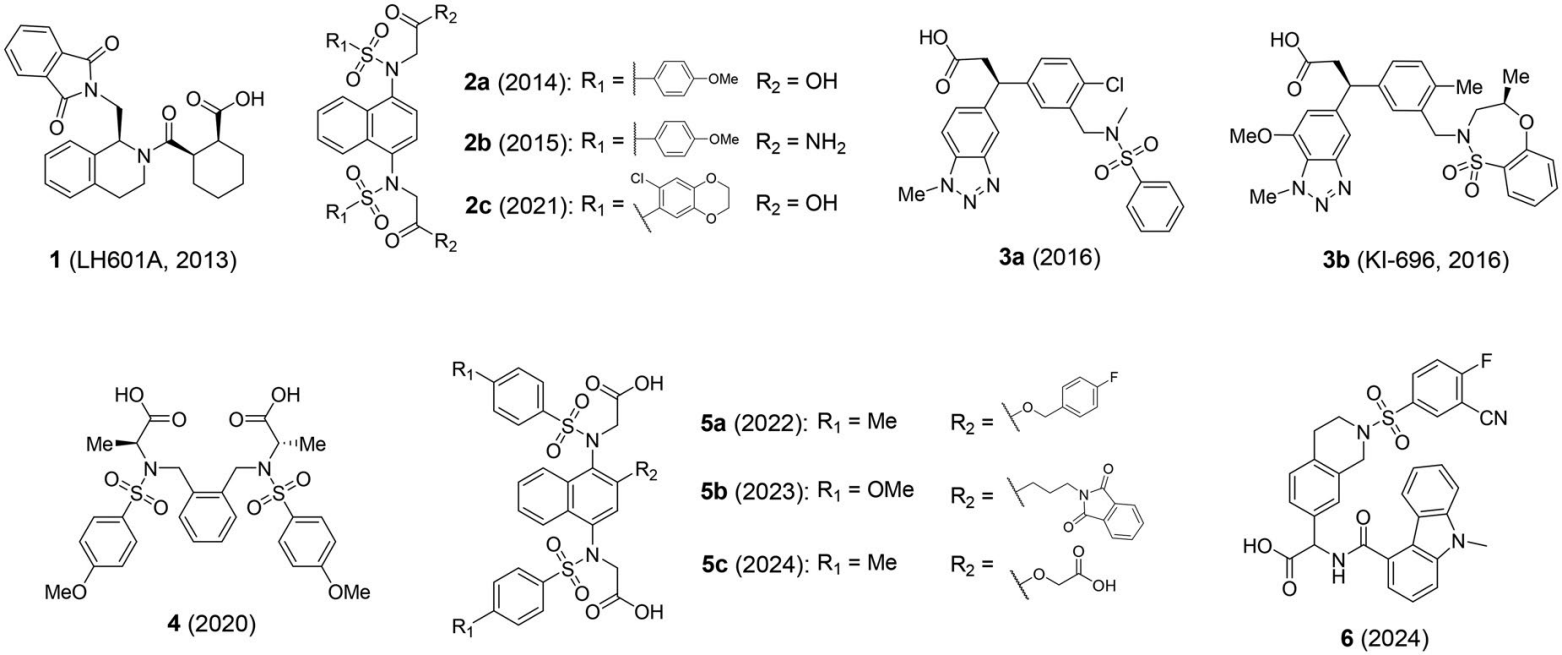
Notable compounds directly interfering with the Keap1-Nrf2 protein complex.

An alternative, more selective strategy involves using direct, non-covalent inhibitors that interfere with the Keap1-Nrf2 binding. These inhibitors target the binding interface where the Keap1 Kelch domain engages the Neh2 domain of Nrf2 *via* its DLG and ETGE motifs^18^^,^^20^. Disruption of one or both of these interactions by a small molecule allows newly formed Nrf2 to move into the nucleus, activating ARE-regulated gene transcription and triggering the antioxidant response^30^^,^^31^. Compared to electrophilic compounds, these direct small-molecule inhibitors have greater selectivity for the Keap1 Kelch domain, reducing undesired effects from binding to other cysteine-containing proteins. Despite these advantages, the development of non-covalent Keap1-Nrf2 inhibitors remains challenging due to the large and arginine-rich nature of the Kelch binding pocket, which often necessitates the incorporation of polar functionalities that can compromise membrane permeability and overall pharmacokinetic profiles. In addition, achieving an optimal balance between potency, selectivity, and drug-like characteristics continues to represent a key hurdle in advancing such inhibitors towards therapeutic applications. Drawing inspiration from well-characterised co-crystallized structures showing the Kelch domain bound to various Nrf2 peptides and small-molecule ligands, direct non-covalent PPI inhibitors have been recently developed^32^^,^^33^. Notably, small molecules bearing tetrahydroisoquinoline (**1**)^34^, 1,4-diaminonaphthalene (**2a-c**)^35–37^, 3-phenylpropanoic acid (**3a-b**)^38^, 1,2-xylylenediamine (**4**)^39^, 2-substituted naphthalene (**5a-c**)^40–42^, and 9*H*-carbazole carboxamide (**6**)^43^ have exhibited remarkable potency as Keap1-Nrf2 PPI inhibitors (Figure 1).

The symmetric 1,4-diaminonaphthalene series was initially discovered through high-throughput screening (HTS) of a 267,551-compound library, which was screened using a homogeneous confocal fluorescence anisotropy (FA) assay^44^. Subsequent computational analyses enabled the rational design of **2a**, in which symmetric acetic acid substituents were incorporated into the sulphonamide moieties^35^. Compound **2a** showed potent suppression of the Keap1-Nrf2 binding, displaying an IC_50_ of 28.6 nM in a FP (fluorescence polarisation) assay. In cell-derived functional experiments, an analog of **2a** bearing 4-acetamidosulfonamide substituents induced Nrf2 activation, leading to upregulation of downstream cytoprotective enzymes, including glutamate–cysteine ligase modifier (GCLM), haem oxygenase 1 (HO-1), and NAD(P)H:quinone oxidoreductase 1 (NQO1). Moreover, significant suppression of pro-inflammatory cytokines was observed in mice exposed to LPS-induced inflammation upon treatment with this compound^35^^,^^45^. In 2016, a 3-phenylpropanoic acid scaffold was identified *via* a fragment-based drug design (FBDD) approach by Astex Pharmaceuticals in collaboration with GlaxoSmithKline^38^. At the early optimisation stage, a fragment growth approach was adopted to produce compound **3a**, which showed an IC_50_ value of 0.27 μM in the FP assay. Subsequent structural optimisation led to the discovery of a cyclic sulphonamide derivative, **3b** (KI-696), which exhibited the most potent inhibitory activity, achieving 95% inhibition at a concentration of 15 nM. Furthermore, both *in vitro* and *in vivo* studies confirmed that KI-696 (**3b**) effectively activated the Nrf2-mediated antioxidant response^38^^,^^46^.

Building on these findings, we report the rational, structure-based design of a new meta-substituted bis(arylsulfonamido)benzene scaffold derived from a molecular hybridisation strategy guided by two previously identified direct inhibitors **2a** and **3a**^47^. Inhibitory activity against the Keap1-Nrf2 interaction was assessed for the proposed compounds (**7a**, **8**, and **9**), identifying **7a** as a suitable lead for subsequent optimisation. Follow-up biological testing revealed that compound **13b** was the most potent inhibitor, with IC_50_ values of 183.4 nM, as measured by the FP assay, and 107.5 nM in a TR-FRET (time-resolved fluorescence resonance energy transfer) assay. These results provide strong support for further investigation of this scaffold.

## Results and discussion

### Design rationale

**Figure 2.**
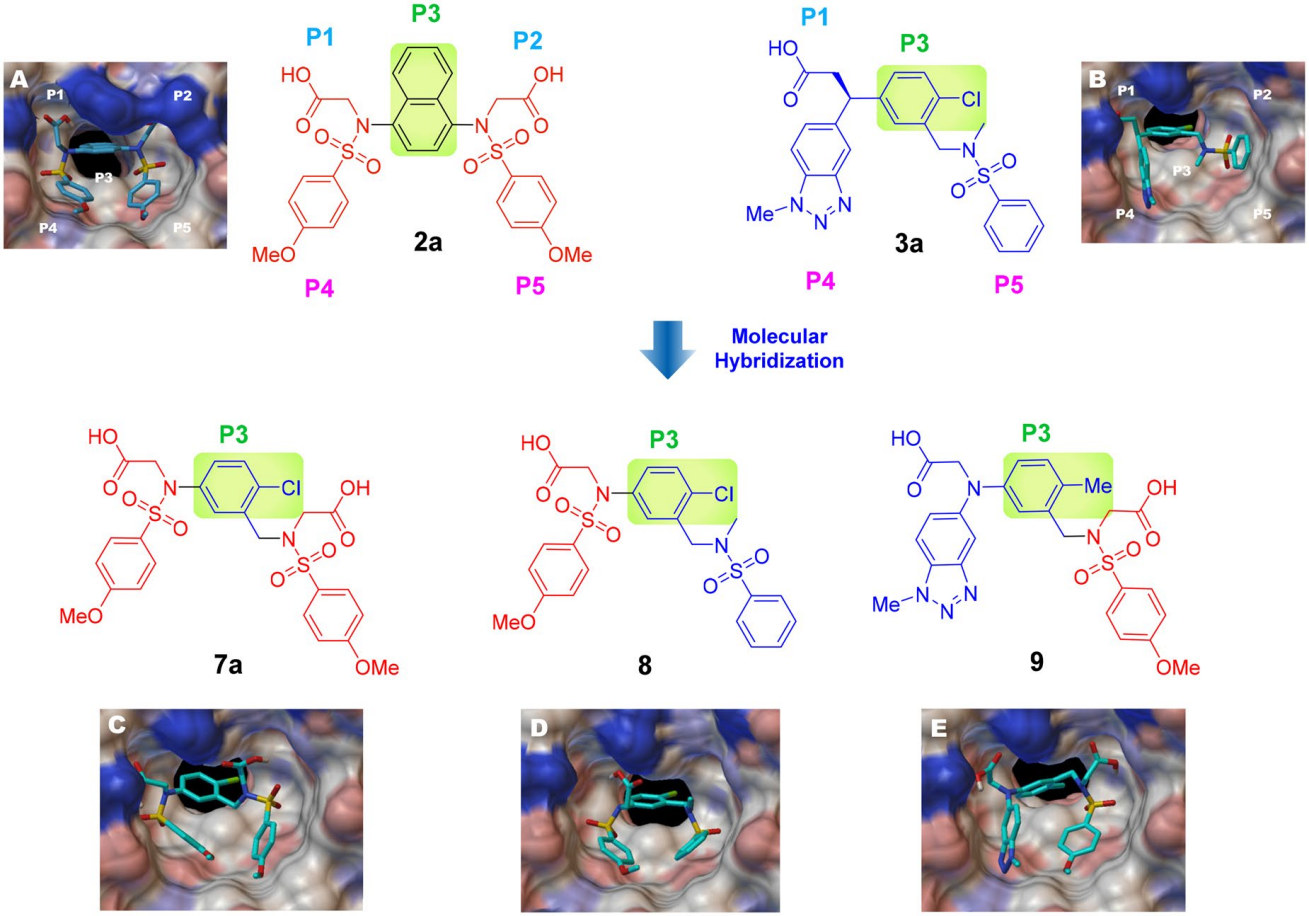
Design strategy for a new meta-substituted bis(arylsulfonamido)benzene scaffold based on known compounds **2a** and **3a** *via* a molecular hybridisation strategy. The Kelch domain of Keap1 comprises P1 to P5, with P3 serving as the central cavity occupied by the ligand’s core scaffold (seen in the green box). (A) Binding conformation of **2a** within the Kelch domain active site (based on 4XMB). (B) Crystal structure showing the Kelch domain bound to **3a** (5FNT). Docked poses of the hybrid compounds in the Keap1 Kelch domain binding site (based on PDB code: 5FNT); (C) **7a**, (D) **8**, (E) **9**.

In an effort to design a novel scaffold as a small-molecule agent that non-covalently disrupts the Keap1-Nrf2 binding, we employed a molecular hybridisation strategy, combining key chemical features from different active compounds to create a new hybrid entity^47^. Our approach was guided by the binding modes of two previously identified inhibitors, **2a** and **3a**, within the Keap1 Kelch domain’s binding cavity, which is divided into five subpockets (P1-P5), each comprising distinct residues that contribute to high binding affinity^48^^,^^49^. P1 and P2 are positively charged regions due to the presence of highly conserved Arg residues, whereas P4 and P5 are hydrophobic pockets, and P3 is a central pocket typically occupied by the core moiety or backbone of the substrate. As seen in Figure 2(A), the naphthalene analog **2a**, which exhibited strong potency in our FP assay (IC_50_ = 63.1 nM), was found to occupy all five subpockets^35^. The naphthalene scaffold resides within the P3 subpocket, whereas the two acetate substituents engage the polar regions P1 and P2. Both benzenesulfonamide groups are positioned within the hydrophobic regions P4 and P5. Compound **3a** with an IC_50_ value of 0.27 μM^38^ was found to occupy four subpockets, leaving P2 unoccupied (Figure 2B). Analogous to the binding profile of **2a**, the acetate group and the *N*-methyl sulphonamide occupied P1 and P5, respectively. The 4-chlorophenyl ring core was positioned in P3, while the benzotriazole moiety resided in the lipophilic P4 pocket. On the basis of these binding mode analyses, we selected the para-substituted phenyl ring as the core scaffold and combined active fragments from subpockets P1, P2, P4, and P5 to design novel hybrid molecules **7a**, **8**, and **9**. As outlined in Figure 2, compound **7a** was generated by replacing the naphthalene core of **2a** with the para-chlorophenyl moiety of **3a**. Compound **8** combined the *N*-sulfonylated glycine from **2a** (highlighted in red) with the *N*-methylated sulphonamide and 4-chlorophenyl groups from **3a** (highlighted in blue). Finally, compound **9** was designed by combining the *N*-sulfonylated glycine of **2a** (red) with the *N*-(1-methyl-1,2,3-benzotriazol-5-yl)glycine and 4-methylphenyl groups of **3a** (blue).

**Figure 3.**
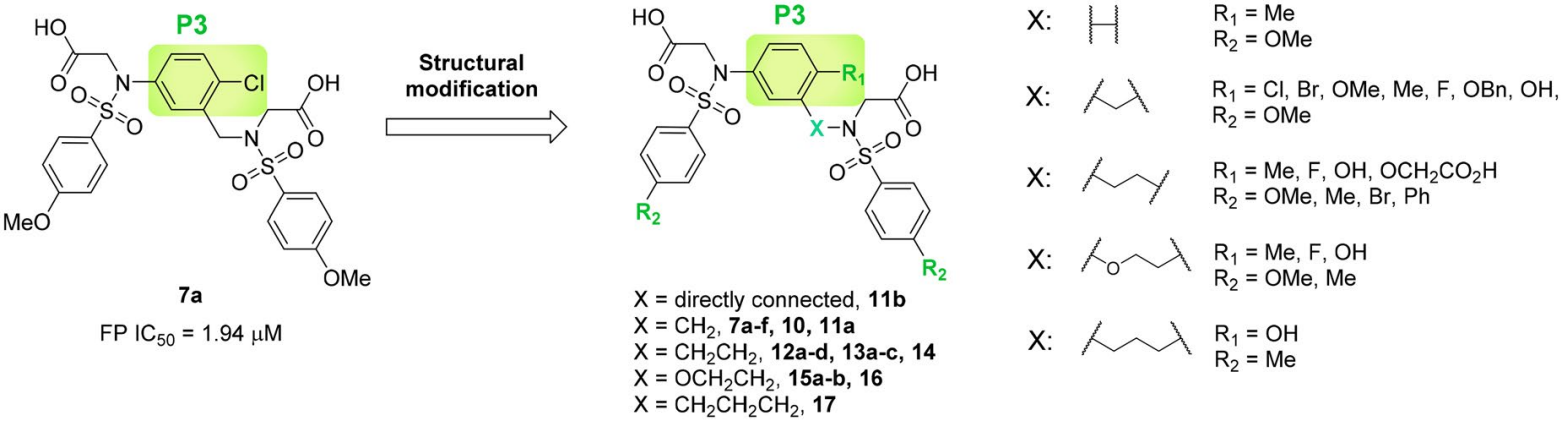
Optimisation of **7a** through modifications on the core (R_1_), benzenesulfonamide (R_2_), and the linker (X).

Docking analysis confirmed our predictions: the diacetate analogs **7a** and **9** occupy all five subpockets (Figure 2C and 2E), whereas the *N*-methyl analogue **8** engages four subpockets with the exception of the polar P2 region (Figure 2D). Interestingly, compound **7a** showed promising inhibitory potency as determined by the FP assay (IC_50_ = 1.94 µM), providing a basis for further structural optimisation to enhance its potency. As outlined in Figure 3, we modified the substituents on the core (R_1_), the benzenesulfonamide (R_2_), and the linker (X).

### Chemistry

**Scheme 1.**
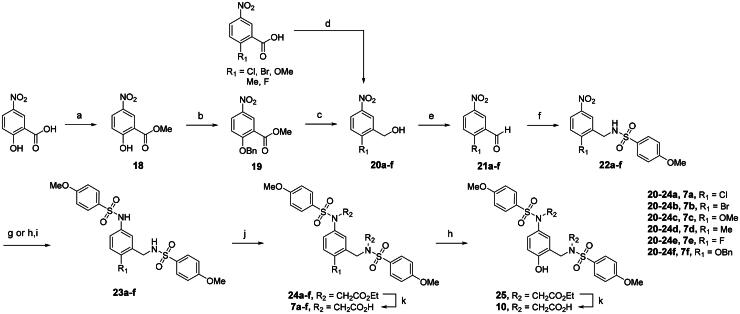
Synthesis of 4-substituted meta-substituted bis(arylsulfonamido)benzene derivatives 7a-f and 10^a^. ^a^Reagents and conditions: (a) SOCl_2_, MeOH, 0 to 70 °C, 77%; (b) BnBr, K_2_CO_3_, DMF, 80 °C, 72%; (c) DIBAL, THF, rt, quantitative; (d) BH_3_, THF, 60 °C, 87% to quantitative; (e) PCC, DCM, rt-55 °C, 56 to 93%; (f) 4-MeOPhSO_2_NH_2_, NaBH(OAc)_3_, TEA, DCM, rt, 18 to 88%; (g) Iron, AcOH, H_2_O, 60–80 °C, 37 to 95% (**23a-b** and **23f**); (h) 10 wt% Pd on carbon, H_2_ (1 atm), MeOH, rt, 75 to 85% (**23c-e** and **25**); (i) 4-MeOPhSO_2_Cl, pyridine, DCM, rt, 13% to quantitative (over two steps); (j) BrCH_2_CO_2_Et, DMF, rt, 76% to quantitative; (k) sodium hydroxide, MeOH, H_2_O, 50–60 °C, 47 to 92%.

The synthesis of compounds **7a**-**f** and **10**, featuring a methylamine linker, was carried out as illustrated in Scheme 1. The primary alcohol intermediates **20a**-**f** were prepared *via* two different synthetic routes. Compound **20f** with a 4-hydroxyl substituent was synthesised in three steps, including methyl esterification of 2-hydroxy-5-nitrobenzoic acid, *O*-benzylation of **18**, and subsequent reduction of the methyl ester to a primary alcohol. In contrast, intermediates **20a**-**e** were obtained by directly reducing the corresponding benzoic acid derivatives using a borane tetrahydrofuran complex solution. Selective oxidation of the alcohols **20a**-**f** to aldehyde analogs **21a**-**f** enabled reductive amination with 4-methoxybenzenesulfonamide, generating **22a**-**f** in 18–88% yield. Catalytic reduction of the nitro substituent in the presence of either a palladium or an iron catalyst, followed by *N*-sulfonylation with 4-methoxybenzenesulfonyl chloride, provided compounds **23a**-**f**. A subsequent two-step sequence, consisting of alkylation with ethyl bromoacetate followed by saponification, afforded the desired products **7a**-**f**. For the synthesis of **10**, the *O*-benzyl protecting group of **24f** was removed using a palladium catalyst, which was then hydrolysed to remove both ethyl esters from the resulting **25**, affording the target compound **10**.

**Scheme 2.**
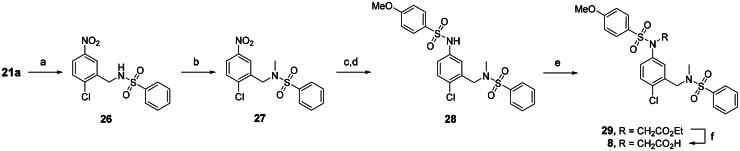
Synthesis of compound 8^a^. ^a^Reagents and conditions: (a) PhSO_2_NH_2_, NaBH(OAc)_3_, TEA, DCM, rt, 46%; (b) MeI, K_2_CO_3_, DMF, rt, 84%; (c) Iron, AcOH, H_2_O, 60 °C, 95%; (d) 4-MeOPhSO_2_Cl, pyridine, DCM, rt, 84%; (e) BrCH_2_CO_2_Et, K_2_CO_3_, DMF, rt, quantitative; (f) sodium hydroxide, MeOH, H_2_O, 60 °C, 80%.

The synthesis of compound **8** began with intermediate **21a**, as shown in Scheme 2. Reductive amination of **21a** with benzenesulfonamide afforded **26**, which was subsequently methylated with methyl iodide to generate **27** in 84% yield. Reducing the nitro group to an amino group enabled *N*-sulfonylation with 4-methoxybenzenesulfonyl chloride, affording sulphonamide **28**. Treatment of **28** with ethyl bromoacetate gave compound **29**, which was finally subjected to saponification to obtain the carboxylic acid analog **8**.

**Scheme 3.**
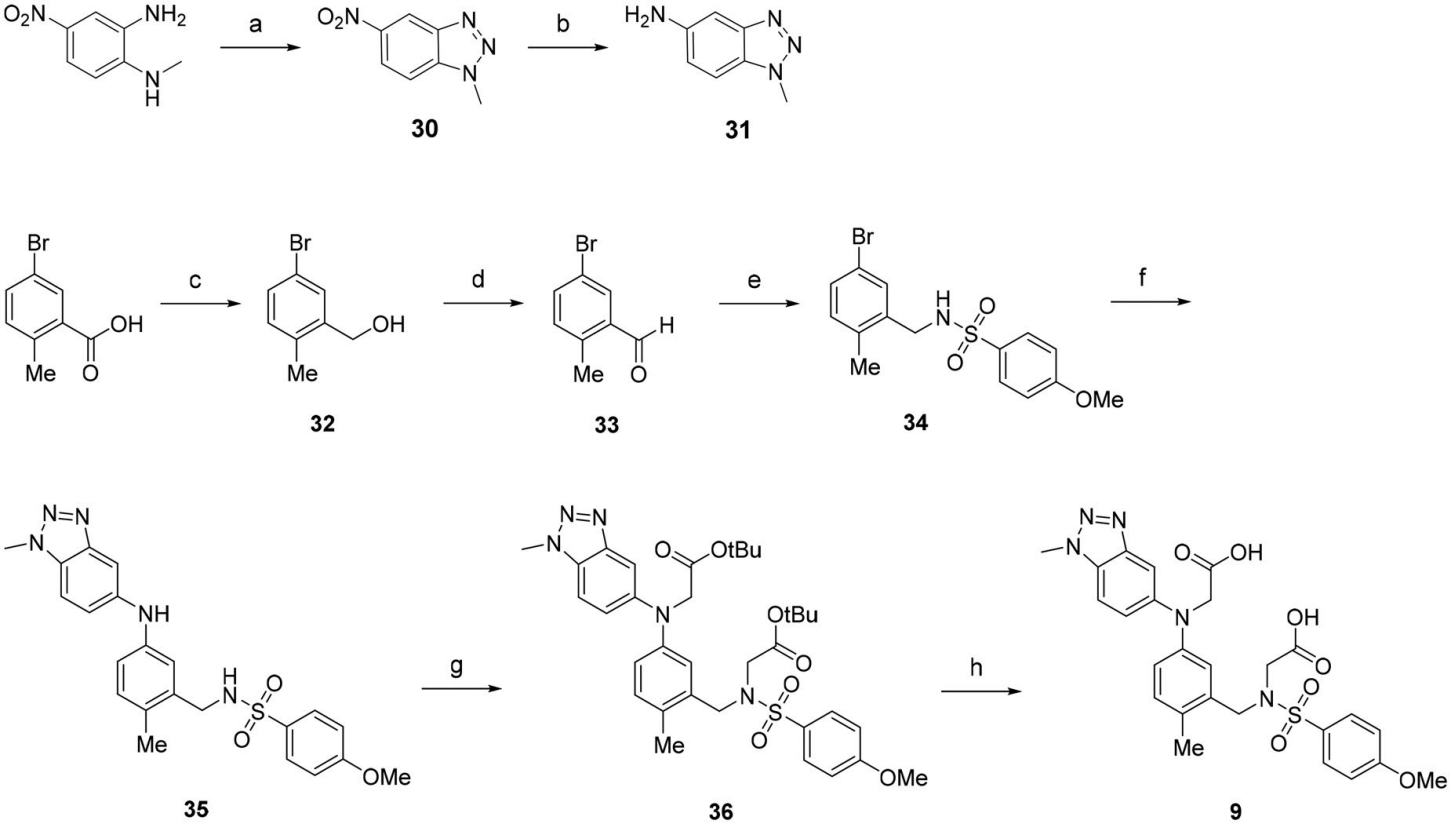
Preparation of compound 9 with a benzotriazole^a^. ^a^Reagents and conditions: (a) NaNO_2_, HCl, H_2_O, 0 °C, 82%; (b) 10 wt% Pd on carbon, H_2_ (1 atm), MeOH, rt, quantitative; (c) BH_3_, THF, 60 °C, 89%; (d) PCC, DCM, rt, 94%; (e) 4-MeOPhSO_2_NH_2_, NaBH(OAc)_3_, NaBH_4_, TEA, DCM, rt, 59%; (f) **31**, Pd_2_(dba)_3_, *t*BuXphos, NaO*t*Bu, toluene, 110 °C, 47%; (g) BrCH_2_CO_2_*t*-Bu, K_2_CO_3_, DMF, rt,4%; (h) Trifluoroacetic acid, DCM, rt, 96%.

The synthetic route of the benzotriazole derivative **9** is outlined in Scheme 3. The amine component **31** for palladium-mediated amination was readily prepared *via* intramolecular cyclisation reaction of *N^1^*-methyl-4-nitro-1,2-phenylenediamine, followed by nitro reduction of **30**. The 1-bromobenzene analogue **34** was synthesised through a synthetic route similar to that used for sulphonamides **22a**-**f**, involving reduction of 5-bromo-2-methylbenzoic acid to alcohol **32**, selective oxidation with PCC, and reductive amination of **33**. Buchwald-Hartwig cross-coupling of **34** with benzotriazole **31** afforded compound **35** in 47% yield, which was subsequently alkylated with *tert*-butyl bromoacetates and subjected to TFA-mediated acid deprotection to provide the final compound **9**.

**Scheme 4.**
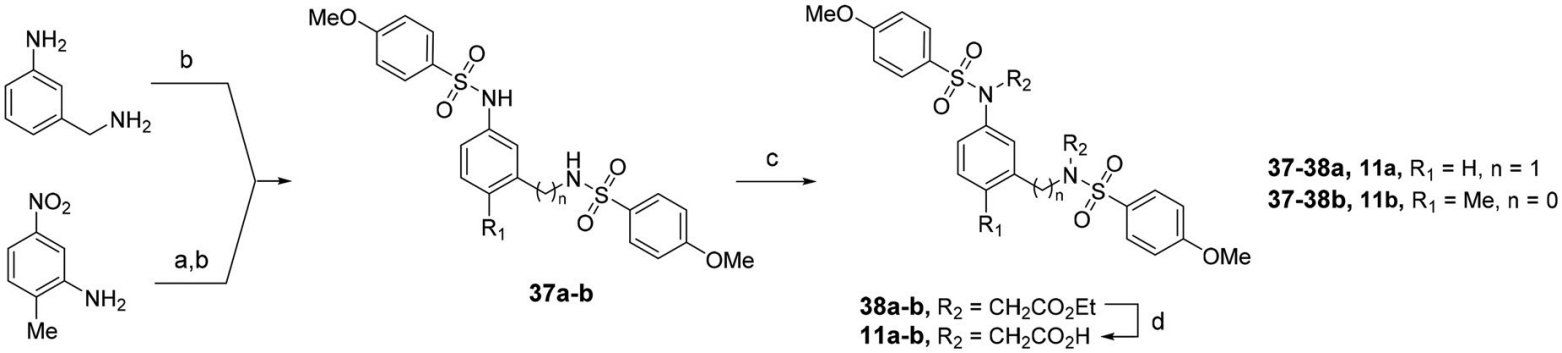
Preparation of meta-substituted bis(arylsulfonamido)benzene derivatives 11a-b from diamine compounds^a^. ^a^Reagents and conditions: (a) 10 wt% Pd on carbon, H_2_ (1 atm), MeOH, rt, quantitative; (b) 4-MeOPhSO_2_Cl, pyridine, DCM, rt, 33 to 61%; (c) BrCH_2_CO_2_Et, K_2_CO_3_, DMF, rt, 71% to quantitative; (d) sodium hydroxide, MeOH, H_2_O, 60 °C, 46 to 64%.

Compounds **11a** and **11b** were obtained through a simplified synthetic route as depicted in Scheme 4. The starting material, 2-methyl-5-nitroaniline, was first reduced under a hydrogen atmosphere. The resulting diamine and 3-(aminomethyl)aniline were reacted with 4-methoxybenzenesulfonyl chloride to produce **37a**-**b** in 33–61% yield. Ethyl bromoacetate was introduced in a subsequent alkylation step, and the diesters **38a**-**b** were converted to the corresponding carboxylic acid analogs **11a**-**b** *via* saponification.

**Scheme 5.**
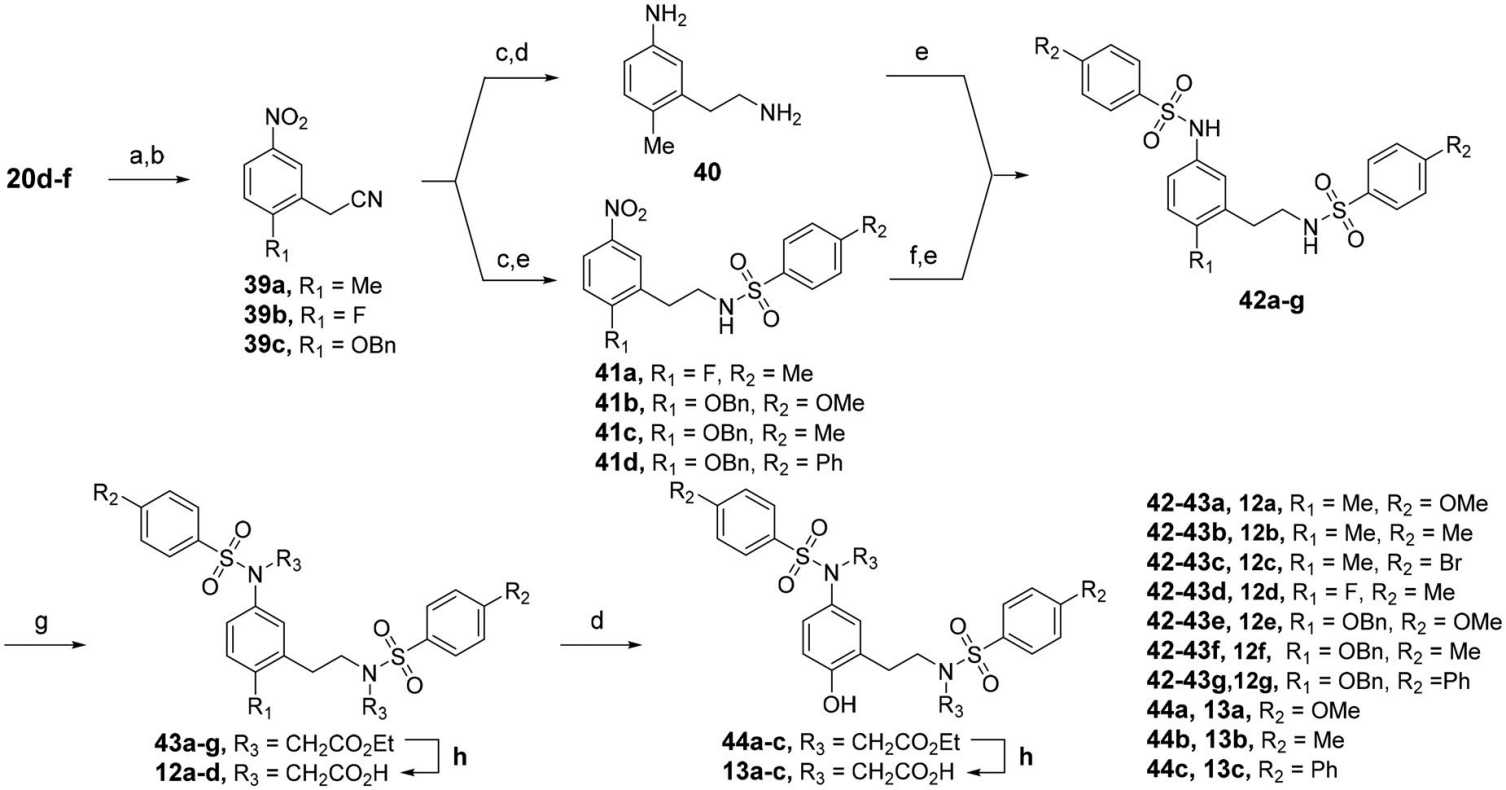
Synthesis of meta-substituted bis(arylsulfonamido)benzene derivatives 12a-d and 13a-c with *N*-ethylamine linker^a^. ^a^Reagents and conditions: (a) TEA, MsCl, DCM, 0 °C, 49% to quantitative; (b) KCN, ACN, rt, 37 to 90%; (c) BH_3_, THF, 60–80 °C, quantitative; (d) 10 wt% Pd on carbon, H_2_ (1 atm), MeOH, rt, 45% to quantitative; (e) R_2_PhSO_2_Cl, pyridine, DCM, rt, 13 to 94% (over two steps); (f) iron, AcOH, H_2_O, 60 °C; (g) BrCH_2_CO_2_Et, K_2_CO_3_, DMF, rt, 56 to 94%; (i) Sodium hydroxide, MeOH, H_2_O, 60 °C, 42 to 90%.

Analogs **12a**-**d** and **13a**-**c**, containing an ethylamine linker, were synthesised from primary alcohol intermediates **20d-f,** as outlined in Scheme 5. Mesylation of **20d**-**f** followed by nucleophilic displacement with KCN afforded nitriles **39a-c**, extending the linker by one carbon. The methyl-substituted **42a** was obtained *via N*-sulfonylation of the diamine derivative **40**, which was synthesised in two steps: nitrile reduction of **39a** to an amine group and subsequent nitro reduction. Alternatively, compounds **42b**-**g** were synthesised through a four-step sequence, in which two sulphonamide moieties were introduced sequentially. The first sulphonamide was formed following nitrile reduction of **39b**-**c**, and the second was introduced after iron-catalyzed nitro reduction of **41a**-**c**. Two acetate groups were introduced into the resulting intermediates **42a**-**g**, and the derived intermediates **43a**-**d** underwent base-mediated hydrolysis to give analogs **12a**-**d**. Additionally, benzylation of **43e**-**g**, alkylation of the intermediates **44a**-**c** with ethyl bromoacetate, and final saponification yielded the final compounds **13a**-**c**.

**Scheme 6.**
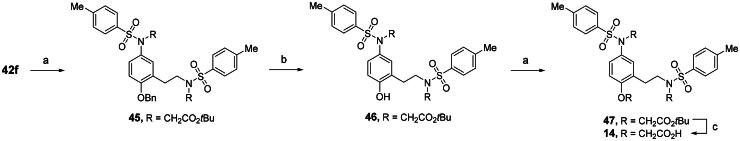
Preparation of meta-substituted bis(arylsulfonamido)benzene analogs 14 with an additional acetate group^a^. ^a^Reagents and conditions: (a) BrCH_2_CO_2_*t*-Bu, K_2_CO_3_, DMF, rt, 96%; (b) 10 wt% Pd on carbon, H_2_ (1 atm), MeOH, rt, 90%; (c) trifluoroacetic acid, DCM, rt, 84%.

Starting from **42f**, compound **14** was prepared in a four-step sequence as depicted in Scheme 6. The addition of acetates to the sulphonamides provided **45**, which underwent hydrogenolysis in the presence of a palladium catalyst, thereby cleaving the benzyl protecting group to yield **46**. The introduction of an additional acetate to the hydroxyl group yielded compound **47**. Finally, TFA treatment removed the three *t*-butyl protecting groups, giving the desired compound **14**.

**Scheme 7.**
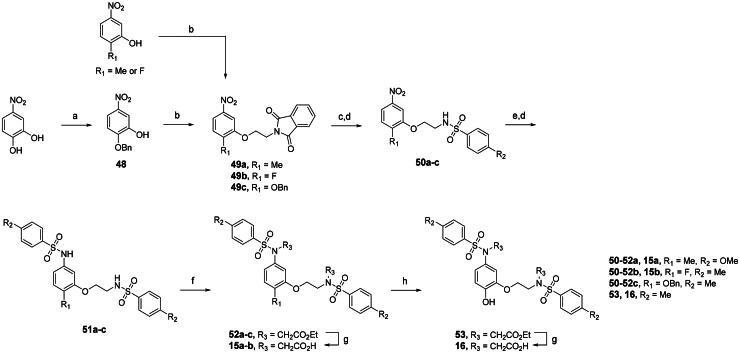
Synthesis of meta-substituted bis(arylsulfonamido)benzene derivatives 15a-b and 16 with *N*-ethanolamine linker^a^. ^a^Reagents and conditions: (a) BnBr, NaH (1 eq), THF, 0 °C to rt, 76%; (b) 2-bromoethyl phthalimide, K_2_CO_3_, DMF, rt-70 °C, 12 to 55%; (c) hydrazine monohydrate, DCM, MeOH, rt-60 °C; (d) R_2_PhSO_2_Cl, pyridine, DCM, rt, 28 to 89% (over two steps); (e) iron, AcOH, H_2_O, 60 °C; (f) BrCH_2_CO_2_Et, K_2_CO_3_, DMF, rt, 66 to 90%; (g) sodium hydroxide, MeOH, H_2_O, 60 °C, 71 to 76%; (h) 10 wt% Pd on carbon, H_2_ (1 atm), MeOH, rt, 91%.

The synthesis of ethanolamine analogs **15a**-**b** and **16** was accomplished as presented in Scheme 7. The phthalimide-protected intermediates **49a**-**c** were prepared by reacting 2-substituted-5-nitrophenol derivatives with 2–(2-bromoethyl)isoindoline-1,3-dione. One of the phenol analogs, compound **48**, was selectively obtained by benzylation of 4-nitrobenzene-1,2-diol in the presence of 1.0 equivalent of NaH^50^. Deprotection of phthalimide derivatives **49a-c** with hydrazine, followed by *N*-sulfonylation, produced intermediates **50a**-**c** in 28–89% yield over two steps. After that, **50a**-**c** were converted into diacetate derivatives **52a**-**c** in a three-step sequence involving iron-mediated nitro reduction, additional sulphonamide formation using 4-methoxybenzenesulfonyl chloride, and alkylation with ethyl bromoacetate to generate the diesters **52a**-**c** in 66–90% yield. Finally, compounds **15a**-**b** were obtained by saponification of **52a**-**b**, while **16** was generated by debenzylating **52c** and subsequently saponifying it.

**Scheme 8.**
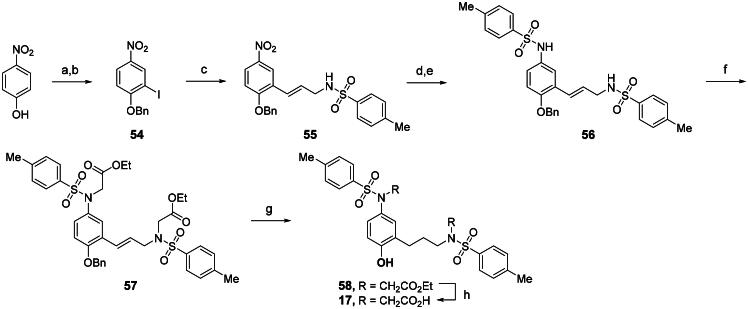
Synthesis of meta-substituted bis(arylsulfonamido)benzene derivative 17 with *N*-propylamine linker^a^ ^a^Reagents and conditions: (a) potassium iodide, KIO_3_, conc.HCl, MeOH, H_2_O, rt; (b) benzyl bromide, K_2_CO_3_, DMF, 80 °C, 12% (over two steps); (c) Pd(OAc)_2_, TEA, *N*-allyl-*p*-toluenesulfonamide, P(*o*-tolyl)_3_, ACN, 80 °C, 45%; (d) iron, AcOH, H_2_O, 60 °C; (e) 4-MePhSO_2_Cl, pyridine, DCM, rt, 48% (over two steps); (f) BrCH_2_CO_2_Et, K_2_CO_3_, DMF, rt, 97%; (g) 10 wt% Pd on carbon, H_2_ (1 atm), MeOH, rt, 57%; (h) sodium hydroxide, MeOH, H_2_O, 60 °C, 56%.

The synthesis of the propylamine analog **17** was carried out as shown in Scheme 8. 1-(Benzyloxy)-2-iodo-4-nitrobenzene (**54**) was synthesised by selective iodination at the 2-position^51^, followed by benzylation. Heck coupling of the 2-iodo substituted **54** with *N*-allyl-*p*-toluenesulfonamide in the catalytic system of P(O-tolyl)_3_ and Pd(OAc)_2_ produced alkene derivative **55**^52^. A two-step process, involving iron-mediated nitro reduction followed by sulfonylation with 4-methoxybenzenesulfonyl chloride, yielded compound **56**, which was subsequently alkylated with ethyl bromoacetate to give diacetate-containing derivative **57**. The *O*-benzyl protecting group was removed by hydrogenolysis, and final saponification of the resulting **58** produced the target compound **17**.

### Biological evaluation in the FP assay and exploration of SAR profiles

To determine the inhibition effect of our designed compounds against the Keap1-Nrf2 binding, FP assay experiments were conducted according to our previously described method^53^. Specifically, the FP method evaluates whether test compounds can replace a fluorescein-tagged 9-mer peptide (originating from the ETGE sequence of Nrf2) from the Keap1 Kelch pocket. Initial screening was performed at three concentrations (50, 5, and, where indicated, 0.5 μM). Compounds exceeding 50% inhibition at 5 μM were advanced for IC_50_ determination.

Table 1 shows that compound **7a** exhibited promising potency, achieving an IC_50_ of 1.91 μM. In contrast, compounds **8** and **9** were largely inactive, showing only 48–53% inhibition at 50 μM. Based on these findings, we selected **7a** as a suitable starting point for a preliminary structure-activity relationship (SAR) study.

**Table 1.** Inhibitory activity of hybrid compounds and meta-substituted bis(arylsulfonamido)benzene derivatives^a^.

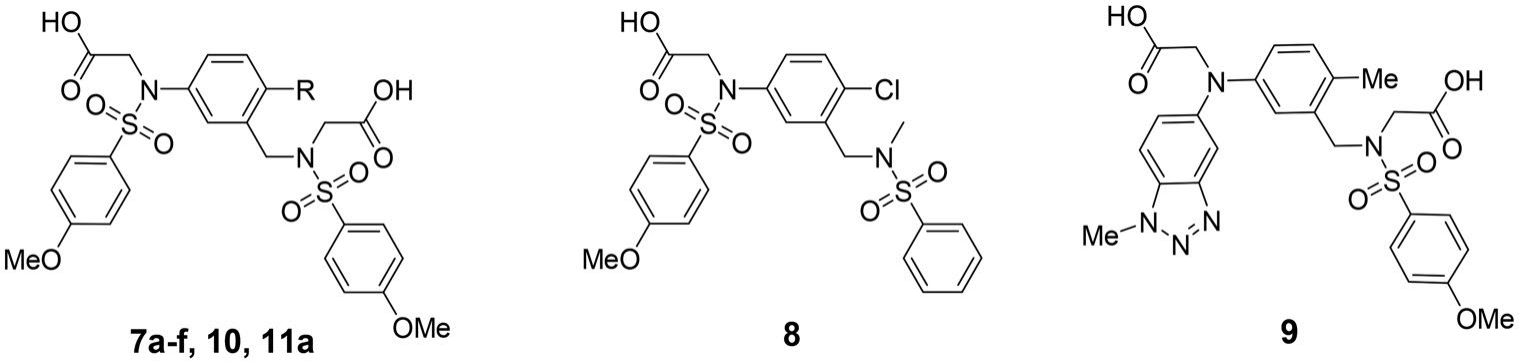						
Compd	R	% inhibition			IC_50_ (µM)^b^	*K_i_* (µM)^c^
		50 µM	5 µM	0.5 µM		
7a	Cl	96	68	ND	1.91 ± 0.50	0.38
7b	Br	85	46	4	ND	ND
7c	OMe	78	39	8	ND	ND
7d	Me	91	42	11	10.03 ± 3.45	2.06
7e	F	111	82	28	1.54 ± 0.27	0.30
7f	OBn	64	16	3	ND	ND
8	–	53	15	ND	ND	ND
9	–	48	13	ND	ND	ND
10	OH	109	78	28	1.08 ± 0.40	0.21
11a	H	85	41	ND	ND	ND

^a^ Inhibitory potency was determined using an FP assay. ND, not determined.

^b^ IC_50_ values represent the average ± SEM derived from triplicate assays.

^c^ *K_i_* values were estimated on the basis of FP-derived IC_50_ results.^53^

On the basis of the chemical structure of **7a** (IC_50_ = 1.91 μM, *K_i_* = 0.38 μM), our optimisation efforts were primarily directed towards the chloro substituent attached at the para site of the phenyl ring, as summarised in Table 1. Both larger bromo (**7b**) and electron-donating methoxy (**7c**) substituents displayed only modest inhibition at 5 µM (46% and 39% inhibition, respectively), suggesting limited activity. Interestingly, the electron-donating methyl group (**7d**) exhibited weaker inhibitory activity than **7a**, with approximately a five-fold decrease in potency (IC_50_ = 10.03 μM, *K_i_* = 2.06 μM).

In addition, the smaller 4-fluoro substituent (**7e**) retained potency in the FP assay (IC_50_ = 1.54 μM, *K_i_* = 0.30 μM), whereas incorporation of a bulky benzyl group (**7f**) resulted in a marked reduction in potency (64% inhibition at 50 μM). With respect to the R modification, the hydroxyl-substituted analog (**10**) ranked among the most potent (IC_50_ = 1.08 μM, *K_i_* = 0.21 μM), along with **7a**, suggesting that the hydroxyl group may contribute to polar contacts with critical residues inside the binding cleft. However, the unsubstituted analogue (**11a**) exhibited limited inhibitory activity, with 41% inhibition at a concentration of 5 μM.

Next, we investigated the effect of linker length (X) and the substituents R_1_ and R_2_ on their inhibitory effect against Keap1-Nrf2 binding, and the results are summarised in Table 2. We hypothesised that the linker length is a critical factor influencing molecular conformation and could be optimised for different R_1_ groups such as methyl, fluoro, or hydroxyl. Increasing linker length generally enhanced inhibitory potency for compounds with a methyl group at the R_1_ position. For example, compounds with no linker (**11b**) or a short methylene linker (**7d**) showed reduced activity in the FP assay (**11b**: 25% inhibition at 5 μM; **7d**: 42% inhibition at 5 μM, IC_50_ = 10 μM, *K_i_* = 3.45 μM). In contrast, the longer ethylene (**12a**) and ethyleneoxy (**15a**) linkers resulted in increased potency (**12a**: IC_50_ = 7.68 μM, *K_i_* = 1.57 μM; **15a**: IC_50_ = 4.29 μM, *K_i_* = 0.87 μM). Notably, compound **15a** with the longest ethyleneoxy linker was approximately 2-fold more potent than **7d** with a methylene linker. Interestingly, the effect of linker length was dependent on the R_1_ substituent. Unlike the trend observed with the 4-methyl group, increasing the linker length from ethylene (**12d**) to ethyleneoxy (**15b**) when R_1_ is a fluoro group led to a decrease in potency (**12d**: IC_50_ = 6.13 μM, *K_i_* = 1.25 μM vs **15b**: IC_50_ = 10.85 μM, *K_i_* = 2.23 μM). When the R_1_ group is hydroxyl, the ethylene linker was found to be optimal. This was supported by two independent SAR analyses. First, extending the linker from methylene (**10**) to ethylene (**13a**) led to a twofold enhancement in potency (**10**: IC_50_ = 1.08 μM, *K_i_* = 0.21 μM, and **13a**: IC_50_ = 0.44 μM, *K_i_* = 0.08 μM). Conversely, longer linkers, such as ethyleneoxy (**16**) and propylene (**17**), resulted in decreased potency compared to the optimal ethylene linker (**13b**, IC_50_ = 0.18 μM, *K_i_* = 0.02 μM vs **16**: IC_50_ = 1.39 μM, *K_i_* = 0.27 μM; **17**: IC_50_ = 0.96 μM, *K_i_* = 0.18 μM).

**Table 2.** Inhibitory activity of meta-substituted bis(arylsulfonamido)benzene derivatives^a^.

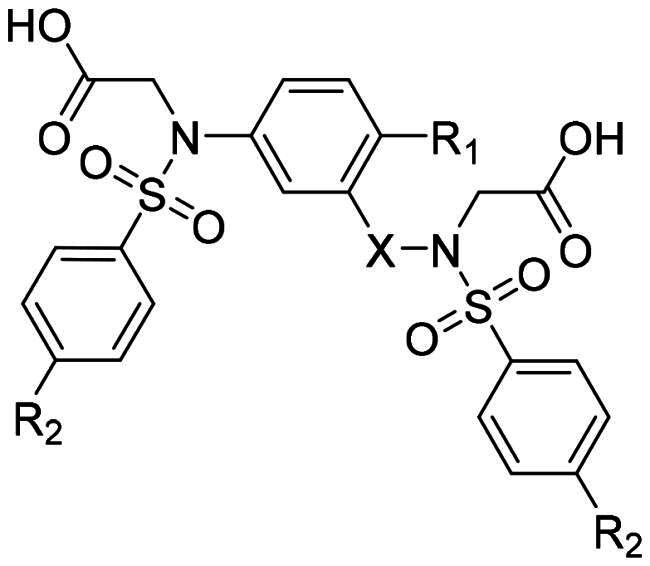								
compd	X	R_1_	R_2_	% inhibition			IC_50_ (µM)^b^	*K_i_* (µM)^c^
				50 µM	5 µM	0.5 µM		
11b	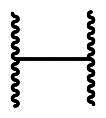	Me	OMe	80	25	ND	ND	ND
12a	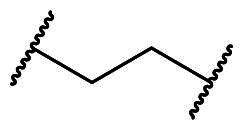	Me	OMe	99	61	28	7.68 ± 2.73	1.57
12b	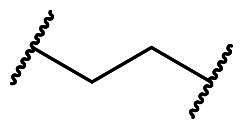	Me	Me	90	57	25	5.22 ± 1.19	1.06
12c	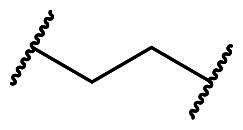	Me	Br	93	50	7	ND	ND
12d	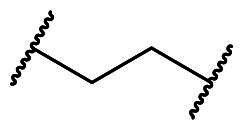	F	Me	94	49	ND	6.13 ± 1.61	1.25
13a	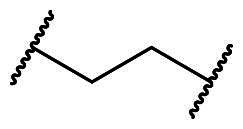	OH	OMe	112	107	83	0.44 ± 0.13	0.08
13b	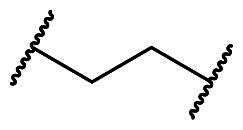	OH	Me	107	93	101	0.18 ± 0.03	0.02
13c	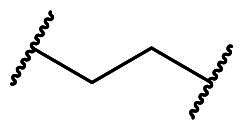	OH	Ph	109	87	ND	1.13 ± 0.27	0.22
14	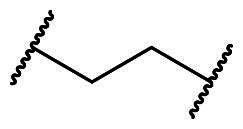	OCH_2_CO_2_H	Me	107	47	29	ND	ND
15a	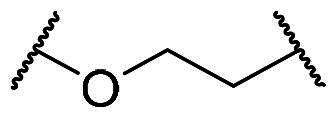	Me	OMe	98	61	ND	4.29 ± 1.39	0.87
15b	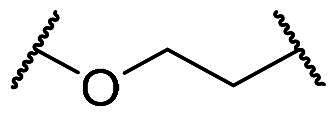	F	Me	90	41	ND	10.85 ± 7.05	2.23
16	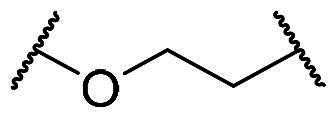	OH	Me	87	71	ND	1.39 ± 0.15	0.27
17	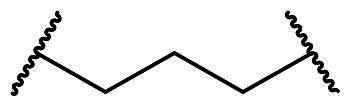	OH	Me	104	89	ND	0.96 ± 0.28	0.18

^a^ Inhibitory potency was determined using an FP assay. ND, not determined.

^b^ IC_50_ values represent the average ± SEM derived from triplicate assays.

^c^ *K_i_* values were estimated on the basis of FP-derived IC_50_ results.

Importantly, our study consistently found that a hydroxy group at the R_1_ position conferred the greatest potency across different linker lengths, suggesting it is crucial for engagement with the Keap1 Kelch domain residues. For instance, compound **13b**, with a hydroxy group at R_1_, was significantly more potent than analogues with methyl (**12b**) or fluoro (**12d**) groups at the same position (**13b**: IC_50_ = 0.18 μM, *K_i_* = 0.02 μM) vs **12b**: IC_50_ = 5.22 μM, *K_i_* = 1.06 μM; **12d**: IC_50_ = 6.13 μM, *K_i_* = 1.25 μM). Furthermore, adding a bulky acetate group to the 4-hydroxyl group (**14**) led to a loss of activity, with only 47% inhibition observed at 5 μM. Similarly, among analogs with a methyl group at R_2_ and an ethyleneoxy linker, **16** with a 4-hydroxy group was more potent than **15b** with a 4-methyl group (**16**: IC_50_ = 1.39 μM, *K_i_* = 0.27 μM vs **15b**: IC_50_ = 10.85 μM, *K_i_* = 2.23 μM). Similar SAR trends are observed in compounds with a methylene linker.

**Figure 4.**
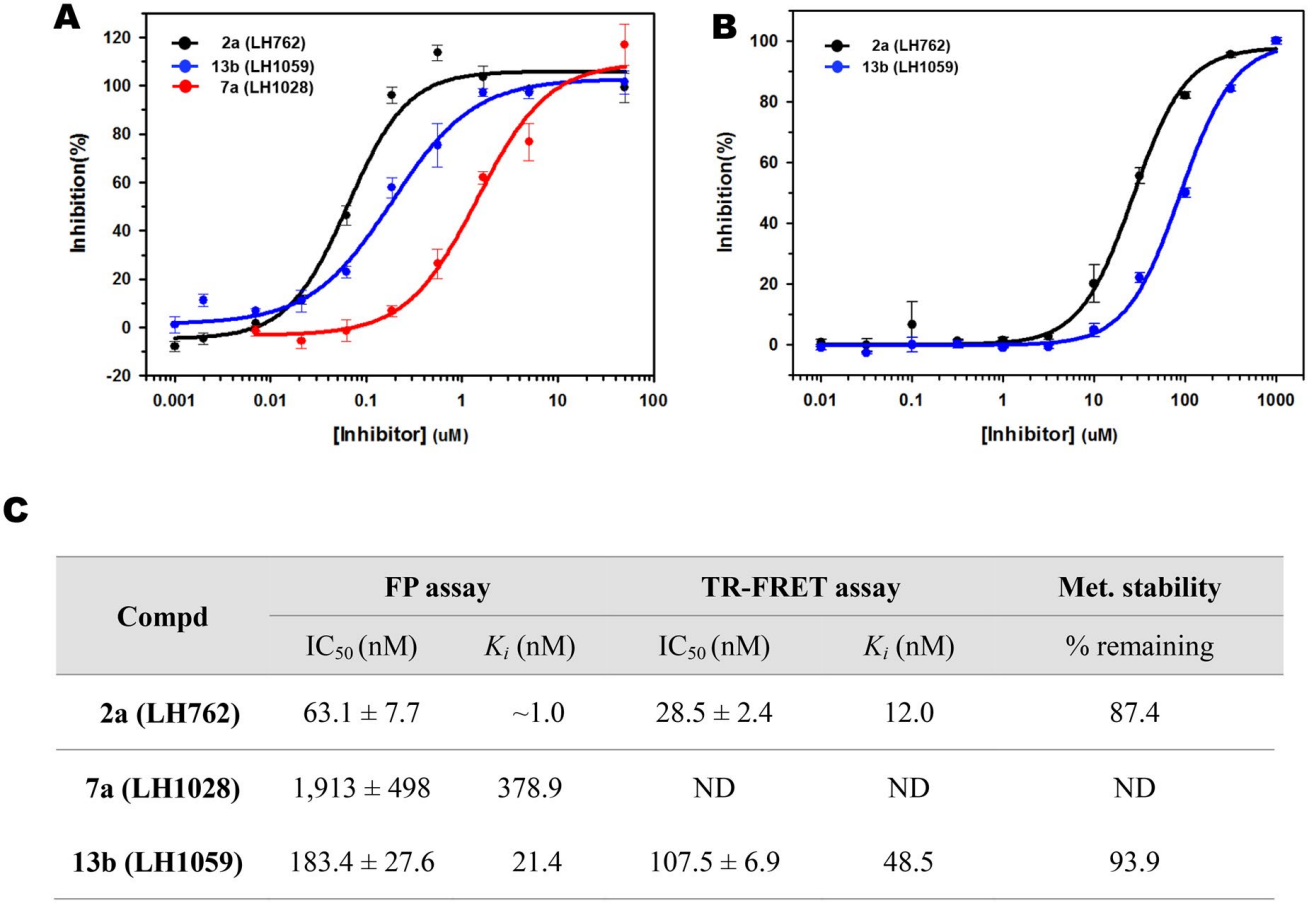
Inhibitory activity and metabolic stability of **2a**, **7a**, and/or **13b**. Concentration-response curves of **2a** (reference compound), **7a**, and **13b** in FP (A) and/or TR-FRET (B) experiments. (C) IC_50_ and *K_i_* parameters determined through TR-FRET and FP analyses, and % remaining after 30 min using human liver microsomal incubation.

For the R_2_ substituents on the benzenesulfonamide moiety, the methyl group consistently led to higher potency. Specifically, various R_2_ substituents, including methoxy (**12a**), methyl (**12b**), and bromo (**12c**) on the 4-methylphenyl core, were generally tolerated. However, in the 4-hydroxyphenyl core series, the methyl substituent in **13b** conferred superior potency compared to the methoxy in **13a** and phenyl in **13c** (**13b**: IC_50_ = 0.18 μM, *K_i_* = 0.02 μM, **13a**: IC_50_ = 0.44 μM, *K_i_* = 0.08 μM, and **13c**: IC_50_ = 1.13 μM, *K_i_* = 0.22 μM). Taken together, compound **13b** emerged as the most potent meta-substituted bis(arylsulfonamido)benzene analog, demonstrating a 10-fold improvement in potency relative to the initial lead compound **7a** (Figure 4C).

### Biological assessment of compound 13b utilising a TR-FRET assay

In this study, we employed the FP assay as a primary tool to estimate hybrid compounds’ inhibitory potency and investigate the SAR around compound **7a**. We conducted a confirmatory TR-FRET assay to assess compounds with submicromolar potency (IC_50_ < 0.2 μM). This proximity-based assay relies on distance-dependent energy transfer from a donor fluorophore (terbium-labeled Keap1 Kelch domain) to an acceptor fluorophore. The competitive inhibition by **13b** was evaluated by measuring the emission intensities at 495 nm and 520 nm, respectively. The previously reported Keap1–Nrf2 PPI inhibitor **2a** was included as a positive reference compound in the TR-FRET assay to validate assay performance and to provide a benchmark for comparison. As a result, the 4-hydroxyphenyl analog **13b** displayed a similar IC_50_ in the TR-FRET measurement (IC_50_ = 107.5 nM, *K_i_* = 48.5 nM) to that observed in the FP assay (IC_50_ = 183.4 nM, *K_i_* = 21.4 nM), as shown in Figure 4.

### Metabolic stability of compound 13b in human liver microsomes

Given the importance of metabolic stability in early drug discovery, we assessed the metabolic profile of the most potent compound in this series, **13b**, using human liver microsomes. After 30 min of incubation, the meta-substituted bis(arylsulfonamido)benzene analogue **13b** remained highly stable, with 93.9% of the parent compound remaining. This value was numerically higher than that of the positive control compound **2a** (87.4% remaining), indicating that **13b** exhibits superior metabolic stability under the same conditions (Figure 4). The high metabolic stability of **13b** in human liver microsomes suggests that the meta-substituted bis(arylsulfonamido)benzene analog may possess favourable metabolic stability, indicating its potential advantage for further development.

### Molecular docking analysis

**Figure 5.**
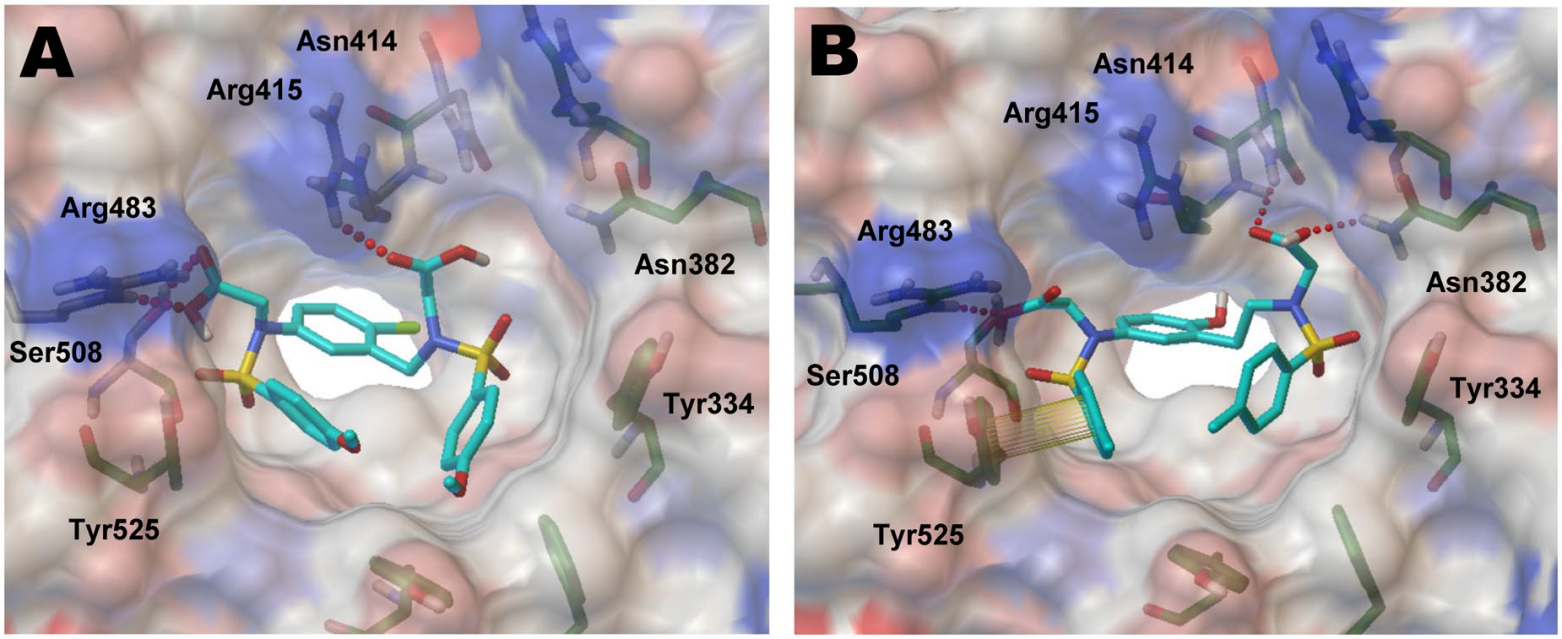
Predicted binding conformations of compounds **7a** (A) and **13b** (B) (shown as cyan sticks), docked into Keap1 Kelch domain (PDB: 5FNT); The protein surface was mapped according to partial charge distribution (red: hydrophobic regions; blue: polar regions). Hydrogen bonding interactions are indicated with red dashed lines, with the corresponding bond distances and angles discussed in the text, while yellow lines illustrate π-related contacts (π-π stacking or cation-π).

The Kelch domain of Keap1 adopts a characteristic six-bladed β-propeller architecture, creating a well-defined binding cavity that recognises the ETGE motif of Nrf2. This binding site is typically organised into five subpockets (P1-P5), each possessing distinct physicochemical properties that collectively govern ligand recognition. Among these, P1 and P2 are characterised by clusters of conserved arginine residues and thus exhibit a positively charged environment, whereas P3 functions as a central pocket that accommodates the core scaffold. In contrast, P4 and P5 primarily form hydrophobic regions that facilitate nonpolar interactions with ligand substituents. To investigate the structural binding modes of the hybrid molecules **7a** and **13b**, the docking analysis was carried out using the co-crystallized structure of **3a** bound to the Keap1 (PDB: 5FNT). Compound **2a**, a well-characterised Keap1-Nrf2 PPI inhibitor, was used as a reference for comparison of binding modes. As anticipated, the meta-substituted bis(arylsulfonamido)benzene analogs occupy all five subpockets (P1-P5), as illustrated in Figure 5. Docking analysis of **7a**, containing the methylene linker, suggested that one of the acetate groups forms hydrogen bonding interactions with polar Arg483 (distance = 1.98 Å, angle = 154.9°) and Ser508 (2.21 Å, 150.0°) in the P1 subpocket, while the other carboxylic acid engages in a hydrogen bond with Arg415 (2.11 Å, 131.8°) (Figure 5a). Although these interactions fall within acceptable hydrogen-bonding distances, the relatively minor bond angle observed for the Arg415 interaction suggests a less optimal hydrogen-bonding geometry. Moreover, the short methylene linker limits the ability of **7a** to extend towards the P2 subpocket, thereby restricting productive polar interactions. These geometric constraints likely contribute to the moderate inhibitory potency of **7a** (IC_50_ = 1.91 μM, *Kᵢ* = 0.38 μM). In contrast, docking simulation of compound **13b**, featuring a more extended ethylene linker (IC_50_ = 183.4 nM, *K_i_* = 21.4 nM in the FP assay), revealed that its acetate group more effectively reaches into the P2 subpocket. Indeed, **13b** formed hydrogen bonds with Asn414 (2.03 Å, 151.2°) and Asn382 (2.04 Å, 145.7°) in the P2 subpocket, as well as a strong interaction with Arg483 in P1 (1.88 Å, 168.8°). Notably, the near-linear hydrogen bond angle observed for the Arg483 interaction suggests an optimal hydrogen bonding geometry, which is expected to contribute significantly to the binding strength. Additionally, the phenyl ring attached to the sulphonamide engages in π-π stacking interactions with Tyr525 in the P4 subpocket. Collectively, the shorter hydrogen bond distances and more favourable bond angles observed for **13b**, together with its extended reach into the P2 subpocket, provide a structural rationale for its enhanced binding affinity and approximately 11-fold greater potency compared to **7a**.

## Conclusion

In summary, a new set of meta-substituted bis(arylsulfonamido)benzene analogues was designed using a molecular hybridisation strategy, based on the known Keap1-Nrf2 PPI inhibitors **2a** and **3a**. Among the hybrid compounds, **7a** emerged as a promising lead with an IC_50_ of 1.91 μM and a *K_i_* of 0.38 μM in our FP assay. A systematic SAR study was conducted at **7a** by modifying the linker and two substituents (R_1_ and R_2_). This exploration led to three key findings. Firstly, regardless of the linker length and polarity, the hydroxy (OH) group proved to be a superior R_1_ substituent on the phenyl core, conferring greater potency than either fluoro or methyl groups. Compounds **10**, **13a**-**c**, and **16–17**, which have a hydroxy group at the R_1_ position, exhibited potent activity with IC_50_ values consistently below 1.5 μM. Secondly, the ethylene linker provided optimal inhibitory activity. Both shorter and longer linkers resulted in decreased potency. Lastly, the methyl group conferred the highest potency among the R_2_ substituents tested (methyl, methoxy, bromo, and phenyl). This SAR study from **7a** led to the discovery of compound **13b** as the most potent analogue among the meta-substituted bis(arylsulfonamido)benzene derivatives, as confirmed by both FP and TR-FRET assays. Overall, the SAR analysis established clear structure-activity trends that guide further lead optimisation. In particular, appropriate linker length and hydrogen-bonding capability at the R_1_ position were identified as key determinants of potency, while small hydrophobic substituents at the R_2_ position were preferred. These insights offer a rational framework for the continued optimisation of this chemical series. Docking simulations provided insight into the binding mechanism. The methylene linker in **7a** was too short to effectively engage polar residues in the P2 subpocket, while the more extended ethylene linker in **13b** enabled strong hydrogen bonding interactions with key residues (Asn414 and Asn382). Furthermore, compound **13b** demonstrated excellent metabolic stability in human liver microsomes, retaining 93.9% of the parent compound after 30 min of incubation. This is a favourable profile compared to the 1,4-diaminonaphthalene analogue **2a**, which showed slightly lower stability (87.4%). Although the present study establishes **13b** as a potent non-covalent inhibitor of the Keap1-Nrf2 PPI with favourable metabolic stability, further cell-based investigations will be necessary to fully elucidate its ability to activate Nrf2 signalling in cellular contexts. Such studies will be the focus of future work aimed at advancing the optimisation and biological validation of this scaffold. This chemical series represents an attractive starting point for further optimisation and biological evaluation in the development of more potent and metabolically stable direct inhibitors of the Keap1-Nrf2 binding.

## Experimental section

### Chemistry

All solvents and reagents were purchased from commercial suppliers (Merck, Sigma-Aldrich, or equivalent) and used as received unless otherwise specified. Analytical TLC was performed on silica gel 60 F254 aluminum-backed plates (Merck or Sigma-Aldrich) and visualised under UV light (254 nm) or by potassium permanganate staining, followed by heating. Flash column chromatography was performed on a Teledyne ISCO Combiflash Companion system using RediSep silica cartridges (230–400 mesh) with solvent gradients of ethyl acetate/hexane (0–100%) or methanol/dichloromethane (0–20%).^1^H and ^13^C NMR spectra were recorded on a Bruker Avance III 400 MHz spectrometer at room temperature. LC/MS analyses were conducted using an Agilent 1200 Infinity HPLC coupled to an Agilent 6410 ESI-MS system with an Inertsil ODS-3 C18 column (3 mm × 33 mm, 3 μm, GL Sciences) maintained at 40 °C. HRMS data were obtained at the Centre for Integrative Proteomics Research (Rutgers University) on a Thermo LTQ Orbitrap Velos ETD mass spectrometer coupled to a Dionex UltiMate 3000 nano-flow 2D LC system. Compound purity was verified by a Waters ACQUITY UPLC^™^ system.

### General synthetic method for basic deprotection of ethyl ester intermediates (method A)

An intermediate containing ethyl esters (0.16 mmol, 1 equiv.) in MeOH (1.5 ml) was combined with sodium hydroxide (3 equiv.) in H_2_O (0.5 ml) at ambient conditions. The reaction solution was maintained under stirring at 60 °C for 5 h. Upon completion, solvents were removed *in vacuo*, and the crude was dissolved in H_2_O and subsequently extracted with DCM. The water layer was adjusted to pH 1 using 6 N HCl and subsequently extracted with DCM. Drying of the organic layer was carried out with sodium sulphate (anhydrous), and the solvent was evaporated *in vacuo*. The resulting residue was dissolved in a small amount of DCM, precipitated with hexane, and collected by filtration to afford the corresponding final compound.

### General synthetic method for acidic deprotection of tert-butyl ester intermediates (method B)

A solution of intermediate containing *tert*-butyl esters (0.01 mmol, 1 equiv.) in DCM (2 ml) was reacted with TFA (0.4 ml) at ambient conditions, followed by stirring for 8 h. Once the reaction was complete, the crude solution was transferred into water and subjected to extraction with dichloromethane. The organic layer was dried with sodium sulphate (anhydrous) and evaporated *in vacuo*. The obtained solid was redissolved in the least amount of DCM, precipitated with hexane, and isolated by filtration to yield the corresponding final compound.

### Structural characterisation of the final compounds

#### N-(5-((N-(Carboxymethyl)-4-methoxyphenyl)sulfonamido)-2-chlorobenzyl)-N-((4-methoxyphenyl)-sulfonyl)glycine (7a)

Obtained using method A from **24a**; White solid; Yield 84% (84 mg); ^1^H NMR (400 MHz, CDCl_3_) *δ* 7.75 (d, *J* = 8.8 Hz, 2H), 7.57 (d, *J* = 8.8 Hz, 2H), 7.35 (brs, 2H, CO_2_H), 7.27 (d, *J* = 2.4 Hz, 1H), 7.22 (d, *J* = 8.8 Hz, 1H), 7.12 (dd, *J* = 8.8, 2.4 Hz, 1H), 6.97 (d, *J* = 8.8 Hz, 2H), 6.93 (d, *J* = 8.8 Hz, 2H), 4.40 (s, 2H), 4.34 (s, 2H), 3.86 (s, 3H),3.85 (s, 3H), 3.80 (s, 2H); ^13^C NMR (100 MHz, CDCl_3_) *δ* 173.5, 163.5, 163.5, 138.9, 134.1, 133.4, 130.4, 130.2, 130.1, 130.0, 129.8, 129.7, 114.6, 114.5, 55.8, 55.8, 52.2, 49.7, 48.5; LC/ESI-MS: *m/z* 613.1 [M + H]^+^; HRMS (ESI): *m/z* [M + H]^+^ calculated for C_25_H_26_ClN_2_O_10_S_2_: 613.0712; found: 613.0723; UPLC (retention time 3.98 min): purity 96.9%.

#### N-(5-((N-(Carboxymethyl)-4-methoxyphenyl)sulfonamido)-2-bromobenzyl)-N-((4-methoxyphenyl)-sulfonyl)glycine (7b)

Obtained using method A from **24b**; White solid; Yield 47% (12 mg); ^1^H NMR (400 MHz, CDCl_3_) *δ* 7.78 (d, *J* = 8.8 Hz, 2H), 7.59 (d, *J* = 8.8 Hz, 2H), 7.43 (d, *J* = 8.4 Hz, 1H), 7.27 (d, *J* = 2.4 Hz, 1H), 7.08 (dd, *J* = 8.4, 2.4 Hz, 1H), 6.99 (d, *J* = 8.8 Hz, 2H), 6.95 (d, *J* = 8.8 Hz, 2H), 4.38 (s, 2H), 4.36 (s, 2H), 3.88 (s, 3H),3.86 (s, 3H), 3.80 (s, 2H); ^13^C NMR (100 MHz, CDCl_3_) *δ* 173.5, 163.6, 139.7, 135.7, 133.8, 130.2, 130.0, 129.9, 129.8, 123.1, 114.6, 114.5, 55.9, 55.8, 52.3, 52.0, 49.6; LC/ESI-MS: *m/z* 657.1 [M(^79^Br) + H]^+^ and 659.1 [M(^81^Br) + H]^+^; HRMS (ESI): *m/z* [M(^79^Br) + H]^+^ and [M(^81^Br) + H]^+^ calculated for C_25_H_26_BrN_2_O_10_S_2_: 657.0168 and 659.0147; found: 657.0219 and 659.0195; UPLC (retention time 4.02 min): purity 95.1%.

#### N-(5-((N-(Carboxymethyl)-4-methoxyphenyl)sulfonamido)-2-methoxybenzyl)-N-((4-methoxy-phenyl)sulfonyl)glycine (7c)

Obtained using method A from **24c**; White solid; Yield 83% 76 mg); ^1^H NMR (400 MHz, CD_3_OD) *δ* 7.65 (d, *J* = 8.8 Hz, 2H), 7.58 (d, *J* = 8.8 Hz, 2H), 7.22 (dd, *J* = 8.8, 2.4 Hz, 1H), 7.03 (d, *J* = 8.8 Hz, 2H), 6.98 (d, *J* = 8.8 Hz, 2H), 6.84 (d, *J* = 2.4 Hz, 1H), 6.81 (d, *J* = 8.8 Hz, 1H), 4.37 (s, 2H), 4.26 (s, 2H), 3.87 (s, 3H),3.86 (s, 3H), 3.81 (s, 2H), 3.69 (s, 3H); ^13^C NMR (100 MHz, CD_3_OD) *δ* 172.1, 164.8, 164.5, 158.7, 133.8, 133.2, 132.0, 131.6, 131.1, 131.0, 130.5, 125.6, 115.3, 115.2, 111.8, 56.2, 56.2, 56.0, 53.8, 47.3; LC/ESI-MS: *m/z* 607.1 [M + H]^+^; HRMS (ESI): *m/z* [M - H]^-^ calculated for C_26_H_27_N_2_O_11_S_2_: 607.1062; found: 607.1067; UPLC (retention time 3.80 min): purity 100.0%.

#### N-(5-((N-(Carboxymethyl)-4-methoxyphenyl)sulfonamido)-2-methylbenzyl)-N-((4-methoxyphenyl)-sulfonyl)glycine (7d)

Obtained using method A from **24d**; White solid; Yield 75% (61 mg); ^1^H NMR (400 MHz, CDCl_3_) *δ* 9.35 (brs, 2H, CO_2_H), 7.78 (d, *J* = 7.6 Hz, 2H), 7.52 (d, *J* = 7.6 Hz, 2H), 7.08 (s, 1H), 7.04 (d, *J* = 8.0 Hz, 1H), 7.00 (d, *J* = 8.0 Hz, 2H), 6.91–6.86 (m, 3H), 4.34 (s, 2H), 4.30 (s, 2H), 3.87 (s, 3H), 3.85 (s, 3H), 3.68 (s, 2H), 2.29 (s, 3H); ^13^C NMR (100 MHz, CDCl_3_) *δ* 174.24, 174.15, 163.5, 163.4, 138.8, 137.5, 133.4, 131.6, 131.3, 130.1, 130.0, 129.9, 129.8, 127.9, 114.5, 114.3, 55.9, 55.8, 52.2, 50.3, 47.0, 18.8; LC/ESI-MS: *m/z* 591.1 [M - H]^-^; HRMS (ESI): *m/z* [M - H]^-^ calculated for C_26_H_27_N_2_O_10_S_2_: 591.1113; found: 591.1116; UPLC (retention time 3.82 min): purity 98.9%.

#### N-(5-((N-(Carboxymethyl)-4-methoxyphenyl)sulfonamido)-2-fluorobenzyl)-N-((4-methoxyphenyl)-sulfonyl)glycine (7e)

Obtained using method A from **24e**; White solid; Yield 92% (84 mg); ^1^H NMR (400 MHz, DMSO-*d_6_*) *δ* 8.63 (brs, 2H, CO_2_H), 7.74 (d, *J* = 8.8 Hz, 2H), 7.56 (d, *J* = 8.8 Hz, 2H), 7.16–7.15 (m, 2H), 6.98–6.89 (m, 5H), 4.34 (s, 2H), 4.33 (s, 2H), 3.86 (s, 3H),3.85 (s, 3H), 3.81 (s, 2H); ^13^C NMR (100 MHz, DMSO-*d_6_*) *δ* 173.9, 163.53, 163.48, 159.1, 136.0, 131.6, 131.24, 131.15, 130.3, 130.0, 129.8 (*J*_C,F_ = 5 Hz), 123.5 (*J*_C,F_ = 16 Hz), 116.4 (*J*_C,F_ = 23 Hz), 114.6, 114.5, 55.8, 55.8, 52.4, 48.1, 45.7; LC/ESI-MS: *m/z* 595.1 [M - H]^-^; HRMS (ESI): *m/z* [M - H]^-^ calculated for C_25_H_24_FN_2_O_10_S_2_: 595.0862; found: 595.0867; UPLC (retention time 3.86 min): purity 99.7%.

#### N-(4-(benzyloxy)-3-(((N-(carboxymethyl)-4-methoxyphenyl)sulfonamido)methyl)phenyl)-N-((4-methoxyphenyl)sulfonyl)glycine (7f)

Obtained using method A from **24f**; Pale yellow solid; Yield 81% (16 mg); ^1^H NMR (400 MHz, CDCl_3_) *δ* 7.71 (d, *J* = 8.4 Hz, 2H), 7.58 (d, *J* = 8.4 Hz, 2H), 7.34–7.32 (m, 5H), 7.14 (d, *J* = 8.4 Hz, 1H), 7.03 (s, 1H), 6.93–6.90 (m, 4H), 6.79 (d, *J* = 8.4 Hz, 1H), 4.96 (s, 2H), 4.34 (s, 2H), 4.29 (s, 2H), 3.85 (s, 3H), 3.83 (s, 3H), 3.78 (s, 2H); ^13^C NMR (100 MHz, CDCl_3_) *δ* 174.0, 163.4, 163.3, 156.6, 136.3, 132.7, 131.2, 130.9, 130.8, 130.3, 130.0, 129.8, 128.8, 128.4, 127.6, 124.5, 114.4, 114.4, 112.4, 70.6, 55.8, 52.7, 48.4, 47.0; LC/ESI-MS: *m/z* 683.1 [M - H]^-^; HRMS (ESI): *m/z* [M - H]^-^ calculated for C_32_H_31_N_2_O_11_S_2_: 683.1375; found: 683.1382; UPLC (retention time 4.23 min): purity 99.7%.

#### N-(4-Chloro-3-((N-methylphenylsulfonamido)methyl)phenyl)-N-((4-methoxyphenyl)sulfonyl)glycine (8)

Obtained using method A from **29**; Pale yellow solid; Yield 80% (54 mg); ^1^H NMR (400 MHz, CDCl_3_) *δ* 7.82 (d, *J* = 8.0 Hz, 2H), 7.66–7.56 (m, 5H), 7.31 (d, *J* = 8.4 Hz, 1H), 7.27–7.24 (m, 1H), 7.20 (s, 1H), 6.96 (d, *J* = 8.0 Hz, 2H), 4.41 (s, 2H), 4.19 (s, 2H), 3.88 (s, 3H), 2.56 (s, 3H); ^13^C NMR (100 MHz, CDCl_3_) *δ* 173.4, 163.5, 139.1, 137.2, 134.6, 133.3, 133.1, 130.6, 130.1, 130.0, 129.9, 129.4, 129.1, 127.5, 114.5, 55.8, 52.3, 51.1, 35.2; LC/ESI-MS: *m/z* 539.1 [M + H]^+^; HRMS (ESI): *m/z* [M + H]^+^ calculated for C_23_H_24_ClN_2_O_7_S_2_: 539.0708; found: 539.0721; UPLC (retention time 4.48 min): purity 96.9%.

#### N-(5-((Carboxymethyl)(1-methyl-1H-benzo[d][1,2,3]triazol-5-yl)amino)-2-methylbenzyl)-N-((4-methoxyphenyl)sulfonyl)glycine (9)

Obtained using method B from **36**; Pale yellow solid; Yield 96% (7 mg); ^1^H NMR (400 MHz, CDCl_3_) *δ* 7.80–7.74 (m, 2H), 7.70 (d, *J* = 8.4 Hz, 1H), 7.40–7.37 (m, 1H), 7.23 (d, *J* = 9.2 Hz, 1H), 7.19–7.16 (m, 1H), 6.99 (d, *J* = 8.4 Hz, 1H), 6.95–6.93 (m, 1H), 6.92–6.89 (m, 2H), 4.43 (s, 1H), 4.23 (s, 3H), 3.90 (s, 2H), 3.83 (s, 1H), 3.81 (s, 3H), 3.31 (s, 2H), 2.32 (s, 3H); ^13^C NMR (100 MHz, CDCl_3_) *δ* 163.4, 163.1, 146.7, 133.0, 132.2, 131.0, 129.8, 129.7, 129.6, 124.5, 123.0, 120.9, 114.4, 114.2, 110.2, 77.4, 55.9, 55.7, 49.8, 40.5, 18.3; LC/ESI-MS: *m/z* 554.2 [M + H]^+^; HRMS (ESI): *m/z* [M + H]^+^ calculated for C_26_H_28_N_5_O_7_S: 554.1704; found: 554.1714; UPLC (retention time 4.13 min): purity 91.9%.

#### N-(5-((N-(Carboxymethyl)-4-methoxyphenyl)sulfonamido)-2-hydroxybenzyl)-N-((4-methoxyphenyl) sulfonyl)glycine (10)

Obtained using method A from **25**; Pale yellow solid; Yield 69% (39 mg); ^1^H NMR (400 MHz, CD_3_OD) *δ* 7.67 (d, *J* = 8.8 Hz, 2H), 7.58 (d, *J* = 9.2 Hz, 2H), 7.07 (dd, *J* = 8.8, 2.4 Hz, 1H), 7.03 (d, *J* = 9.2 Hz, 2H), 6.97 (d, *J* = 8.8 Hz, 2H), 6.73 (d, *J* = 2.4 Hz, 1H), 6.65 (d, *J* = 8.8 Hz, 1H), 4.36 (s, 2H), 4.27 (s, 2H), 3.87 (s, 3H), 3.86 (s, 3H), 3.84 (s, 2H); ^13^C NMR (100 MHz, CD_3_OD) *δ* 172.5, 172.3, 164.8, 164.5, 157.0, 133.0, 132.7, 132.0, 131.6, 131.3, 131.0, 130.5, 123.8, 116.3, 115.3, 115.2, 56.2, 56.2, 53.8; LC/ESI-MS: *m/z* 595.1 [M + H]^+^; HRMS (ESI): *m/z* [M + H]^+^ calculated for C_25_H_27_N_2_O_11_S_2_: 595.1051; found: 595.1063; UPLC (retention time 3.56 min): purity 98.2%.

#### N-(3-((N-(Carboxymethyl)-4-methoxyphenyl)sulfonamido)benzyl)-N-((4-methoxyphenyl)sulfonyl)-glycine (11a)

Obtained using method A from **38a**; White solid; Yield 64% (80 mg); ^1^H NMR (400 MHz, DMSO-*d_6_*) *δ* 12.80 (brs, 2H, CO_2_H), 7.73 (d, *J* = 8.8 Hz, 2H), 7.54 (d, *J* = 8.8 Hz, 2H), 7.27 (t, *J* = 7.6 Hz, 1H), 7.14–7.13 (m, 2H), 7.08–7.05 (m, 4H), 6.90 (s, 2H), 4.33 (s, 2H), 4.27 (s, 2H), 3.84 (s, 3H), 3.83 (s, 3H), 3.69 (s, 2H); ^13^C NMR (100 MHz, DMSO-*d_6_*) *δ* 169.8, 169.7, 162.7, 162.5, 140.1, 136.9, 131.2, 129.8, 129.5, 129.2, 129.0, 127.4, 126.8, 114.3, 114.3, 55.6, 54.9, 51.9, 50.5, 47.2; LC/ESI-MS: *m/z* 577.1 [M - H]^-^; HRMS (ESI): *m/z* [M - H]^-^ calculated for C_25_H_25_N_2_O_10_S_2_: 577.0956; found: 577.0960; UPLC (retention time 3.81 min): purity 99.5%.

#### 2,2’-((4-Methyl-1,3-phenylene)bis(((4-methoxyphenyl)sulfonyl)azanediyl))diacetic acid (11b)

Obtained using method A from **38b**; White solid; Yield 46% 41 mg); ^1^H NMR (400 MHz, CDCl_3_) *δ* 10.10 (brs, 2H, CO_2_H), 7.54 (d, *J* = 8.4 Hz, 4H), 7.11 (d, *J* = 8.0 Hz, 1H), 7.05 (s, 1H), 6.971–6.93 (m, 5H), 4.44–4.33 (m, 2H), 4.21–4.17 (m, 2H), 4.07–4.03 (m, 2H), 3.85 (s, 3H), 3.83 (s, 3H), 2.21 (s, 3H); ^13^C NMR (100 MHz, CDCl_3_) *δ* 174.7, 163.5, 163.5, 139.6, 138.5, 137.5, 131.8, 130.5, 130.3, 130.0, 129.9, 129.8, 128.3, 114.5, 114.4, 55.8, 51.7, 51.6, 17.9; LC/ESI-MS: *m/z* 577.1 [M - H]^-^; HRMS (ESI): *m/z* [M - H]^-^ calculated for C_25_H_25_N_2_O_10_S_2_: 577.0956; found: 577.0960; UPLC (retention time 3.84 min): purity 99.7%.

#### N-(5-((N-(Carboxymethyl)-4-methoxyphenyl)sulfonamido)-2-methylphenethyl)-N-((4-methoxy-phenyl)sulfonyl)glycine (12a)

Obtained using method A from **43a**; White solid; Yield 42% (12 mg); ^1^H NMR (400 MHz, CDCl_3_) *δ* 9.20 (brs, 2H, CO_2_H), 7.73 (d, *J* = 8.8 Hz, 2H), 7.55 (d, *J* = 8.8 Hz, 2H), 7.01–6.99 (m, 2H), 6.93 (d, *J* = 8.8 Hz, 2H), 6.89 (d, *J* = 8.8 Hz, 2H), 6.76 (dd, *J* = 8.4, 2.0 Hz, 1H), 4.34 (s, 2H), 3.94 (s, 2H), 3.83 (s, 6H), 3.30 (t, *J* = 7.6 Hz, 2H), 2.79 (t, *J* = 7.6 Hz, 2H), 2.23 (s, 3H); ^13^C NMR (100 MHz, CDCl_3_) *δ* 174.4, 163.3, 163.3, 137.8, 137.6, 137.0, 131.3, 131.0, 130.4, 130.2, 130.0, 129.6, 126.3, 114.4, 114.2, 55.8, 52.6, 49.0, 32.6, 29.8, 19.1; LC/ESI-MS: *m/z* 605.1 [M - H]^-^; HRMS (ESI): *m/z* [M - H]^-^ calculated for C_27_H_29_N_2_O_10_S_2_: 605.1269; found: 605.1274; UPLC (retention time 3.99 min): purity 97.9%.

#### N-(5-((N-(Carboxymethyl)-4-methylphenyl)sulfonamido)-2-methylphenethyl)-N-((4-methylphenyl)-sulfonyl)glycine (12b)

Obtained using method A from **43b**; Pale yellow solid; Yield 61% (10 mg); ^1^H NMR (400 MHz, CD_3_OD) *δ* 9.20 (brs, 2H, CO_2_H), 7.69 (d, *J* = 8.4 Hz, 2H), 7.50 (d, *J* = 8.4 Hz, 2H), 7.36 (d, *J* = 8.4 Hz, 2H), 7.30 (d, *J* = 8.4 Hz, 2H), 7.05 (d, *J* = 8.4 Hz, 1H), 6.96 (dd, *J* = 8.4, 2.0 Hz, 1H), 6.81 (s, 1H), 4.34 (s, 2H), 3.97 (s, 2H), 3.28–3.26 (m, 2H), 2.74 (t, *J* = 8.0 Hz, 2H), 2.43 (s, 3H), 2.39 (s, 3H), 2.22 (s, 3H); ^13^C NMR (100 MHz, DMSO-*d_6_*) *δ* 170.4, 169.8, 143.5, 143.1, 137.5, 137.1, 136.8, 135.6, 135.6, 130.5, 129.7, 129.5, 128.6, 127.3, 126.9, 126.2, 52.2, 48.4, 48.3, 31.6, 21.0, 18.2; LC/ESI-MS: *m/z* 573.1 [M - H]^-^; HRMS (ESI): *m/z* [M - H]^-^ calculated for C_27_H_29_N_2_O_8_S_2_: 573.1371; found: 573.1376; UPLC (retention time 4.24 min): purity 95.2%.

#### N-(3–(2-((4-bromo-N-(carboxymethyl)phenyl)sulfonamido)ethyl)-4-methylphenyl)-N-((4-bromophenyl)-sulfonyl)glycine (12c)

Obtained using method A from **43c**; White solid; Yield 54% (172 mg); ^1^H NMR (400 MHz, DMSO-*d_6_*) *δ* 12.75 (brs, 2H, CO_2_H), 7.76–7.70 (m, 6H), 7.55 (d, *J* = 8.8 Hz, 2H), 7.07 (d, *J* = 8.0 Hz, 1H), 6.97 (d, *J* = 1.6 Hz, 1H), 6.91 (d, *J* = 8.0 Hz, 1H), 4.35 (s, 2H), 4.05 (s, 2H), 3.23 (t, *J* = 8.0 Hz, 2H), 2.76 (t, *J* = 8.0 Hz, 2H), 2.19 (s, 3H); ^13^C NMR (100 MHz, DMSO-*d_6_*) *δ* 170.1, 169.7, 138.8, 137.8, 137.2, 137.2, 136.0, 132.2, 132.2, 130.6, 129.2, 129.0, 128.9, 127.0, 126.7, 126.2, 52.3, 48.4, 48.3, 32.7, 18.2; LC/ESI-MS: *m/z* 703.0 [M(2^79^Br) + H]^+^, 705.0 [M(^79^Br, ^81^Br) + H]^+^, 707.0 [M(2^81^Br) + H]^+^; UPLC (retention time 4.57 min): purity 99.4%.

#### N-(5-((N-(Carboxymethyl)-4-methylphenyl)sulfonamido)-2-fluorophenethyl)-N-tosylglycine (12d)

Obtained using method A from **43d**; White solid; Yield 64% (50 mg); ^1^H NMR (400 MHz, CD_3_OD) *δ* 7.64 (d, *J* = 8.4 Hz, 2H), 7.52 (d, *J* = 8.4 Hz, 2H), 7.33 (d, *J* = 7.2 Hz, 4H), 7.12–7.08 (m, 1H), 6.98–6.96 (m, 1H), 6.94–6.90 (m, 1H), 4.37 (s, 2H), 3.97 (s, 2H), 3.40 (t, *J* = 7.2 Hz, 2H), 2.75 (t, *J* = 7.2 Hz, 2H), 2.41 (s, 3H), 2.40 (s, 3H); ^13^C NMR (100 MHz, CD_3_OD) *δ* 172.3, 172.1, 161.7 (*J*_C,F_ = 245 Hz), 145.6, 145.1, 138.3, 137.2 (*J*_C,F_ = 4 Hz), 137.0, 132.9 (*J*_C,F_ = 6 Hz), 130.8, 130.7, 130.6, 128.9, 128.4, 127.4 (*J*_C,F_ = 17 Hz), 116.8 (*J*_C,F_ = 23 Hz), 53.6, 49.3, 28.9, 21.5, 21.5; LC/ESI-MS: *m/z* 577.1 [M - H]^-^; HRMS (ESI) *m/z* [M - H]^-^ calculated for C_26_H_26_FN_2_O_8_S_2_: 577.1120; found: 577.1126; UPLC (retention time 4.18 min): purity 94.9%.

#### N-(4-hydroxy-3–(2-((4-methoxy-N-(2-methoxy-2-oxoethyl)phenyl)sulfonamido) ethyl)phenyl)-N-((4-methoxyphenyl)sulfonyl)glycine (13a)

Obtained using method A from **44a**; White solid; Yield 90% (79 mg); ^1^H NMR (400 MHz, DMSO-*d_6_*) *δ* 12.73 (brs, 2H, CO_2_H), 9.64 (brs, 1H, OH), 7.69 (d, *J* = 8.8 Hz, 2H), 7.53 (d, *J* = 8.8 Hz, 2H), 7.06 (d, *J* = 9.2 Hz, 2H), 7.05 (d, *J* = 8.8 Hz, 2H), 6.80 (dd, *J* = 8.8, 2.8 Hz, 1H), 6.72 (d, *J* = 2.8 Hz, 1H), 6.63 (d, *J* = 8.8 Hz, 1H), 4.22 (s, 2H), 3.84 (s, 2H), 3.82 (s, 6H), 3.25 (t, *J* = 7.6 Hz, 2H), 2.57–2.50 (m, 2H); ^13^C NMR (100 MHz, DMSO-*d_6_*) *δ* 170.3, 170.0, 162.5, 162.3, 154.8, 131.4, 130.7, 130.4, 130.2, 129.5, 129.0, 128.1, 124.7, 114.9, 114.3, 114.1, 55.6, 52.5, 47.9, 47.4, 30.9, 28.7; LC/ESI-MS: *m/z* 607.1 [M - H]^-^; HRMS (ESI): *m/z* [M - H]^-^ calculated for C_26_H_27_N_2_O_11_S_2_: 607.1062; found: 607.1068; UPLC (retention time 3.60 min): purity 99.4%.

#### N-(4-hydroxy-3–(2-((4-methyl-N-(2-methoxy-2-oxoethyl)phenyl)sulfonamido)ethyl)phenyl)-N-((4-methylphenyl)sulfonyl)glycine (13b)

Obtained using method A from **44b**; Pale yellow solid; Yield 75% (83 mg); ^1^H NMR (400 MHz, DMSO-*d_6_*) *δ* 12.75 (brs, 2H, CO_2_H), 9.67 (brs, 1H, OH), 7.64 (d, *J* = 8.4 Hz, 2H), 7.48 (d, *J* = 8.4 Hz, 2H), 7.36 (d, *J* = 8.0 Hz, 2H), 7.34 (d, *J* = 8.0 Hz, 2H), 6.81 (dd, *J* = 8.8, 2.4 Hz, 1H), 6.66 (d, *J* = 2.4 Hz, 1H), 6.34 (d, *J* = 8.8 Hz, 1H), 4.23 (s, 2H), 3.92 (s, 2H), 3.24 (t, *J* = 7.6 Hz, 2H), 2.56–2.50 (m, 2H), 2.38 (s, 3H), 2.36 (s, 3H); ^13^C NMR (100 MHz, DMSO-*d_6_*) *δ* 170.3, 169.9, 154.9, 143.3, 143.0, 137.0, 135.7, 130.5, 130.3, 129.6, 129.4, 128.2, 127.3, 126.8, 124.7, 114.9, 52.5, 47.9, 47.5, 28.6, 20.9; LC/ESI-MS: *m/z* 575.1 [M - H]^-^; HRMS (ESI): *m/z* [M - H]^-^ calculated for C_26_H_27_N_2_O_9_S_2_: 575.1163; found: 575.1169; UPLC (retention time 3.82 min): purity 98.9%.

#### N-([1,1’-Biphenyl]-4-ylsulfonyl)-N-(4-hydroxy-3–(2-(N-(2-methoxy-2-oxoethyl)-[1,1’-biphenyl]-4-sulfonamido)ethyl)phenyl)glycine (13c)

Obtained using method A from **44c**; White solid; Yield 50% (10 mg); ^1^H NMR (400 MHz, CD_3_OD) *δ* 7.78–7.75 (m, 6H), 7.69–7.67 (m, 5H), 7.47–7.44 (m, 5H), 7.42–7.38 (m, 2H), 6.93 (d, *J* = 8.4 Hz, 1H), 6.83 (s, 1H), 6.56 (d, *J* = 8.4 Hz, 1H), 4.37 (s, 2H), 3.98 (s, 2H), 3.50 (t, *J* = 7.6 Hz, 2H), 2.68 (t, *J* = 6.8 Hz, 2H); ^13^C NMR (100 MHz, CD_3_OD) *δ* 172.6, 172.6, 156.7, 147.1, 146.6, 140.6, 140.5, 140.1, 139.0, 132.4, 130.1, 130.1, 129.5, 129.5, 128.9, 128.5, 128.4, 128.4, 128.3, 126.8, 116.2, 54.1, 30.4; LC/ESI-MS: *m/z* 699.2 [M - H]^-^; HRMS (ESI): *m/z* [M - H]^-^ calculated for C_36_H_31_N_2_O_9_S_2_: 699.1476; found: 699.1487; UPLC (retention time 4.53 min): purity 97.5%.

#### N-(4-(carboxymethoxy)-3–(2-((N-(carboxymethyl)-4-methylphenyl)sulfonamido)ethyl)phenyl)-N-tosyl-glycine (14)

Obtained using method B from **47**; White solid; Yield 84% (62 mg); ^1^H NMR (400 MHz, CD_3_OD) *δ* 7.65 (d, *J* = 8.4 Hz, 2H), 7.52 (d, *J* = 8.4 Hz, 2H), 7.33 (d, *J* = 8.4 Hz, 2H), 7.32 (d, *J* = 8.4 Hz, 2H), 7.04 (dd, *J* = 8.8, 2.4 Hz, 1H), 6.80 (d, *J* = 2.4 Hz, 1H), 6.69 (d, *J* = 8.8 Hz, 1H), 4.58 (s, 2H), 4.33 (s, 2H), 3.97 (s, 2H), 3.46 (t, *J* = 7.2 Hz, 2H), 2.71 (t, *J* = 7.2 Hz, 2H), 2.42 (s, 6H); ^13^C NMR (100 MHz, CD_3_OD) *δ* 172.29, 172.27, 172.25, 157.0, 145.4, 144.9, 138.6, 137.2, 134.2, 132.4, 130.7, 130.6, 130.0, 129.0, 128.9, 128.5, 112.6, 66.0, 53.8, 53.7, 30.5, 21.5, 21.5; LC/ESI-MS: *m/z* 633.2 [M - H]^-^; HRMS (ESI): *m/z* [M - H]^-^ calculated for C_28_H_29_N_2_O_11_S_2_: 633.1218; found: 633.1227; UPLC (retention time 3.76 min): purity 95.1%.

#### N-(2–(5-((N-(Carboxymethyl)-4-methoxyphenyl)sulfonamido)-2-methylphenoxy)ethyl)-N-((4-meth-oxyphenyl)sulfonyl)glycine (15a)

Obtained using method A from **52a**; Pale yellow solid; Yield 76% (28 mg); ^1^H NMR (400 MHz, CDCl_3_) *δ* 8.37 (brs, 2H, CO_2_H), 7.75 (d, *J* = 8.8 Hz, 2H), 7.57 (d, *J* = 8.8 Hz, 2H), 6.95–6.89 (m, 5H), 6.30 (s, 1H), 6.53 (d, *J* = 7.6 Hz, 1H), 4.44 (s, 2H), 4.28 (s, 2H), 4.03–4.00 (m, 2H), 3.88 (s, 3H), 3.86 (s, 3H), 3.59–3.56 (m, 2H), 2.06 (s, 3H); ^13^C NMR (100 MHz, CDCl_3_) *δ* 174.3, 173.9, 163.3, 163.3, 156.3, 138.4, 130.9, 130.9, 130.2, 130.0, 129.5, 127.2, 120.7, 114.5, 114.1, 112.3, 67.2, 55.8, 52.7, 50.0, 48.1, 16.0; LC/ESI-MS: *m/z* 621.1 [M - H]^-^; HRMS (ESI): *m/z* [M - H]^-^ calculated for C_27_H_29_N_2_O_11_S_2_: 621.1218; found: 621.1223; UPLC (retention time 4.02 min): purity 98.4%.

#### N-(2–(5-((N-(Carboxymethyl)-4-methylphenyl)sulfonamido)-2-fluorophenoxy)ethyl)-N-tosylglycine (15b)

Obtained using method A from **52b**; White solid; Yield 72% (69 mg); ^1^H NMR (400 MHz, CD_3_OD) *δ* 7.73 (d, *J* = 8.4 Hz, 2H), 7.54 (d, *J* = 8.4 Hz, 2H), 7.36 (d, *J* = 8.4 Hz, 2H), 7.32 (d, *J* = 8.4 Hz, 2H), 7.02–6.97 (m, 1H), 6.80–6.75 (m, 2H), 4.38 (s, 2H), 4.17 (s, 2H), 4.02 (t, *J* = 5.4 Hz, 2H), 3.63 (t, *J* = 5.4 Hz, 2H), 2.43 (s, 3H), 2.38 (s, 3H); ^13^C NMR (100 MHz, CD_3_OD) *δ* 172.4, 172.1, 153.2 (*J*_C,F_ = 246 Hz), 147.6 (*J*_C,F_ = 10 Hz), 145.7, 145.1, 138.3, 137.4 (*J*_C,F_ = 3 Hz), 137.0, 130.8, 130.7, 129.0, 128.3, 123.3 (*J*_C,F_ = 7 Hz), 117.0 (*J*_C,F_ = 3 Hz), 116.9 (*J*_C,F_ = 20 Hz), 69.6, 53.6, 50.9, 21.5, 21.5; LC/ESI-MS: *m/z* 595.2 [M + H]^+^; HRMS (ESI) *m/z* [M + H]^+^ calculated for C_26_H_28_FN_2_O_9_S_2_: 595.1215; found: 595.1218; UPLC (retention time 4.91 min): purity 99.3%.

#### N-(4-hydroxy-3–(2-((N-(2-methoxy-2-oxoethyl)-4-methylphenyl)sulfonamido)ethoxy)phenyl)-N-tosyl-glycine (16)

Obtained using method A from **53**; White solid; Yield 71% (39 mg); ^1^H NMR (400 MHz, CD_3_OD) *δ* 7.73 (d, *J* = 8.4 Hz, 2H), 7.53 (d, *J* = 8.4 Hz, 2H), 7.33 (d, *J* = 7.6 Hz, 4H), 6.69–6.60 (m, 3H), 5.49 (bss, 1H, OH), 4.33 (s, 2H), 4.19 (s, 2H), 3.98 (t, *J* = 5.4 Hz, 2H), 3.60 (t, *J* = 5.4 Hz, 2H), 2.42 (s, 3H), 2.40 (s, 3H); ^13^C NMR (100 MHz, CD_3_OD) *δ* 172.6, 172.3, 148.3, 147.3, 145.4, 145.1, 138.2, 137.2, 132.6, 130.8, 130.6, 129.0, 128.4, 123.9, 116.2, 115.7, 68.9, 53.9, 50.6, 21.5, 21.5; LC/ESI-MS: *m/z* 591.2 [M - H]^-^; HRMS (ESI): *m/z* [M - H]^-^ calculated for C_26_H_27_N_2_O_10_S_2_: 591.1113; found: 591.1121; UPLC (retention time 3.93 min): purity 97.4%.

#### N-(3–(5-((N-(Carboxymethyl)-4-methylphenyl)sulfonamido)-2-hydroxyphenyl)propyl)-N-tosyl-glycine (17)

Obtained using method A from **58**; White solid; Yield 56% (35 mg); ^1^H NMR (400 MHz, CD_3_OD) *δ* 7.68 (d, *J* = 8.4 Hz, 2H), 7.50 (d, *J* = 8.4 Hz, 2H), 7.35–7.31 (m, 4H), 6.87 (dd, *J* = 8.4, 2.4 Hz, 1H), 6.73 (d, *J* = 2.4 Hz, 1H), 6.64 (d, *J* = 8.8 Hz, 1H), 4.31 (s, 2H), 3.99 (s, 2H), 3.19 (d, *J* = 7.6 Hz, 2H), 2.42 (s, 3H), 2.41 (s, 3H), 2.39–2.37 (m, 2H), 1.72–1.65 (m, 2H); ^13^C NMR (100 MHz, CD_3_OD) *δ* 172.5, 172.4, 156.4, 145.2, 144.9, 138.2, 137.2, 132.3, 131.6, 130.7, 130.5, 129.5, 129.5, 129.0, 128.4, 116.0, 54.0, 28.6, 28.0, 21.5, 21.5; LC/ESI-MS *m/z* 589.1 [M - H]^-^; HRMS (ESI): *m/z* [M - H]^-^ calculated for C_27_H_29_N_2_O_9_S_2_: 589.1320; found: 589.1326; UPLC (retention time 3.89 min): purity 97.6%.

### Fluorescence polarization assay

The FP assay was performed as previously described, with minor modifications^53^. Briefly, the assay was conducted in 384-well black non-binding surface plates (Corning, Cat. No. 3575) using a Wallac Victor 3 V microplate reader (PerkinElmer, Shelton, CT) equipped with excitation and emission filters at 485 and 535 nm, respectively. A fluorescein-labeled Nrf2 peptide (FITC-LDEETGEFL-NH_2_) and recombinant Keap1 Kelch-domain protein were used in 10 mM HEPES buffer (pH 7.4) containing 3.4 mM EDTA, 150 mM NaCl, and 0.005% Tween-20. In each well, 10 µL of the test compound solution was incubated with 10 µL of 400 nM Keap1 protein for 30 min at room temperature, followed by the addition of 20 µL of 20 nM fluorescent peptide. Fluorescence polarisation was measured after 1 h of incubation at room temperature. The IC_50_ values were calculated by fitting the inhibition data to a four-parameter logistic model using SigmaPlot 12.3 software. The *K_i_* values for the FP assay were determined using the same equation described in the TR-FRET assay section, with the following parameters: *R_0_* = 100 nM, *L_0_* = 10 nM, and *K_d_* = 25.6 nM.

### Time-resolved fluorescence resonance energy transfer assay

The TR-FRET assay was performed as previously described, with minor modifications^54^. Briefly, the assay was conducted in 384-well white non-binding surface plates (Corning, Cat. No. 3574) using a Wallac Victor 3 V microplate reader (PerkinElmer, Shelton, CT) equipped with excitation and emission filters at 340, 495, and 520 nm for terbium and fluorescein detection, respectively. The assay buffer consisted of 10 mM HEPES (pH 7.4), 0.5 mM EDTA, 150 mM NaCl, and 0.005% Tween-20. A terbium-labeled anti-His antibody (LanthaScreen^™^ Elite Tb-anti-His Antibody, Thermo Fisher Scientific, 3.4 µM stock) and a FITC-labeled Nrf2 peptide (FITC-LDEETGEFL-NH_2_) were used as donor and acceptor, respectively. A mixture of 20 nM His-tagged Keap1 Kelch-domain protein and 2 nM terbium-conjugated anti-His antibody (diluted ∼1:1700 in assay buffer) was pre-incubated in assay buffer (1:1, v/v) for 30 min at room temperature. Then, 10 µL of this mixture was dispensed into each well (final concentrations: protein 5 nM, Tb-antibody 0.5 nM), followed by the addition of 0.2 µL of test compound solution (serially diluted in DMSO, final concentrations ranging from 1 µM to 0.01 nM). After incubation for 30 min, 9.8 µL of FITC-labeled Nrf2 peptide (final, 25 nM) was added and further incubated for 1 h at room temperature. TR-FRET signals were recorded at 495 nm and 520 nm, and the emission ratio (520/495 × 10,000) was calculated. IC_50_ values were determined by fitting the normalised data to a four-parameter logistic model using SigmaPlot 12.3 software. The IC_50_ values were converted to the inhibition constants (*K_i_*) using the previously reported equations^54^.

$${\text{IC}}_{50}=((1-0.5f_{0})R_{0}-\frac{f_{0}\times {\;K}_{d}\times R_{0}}{2L_{0}-{\;f}_{0}\times R_{0}})(\frac{K_{i}(2L_{0}-{\;f}_{0}\times R_{0})}{K_{d}\times {\;f}_{0}\times R_{0}}+1)$$

where *R_0_* is the total concentration of the Keap1 protein (5 nM), *L_0_* is the total concentration of the FITC-labeled probe (25 nM), *K_d_* is the dissociation constant of the probe-protein complex (22.6 nM), and *f_0_* is the fraction of Keap1 protein bound to the probe (calculated as 0.469).

### Metabolic stability

Metabolic stability was evaluated using 0.5 mg/mL pooled human liver microsomal protein (Corning, #452117) in 0.1 M K_2_HPO_4_/KH_2_PO_4_ buffer (pH 7.4). Test compounds (1 µM) were preincubated at 37 °C for 5 min before initiating the reaction with an NADPH regeneration system (Promega, V9510). After incubation for 30 min at 37 °C, the metabolic reaction was quenched by adding ice-cold acetonitrile containing an internal standard (chlorpropamide, 1 µM). The mixture was centrifuged at 15,000 rpm (5 min and 4 °C), and the clear supernatant was analysed using LC-MS/MS (Agilent 1290 Infinity LC system coupled to a Triple Quad 5500 MS, Applied Biosystems). Chromatographic separation was performed on a Kinetex C18 column (2.1 × 100 mm, 1.7 µm; Phenomenex) with a gradient elution using 0.1% HCOOH in water (A) and ACN (B). Data collection was performed in MRM mode with the Xcalibur software. Metabolic stability was expressed as the percentage of parent compound that persisted after a 30-min incubation. Verapamil (1 µM) was used as a positive control to validate assay performance.

### Molecular docking studies

Molecular docking simulations were performed as previously described, with minor modifications^40^. The co-crystal structure of Keap1 bound to compound **3a** (PDB ID: 5FNT) was retrieved from the RCSB Protein Data Bank. Protein preparation was carried out using AutoDock Tools, which involved removing water molecules, adding hydrogen atoms, and assigning Kollman charges^55^^,^^56^. Ligand structures were generated in MOE and converted into docking-ready formats for AutoDock 4.0. The docking grid box was centred on the co-crystallized ligand site and defined as a 40 × 40 × 40-point grid in the X, Y, and Z dimensions, with a grid spacing of 0.375 Å. Docking runs employed the Lamarckian Genetic Algorithm (LGA) with 100 independent trials, while other parameters were kept at default settings. The lowest-energy conformations were considered the best docking poses. The reliability of the protocol was validated by re-docking compound **3a**, which reproduced the experimental binding mode with an RMSD of 0.60 Å, confirming the robustness of the docking procedure.

## Supplementary Material

Keap1Nrf2_MS3 SP_v9_122822025_anonymous.docx

## Data Availability

The data supporting this article are included as part of the article or the supplemental material.
