# Supplementary material for: Design and optimisation of meta-substituted bis(arylsulfonamido)benzene inhibitors through a molecular hybridisation strategy targeting the Keap1-Nrf2 protein-protein interaction
Source: J Enzyme Inhib Med Chem. 2026 Feb 6;41(1):2622777. doi: 10.1080/14756366.2026.2622777 (PMC12885012; doi:10.1080/14756366.2026.2622777)

**Supporting information**

**Design and optimization of meta-substituted bis(arylsulfonamido)benzene inhibitors through a molecular hybridization strategy targeting the Keap1-Nrf2 protein-protein interaction**

**Table of Contents:**

1. General chemistry S2

2. Synthetic procedure and characterization of intermediates S3

3. Table S1 original data of activity in FP assay for final compounds S41

4. ^1^H and ^13^C NMR spectra of final compounds S44

5. UPLC analysis and purity spectra of final compounds S67

**1. General chemistry**

All solvents and reagents were obtained from commercial suppliers and used as received without further purification unless otherwise stated. Moisture-sensitive reactions were carried out in dry solvents under a nitrogen atmosphere. Analytical thin-layer chromatography (TLC) was performed on aluminum-backed Silica G TLC plates coated with DC Kieselgel 60 F254 (Merk or Sigma-Aldrich). TLC plates were visualized under ultraviolet light (254 nm) and/or by staining with potassium permanganate followed by heating. Flash column chromatography was conducted using a Teledyne ISCO Combiflash Companion system equipped with prepacked Teledyne ISCO RediSep normal-phase silica cartridges (230-400 mesh), eluting with a gradient of 0-100% ethyl acetate/ hexane or 0-20% methanol/dichloromethane. ^1^H and ^13^C NMR spectra were recorded at 400 MHz and 100 MHz, respectively, at ambient temperature using a Bruker Avance III 400 Multinuclear NMR spectrometer**.** Chemical shifts (*δ*) are reported in parts per million (ppm) relative to the residual solvent signal as an internal reference (CDCl_3_, CD_3_OD, or DMSO-*d_6_*). In the NMR tabulation, spin multiplicities are designated as s (singlet), d (doublet), dd (doublet of doublets), t (triplet), q (quartet), m (multiplet), and brs (broad singlet). Coupling constants (*J*) are given in hertz (Hz). Analytical liquid chromatography/mass spectrometry (LC/MS) was performed on an Agilent 1200 Infinity high-performance LC (HPLC) system equipped with an Agilent 6410 quadrupole mass spectrometer operating with an electrospray ionization (ESI) source, using an Inertsil ODS-3 C18 column (3 mm × 33 mm, 3 μM) at a temperature of 40 °C. The mobile phases A and B consisted of water with 0.1% formic acid and methanol with 0.1% formic acid, respectively. The proportion of mobile phase B was linearly increased from 5% to 90% over 5 minutes at a flow rate of 0.8 mL/min, with UV detection at 280 nm. High-resolution mass spectra (HRMS) analyses were conducted at the Center for Integrative Proteomics Research (CIPR) at xxx University. Solutions at a 1 μg/mL concentration were directly infused using a Thermo LTQ Orbitrap Velos ETD system coupled with a Dionex UltiMate 3000 nano-flow 2D LC. The purities of the final compounds, except for compound **9**, were determined to be ≥95% (calculated by rounding to the nearest integer). Purity analysis was performed using a Waters ACQUITY UPLC^TM^ system. Metabolic stability studies were performed at the Daegu-Gyeongbuk Medical Innovation Foundation (K-MEDI hub) in Korea. We thank the Center for Integrative Proteomics Research (CIPR), xxx University, for performing the HRMS experiments.

**2. Synthetic procedure and characterization of compounds**

*Methyl 2-hydroxy-5-nitrobenzoate (****18****).* To a solution of 2-hydroxy-5-nitrobenzoic acid (2.0 g, 10.92 mmol) in methanol (25 mL) was added thionyl chloride (13.0 g, 109.22 mmol) at 0 °C. The reaction mixture was stirred at 70 °C overnight. After the reaction was completed, the crude mixture was cooled to room temperature, added to sat. NaHCO_3_ and then extracted with dichloromethane. The organic layer was dried over anhydrous Na_2_SO_4_ and concentrated under reduced pressure. The residue was purified by flash column chromatograph (0-60% ethyl acetate/hexane) to give the product as a yellow solid (1.7 g, 77%);^1^H NMR (400 MHz, DMSO-*d_6_*) *δ* 8.54 (s, 1H), 7.91 (d, *J* = 9.2 Hz, 1H), 6.42 (d, *J* = 9.2 Hz, 1H), 3.75 (s, 3H); ^13^C NMR (100 MHz, DMSO-*d_6_*) *δ* 175.0, 166.8, 130.7, 130.0, 128.2, 122.9, 115.0, 51.2; LC/MS (ESI) *m/z* 196.1 [M - H]^-^.

*Methyl 2-(benzyloxy)-5-nitrobenzoate (****19****).* To a solution of **18** (500 mg, 2.54 mmol) in *N,N*-dimethylformamide (10 mL) were added potassium carbonate (701 mg, 5.07 mmol) and benzyl bromide (521 mg, 3.04 mmol) at room temperature. The reaction mixture was stirred at 80 ^o^C for 8 hours. After the reaction was completed, the crude mixture was cooled to room temperature, diluted with water, and extracted with ethyl acetate. The organic layer was dried over anhydrous Na_2_SO_4_ and concentrated under reduced pressure. The residue was purified by flash column chromatograph (0-30% ethyl acetate/hexane) to give the product as a yellow solid (523 mg, 72%); ^1^H NMR (400 MHz, CDCl_3_) *δ* 8.73 (d, *J* = 2.8 Hz, 1H), 8.31 (dd, *J* = 9.2, 2.8 Hz, 1H), 7.48 (d, *J* = 7.2 Hz, 2H), 7.43-7.32 (m, 3H), 7.10 (d, *J* = 9.2 Hz, 1H), 5.30 (s, 2H), 3.94 (s, 3H); ^13^C NMR (100 MHz, CDCl_3_) *δ* 164.6, 162.7, 140.9, 135.3, 128.9, 128.9, 128.5, 128.0, 126.9, 121.1, 113.6, 71.3, 52.7.

*(2-Chloro-5-nitrophenyl)methanol (****20a****).* To a solution of 2-chloro-5-nitrobenzoic acid (1.5 g, 7.44 mmol) in tetrahydrofuran (15 mL) was added borane tetrahydrofuran complex solution (1.0 M in THF, 15 mL, 14.88 mmol) at room temperature. The reaction mixture was stirred at 60 °C for 2 hours. After the reaction was completed, the crude mixture was added to methanol and water and then concentrated under reduced pressure. The aqueous solution was acidified with 6 N HCl until the pH reached around one and extracted with ethyl acetate. The organic layer was dried over anhydrous Na_2_SO_4_ and concentrated under reduced pressure to give the product as a white solid (1.4 g, 98%); ^1^H NMR (400 MHz, DMSO-*d_6_*) *δ* 8.33 (d, *J* = 2.8 Hz, 1H), 8.17 (dd, *J* = 8.8, 2.8 Hz, 1H), 7.70 (d, *J* = 8.8 Hz, 1H), 5.79 (t, *J* = 5.6 Hz, 1H, OH), 4.62 (d, *J* = 5.6 Hz, 2H); ^13^C NMR (100 MHz, DMSO-*d_6_*) *δ* 146.5. 141.9. 137.5. 130.2. 123.0. 122.1. 59.7; LC/MS (ESI) *m/z* 186.1 [M - H]^-^.

*(2-Bromo-5-nitrophenyl)methanol (****20b****).* Prepared as described in the experimental procedure of **20a** from 2-bromo-5-nitrobenzoic acid to obtain the title compound as a pale yellow solid (478 mg, quantitative); ^1^H NMR (400 MHz, CDCl_3_) *δ* 8.43 (d, *J* = 2.8 Hz, 1H), 8.02 (dd, *J* = 8.8, 2.8 Hz, 1H), 7.72 (d, *J* = 8.8 Hz, 1H), 4.83 (d, *J* = 5.6 Hz, 2H), 2.14 (t, *J* = 5.6 Hz, 1H, OH); ^13^C NMR (100 MHz, CDCl_3_) *δ* 147.7, 142.1, 133.5, 128.9, 123.5, 123.1, 64.2.

*(2-Methoxy-5-nitrophenyl)methanol (****20c****).* Prepared as described in the experimental procedure of **20a** from 2-methoxy-5-nitrobenzoic acid to obtain the title compound as a white solid (451 mg, 97%); ^1^H NMR (400 MHz, DMSO-*d_6_*) *δ* 8.23 (d, *J* = 2.0 Hz, 1H), 8.15 (dd, *J* = 8.8, 2.0 Hz, 1H), 7.15 (d, *J* = 8.8 Hz, 1H), 5.43 (t, *J* = 5.2 Hz, 1H, OH), 4.52 (d, *J* = 5.2 Hz, 2H), 3.91 (s, 3H); ^13^C NMR (100 MHz, DMSO-*d_6_*) *δ* 161.1, 140.7, 132.0, 124.1, 121.7, 110.5, 57.3, 56.3; LC/MS (ESI) *m/z* 184.1 [M + H]^+^.

*(2-Methyl-5-nitrophenyl)methanol (****20d****).* Prepared as described in the experimental procedure of **20a** from 2-methyl-5-nitrobenzoic acid to obtain the title compound as a white solid (871 mg, 94%); ^1^H NMR (400 MHz, CDCl_3_) *δ* 8.31 (d, *J* = 2.4 Hz, 1H), 8.06 (dd, *J* = 8.4, 2.4 Hz, 1H), 7.31 (d, *J* = 8.4 Hz, 1H), 4.78 (d, *J* = 5.6 Hz, 2H), 2.42 (s, 3H), 1.82 (t, *J* = 5.6 Hz, 1H, OH); ^13^C NMR (100 MHz, CDCl_3_) *δ* 143.6, 140.5, 131.1, 122.6, 122.0, 62.6, 19.0; LC/MS (ESI) *m/z* 166.1 [M - H]^-^.

*(2-Fluoro-5-nitrophenyl)methanol (****20e****).* Prepared as described in the experimental procedure of **20a** from 2-fluoro-5-nitrobenzoic acid to obtain the title compound as a pale yellow oil (404 mg, 87%); ^1^H NMR (400 MHz, CDCl_3_) *δ* 8.41-8.38 (m, 1H), 8.18-8.14 (m, 1H), 7.20-7.15 (m, 1H), 4.83 (s, 2H); ^13^C NMR (100 MHz, CDCl_3_) *δ* 163.7 (*J*_C,F_ = 256 Hz), 144.5, 130.0 (*J*_C,F_ = 17 Hz), 125.1 (*J*_C,F_ = 10 Hz), 124.8 (*J*_C,F_ = 6 Hz), 116.3 (*J*_C,F_ = 24 Hz), 58.4 (*J*_C,F_ = 4 Hz).

*(2-(Benzyloxy)-5-nitrophenyl)methanol (****20f****).* To a solution of **7** (498 mg, 1.73 mmol) in tetrahydrofuran (5 mL) was added diisobutylaluminum hydride (1.0 M in THF, 3.5 mL, 3.47 mmol) at room temperature. The reaction mixture was stirred at room temperature for 6 hours. After the reaction was completed, the crude mixture was added to 6 N HCl, filtered off through celite, and then extracted with dichloromethane. The organic layer was dried over anhydrous Na_2_SO_4_ and concentrated under reduced pressure. The residue was purified by flash column chromatograph (0-50% ethyl acetate/hexane) to give the product as a yellow solid (454 mg, quantitative); ^1^H NMR (400 MHz, DMSO-*d_6_*) *δ* 8.27 (d, *J* = 2.4 Hz, 1H), 8.15 (dd, *J* = 8.8, 2.4 Hz, 1H), 7.47 (d, *J* = 7.6 Hz, 2H), 7.43-7.39 (m, 2H), 7.36-7.34 (m, 1H), 7.25 (d, *J* = 8.8 Hz, 1H), 5.47 (brs, 1H, OH), 5.29 (s, 2H), 4.59 (s, 2H); ^13^C NMR (100 MHz, DMSO-*d_6_*) *δ* 160.0, 140.9, 136.2, 132.4, 128.6, 128.1, 127.4, 124.0, 121.8, 111.8, 70.0, 57.4; LC/MS (ESI) *m/z* 260.1 [M + H]^+^.

*2-Chloro-5-nitrobenzaldehyde (****21a****).* To a solution of **20a** (1.4 g, 7.20 mmol) in dichloromethane (36 mL) was added pyridinium chlorochromate (2.3 g, 10.80 mmol) at room temperature. The reaction mixture was stirred at 55 °C for 1 hour. After the reaction was completed, the crude mixture was filtered off through celite, diluted with water, and then extracted with dichloromethane. The organic layer was dried over anhydrous Na_2_SO_4_ and concentrated under reduced pressure. The residue was purified by flash column chromatograph (0-20% ethyl acetate/hexane) to give the product as a white solid (1.2 g, 93%); ^1^H NMR (400 MHz, CDCl_3_) *δ* 10.49 (s, 1H), 8.75 (d, *J* = 2.8 Hz, 1H), 8.38 (dd, *J* = 8.8, 2.8 Hz, 1H), 7.69 (d, *J* = 8.8 Hz, 1H); ^13^C NMR (100 MHz, CDCl_3_) *δ* 187.5, 147.2, 143.7, 133.3, 132.2, 129.0, 124.6.

*2-Bromo-5-nitrobenzaldehyde (****21b****).* Prepared as described in the experimental procedure of **21a** from intermediate **20b** to obtain the title compound as a white solid (300 mg, 56%); ^1^H NMR (400 MHz, CDCl_3_) *δ* 10.38 (s, 1H), 8.72 (d, *J* = 2.8 Hz, 1H), 8.29 (dd, *J* = 8.8, 2.8 Hz, 1H), 7.89 (d, *J* = 8.8 Hz, 1H); ^13^C NMR (100 MHz, CDCl_3_) *δ* 189.5, 135.4, 134.5, 133.1, 128.9, 124.8.

*2-Methoxy-5-nitrobenzaldehyde (****21c****).* Prepared as described in the experimental procedure of **21a** from intermediate **20c** to obtain the title compound as a white solid (314 mg, 76%); ^1^H NMR (400 MHz, CDCl_3_) *δ* 10.43 (s, 1H), 8.67 (d, *J* = 2.8 Hz, 1H), 8.42 (dd, *J* = 9.2, 2.8 Hz, 1H), 7.13 (d, *J* = 9.2 Hz, 1H), 4.06 (s, 3H); ^13^C NMR (100 MHz, CDCl_3_) *δ* 187.6, 165.7, 141.7, 130.8, 124.7, 112.4, 56.9.

*2-Methyl-5-nitrobenzaldehyde (****21d****).* Prepared as described in the experimental procedure of **21a** from intermediate **20d** to obtain the title compound as a pale yellow solid (154 mg, 58%); ^1^H NMR (400 MHz, CDCl_3_) *δ* 10.31 (s, 1H), 8.63 (d, *J* = 2.4 Hz, 1H), 8.30 (dd, *J* = 8.4, 2.4 Hz, 1H), 7.47 (d, *J* = 8.4 Hz, 1H), 2.78 (s, 3H); ^13^C NMR (100 MHz, CDCl_3_) *δ* 190.5, 147.7, 146.8, 134.7, 133.2, 127.6, 126.6, 19.9; LC/MS (ESI) *m/z* 166.1 [M + H]^+^.

*2-Fluoro-5-nitrobenzaldehyde (****21e****).* Prepared as described in the experimental procedure of **21a** from intermediate **20e** to obtain the title compound as a white solid (275 mg, 73%); ^1^H NMR (400 MHz, CDCl_3_) *δ* 10.36 (s, 1H), 8.74-8.72 (m, 1H), 8.51-8.47 (m, 1H), 7.43-7.39 (m, 1H); ^13^C NMR (100 MHz, CDCl_3_) *δ* 184.9 (*J*_C,F_ = 5 Hz), 167.3 (*J*_C,F_ = 267 Hz), 144.9, 131.1 (*J*_C,F_ = 11Hz), 124.9 (*J*_C,F_ = 4 Hz), 124.6 (*J*_C,F_ = 11 Hz), 118.3 (*J*_C,F_ = 23 Hz); LC/MS (ESI) *m/z* 168.9 [M]^-^.

*2-(Benzyloxy)-5-nitrobenzaldehyde (****21f****).* Prepared as described in the experimental procedure of **21a** from intermediate **20f** to obtain the title compound as a yellow solid (268 mg, 61%); ^1^H NMR (400 MHz, CDCl_3_) *δ* 10.51 (s, 1H), 8.71 (d, *J* = 2.8 Hz, 1H), 8.40 (dd, *J* = 9.2, 2.8 Hz, 1H), 7.45-7.39 (m, 5H), 7.19 (d, *J* = 9.2 Hz, 1H), 5.33 (s, 2H); ^13^C NMR (100 MHz, CDCl_3_) *δ* 187.6, 164.8, 141.9, 134.7, 130.7, 129.1, 129.0, 127.6, 125.1, 124.8, 113.6, 71.7.

*N-(2-Chloro-5-nitrobenzyl)-4-methoxybenzenesulfonamide (****22a****).* To a solution of **21a** (200 mg, 1.08 mmol) in dichloromethane (6 mL) were added sodium triacetoxyborohydride (457 mg, 2.16 mmol), 4-methoxybenzenesulfonamide (212 mg, 1.13 mmol), acetic acid (3 drops), and triethylamine (218 mg, 2.16 mmol) at room temperature. The reaction mixture was stirred at room temperature overnight. After the reaction was completed, the crude mixture was added to water and extracted with dichloromethane. The organic layer was dried over anhydrous Na_2_SO_4_ and concentrated under reduced pressure. The residue was purified by flash column chromatograph (0-30% ethyl acetate/hexane) to give the product as a pale yellow solid (272 mg, 71%); ^1^H NMR (400 MHz, DMSO-*d_6_*) *δ* 8.34 (brs, 1H, NH), 8.19 (s, 1H), 8.09 (d, *J* = 8.8 Hz, 1H), 7.71-7.67 (m, 3H), 7.05 (d, *J* = 8.4 Hz, 2H), 4.17 (s, 2H), 3.81 (s, 3H); ^13^C NMR (100 MHz, DMSO-*d_6_*) *δ* 162.2, 146.2, 138.9, 137.2, 132.0, 130.6, 128.6, 124.1, 123.6, 114.3, 55.6, 43.2; LC/MS (ESI) *m/z* 357.1 [M + H]^+^.

*N-(2-Bromo-5-nitrobenzyl)-4-methoxybenzenesulfonamide (****22b****).* Prepared as described in the experimental procedure of **22a** from intermediate **21b** to obtain the title compound as a white solid (206 mg, 47%); ^1^H NMR (400 MHz, CDCl_3_) *δ* 8.14 (d, *J* = 2.4 Hz, 1H), 7.86 (dd, *J* = 8.8, 2.4 Hz, 1H), 7.73 (d, *J* = 8.8 Hz, 2H), 7.59 (d, *J* = 8.8 Hz, 1H), 6.86 (d, *J* = 8.8 Hz, 2H), 6.53 (t, *J* = 6.4 Hz, 1H, NH), 4.22 (d, *J* = 6.4 Hz, 2H), 3.79 (s, 3H); ^13^C NMR (100 MHz, CDCl_3_) *δ* 162.8, 147.1, 138.5, 133.6, 131.5, 130.1, 129.1, 124.3, 123.4, 114.3, 55.6, 46.6; LC/MS (ESI) *m/z* 401.1 [M(^79^Br) + H]^+^, 403.0 [M(^81^Br) + H]^+^.

*N-(2-Methoxy-5-nitrobenzyl)-4-methoxybenzenesulfonamide (****22c****).* Prepared as described in the experimental procedure of **22a** from intermediate **21c** to obtain the title compound as a yellow solid (513 mg, 88%); ^1^H NMR (400 MHz, DMSO-*d_6_*) *δ* 8.11-8.08 (m, 1H), 7.78 (d, *J* = 8.4 Hz, 1H), 7.69 (d, *J* = 8.4 Hz, 2H), 7.22 (s, 1H), 7.04 (d, *J* = 8.4 Hz, 2H), 4.00 (d, *J* = 6.4 Hz, 2H), 3.86 (s, 3H), 3.80 (s, 3H); ^13^C NMR (100 MHz, DMSO-*d_6_*) *δ* 162.1, 140.3, 132.2, 128.6, 126.8, 124.8, 123.7, 114.2, 110.9, 56.5, 55.6, 40.4.

*N-(2-Methyl-5-nitrobenzyl)-4-methoxybenzenesulfonamide (****22d****).* Prepared as described in the experimental procedure of **22a** from intermediate **21d** to obtain the title compound as a white solid (57 mg, 18%); ^1^H NMR (400 MHz, DMSO-*d_6_*) *δ* 8.12 (t, *J* = 6.0 Hz, 1H, NH), 8.04 (d, *J* = 2.4 Hz, 1H), 7.99 (dd, *J* = 8.4, 2.4 Hz, 1H), 7.72 (d, *J* = 8.4 Hz, 2H), 7.40 (d, *J* = 8.4 Hz, 1H), 7.06 (d, *J* = 6.4 Hz, 2H), 4.06 (d, *J* = 6.0 Hz, 2H), 3.82 (s, 3H), 2.33 (s, 3H); ^13^C NMR (100 MHz, DMSO-*d_6_*) *δ* 162.2, 145.6, 144.5, 137.6, 132.2, 131.2, 128.6, 122.6, 122.0, 114.3, 55.6, 43.4, 18.7; LC/MS (ESI) *m/z* 337.1 [M + H]^+^.

*N-(2-Fluoro-5-nitrobenzyl)-4-methoxybenzenesulfonamide (****22e****).* Prepared as described in the experimental procedure of **22a** from intermediate **21e** to obtain the title compound as a pale yellow solid (245 mg, 49%); ^1^H NMR (400 MHz, DMSO-*d_6_*) *δ* 8.26 (t, *J* = 6.0 Hz, 1H, NH), 8.17-8.16 (m, 1H), 7.54-7.52 (m, 2H), 7.67 (d, *J* = 8.8 Hz, 2H), 7.42-7.38 (m, 1H), 7.02 (d, *J* = 8.8 Hz, 2H), 4.13 (d, *J* = 6.0 Hz, 1H), 3.80 (s, 3H); ^13^C NMR (100 MHz, DMSO-*d_6_*) *δ* 163.3 (*J*_C,F_ = 254 Hz), 162.2, 143.7, 132.1, 128.6, 126.7 (*J*_C,F_ = 17 Hz), 125.7 (*J*_C,F_ = 6 Hz), 125.2 (*J*_C,F_ = 11 Hz), 116.7 (*J*_C,F_ = 24 Hz), 114.3, 55.6; LC/MS (ESI) *m/z* 341.0 [M + H]^+^.

*N-(2-(Benzyloxy)-5-nitrobenzyl)-4-methoxybenzenesulfonamide (****22f****).* Prepared as described in the experimental procedure of **22a** from intermediate **21f** to obtain the title compound as a pale yellow solid (249 mg, 60%); ^1^H NMR (400 MHz, DMSO-*d_6_*) *δ* 8.15-8.09 (m, 3H), 7.68 (d, *J* = 8.8 Hz, 2H), 7.46-7.35 (m, 5H), 7.21 (d, *J* = 8.8 Hz, 1H), 7.00 (d, *J* = 8.8 Hz, 2H), 5.27 (s, 2H), 4.07 (d, *J* = 6.0 Hz, 2H), 3.79 (s, 3H); ^13^C NMR (100 MHz, DMSO-*d_6_*) *δ* 162.1, 160.5, 140.5, 136.0, 132.1, 128.6, 128.5, 128.1, 127.4, 127.3, 124.6, 123.9, 114.2, 112.2, 70.2, 55.6, 40.3; LC/MS (ESI) *m/z* 429.1 [M + H]^+^.

*N-(4-Chloro-3-(((4-methoxyphenyl)sulfonamido)methyl)phenyl)-4-methoxybenzenesulfonamide (****23a****).* To a solution of **22a** (100 mg, 0.28 mmol) in acetic acid (1.5 mL) and water (0.5 mL) was added iron powder (157 mg, 2.80 mmol) at room temperature. The reaction mixture was stirred at 60 °C for 30 minutes. After the reaction was completed, the crude mixture was cooled to room temperature, added to sat. NaHCO_3_ and then extracted with ethyl acetate. The organic layer was dried over anhydrous Na_2_SO_4,_ and concentrated under reduced pressure to give *N*-(5-amino-2-chlorobenzyl)-4-methoxybenzenesulfonamide as a pale brown oil (108 mg, quantitative); ^1^H NMR (400 MHz, CDCl_3_) *δ* 7.74 (d, *J* = 8.4 Hz, 2H), 6.97 (d, *J* = 8.4 Hz, 1H), 6.91 (d, *J* = 8.4 Hz, 2H), 6.61 (s, 1H), 6.45 (d, *J* = 8.4 Hz, 1H), 5.39 (brs, 1H, NH), 4.07 (d, *J* = 5.6 Hz, 2H), 3.83 (s, 3H), 2.85 (brs, 2H, NH); ^13^C NMR (100 MHz, CDCl_3_) *δ* 163.0, 145.7, 134.5, 131.6, 130.0, 129.3, 121.9, 116.6, 115.7, 114.3, 55.7, 45.1; LC/MS (ESI) *m/z* 327.1 [M + H]^+^.

To a solution of *N*-(5-amino-2-chlorobenzyl)-4-methoxybenzenesulfonamide (108 mg, 0.33 mmol) in dichloromethane (2 mL) were added pyridine (39 mg, 0.50 mmol) and 4-methoxybenzenesulfonyl chloride (102 mg, 0.50 mmol) at room temperature. The reaction mixture was stirred at room temperature overnight. After the reaction was completed, the crude mixture was added to 1N HCl and extracted with dichloromethane. The organic layer was dried over anhydrous Na_2_SO_4_ and concentrated under reduced pressure. The residue was purified by flash column chromatograph (0-30% ethyl acetate/hexane) to give the product as a pale yellow solid (125 mg, 77%); ^1^H NMR (400 MHz, DMSO-*d_6_*) *δ* 10.35 (brs, 1H, NH), 8.07 (t, *J* = 6.4 Hz, 1H, NH), 7.73 (d, *J* = 8.8 Hz, 4H), 7.33 (d, *J* = 2.8 Hz, 1H), 7.23 (d, *J* = 8.8 Hz, 1H), 7.09 (d, *J* = 8.8 Hz, 2H), 7.06 (d, *J* = 8.8 Hz, 2H), 6.98 (dd, *J* = 8.8, 2.8 Hz, 1H), 3.86 (d, *J* = 6.4 Hz, 2H), 3.84 (s, 3H), 3.79 (s, 3H); ^13^C NMR (100 MHz, DMSO-*d_6_*) *δ* 162.5, 162.2, 137.1, 135.9, 131.9, 130.9, 129.6, 129.0, 128.7, 126.6, 120.5, 119.5, 114.5, 114.4, 55.6, 43.4; LC/MS (ESI) *m/z* 497.1 [M + H]^+^.

*N-(4-Bromo-3-(((4-methoxyphenyl)sulfonamido)methyl)phenyl)-4-methoxybenzenesulfonamide (****23b****).* Prepared as described in the experimental procedure of **23a** from intermediate **22b** to obtain *N*-(5-amino-2-bromobenzyl)-4-methoxybenzenesulfonamide as a black solid (70 mg, 37%); ^1^H NMR (400 MHz, DMSO-*d_6_*) *δ* 8.02 (brs, 1H, NH), 7.77 (d, *J* = 8.4 Hz, 2H), 7.14-7.10 (m, 3H), 6.73 (s, 1H), 6.40 (d, *J* = 8.8 Hz, 1H), 5.33 (s, 2H, NH), 3.84 (s, 3H), 3.78 (s, 2H); ^13^C NMR (100 MHz, DMSO-*d_6_*) *δ* 162.2, 148.4, 136.3, 132.3, 132.0, 128.7, 114.8, 114.6, 114.4, 106.5, 55.6, 46.1; LC/MS (ESI) *m/z* 371.0 [M(^79^Br) + H]^+^, 373.0 [M(^81^Br) + H]^+^.

Prepared as described in the experimental procedure of **23a** from *N*-(5-amino-2-bromobenzyl)-4-methoxybenzenesulfonamide to obtain the title compound as a white solid (13 mg, 13%); ^1^H NMR (400 MHz, CDCl_3_) *δ* 8.48 (brs, 1H, NH), 7.70 (d, *J* = 9.2 Hz, 2H), 7.69 (d, *J* = 9.2 Hz, 2H), 8.21 (d, *J* = 8.8 Hz, 1H), 7.07 (d, *J* = 2.8 Hz, 1H), 6.91-6.83 (m, 3H), 5.99 (t, *J* = 6.4 Hz, 1H, NH), 4.02 (d, *J* = 6.4 Hz, 2H), 3.80 (s, 3H), 3.76 (s, 3H); ^13^C NMR (100 MHz, CDCl_3_) *δ* 163.2, 163.0, 137.1, 136.9, 133.3, 131.4, 130.6, 129.5, 129.2, 121.7, 120.9, 117.9, 114.4, 114.3, 55.7, 55.6, 46.9; LC/MS (ESI) *m/z* 541.0 [M(^79^Br) + H]^+^, 543.0 [M(^81^Br) + H]^+^.

*N-(4-Methoxy-3-(((4-methoxyphenyl)sulfonamido)methyl)phenyl)-4-methoxybenzenesulfonamide (****23c****).* Prepared as described in the experimental procedure of **23a** from intermediate **22c** to obtain *N*-(5-amino-2-methoxybenzyl)-4-methoxybenzenesulfonamide as a beige solid (328 mg, 75%); ^1^H NMR (400 MHz, CDCl_3_) *δ* 7.70 (d, *J* = 8.8 Hz, 2H), δ 6.87 (d, *J* = 8.8 Hz, 2H), 6.53-6.51 (m, 2H), 6.45 (d, *J* = 2.0 Hz, 1H), 5.37 (s, 1H, NH), 4.00 (s, 2H, NH), 3.82 (s, 3H), 3.62 (s, 3H), 3.54 (s, 2H); ^13^C NMR (100 MHz, CDCl_3_) *δ* 162.7, 150.4, 139.6, 131.9, 129.6, 125.2, 117.5, 115.4, 114.0, 111.4, 55.7, 43.7; LC/MS (ESI) *m/z* 321.1 [M + H]^+^.

Prepared as described in the experimental procedure of **23a** from *N*-(5-amino-2-methoxy benzyl)-4-methoxybenzenesulfonamide to obtain the title compound as a pale yellow solid (399 mg, 96%); ^1^H NMR (400 MHz, DMSO-*d_6_*) *δ* 9.87 (brs, 1H, NH), 7.86 (t, *J* = 5.6 Hz, 1H, NH), 7.75-7.70 (m, 4H), 7.21 (s, 1H), 7.08-7.03 (m, 4H), 6.93 (d, *J* = 8.8 Hz, 1H), 7.74 (d, *J* = 8.8 Hz, 1H), 3.81 (s, 5H), 3.77 (s, 3H), 3.60 (s, 3H); ^13^C NMR (100 MHz, DMSO-*d_6_*) *δ* 162.4, 162.1, 153.5, 132.2, 131.4, 130.3, 129.0, 128.8, 126.1, 122.4, 121.4, 114.3, 114.3, 110.8, 55.6, 55.6, 55.4, 40.9; LC/MS (ESI) *m/z* 493.2 [M + H]^+^.

*N-(4-Methyl-3-(((4-methoxyphenyl)sulfonamido)methyl)phenyl)-4-methoxybenzenesulfonamide (****23d****).* To a solution of **22d** (57 mg, 0.45 mmol) in methanol (15 mL) was added Pd/C (10% wt.) (5 mg) at room temperature under N_2_. The reaction mixture was stirred at room temperature for 2 hours under H_2_. After the reaction was completed, the crude mixture was filtered off through celite, and then concentrated under reduced pressure to give *N*-(5-amino-2-methylbenzyl)-4-methoxybenzenesulfonamide, as a pale yellow oil. The resulting crude compound was used for the following reaction without further purification.

To a solution of *N*-(5-amino-2-methylbenzyl)-4-methoxybenzenesulfonamide (78 mg, 0.25 mmol) in dichloromethane (1.5 mL) were added pyridine (60 mg, 0.76 mmol) and 4-methoxybenzenesulfonyl chloride (131 mg, 0.63 mmol) at room temperature. The reaction mixture was stirred at room temperature overnight. After the reaction was completed, the crude mixture was added to 1N HCl and extracted with dichloromethane. The organic layer was dried over anhydrous Na_2_SO_4_ and concentrated under reduced pressure. The residue was purified by flash column chromatograph (0-40% ethyl acetate/hexane) to give the product as a colorless oil (90 mg, quantitative over two steps); ^1^H NMR (400 MHz, DMSO-*d_6_*) *δ* 10.01 (brs, 1H, NH), 7.82 (t, *J* = 6.0 Hz, 1H, NH), 7.73 (d, *J* = 8.8 Hz, 2H), 7.68 (d, *J* = 8.8 Hz, 2H), 7.11-7.07 (m, 3H), 7.03 (d, *J* = 8.8 Hz, 2H), 6.94 (d, *J* = 8.0 Hz, 1H), 6.86 (dd, *J* = 8.0, 2.4 Hz, 1H), 3.84 (s, 3H), 3.78 (s, 3H), 3.74 (d, *J* = 6.0 Hz, 2H), 2.05 (s, 3H); ^13^C NMR (100 MHz, DMSO-*d_6_*) *δ* 162.3, 162.1, 136.2, 135.7, 132.0, 131.4, 131.3, 130.5, 128.9, 128.7, 120.5, 118.7, 114.3, 55.6, 55.6, 44.1, 17.7; LC/MS (ESI) *m/z* 477.2 [M + H]^+^.

*N-(4-Fluoro-3-(((4-methoxyphenyl)sulfonamido)methyl)phenyl)-4-methoxybenzenesulfonamide (****23e****).* Prepared as described in the experimental procedure of **23d** from intermediate **22e** to obtain *N*-(5-amino-2-fluorobenzyl)-4-methoxybenzenesulfonamide as a pale pink solid (137 mg, 75%); ^1^H NMR (400 MHz, DMSO-*d_6_*) *δ* 7.88 (brs, 1H, NH), 7.74 (d, *J* = 8.8 Hz, 2H), 7.10 (d, *J* = 8.8 Hz, 2H), 6.78-6.73 (m, 1H), 6.57-6.55 (m, 1H), 6.43-6.40 (m, 1H), 4.96 (s, 2H, NH), 3.84 (s, 3H), 3.78 (s, 2H); ^13^C NMR (100 MHz, DMSO-*d_6_*) *δ* 162.1, 151.8 (*J*_C,F_ = 230 Hz), 145.0, 132.1, 128.7, 124.2 (*J*_C,F_ = 15 Hz), 115.1, 114.9, 114.4 (*J*_C,F_ = 17 Hz), 113.6 (*J*_C,F_ = 7 Hz), 55.6; LC/MS (ESI) *m/z* 311.1 [M + H]^+^.

Prepared as described in the experimental procedure of **23d** from *N*-(5-amino-2-fluorobenzyl)-4-methoxybenzenesulfonamide to obtain the title compound as a pale yellow solid (183 mg, 93%); H NMR (400 MHz, DMSO-*d_6_*) *δ* 10.13 (brs, 1H, NH), 8.03 (t, *J* = 5.6 Hz, 1H, NH), 7.72-7.68 (m, 4H), 7.22-7.20 (m, 1H), 7.07-7.04 (m, 4H), 6.99-6.96 (m, 2H), 3.86 (d, *J* = 5.6 Hz, 2H), 3.83 (s, 3H), 3.79 (s, 3H); ^13^C NMR (100 MHz, DMSO-*d_6_*) *δ* 162.4, 162.2, 156.5 (*J*_C,F_ = 241 Hz), 134.0, 131.9, 131.0, 128.9, 128.7, 125.4 (*J*_C,F_ = 16 Hz), 122.2, 121.0 (*J*_C,F_ = 8 Hz), 115.6 (*J*_C,F_ = 22 Hz), 114.4, 114.3, 55.6; LC/MS (ESI) *m/z* 481.1 [M + H]^+^.

*N-(4-(Benzyloxy)-3-(((4-methoxyphenyl)sulfonamido)methyl)phenyl)-4-methoxybenzenesulfon-amine (****23f****).* Prepared as described in the experimental procedure of **23a** from intermediate **22f** to obtain *N*-(5-amino-2-(benzyloxy)benzyl)-4-methoxybenzenesulfonamide as a dark brown oil (176 mg, 95%); ^1^H NMR (400 MHz, DMSO-*d_6_*) *δ* 7.74 (d, *J* = 8.8 Hz, 2H), 7.68 (t, *J* = 6.0 Hz, 1H, NH), 7.36-7.29 (m, 5H), 7.08 (d, *J* = 8.8 Hz, 2H), 6.72 (d, *J* = 8.8 Hz, 1H), 6.61 (d, *J* = 2.4 Hz, 1H), 6.40 (dd, *J* = 8.8, 2.4 Hz, 1H), 4.88 (s, 2H), 4.70 (s, 2H, NH), 3.83 (s, 3H), 3.82-3.80 (m, 2H); ^13^C NMR (100 MHz, DMSO-*d_6_*) *δ* 162.0, 146.8, 142.6, 137.7, 132.0, 128.7, 128.2, 127.5, 127.2, 126.4, 114.8, 114.2, 114.1, 113.2, 70.4, 55.6, 41.0; LC/MS (ESI) *m/z* 399.2 [M + H]^+^.

Prepared as described in the experimental procedure of **23a** from *N*-(5-amino-2-(benzyloxy) benzyl)-4-methoxybenzenesulfonamide to obtain the title compound as a pale yellow solid (114 mg, 44%); ^1^H NMR (400 MHz, DMSO-*d_6_*) *δ* 9.84 (brs, 1H, NH), 7.82 (t, *J* = 6.0 Hz, 1H, NH), 7.70 (d, *J* = 8.8 Hz, 2H), 7.65 (d, *J* = 9.2 Hz, 2H), 7.36-7.30 (m, 5H), 7.17 (s, 1H), 7.04 (d, *J* = 8.8 Hz, 2H), 7.04 (d, *J* = 9.2 Hz, 2H), 6.86-6.85 (m, 2H), 3.82 (s, 2H), 3.81 (s, 3H), 3.79 (s, 3H); ^13^C NMR (100 MHz, DMSO-*d_6_*) *δ* 162.3, 162.1, 152.4, 137.0, 131.9, 131.3, 130.6, 128.9, 128.7, 128.3, 127.7, 127.2, 126.5, 122.3, 121.1, 114.3, 114.2, 112.4, 69.5, 55.6, 55.6, 40.6; LC/MS (ESI) *m/z* 569.2 [M + H]^+^.

*Ethyl N-(4-chloro-3-(((N-(2-ethoxy-2-oxoethyl)-4-methoxyphenyl)sulfonamido) methyl)phenyl)-N-((4-methoxyphenyl)sulfonyl)glycinate (****24a****).* To a solution of **23a** (100 mg, 0.20 mmol) in *N,N*-dimethylformamide (1 mL) were added potassium carbonate (84 mg, 0.60 mmol) and ethyl 2-bromoacetate (101 mg, 0.60 mmol) at room temperature. The reaction mixture was stirred at room temperature overnight. After the reaction was completed, the crude mixture was diluted with water and extracted with ethyl acetate. The organic layer was dried over anhydrous Na_2_SO_4_ and concentrated under reduced pressure. The residue was purified by flash column chromatograph (0-30% ethyl acetate/hexane) to give the product as a colorless oil (119 mg, 88%); ^1^H NMR (400 MHz, CDCl_3_) *δ* 7.76 (d, *J* = 8.8 Hz, 2H), 7.64 (d, *J* = 8.8 Hz, 2H), 7.28-7.27 (m, 2H), 7.18 (s, 1H), 7.00-6.95 (m, 4H), 4.50 (s, 2H), 4.36 (s, 2H), 4.16 (q, *J* = 7.2 Hz, 2H), 4.04 (q, *J* = 7.2 Hz, 2H), 3.88 (s, 6H), 3.83 (s, 2H), 1.24 (t, *J* = 7.2 Hz, 3H), 1.17 (t, *J* = 7.2 Hz, 3H); ^13^C NMR (100 MHz, CDCl_3_) *δ* 168.7, 168.6, 163.4, 163.2, 139.3, 134.4, 133.2, 131.2, 130.4, 130.2, 130.0, 129.7, 129.2, 114.4, 114.3, 61.7, 61.5, 55.7, 52.5, 50.9, 48.7, 47.8, 14.2, 14.1; LC/MS (ESI) *m/z* 669.2 [M + H]^+^.

*Ethyl N-(4-bromo-3-(((N-(2-ethoxy-2-oxoethyl)-4-methoxyphenyl)sulfonamido) methyl)phenyl)-N-((4-methoxyphenyl)sulfonyl)glycinate (****24b****).* Prepared as described in the experimental procedure of **24a** from intermediate **23b** to obtain the title compound as a colorless oil (27 mg, quantitative); ^1^H NMR (400 MHz, CDCl_3_) *δ* 7.75 (d, *J* = 8.8 Hz, 2H), 7.63 (d, *J* = 8.8 Hz, 2H), 7.46 (d, *J* = 8.4 Hz, 1H), 7.21-7.16 (m, 2H), 6.98-6.94 (m, 4H), 4.47 (s, 2H), 4.34 (s, 2H), 4.15 (q, *J* = 7.2 Hz, 2H), 4.02 (q, *J* = 7.2 Hz, 2H), 3.87 (s, 2H), 3.86 (s, 6H), 1.25-1.14 (m, 6H); ^13^C NMR (100 MHz, CDCl_3_) δ 168.7, 168.6, 163.4, 163.2, 140.0, 136.1, 133.7, 131.2, 130.4, 130.2, 130.0, 129.7, 129.0, 122.9, 114.4, 114.3, 61.7, 61.5, 55.7, 52.4, 51.2, 47.9, 14.2, 14.1; LC/MS (ESI) *m/z* 713.1 [M(^79^Br) + H]^+^, 715.1 [M(^81^Br) + H]^+^.

*Ethyl N-(4-methoxy-3-(((N-(2-ethoxy-2-oxoethyl)-4-methoxyphenyl)sulfonamido)methyl)phenyl)-N-((4-methoxyphenyl)sulfonyl)glycinate (****24c****).* Prepared as described in the experimental procedure of **24a** from intermediate **23c** to obtain the title compound as a colorless oil (220 mg, 82%); ^1^H NMR (400 MHz, CDCl_3_) *δ* 7.65 (d, *J* = 8.4 Hz, 2H), 7.55 (d, *J* = 8.4 Hz, 2H), 7.21 (dd, *J* = 8.4, 2.0 Hz, 1H), 6.90-6.87 (m, 5H), 6.69 (d, *J* = 8.4 Hz, 1H), 4.31 (s, 2H), 4.24 (s, 2H), 4.08 (q, *J* = 7.2 Hz, 2H), 3.98 (q, *J* = 7.2 Hz, 2H), 3.80 (s, 3H), 3.79 (s, 3H), 3.77 (s, 2H), 3.63 (s, 3H), 1.16 (t, *J* = 7.2 Hz, 3H), 1.10 (t, *J* = 7.2 Hz, 3H); ^13^C NMR (100 MHz, CDCl_3_) *δ* 168.8, 168.7, 163.0, 162.8, 157.1, 132.4, 131.6, 130.9, 130.5, 129.7, 129.4, 124.3, 114.0, 113.9, 110.6, 61.3, 61.0, 55.5, 55.3, 52.8, 47.6, 45.7, 14.0, 13.9; LC/MS (ESI) *m/z* 665.2 [M + H]^+^.

*Ethyl N-(4-methyl-3-(((N-(2-ethoxy-2-oxoethyl)-4-methoxyphenyl)sulfonamido) methyl)phenyl)-N-((4-methoxyphenyl)sulfonyl)glycinate (****24d****).* Prepared as described in the experimental procedure of **24a** from intermediate **23d** to obtain the title compound as a colorless oil (89 mg, 83%); ^1^H NMR (400 MHz, CDCl_3_) *δ* 7.77 (d, *J* = 8.4 Hz, 2H), 7.59 (d, *J* = 9.2 Hz, 2H), 7.07-7.06 (m, 2H), 7.00-6.97 (m, 3H), 6.91 (d, *J* = 8.8 Hz, 2H), 4.42 (s, 2H), 4.30 (s, 2H), 4.13-4.11 (m, 2H), 3.98-3.96 (m, 2H), 3.88 (s, 3H), 3.86 (s, 3H), 3.70 (s, 2H), 2.27 (s, 3H), 1.27-1.21 (m, 3H), 1.19-1.12 (m, 3H); ^13^C NMR (100 MHz, CDCl_3_) *δ* 168.9, 168.7, 163.2, 138.1, 138.0, 133.9, 131.6, 131.0, 130.7, 130.0, 129.8, 129.5, 128.7, 114.3, 114.1, 61.5, 61.4, 55.8, 55.7, 52.7, 49.3, 46.9, 18.9, 14.2, 14.1; LC/MS (ESI) *m/z* 649.1 [M + H]^+^.

*Ethyl N-(4-fluoro-3-(((N-(2-ethoxy-2-oxoethyl)-4-methoxyphenyl)sulfonamido)methyl)phenyl)-N-((4-methoxyphenyl)sulfonyl)glycinate (****24e****).* Prepared as described in the experimental procedure of **24a** from intermediate **23e** to obtain the title compound as a colorless oil (146 mg, 76%); ^1^H NMR (400 MHz, CDCl_3_) *δ* 7.73 (d, *J* = 8.8 Hz, 2H), 7.61 (d, *J* = 8.8 Hz, 2H), 7.31-7.27 (m, 1H), 7.10-7.08 (m, 1H), 6.97-6.93 (m, 5H), 4.41 (s, 2H), 4.36 (s, 2H), 4.14 (q, *J* = 7.2 Hz, 2H), 4.02 (q, *J* = 7.2 Hz, 2H), 3.86 (s, 3H), 3.86 (s, 3H), 3.82 (s, 2H), 1.22 (t, *J* = 7.2 Hz, 3H), 1.15 (t, *J* = 7.2 Hz, 3H); ^13^C NMR (100 MHz, CDCl_3_) *δ* 168.7, 168.6, 163.3, 163.2, 160.4 (*J*_C,F_ = 248 Hz), 136.4, 131.6 (*J*_C,F_ = 8 Hz), 131.2, 130.7 (*J*_C,F_ = 5 Hz), 130.3, 130.0, 129.7, 123.7 (*J*_C,F_ = 15 Hz), 116.3 (*J*_C,F_ = 23 Hz), 114.3, 114.3, 61.6, 61.4, 55.7, 52.7, 47.6, 44.7, 14.2, 14.1; LC/MS (ESI) *m/z* 653.2 [M + H]^+^.

*Ethyl N-(4-(benzyloxy)-3-(((N-(2-ethoxy-2-oxoethyl)-4-methoxyphenyl)sulfonamido)methyl)phenyl)-N-((4-methoxyphenyl)sulfonyl)glycinate (****24f****).* Prepared as described in the experimental procedure of **24a** from intermediate **23f** to obtain the title compound as a colorless oil (119 mg, 91%); ^1^H NMR (400 MHz, CDCl_3_) *δ* 7.69 (d, *J* = 8.8 Hz, 2H), 7.63 (d, *J* = 8.8 Hz, 2H), 7.37-7.26 (m, 6H), 7.00 (d, *J* = 2.4 Hz, 1H), 6.95 (d, *J* = 8.8 Hz, 2H), 6.91 (d, *J* = 8.8 Hz, 2H), 6.82 (d, *J* = 8.8 Hz, 1H), 4.97 (s, 2H), 4.42 (s, 2H), 4.31 (s, 2H), 4.14 (q, *J* = 7.2 Hz, 2H), 3.90 (q, *J* = 7.2 Hz, 2H), 3.86 (s, 3H), 3.84 (s, 3H), 3.81 (s, 2H), 1.23 (t, *J* = 7.2 Hz, 3H), 1.07 (t, *J* = 7.2 Hz, 3H); ^13^C NMR (100 MHz, CDCl_3_) *δ* 168.9, 163.2, 162.9, 156.5, 136.3, 133.0, 131.8, 131.2, 130.7, 130.1, 130.0, 129.6, 128.7, 128.3, 127.5, 125.1, 114.2, 114.1, 112.2, 70.5, 61.5, 61.2, 55.68, 55.66, 52.9, 48.0, 46.0, 14.2, 14.0; LC/MS (ESI) *m/z* 741.2 [M + H]^+^.

*Ethyl N-(5-((N-(2-ethoxy-2-oxoethyl)-4-methoxyphenyl)sulfonamido)-2-hydroxybenzyl)-N-((4-methoxy-phenyl)sulfonyl)glycinate (****25****).* To a solution of **24f** (83 mg, 1.44 mmol) in methanol (5 mL) was added Pd/C (10% wt.) (8 mg) at room temperature under N_2_. The reaction mixture was stirred at room temperature for 1 hour under H_2_. After the reaction was completed, the crude mixture was filtered off through celite and then concentrated under reduced pressure. The residue was purified by flash column chromatograph (0-60% ethyl acetate/hexane) to give the product as a pale yellow solid (62 mg, 85%); ^1^H NMR (400 MHz, DMSO-*d_6_*) *δ* 9.93 (brs, 1H, OH), 7.68 (d, *J* = 8.8 Hz, 2H), 7.56 (d, *J* = 8.8 Hz, 2H), 7.09-7.05 (m, 4H), 6.94 (d, *J* = 8.4 Hz, 1H), 6.71-6.69 (m, 2H), 4.25 (s, 4H), 4.08 (q, *J* = 7.2 Hz, 2H), 3.94 (q, *J* = 7.2 Hz, 2H), 3.85 (s, 6H), 3.77 (s, 2H), 1.15 (t, *J* = 7.2 Hz, 3H), 1.08 (t, *J* = 7.2 Hz, 3H); ^13^C NMR (100 MHz, DMSO-*d_6_*) *δ* 168.6, 168.5, 162.7, 162.5, 155.2, 131.1, 130.8, 130.1, 129.8, 129.5, 129.3, 129.2, 122.1, 115.3, 114.4, 114.3, 60.9, 60.7, 55.7, 55.7, 52.7, 47.7, 45.7, 13.9, 13.8; LC/MS (ESI) *m/z* 651.2 [M + H]^+^.

*N-(2-Chloro-5-nitrobenzyl)benzenesulfonamide (****26****).* Prepared as described in the experimental procedure of **22a** from intermediate **21a** and benzenesulfonamide to obtain the title compound as a pale yellow solid (483 mg, 46%); ^1^H NMR (400 MHz, DMSO-*d_6_*) *δ* 8.52 (brs, 1H, NH), 8.21 (d, *J* = 2.4 Hz, 1H), 8.09 (dd, *J* = 8.8, 2.4 Hz, 1H), 7.78 (d, *J* = 8.0 Hz, 2H), 7.68 (d, *J* = 8.8 Hz, 1H), 7.62-7.58 (m, 1H), 7.54-7.52 (m, 2H), 4.22 (s, 2H); ^13^C NMR (100 MHz, DMSO-*d_6_*) *δ* 146.2, 140.4, 138.9, 137.1, 132.5, 130.6, 129.2, 126.4, 124.1, 123.7, 43.2; LC/MS (ESI) *m/z* 325.0 [M - H]^-^.

*N-(2-Chloro-5-nitrobenzyl)-N-methylbenzenesulfonamide (****27****).* To a solution of **26** (100 mg, 0.31 mmol) in *N,N*-dimethylformamide (1 mL) were added potassium carbonate (63 mg, 0.46 mmol) and methyl iodide (65 mg, 0.46 mmol) at room temperature. The reaction mixture was stirred at room temperature for 3 hours. After the reaction was completed, the crude mixture was added to water and extracted with ethyl acetate. The organic layer was dried over anhydrous Na_2_SO_4_ and concentrated under reduced pressure. The residue was purified by flash column chromatograph (0-30% ethyl acetate/hexane) to give the product as a pale yellow solid (88 mg, 84%); ^1^H NMR (400 MHz, CDCl_3_) *δ* 8.35 (d, *J* = 2.4 Hz, 1H), 8.09 (dd, *J* = 8.4, 2.4 Hz, 1H), 7.88-7.85 (m, 2H), 7.68-7.64 (m, 1H), 7.61-7.57 (m, 2H), 7.53 (d, *J* = 8.4 Hz, 1H), 4.37 (s, 2H), 2.77 (s, 3H); ^13^C NMR (100 MHz, CDCl_3_) *δ* 147.2, 140.1, 137.2, 136.0, 133.3, 130.7, 129.5, 127.5, 124.4, 123.9, 51.3, 35.8; LC/MS (ESI) *m/z* 341.1 [M + H]^+^.

*N-(2-Chloro-5-((4-methoxyphenyl)sulfonamido)benzyl)-N-methylbenzenesulfonamide(****28****).* Prepared as described in the experimental procedure of **23a** from intermediate **27** to obtain *N*-(5-amino-2-chlorobenzyl)-*N*-methylbenzenesulfonamide as a pale yellow oil (63 mg, 95%); ^1^H NMR (400 MHz, CDCl_3_) *δ* 7.85 (d, *J* = 7.2 Hz, 2H), 7.64-7.55 (m, 3H), 7.06 (d, *J* = 8.8 Hz, 1H), 6.86 (d, *J* = 2.8 Hz, 1H), 6.53 (dd, *J* = 8.8, 2.8 Hz, 1H), 4.22 (s, 2H), 2.68 (s, 3H); ^13^C NMR (100 MHz, CDCl_3_) *δ* 145.9, 137.6, 133.9, 132.9, 130.1, 129.3, 127.5, 122.2, 115.9, 115.8, 51.0, 35.1; LC/MS (ESI) *m/z* 311.1 [M + H]^+^.

Prepared as described in the experimental procedure of **23a** from *N*-(5-amino-2-chlorobenzyl)-*N*-methylbenzenesulfonamide to obtain the title compound as a white solid (71 mg, 84%); ^1^H NMR (400 MHz, CDCl_3_) *δ* 7.84 (d, *J* = 7.2 Hz, 2H), 7.75 (d, *J* = 8.8 Hz, 2H), 7.65-7.55 (m, 3H), 7.20-7.18 (m, 2H), 7.11 (dd, *J* = 8.8, 2.8 Hz, 1H), 6.89 (d, *J* = 8.8 Hz, 2H), 4.18 (s, 2H), 3.80 (s, 2H), 2.57 (s, 3H); ^13^C NMR (100 MHz, CDCl_3_) *δ* 163.3, 137.3, 136.5, 134.4, 133.1, 130.4, 130.3, 129.7, 129.4, 129.3, 127.5, 121.8, 121.6, 114.4, 55.7, 51.1, 35.1; LC/MS (ESI) *m/z* 481.1 [M + H]^+^.

*Ethyl N-(4-chloro-3-((N-methylphenylsulfonamido)methyl)phenyl)-N-((4-methoxyphenyl)sulfonyl)-glycinate (****29****).* To a solution of **28** (57 mg, 0.12 mmol) in *N,N*-dimethylformamide (1 mL) were added potassium carbonate (25 mg, 0.18 mmol) and ethyl 2-bromoacetate (30 mg, 0.18 mmol) at room temperature. The reaction mixture was stirred at room temperature overnight. After the reaction was completed, the crude mixture was added to water and extracted with ethyl acetate. The organic layer was dried over anhydrous Na_2_SO_4_ and concentrated under reduced pressure. The residue was purified by flash column chromatograph (0-30% ethyl acetate/hexane) to give the product as a colorless oil (71 mg, quantitative); ^1^H NMR (400 MHz, CDCl_3_) *δ* 7.78 (d, *J* = 8.8 Hz, 2H), 7.63-7.52 (m, 5H), 7.28-7.27 (m, 1H), 7.23-7.17 (m, 2H), 6.93 (d, *J* = 8.8 Hz, 2H), 4.34 (s, 2H), 4.15 (s, 2H), 4.11 (q, *J* = 7.2 Hz, 2H), 3.84 (s, 3H), 2.53 (s, 3H), 1.20 (t, *J* = 7.2 Hz, 3H); ^13^C NMR (100 MHz, CDCl_3_) *δ* 168.5, 163.3, 139.2, 137.1, 134.4, 133.0, 133.0, 130.3, 130.0, 129.8, 129.3, 128.9, 127.4, 114.3, 612, 55.7, 52.5, 51.0, 35.0, 14.1; LC/MS (ESI) *m/z* 567.1 [M + H]^+^.

*1-Methyl-5-nitro-1H-benzo[d][1,2,3]triazole (****30****).* To a solution of *N^1^*-methyl-4-nitro-1,2-phenylene-diamine (200 mg, 1.20 mmol) in con. HCl (1 mL) and water (4 mL) were added to sodium nitrite (104 mg, 1.51 mmol) in water (2 mL) at 0 °C. The reaction mixture was stirred at 0 °C for 3 hours. After the reaction was completed, the crude mixture was warmed to room temperature and basified with sat. NaOH until the pH reached around 7. The resulting solid was filtered to give the product as a dark brown solid (175 mg, 82%); ^1^H NMR (400 MHz, CDCl_3_) *δ* 9.01 (s, 1H), 8.42 (dd, *J* = 8.8, 2.0 Hz, 1H), 7.64 (d, *J* = 8.8 Hz, 1H), 4.39 (s, 3H); ^13^C NMR (100 MHz, CDCl_3_) *δ* 145.2, 144.9, 136.2, 122.7, 117.5, 109.9, 34.8; LC/MS (ESI) *m/z* 179.1 [M + H]^+^.

*1-Methyl-1H-benzo[d][1,2,3]triazol-5-amine (****31****).* To a solution of **30** (355 mg, 0.47 mmol) in methanol (20 mL) was added Pd/C (10% wt.) (36 mg) at room temperature under N_2_. The reaction mixture was stirred at room temperature for 1 hour under H_2_. After the reaction was completed, the crude mixture was filtered off through celite, and then concentrated under reduced pressure to give the product as a brown solid (311 mg, quantitative); ^1^H NMR (400 MHz, DMSO-*d_6_*) *δ* 7.48 (d, *J* = 8.4 Hz, 1H), 6.94 (d, *J* = 8.4 Hz, 1H), 6.91 (s, 1H), 5.22 (s, 1H, NH), 4.16 (s, 3H), 3.36 (s, 1H, NH); ^13^C NMR (100 MHz, DMSO-*d_6_*) *δ* 146.9, 145.8, 129.2, 119.0, 110.1, 97.5, 33.9; LC/MS (ESI) *m/z* 149.2 [M + H]^+^.

*(5-Bromo-2-methylphenyl)methanol (****32****).* Prepared as described in the experimental procedure of **20a** from 5-bromo-2-methylbenzoic acid to obtain the title compound as a pale yellow solid (830 mg, 89%); ^1^H NMR (400 MHz, CDCl_3_) *δ* 7.51 (d, *J* = 2.0 Hz, 1H), 7.31 (dd, *J* = 8.0, 2.0 Hz, 1H), 7.02 (d, *J* = 8.0 Hz, 1H), 4.63 (s, 2H), 3.26 (s, 3H); ^13^C NMR (100 MHz, CDCl_3_) *δ* 140.9, 134.7, 132.0, 130.5, 130.0, 119.7, 62.8, 18.3.

*5-Bromo-2-methylbenzaldehyde (****33****).* Prepared as described in the experimental procedure of **21a** from intermediate **32** to obtain the title compound as a colorless oil (473 mg, 94%); ^1^H NMR (400 MHz, CDCl_3_) *δ* 10.19 (s, 1H), 7.89 (d, *J* = 2.4 Hz, 1H), 7.56 (dd, *J* = 8.0, 2.4 Hz, 1H), 7.13 (d, *J* = 8.0 Hz, 1H), 2.60 (s, 3H); ^13^C NMR (100 MHz, CDCl_3_) *δ* 191.1, 139.4, 136.5, 135.6, 134.2, 133.6, 120.2, 19.0.

*N-(5-Bromo-2-methylbenzyl)-4-methoxybenzenesulfonamide (****34****).* Prepared as described in the experimental procedure of **22a** from intermediate **33** to obtain the title compound as a white solid (154 mg, 59%); ^1^H NMR (400 MHz, CDCl_3_) *δ* 7.77 (d, *J* = 8.8 Hz, 2H), 7.23 (dd, *J* = 8.0, 2.0 Hz, 1H), 7.19 (d, *J* = 2.0 Hz, 1H), 6.98-6.95 (m, 3H), 7.17 (d, *J* = 8.0 Hz, 2H), 4.05 (d, *J* = 6.0 Hz, 2H), 3.88 (s, 3H), 2.20 (s, 3H); ^13^C NMR (100 MHz, CDCl_3_) *δ* 163.2, 136.3, 135.6, 132.3, 131.6, 131.3, 131.1, 129.4, 119.7, 114.5, 55.8, 45.0, 18.5; LC/MS (ESI) *m/z* 370.1 [M(^79^Br) + H]^+^, 372.0 [M(^81^Br) + H]^+^.

*4-Methoxy-N-(2-methyl-5-((1-methyl-1H-benzo[d][1,2,3]triazol-5-yl)amino)benzyl)benzenesulfon-amide (****35****).* To a solution of **34** (281 mg, 0.76 mmol) in toluene (3.6 mL) were added intermediate **31** (107 mg, 0.72 mmol), Tris(dibenzylideneacetone)dipalladium(0) (66 mg, 0.07 mmol), 2-di-*tert*-butylphosphino-2′,4′,6′-triisopropylbiphenyl (61 mg, 0.14 mmol) and sodium *tert*-butoxide (104 mg, 1.08 mmol) at room temperature. The reaction mixture was stirred at 80 °C for overnight. After the reaction was completed, the crude mixture was cooled to room temperature, filtered off through celite, and then concentrated under reduced pressure. The residue was purified by flash column chromatograph (0-60% ethyl acetate/hexane) to give the product as a pale yellow solid (156 mg, 47%); ^1^H NMR (400 MHz, CDCl_3_) *δ* 7.79 (d, *J* = 8.8 Hz, 2H), 7.54 (d, *J* = 2.0 Hz, 1H), 7.36 (d, *J* = 8.8 Hz, 1H), 7.16 (dd, *J* = 8.8, 2.0 Hz, 1H), 7.01 (d, *J* = 8.0 Hz, 1H), 6.94-6.90 (m, 4H), 5.93 (brs, 1H, NH), 4.24 (s, 3H), 4.04 (d, *J* = 6.0 Hz, 2H), 3.83 (s, 3H), 2.17 (s, 3H); ^13^C NMR (100 MHz, CDCl_3_) *δ* 163.0, 147.3, 141.5, 140.5, 135.4, 131.6, 131.5, 129.7, 129.4, 129.3, 121.9, 118.6, 117.6, 114.4, 110.0, 104.8, 55.8, 45.4, 34.4, 18.2; LC/MS (ESI) *m/z* 438.2 [M + H]^+^.

*tert-Butyl N-(5-((2-(tert-butoxy)-2-oxoethyl)(1-methyl-1H-benzo[d][1,2,3]triazol-5-yl)amino)-2-methylbenzyl)-N-((4-methoxyphenyl)sulfonyl)glycinate (****36****).* Prepared as described in the experimental procedure of **24a** from intermediate **35** and *tert*-butyl 2-bromoacetate to obtain the title compound as a colorless oil (10 mg, 4%); %); ^1^H NMR (400 MHz, CDCl_3_) *δ* 7.76 (d, *J* = 8.8 Hz, 2H), 7.50 (d, *J* = 2.0 Hz, 1H), 7.34 (d, *J* = 8.8 Hz, 1H), 7.21 (dd, *J* = 8.8, 2.0 Hz, 1H), 7.01 (d, *J* = 8.0 Hz, 1H), 6.91 (d, *J* = 8.8 Hz, 2H), 6.84 (dd, *J* = 8.0, 2.4 Hz, 1H), 6.79 (d, *J* = 2.4 Hz, 1H), 4.24 (s, 2H), 4.24 (s, 2H), 4.24 (s, 3H), 3.83 (s, 3H), 4.24 (s, 2H), 4.24 (s, 3H), 4.24 (s, 9H), 2.17 (s, 9H); LC/MS (ESI) *m/z* 667.3 [M + H]^+^.

*4-Methoxy-N-(3-((4-methoxyphenyl)sulfonamido)benzyl)benzenesulfonamide (****37a****).* To a solution of 3-(aminomethyl)aniline (100 mg, 0.82 mmol) in dichloromethane (4 mL) were added pyridine (194 mg, 2.46 mmol) and 4-methoxybenzenesulfonyl chloride (507 mg, 2.46 mmol) at room temperature. The reaction mixture was stirred at room temperature overnight. After the reaction was completed, the crude mixture was added to 1 N HCl and extracted with dichloromethane. The organic layer was dried over anhydrous Na_2_SO_4_ and concentrated under reduced pressure. The residue was purified by flash column chromatograph (0-30% ethyl acetate/hexane) to give the product as a pale yellow solid (230 mg, 61%); ^1^H NMR (400 MHz, DMSO-*d_6_*) *δ* 10.16 (brs, 1H, NH), 7.95 (t, *J* = 6.4 Hz, 1H, NH), 7.70 (d, *J* = 8.8 Hz, 4H), 7.12-7.03 (m, 6H), 6.94 (d, *J* = 7.6 Hz, 1H), 6.85 (d, *J* = 7.2 Hz, 1H), 3.83 (s, 3H), 3.80 (s, 2H), 3.78 (s, 3H); ^13^C NMR (100 MHz, DMSO-*d_6_)* *δ* 162.4, 162.1, 138.9, 138.0, 132.2, 131.2, 128.9, 128.6, 122.9, 118.8, 118.2, 114.3, 114.3, 55.6, 45.9; LC/MS (ESI) *m/z* 461.1 [M - H]^-^.

*N,N'-(4-Methyl-1,3-phenylene)bis(4-methoxybenzenesulfonamide) (****37b****).* Prepared as described in the experimental procedure of **23d** from 2-methyl-5-nitroaniline to obtain the title compound as a white solid (100 mg, 33% over 2 steps); ^1^H NMR (400 MHz, DMSO-*d_6_*) *δ* 10.02 (brs, 1H, NH), 9.36 (brs, 1H, NH), 7.61 (d, *J* = 8.8 Hz, 2H), 7.50 (d, *J* = 8.8 Hz, 2H), 7.05-7.00 (m, 5H), 6.92 (d, *J* = 8.4 Hz, 1H), 6.77 (dd, *J* = 8.4, 2.4 Hz, 1H), 3.81 (s, 3H), 3.78 (s, 3H), 1.82 (s, 3H); ^13^C NMR (100 MHz, DMSO-*d_6_*) *δ* 162.4, 162.3, 136.1, 135.5, 132.1, 131.00, 130.95, 128.9, 128.6, 128.5, 117.6, 117.3, 114.2, 114.2, 55.6, 55.6, 16.9; LC/MS (ESI) *m/z* 463.1 [M + H]^+^.

*Ethyl N-(3-((N-(2-ethoxy-2-oxoethyl)-4-methoxyphenyl)sulfonamido)benzyl)-N-((4-methoxyphenyl)-sulfonyl)glycinate (****38a****).* Prepared as described in the experimental procedure of **24a** from intermediate **37a** to obtain the title compound as a colorless oil (138 mg, quantitative); ^1^H NMR (400 MHz, CDCl_3_) *δ* 7.79 (d, *J* = 8.8 Hz, 2H), 7.60 (d, *J* = 8.8 Hz, 2H), 7.31-7.25 (m, 1H), 7.22-7.18 (m, 2H), 7.05 (s, 1H), 6.99 (d, *J* = 8.8 Hz, 2H), 6.93 (d, *J* = 8.8 Hz, 2H), 4.41 (s, 2H), 4.36 (s, 2H), 4.14 (q, *J* = 7.2 Hz, 2H), 4.02 (q, *J* = 7.2 Hz, 2H), 3.88 (s, 3H), 3.86 (s, 3H), 3.81 (s, 2H), 1.22 (t, *J* = 7.2 Hz, 3H), 1.16 (t, *J* = 7.2 Hz, 3H); ^13^C NMR (100 MHz, CDCl_3_) *δ* 168.6, 168.5, 163.2, 163.0, 140.4, 136.6, 131.2, 130.2, 129.8, 129.5, 129.5, 128.3, 128.2, 128.1, 114.2, 114.0, 61.4, 61.2, 55.6, 55.6, 52.4, 50.8, 46.8, 14.0, 14.0; LC/MS (ESI) *m/z* 635.2 [M + H]^+^.

*Diethyl 2,2'-((4-methyl-1,3-phenylene)bis(((4-methoxyphenyl)sulfonyl)azanediyl))diacetate (****38b****).* Prepared as described in the experimental procedure of **12a** from intermediate **26b** to obtain the title compound as a white solid (98 mg, 71%); ^1^H NMR (400 MHz, CD_3_OD) *δ* 7.55 (d, *J* = 8.8Hz, 2H), 7.54 (d, *J* = 8.8, 2H), 7.21 (d, *J* = 8.0 Hz, 1H), 7.11 (dd, *J* = 8.0, 2.4 Hz, 1H), 7.03 (d, *J* = 8.8 Hz, 2H), 6.99 (d, *J* = 8.8 Hz, 2H), 6.74 (d, *J* = 2.4 Hz, 1H), 4.19 (s, 2H), 4.16 (s, 2H), 4.15-4.07 (m, 4H), 3.89 (s, 3H), 3.86 (s, 3H), 2.32 (s, 3H), 1.29-1.15 (m, 6H); ^13^C NMR (100 MHz, CD_3_OD) *δ* 170.2, 169.9, 164.9, 164.9, 141.2, 140.5, 139.5, 132.6, 131.4, 131.4, 131.2, 131.0, 130.2, 129.9, 115.4, 115.3, 62.5, 62.5, 56.3, 56.3, 53.8, 53.5, 18.3, 14.4, 14.4; LC/MS (ESI) *m/z* 635.3 [M + H]^+^.

*2-(2-Methyl-5-nitrophenyl)acetonitrile (****39a****).* To a solution of **20d** (100 mg, 0.60 mmol) in dichloromethane (3 mL) were added triethylamine (73 mg, 0.72 mmol) and methanesulfonyl chloride (72 mg, 0.63 mmol) at 0 °C. The reaction mixture was stirred at 0 °C for 10 minutes. After the reaction was completed, the crude mixture was added to water and extracted with dichloromethane. The organic layer was dried over anhydrous Na_2_SO_4_ and concentrated under reduced pressure to give 2-methyl-5-nitrobenzyl methanesulfonate as a pale yellow oil (143 mg, 98%); ^1^H NMR (400 MHz, CDCl_3_) *δ* 8.23 (d, *J* = 2.4 Hz, 1H), 8.12 (dd, *J* = 8.4, 2.4 Hz, 1H), 7.39 (d, *J* = 8.4 Hz, 1H), 5.29 (s, 2H), 3.06 (s, 3H), 2.49 (s, 3H); ^13^C NMR (100 MHz, CDCl_3_) *δ* 146.6, 145.1, 133.4, 131.7, 124.34, 124.30, 68.9, 38.2, 19.2.

To a solution of 2-methyl-5-nitrobenzyl methanesulfonate (103 mg, 0.42 mmol) in acetonitrile (2.1 mL) was added potassium cyanide (82 mg, 1.26 mmol) at room temperature. The reaction mixture was stirred at room temperature overnight. After the reaction was completed, the crude mixture was added to water and extracted with dichloromethane. The organic layer was dried over anhydrous Na_2_SO_4_ and concentrated under reduced pressure. The residue was purified by flash column chromatograph (0-20% ethyl acetate/hexane) to give the product as a pale yellow solid (38 mg, 37%); ^1^H NMR (400 MHz, CDCl_3_) *δ* 8.22 (d, *J* = 2.4 Hz, 1H), 8.10 (dd, *J* = 8.4, 2.4 Hz, 1H), 7.40 (d, *J* = 8.4 Hz, 1H), 3.77 (s, 2H), 2.46 (s, 3H); ^13^C NMR (100 MHz, CDCl_3_) *δ* 146.7, 144.2, 131.7, 130.4, 123.8, 123.5, 116.4, 22.0, 19.7; LC/MS (ESI) *m/z* 175.1 [M - H]^-^.

*2-(2-Fluoro-5-nitrophenyl)acetonitrile (****39b****).* Prepared as described in the experimental procedure of **39a** from intermediate **20e** to obtain 2-fluoro-5-nitrobenzyl methanesulfonate as a pale yellow solid (637 mg, 49%); ^1^H NMR (400 MHz, CDCl_3_) *δ* 8.40-8.38 (m, 1H), 8.33-8.30 (m, 1H), 7.32-7.26 (m, 1H), 5.34 (s, 2H), 3.11 (s, 3H); ^13^C NMR (100 MHz, CDCl_3_) *δ* 164.2 (*J*_C,F_ = 259 Hz), 144.5, 127.1 (*J*_C,F_ = 10 Hz), 126.6 (*J*_C,F_ = 5 Hz), 123.1 (*J*_C,F_ = 17 Hz), 117.1 (*J*_C,F_ = 24 Hz), 63.4 (*J*_C,F_ = 4 Hz), 38.2.

Prepared as described in the experimental procedure of **39a** from 2-fluoro-5-nitrobenzyl methanesulfonate to obtain the title compound as a pale yellow solid (363 mg, 81%); ^1^H NMR (400 MHz, CDCl_3_) *δ* 8.40-8.35 (m, 1H), 8.30-8.24 (m, 1H), 7.33-7.26 (m, 1H), 3.87 (s, 2H); ^13^C NMR (100 MHz, CDCl_3_) *δ* 163.8 (*J*_C,F_ = 257 Hz), 144.6, 126.4 (*J*_C,F_ = 10 Hz), 126.0 (*J*_C,F_ = 4 Hz), 119.7 (*J*_C,F_ = 17 Hz), 117.0 (*J*_C,F_ = 23 Hz), 115.6, 17.8.

*2-(2-(Benzyloxy)-5-nitrophenyl)acetonitrile (****39c****).* Prepared as described in the experimental procedure of **39a** from intermediate **20f** to obtain 2-(benzyloxy)-5-nitrobenzyl methanesulfonate as a pale yellow solid (1.6 g, quantitative); ^1^H NMR (400 MHz, CDCl_3_) *δ* 8.31 (d, *J* = 2.8 Hz, 1H), 8.25 (dd, *J* = 8.8, 2.8 Hz, 1H), 7.43-7.42 (m, 2H), 7.06 (d, *J* = 8.8 Hz, 1H), 5.31 (s, 2H), 5.23 (s, 2H), 2.96 (s, 3H); ^13^C NMR (100 MHz, CDCl_3_) *δ* 161.3, 141.6, 135.0, 129.1, 128.9, 127.7, 126.8, 125.8, 123.6, 111.9, 71.4, 65.8, 37.8.

Prepared as described in the experimental procedure of **39a** from 2-(benzyloxy)-5-nitrobenzyl methanesulfonate to obtain the title compound as a pale yellow solid (1.1 g, 90%); ^1^H NMR (400 MHz, DMSO-*d_6_*) *δ* 8.32 (d, *J* = 2.8 Hz, 1H), 8.26 (dd, *J* = 9.2, 2.8 Hz, 1H), 7.54 (d, *J* = 7.2 Hz, 2H), 7.42 (d, *J* = 7.2, 7.2 Hz, 2H), 7.37-7.34 (m, 2H), 4.27 (s, 2H), 4.27 (s, 2H); ^13^C NMR (100 MHz, DMSO-*d_6_*) *δ* 161.0, 140.6, 135.7, 128.5, 128.2, 127.5, 125.8, 125.2, 121.0, 118.0, 112.6, 70.6, 18.2.

*3-(2-Aminoethyl)-4-methylaniline (****40****).* To a solution of **39a** (430 mg, 2.44 mmol) in tetrahydrofuran (5 mL) was added borane tetrahydrofuran complex solution (1.0 M in THF, 4.9 mL, 4.88 mmol) at room temperature. The reaction mixture was stirred at 60 °C for 1 hour. After the reaction was completed, the crude mixture was added to methanol and water and then concentrated under reduced pressure. The aqueous solution was acidified with 6 N HCl until the pH reached around one and stirred at 70 °C for 1 hour. After cooling to room temperature, the solution was basified with sat. NaHCO_3_ until the pH reached around 10, and then it was extracted with dichloromethane. The organic layer was dried over anhydrous Na_2_SO_4_ and concentrated under reduced pressure to give 2-(2-methyl-5-nitrophenyl)ethan-1-amine as a pale yellow oil (440 mg, quantitative); ^1^H NMR (400 MHz, CDCl_3_) *δ* 8.02 (d, *J* = 2.4 Hz, 1H), 7.97 (dd, *J* = 8.4, 2.4 Hz, 1H), 7.29 (d, *J* = 8.4 Hz, 1H), 3.00 (t, *J* = 7.2 Hz, 2H), 2.84 (t, *J* = 7.2 Hz, 2H), 2.42 (s, 3H); ^13^C NMR (100 MHz, CDCl_3_) *δ* 146.6, 144.5, 140.0, 131.1, 124.1, 121.5, 42.1, 37.3, 19.9; LC/MS (ESI) *m/z* 181.1 [M + H]^+^.

Prepared as described in the experimental procedure of **31** from 2-(2-methyl-5-nitrophenyl)-ethan-1-amine to obtain the title compound as a pale yellow oil (394 mg, 99%); ^1^H NMR (400 MHz, CDCl_3_) *δ* 6.91 (d, *J* = 7.6 Hz, 1H), 6.48-6.45 (m, 2H), 2.87 (t, *J* = 7.2 Hz, 2H), 2.65 (t, *J* = 7.2 Hz, 2H), 2.18 (s, 3H); ^13^C NMR (100 MHz, CDCl_3_) *δ* 144.4, 138.6, 131.0, 126.0, 116.5, 113.3, 42.2, 37.5, 18.4.

*N-(2-Fluoro-5-nitrophenethyl)-4-methylbenzenesulfonamide (****41a****).* To a solution of **39b** (804 mg, 4.46 mmol) in tetrahydrofuran (8 mL) was added borane tetrahydrofuran complex solution (1.0 M in THF, 8.9 mL, 8.93 mmol) at room temperature. The reaction mixture was stirred at 60 °C for 1 hour. After the reaction was completed, the crude mixture was added to methanol and water and then concentrated under reduced pressure. The aqueous solution was acidified with 6 N HCl until the pH reached around one and stirred at 80 °C for 30 minutes. After cooling to room temperature, the solution was basified with sat. NaHCO_3_ until the pH reached around 10, and then it was extracted with dichloromethane. The organic layer was dried over anhydrous Na_2_SO_4_ and concentrated under reduced pressure to give the product, 2-(2-fluoro-5-nitrophenyl)ethan-1-amine, as a pale yellow oil. The resulting crude compound was used for the next reaction without further purification.

To a solution of 2-(2-fluoro-5-nitrophenyl)ethan-1-amine (300 mg, 1.63 mmol) in dichloromethane (8 mL) were added pyridine (193 mg, 2.44 mmol) and 4-methylbenzenesulfonyl chloride (466 mg, 2.44 mmol) at room temperature. The reaction mixture was stirred at room temperature overnight. After the reaction was completed, the crude mixture was added to 1N HCl and extracted with dichloromethane. The organic layer was dried over anhydrous Na_2_SO_4_ and concentrated under reduced pressure. The residue was purified by flash column chromatograph (0-35% ethyl acetate/hexane) to give the product as a pale yellow solid (227 mg, 28% over two steps); ^1^H NMR (400 MHz, CDCl_3_) *δ* 8.07-8.03 (m, 1H), 7.95-7.93 (m, 1H), 7.64 (d, *J* = 8.0 Hz, 2H), 7.23 (d, *J* = 8.0 Hz, 2H), 7.10-7.06 (m, 1H), 4.78 (t, *J* = 6.4 Hz, 1H, NH), 3.26-3.21 (m, 2H), 2.84 (t, *J* = 6.8 Hz, 2H), 2.37 (s, 3H); ^13^C NMR (100 MHz, CDCl_3_) *δ* 164.8 (*J*_C,F_ = 255 Hz), 144.3 (*J*_C,F_ = 4 Hz), 143.9, 137.0, 130.2, 129.9, 127.2 (*J*_C,F_ = 17 Hz), 127.1, 124.7 (*J*_C,F_ = 10 Hz), 116.5 (*J*_C,F_ = 25 Hz), 42.6, 29.8, 21.6; LC/MS (ESI) *m/z* 339.1 [M + H]^+^.

*N-(2-(Benzyloxy)-5-nitrophenethyl)-4-methoxybenzenesulfonamide (****41b****).* Prepared as described in the experimental procedure of **41a** from intermediate **39c** to obtain 2-(2-(benzyloxy)-5-nitrophenyl)ethan-1-amine as a pale yellow oil (1.1 g, quantitative); ^1^H NMR (400 MHz, CDCl_3_) *δ* 8.10-8.07 (m, 2H), 7.41-7.40 (m, 5H), 6.95 (d, *J* = 8.8 Hz, 1H), 5.18 (s, 2H), 2.99 (t, *J* = 6.8 Hz, 2H), 2.86 (t, *J* = 6.8 Hz, 2H); ^13^C NMR (100 MHz, CDCl_3_) *δ* 161.8, 141.4, 135.8, 129.8, 128.9, 128.5, 127.3, 126.1, 124.1, 111.3, 70.8, 41.6, 34.6; LC/MS (ESI) *m/z* 273.2 [M + H]^+^.

Prepared as described in the experimental procedure of **41a** from 2-(2-(benzyloxy)-5-nitrophenyl)ethan-1-amine to obtain the title compound as a pale yellow solid (258 mg, 29%); ^1^H NMR (400 MHz, CDCl_3_) *δ* 8.06 (dd, *J* = 8.8, 2.8 Hz, 1H), 7.87 (d, *J* = 2.8 Hz, 1H), 7.65 (d, *J* = 8.8 Hz, 2H), 7.44-7.37 (m, 5H), 6.92 (d, *J* = 8.8 Hz, 1H), 6.87 (d, *J* = 8.8 Hz, 2H), 5.14 (s, 2H), 4.72 (t, *J* = 6.4 Hz, 1H, NH), 3.84 (s, 3H), 3.28-3.23 (m, 2H), 2.83 (t, *J* = 6.8 Hz, 2H); ^13^C NMR (100 MHz, CDCl_3_) *δ* 162.9, 161.7, 141.3, 135.5, 131.6, 129.1, 128.9, 128.6, 128.0, 127.5, 126.3, 124.5, 114.3, 111.4, 70.9, 55.6, 42.4, 30.8; LC/MS (ESI) *m/z* 443.1 [M + H]^+^.

*N-(2-(Benzyloxy)-5-nitrophenethyl)-4-methylbenzenesulfonamide (****41c****).* To a solution of 2-(2-(benzyloxy)-5-nitrophenyl)ethan-1-amine (539 mg, 1.98 mmol) in dichloromethane (10 mL) were added pyridine (235 mg, 2.97 mmol) and 4-methylbenzenesulfonyl chloride (566 mg, 2.97 mmol) at room temperature. The reaction mixture was stirred at room temperature overnight. After the reaction was completed, the crude mixture was added to 1 N HCl and extracted with dichloromethane. The organic layer was dried over anhydrous Na_2_SO_4_ and concentrated under reduced pressure. The residue was purified by flash column chromatograph (0-40% ethyl acetate/hexane) to give the product as a pale yellow solid (256 mg, 30%); ^1^H NMR (400 MHz, DMSO-*d_6_*) *δ* 8.10 (d, *J* = 8.8 Hz, 1H), 7.99 (s, 1H), 7.65 (t, *J* = 5.2 Hz, 1H, NH), 7.56 (d, *J* = 8.0 Hz, 2H), 7.44-7.36 (m, 5H), 7.28 (d, *J* = 8.0 Hz, 2H), 7.20 (d, *J* = 8.8 Hz, 1H), 5.24 (s, 2H), 3.34 (s, 2H), 3.07-3.02 (m, 2H), 2.77 (t, *J* = 6.8 Hz, 2H), 2.35 (s, 3H); ^13^C NMR (100 MHz, DMSO-*d_6_*) *δ* 161.5, 142.4, 140.4, 137.6, 136.0, 129.5, 128.5, 128.1, 127.4, 126.3, 125.9, 124.2, 112.1, 70.1, 41.5, 30.0, 20.9; LC/MS (ESI) *m/z* 427.2 [M + H]^+^.

*N-(2-(Benzyloxy)-5-nitrophenethyl)-[1,1'-biphenyl]-4-sulfonamide (****41d****).* Prepared as described in the experimental procedure of **41c** from 2-(2-(benzyloxy)-5-nitrophenyl)ethan-1-amine and 4-biphenylsulfonyl chloride to obtain the title compound as a pale yellow solid (229 mg, 25%); ^1^H NMR (400 MHz, DMSO-*d_6_*) *δ* 8.07 (dd, *J* = 8.8, 2.4 Hz, 1H), 8.03 (d, *J* = 2.4 Hz, 1H), 7.80-7.73 (m, 4H), 7.70 (d, *J* = 7.2 Hz, 2H), 7.53-7.49 (m, 2H), 7.45-7.33 (m, 6H), 7.19 (d, *J* = 8.8 Hz, 1H), 5.24 (s, 2H), 3.11-3.10 (m, 2H), 2.82-2.79 (m, 2H); ^13^C NMR (100 MHz, DMSO-*d_6_*) *δ* 161.6, 143.8, 140.5, 139.2, 138.6, 136.1, 129.1, 128.6, 128.5, 128.1, 128.0, 127.50, 127.45, 127.3, 127.0, 126.0, 124.2, 112.1, 70.1, 41.6, 30.1; LC/MS (ESI) *m/z* 489.3 [M + H]^+^.

*4-Methoxy-N-(5-((4-methoxyphenyl)sulfonamido)-2-methylphenethyl)benzenesulfonamide (****42a****).* Prepared as described in the experimental procedure of **37a** from intermediate **40** and 4-methoxylbenzenesulfonyl chloride to obtain the title compound as a white solid (41 mg, 18%); ^1^H NMR (400 MHz, CDCl_3_) *δ* 7.73-7.69 (m, 4H), 7.30 (brs, 1H, NH), 6.93-6.90 (m, 3H), 6.87-6.84 (m, 3H), 6.81 (s, 1H), 4.98 (t, *J* = 6.0 Hz, 1H, NH), 3.83 (s, 3H), 3.78 (s, 3H), 3.08-3.03 (m, 2H), 2.64 (t, *J* = 7.2 Hz, 2H), 2.08 (s, 3H); ^13^C NMR (100 MHz, CDCl_3_) *δ* 163.2, 163.0, 137.3, 134.8, 133.4, 131.4, 131.3, 130.6, 129.6, 129.3, 122.8, 120.2, 114.4, 114.3, 55.8, 55.7, 42.8, 33.2, 18.7; LC/MS (ESI) *m/z* 491.1 [M + H]^+^.

*4-Methyl-N-(5-((4-methylphenyl)sulfonamido)-2-methylphenethyl)benzenesulfonamide (****42b****).* Prepared as described in the experimental procedure of **37a** from intermediate **40** and 4-methylbenzenesulfonyl chloride to obtain the title compound as a pale yellow solid (14 mg, 13%); ^1^H NMR (400 MHz, CDCl_3_) *δ* 7.68 (d, *J* = 8.0 Hz, 2H), 7.64 (d, *J* = 8.0 Hz, 2H), 7.27 (d, *J* = 8.0 Hz, 2H), 7.20 (d, *J* = 8.0 Hz, 2H), 6.99 (brs, 1H, NH), 6.94 (d, *J* = 8.4 Hz, 1H), 6.84 (dd, *J* = 8.4, 2.0 Hz, 1H), 6.78 (d, *J* = 2.0 Hz, 1H), 4.78 (t, *J* = 6.0 Hz, 1H, NH), 3.11-3.06 (m, 2H), 2.66 (t, *J* = 6.8 Hz, 2H), 2.41 (s, 3H), 2.35 (s, 3H), 2.11 (s, 3H); ^13^C NMR (100 MHz, CDCl_3_) *δ* 144.0. 143.6. 137.2. 137.1. 136.3. 134.7. 133.6. 131.4. 130.0. 129.8. 127.4. 127.2. 123.0. 120.4. 42.9. 33.3. 21.6. 18.8; LC/MS (ESI) *m/z* 459.2 [M + H]^+^.

*4-Bromo-N-(5-((4-bromophenyl)sulfonamido)-2-methylphenethyl)benzenesulfonamide (****42c****).* Prepared as described in the experimental procedure of **37a** from intermediate **40** and 4-bromobenzenesulfonyl chloride to obtain the title compound as a pale yellow solid (509 mg, 32%); ^1^H NMR (400 MHz, CDCl_3_) *δ* 7.66-7.54 (m, 8H), 7.12 (brs, 1H, NH), 6.97 (d, *J* = 8.0 Hz, 1H), 6.87 (d, *J* = 2.4 Hz, 1H), 6.83 (dd, *J* = 8.0, 2.4 Hz, 1H), 4.98 (t, *J* = 6.0 Hz, 1H, NH), 3.15-3.10 (m, 2H), 2.71 (t, *J* = 6.8 Hz, 2H), 2.14 (s, 3H); ^13^C NMR (100 MHz, CDCl_3_) *δ* 139.0, 138.1, 137.3, 134.2, 132.6, 132.5, 131.6, 129.0, 128.7, 128.2, 127.8, 123.5, 120.7, 42.8, 33.4, 18.8; LC/MS (ESI) *m/z* 587.0 [M(2^79^Br,) + H]^+^, 589.0 [M(^79^Br, ^81^Br) + H]^+^, 590.9 [M(2^81^Br) + H]^+^.

*N-(4-Fluoro-3-(2-((4-methylphenyl)sulfonamido)ethyl)phenyl)-4-methylbenzenesulfonamide (****42d****).* Prepared as described in the experimental procedure of **23a** from intermediate **41a** to obtain the title compound as a pale yellow oil (189 mg, 65% over 2 steps); ^1^H NMR (400 MHz, CDCl_3_) *δ* 7.66 (d, *J* = 8.4 Hz, 2H), 7.62 (d, *J* = 8.4 Hz, 2H), 7.26 (d, *J* = 8.0 Hz, 2H), 7.22 (d, *J* = 8.0 Hz, 2H), 6.94-6.90 (m, 1H), 6.88-6.85 (m, 1H), 6.83-6.79 (m, 1H), 3.12 (t, *J* = 6.4 Hz, 2H), 2.68 (t, *J* = 6.4 Hz, 2H), 2.41 (s, 3H), 2.36 (s, 3H); ^13^C NMR (100 MHz, CDCl_3_) *δ* 158.9 (*J*_C,F_ = 242 Hz), 144.2, 143.7, 136.9, 135.9, 132.7 (*J*_C,F_ = 3 Hz), 129.9, 129.9, 129.8 (*J*_C,F_ = 6 Hz), 127.4, 127.1, 126.0 (*J*_C,F_ = 17 Hz), 125.4 (*J*_C,F_ = 5 Hz), 116.2 (*J*_C,F_ = 23 Hz), 42.8, 29.5, 21.7; LC/MS (ESI) *m/z* 463.2 [M + H]^+^.

*N-(4-(Benzyloxy)-3-(2-((4-methoxyphenyl)sulfonamido)ethyl)phenyl)-4-methoxybenzenesulfon-amide* ***(42e****).* Prepared as described in the experimental procedure of **23a** from intermediate **41b** to obtain the title compound as a white solid (282 mg, 94% over two steps); ^1^H NMR (400 MHz, DMSO-*d_6_*) *δ* 9.72 (brs, 1H, NH), 7.64 (d, *J* = 8.8 Hz, 2H), 7.61 (d, *J* = 8.8 Hz, 2H), 7.51 (t, *J* = 6.0 Hz, 1H, NH), 7.36-7.30 (m, 5H), 7.04 (d, *J* = 8.8 Hz, 2H), 7.01 (d, *J* = 8.8 Hz, 2H), 6.88-6.83 (m, 3H), 3.81 (s, 2H), 3.78 (s, 3H), 2.84-2.79 (m, 2H), 2.56 (t, *J* = 7.6 Hz, 2H); ^13^C NMR (100 MHz, DMSO-*d_6_*) *δ* 162.2, 162.0, 153.2, 137.0, 132.0, 131.2, 130.4, 128.8, 128.6, 128.3, 127.7, 127.3, 124.0, 120.9, 114.19, 114.15, 112.4, 69.3, 55.5, 42.3, 30.4; LC/MS (ESI) *m/z* 583.2 [M + H]^+^.

*N-(4-(Benzyloxy)-3-(2-((4-methylphenyl)sulfonamido)ethyl)phenyl)-4-methylbenzenesulfonamide* ***(42f****).* Prepared as described in the experimental procedure of **23a** from intermediate **41c** to obtain the title compound as a white solid (175 mg, 57%); ^1^H NMR (400 MHz, DMSO-*d_6_*) *δ* 9.78 (brs, 1H, NH), 7.59 (brs, 1H, NH), 7.59 (d, *J* = 8.0 Hz, 2H), 7.59 (d, *J* = 8.0 Hz, 2H), 7.36-7.28 (m, 9H), 6.87-6.80 (m, 3H), 4.96 (s, 2H), 2.83-2.78 (m, 2H), 2.57-2.50 (m, 2H), 2.36 (s, 3H), 2.31 (s, 3H); ^13^C NMR (100 MHz, DMSO-*d_6_*) *δ* 153.2, 142.9, 142.4, 137.5, 137.0, 136.6, 130.2, 129.5, 129.4, 128.3, 127.7, 127.3, 127.2, 126.7, 126.4, 124.0, 121.0, 112.4, 69.3, 42.3, 30.4, 20.9, 20.9; LC/MS (ESI) *m/z* 551.2 [M + H]^+^.

*N-(5-([1,1'-Biphenyl]-4-sulfonamido)-2-(benzyloxy)phenethyl)-[1,1'-biphenyl]-4-sulfonamide (****42g****).* Prepared as described in the experimental procedure of **23a** from intermediate **41d** to obtain the title compound as a white solid (201 mg, 67% over two steps); ^1^H NMR (400 MHz, CDCl_3_) *δ* 7.81 (d, *J* = 7.2 Hz, 2H), 7.73 (d, *J* = 8.4 Hz, 2H), 7.63 (d, *J* = 8.0 Hz, 2H), 7.56-7.54 (m, 4H), 7.48-7.38 (m, 9H), 7.35-7.31 (m, 4H), 6.96 (d, *J* = 8.4 Hz, 1H), 6.92 (s, 1H), 6.71 (d, *J* = 8.8 Hz, 1H), 4.90 (s, 2H), 3.21 (dd, *J* = 12.4, 6.4 Hz, 1H), 7.59 (t, *J* = 6.4 Hz, 1H); ^13^C NMR (100 MHz, CDCl_3_) *δ* 155.0, 145.8, 145.4, 139.4, 139.2, 138.6, 137.7, 136.6, 129.4, 129.2, 129.1, 128.8, 128.7, 128.6, 128.6, 128.3, 128.0, 127.7, 127.7, 127.6, 127.6, 127.4, 127.4, 126.5, 123.4, 112.4, 70.4, 42.9, 30.7; LC/MS (ESI) *m/z* 675.3 [M + H]^+^.

*Ethyl N-(5-((N-(2-ethoxy-2-oxoethyl)-4-methoxyphenyl)sulfonamido)-2-methylphenethyl)-N-((4-methoxyphenyl)sulfonyl)glycinate (****43a****).* Prepared as described in the experimental procedure of **24a** from intermediate **42a** to obtain the title compound as a colorless oil (32 mg, 60%); ^1^H NMR (400 MHz, CDCl_3_) *δ* 7.76 (d, *J* = 8.8Hz, 2H), 7.60 (d, *J* = 8.8, 2H), 7.02 (d, *J* = 8.0 Hz, 1H), 6.96 (d, *J* = 8.8 Hz, 2H), 6.93-6.90 (m, 4H), 4.33 (s, 2H), 4.15-4.07 (m, 4H), 3.99 (s, 2H), 3.86 (s, 3H), 3.85 (s, 3H), 3.30 (t, *J* = 8.0 Hz, 2H), 2.80 (t, *J* = 8.0 Hz, 2H), 2.24 (s, 3H), 1.21 (t, *J* = 7.2 Hz, 6H); ^13^C NMR (100 MHz, CDCl_3_) *δ* 169.1, 169.0, 163.2, 163.1, 138.0, 137.6, 136.7, 131.5, 131.3, 130.8, 130.1, 129.9, 129.7, 127.0, 114.3, 114.0, 61.5, 55.7, 52.8, 48.8, 48.8, 32.7, 19.0, 14.2; LC/MS (ESI) *m/z* 663.3 [M + H]^+^.

*Ethyl N-(5-((N-(2-ethoxy-2-oxoethyl)-4-methylphenyl)sulfonamido)-2-methylphenethyl)-N-((4-methyl-phenyl)sulfonyl)glycinate (****43b****).* Prepared as described in the experimental procedure of **24a** from intermediate **42b** to obtain the title compound as a colorless oil (18 mg, 94%); ^1^H NMR (400 MHz, CDCl_3_) *δ* 7.70 (d, *J* = 8.4 Hz, 2H), 7.53 (d, *J* = 8.4, 2H), 7.28 (d, *J* = 8.4 Hz, 2H), 7.24 (d, *J* = 8.0, 2H), 7.03 (d, *J* = 8.0 Hz, 1H), 6.93 (d, *J* = 2.0 Hz, 1H), 6.89 (dd, *J* = 8.0, 2.0 Hz, 1H), 4.33 (s, 2H), 4.15-4.05 (m, 4H), 3.99 (s, 2H), 3.31 (t, *J* = 8.0 Hz, 2H), 2.81 (t, *J* = 8.0 Hz, 2H), 2.41 (s, 3H), 2.40 (s, 3H), 2.25 (s, 3H), 1.22-1.18 (m, 6H); ^13^C NMR (100 MHz, CDCl_3_) *δ* 169.0, 168.9, 143.8, 143.7, 137.9, 137.5, 136.8, 136.1, 131.2, 129.9, 129.7, 129.5, 127.9, 127.5, 127.0, 61.5, 52.8, 48.8, 32.7, 21.7, 19.0, 14.2; LC/MS (ESI) *m/z* 631.2 [M + H]^+^.

*Ethyl N-(3-(2-((4-bromo-N-(2-ethoxy-2-oxoethyl)phenyl)sulfonamido)ethyl)-4-methylphenyl)-N-((4-bromophenyl)sulfonyl)glycinate (****43c****).* Prepared as described in the experimental procedure of **24a** from intermediate **42c** to obtain the title compound as a colorless oil (355 mg, 56%); ^1^H NMR (400 MHz, CDCl_3_) *δ* 7.70 (d, *J* = 8.4 Hz, 2H), 7.64-7.53 (m, 6H), 7.05 (d, *J* = 8.0 Hz, 1H), 7.02 (d, *J* = 2.0 Hz, 1H), 6.89 (dd, *J* = 8.0, 2.0 Hz, 1H), 4.36 (s, 2H), 4.16-4.07 (m, 4H), 4.01 (s, 2H), 3.34 (t, *J* = 7.6 Hz, 2H), 2.85 (t, *J* = 7.6 Hz, 2H), 2.27 (s, 3H), 1.27-1.19 (m, 6H); ^13^C NMR (100 MHz, CDCl_3_) *δ* 168.8, 168.7, 139.0, 138.4, 137.6, 137.2, 132.4, 132.1, 131.5, 130.2, 129.5, 129.1, 128.0, 127.9, 126.9, 61.7, 53.0, 48.7, 48.7, 32.6, 19.0, 14.2; LC/MS (ESI) *m/z* 759.0 [M(2^79^Br,) + H]^+^, 761.0 [M(^79^Br, ^81^Br) + H]^+^, 763.0 [M(2^81^Br) + H]^+^.

*Ethyl N-(5-((N-(2-ethoxy-2-oxoethyl)-4-methylphenyl)sulfonamido)-2-fluorophenethyl)-N-tosyl-glycinate (****43d****).* Prepared as described in the experimental procedure of **24a** from intermediate **42d** to obtain the title compound as a colorless oil (169 mg, 65%); ^1^H NMR (400 MHz, CDCl_3_) *δ* 7.64 (d, *J* = 8.0 Hz, 2H), 7.50 (d, *J* = 8.4 Hz, 2H), 7.25-7.22 (m, 4H), 7.03-6.98 (m, 2H), 6.88-6.84 (m, 1H), 4.31 (s, 2H), 4.10 (q, *J* = 7.2 Hz, 2H), 4.04 (q, *J* = 7.2 Hz, 2H), 3.94 (s, 2H), 3.37 (t, *J* = 7.6 Hz, 2H), 2.77 (t, *J* = 7.6 Hz, 2H), 2.38 (s, 3H), 2.37 (s, 3H), 1.23-1.13 (m, 6H); ^13^C NMR (100 MHz, CDCl_3_) *δ* 168.9, 168.7, 160.6 (*J*_C,F_ = 246 Hz), 144.1, 143.6, 136.7, 135.75 (*J*_C,F_ = 3 Hz), 135.71, 132.0 (*J*_C,F_ = 5 Hz), 129.7, 129.6, 129.4 (*J*_C,F_ = 8 Hz), 127.8, 127.4, 126.2 (*J*_C,F_ = 17 Hz), 116.1 (*J*_C,F_ = 24 Hz), 61.6, 61.4, 52.8, 50.7, 48.6, 28.6, 21.6, 21.6, 14.1, 14.1; LC/MS (ESI) *m/z* 635.3 [M + H]^+^.

*Ethyl N-(4-(benzyloxy)-3-(2-((N-(2-ethoxy-2-oxoethyl)-4-methoxyphenyl)sulfonamido)ethyl)-phenyl)-N-((4-methoxyphenyl)sulfonyl)glycinate (****43e****).* Prepared as described in the experimental procedure of **24a** from intermediate **42e** to obtain the title compound as a colorless oil (311 mg, 92%); ^1^H NMR (400 MHz, CDCl_3_) *δ* 7.66 (d, *J* = 8.4 Hz, 2H), 7.61 (d, *J* = 8.4 Hz, 2H), 7.39-7.30 (m, 5H), 7.07 (dd, *J* = 8.4, 2.0 Hz, 1H), 6.93 (d, *J* = 8.4 Hz, 2H), 6.89-6.87 (m, 3H), 6.76 (d, *J* = 8.4 Hz, 1H), 4.98 (s, 2H), 4.33 (s, 2H), 4.14 (q, *J* = 7.2 Hz, 2H), 4.02 (q, *J* = 7.2 Hz, 2H), 3.88 (s, 2H), 3.85 (s, 3H), 3.83 (s, 3H), 3.38 (t, *J* = 7.6 Hz, 2H), 2.76 (t, *J* = 7.6 Hz, 2H), 1.22 (t, *J* = 7.2 Hz, 3H), 1.15 (t, *J* = 7.2 Hz, 3H); ^13^C NMR (100 MHz, CDCl_3_) *δ* 169.1, 169.0, 163.2, 162.9, 156.5, 136.6, 132.6, 131.7, 131.2, 130.7, 130.1, 129.5, 129.3, 128.7, 128.2, 127.7, 127.4, 114.1, 114.0, 111.9, 70.3, 61.4, 61.3, 55.7, 55.6, 53.0, 48.4, 48.0, 30.0, 14.2, 14.1; LC/MS (ESI) *m/z* 755.3 [M + H]^+^.

*Ethyl N-(4-(benzyloxy)-3-(2-((N-(2-ethoxy-2-oxoethyl)-4-methylphenyl) sulfonamido)ethyl)phenyl)-N-((4-methylphenyl)sulfonyl)glycinate (****43f****).* Prepared as described in the experimental procedure of **24a** from intermediate **42f** to obtain the title compound as a colorless oil (41 mg, 83%); ^1^H NMR (400 MHz, CDCl_3_) *δ* 7.61 (d, *J* = 8.4 Hz, 2H), 7.56 (d, *J* = 8.4 Hz, 2H), 7.39-7.30 (m, 5H), 7.26 (d, *J* = 8.4 Hz, 2H), 7.21 (d, *J* = 8.4 Hz, 2H), 7.06 (dd, *J* = 8.8, 2.4 Hz, 1H), 6.88 (d, *J* = 2.4 Hz, 1H), 6.76 (d, *J* = 8.8 Hz, 1H), 4.98 (s, 2H), 4.33 (s, 2H), 4.12 (q, *J* = 7.2 Hz, 2H), 4.00 (q, *J* = 7.2 Hz, 2H), 3.88 (s, 2H), 3.40 (t, *J* = 7.6 Hz, 2H), 2.77 (t, *J* = 7.6 Hz, 2H), 2.41 (s, 3H), 2.38 (s, 3H), 1.21 (t, *J* = 7.2 Hz, 3H), 1.14 (t, *J* = 7.2 Hz, 3H); ^13^C NMR (100 MHz, CDCl_3_) *δ* 169.0, 168.9, 156.5, 143.8, 143.3, 137.1, 136.6, 136.2, 132.5, 131.3, 129.5, 129.5, 129.2, 128.7, 128.2, 127.9, 127.6, 127.4, 111.9, 70.3, 61.5, 61.2, 53.0, 48.4, 48.0, 30.0, 21.6, 21.6, 14.2, 14.1; LC/MS (ESI) *m/z* 723.2 [M + H]^+^.

*Ethyl N-([1,1'-biphenyl]-4-ylsulfonyl)-N-(4-(benzyloxy)-3-(2-(N-(2-ethoxy-2-oxoethyl)-[1,1'-bi-phenyl]-4-sulfonamido)ethyl)phenyl)glycinate (****43g****).* Prepared as described in the experimental procedure of **24a** from intermediate **42g** to obtain the title compound as a white solid (152 mg, 60%); ^1^H NMR (400 MHz, CDCl_3_) *δ* 7.79-7.76 (m, 4H), 7.69 (d, *J* = 8.8 Hz, 2H), 7.64-7.56 (m, 6H), 7.48 (d, *J* = 7.2 Hz, 2H), 7.46 (d, *J* = 7.6 Hz, 2H), 7.42-7.38 (m, 6H), 7.36-7.32 (m, 1H), 7.12 (dd, *J* = 8.8, 2.4 Hz, 1H), 7.05 (d, *J* = 2.4 Hz, 1H), 6.78 (d, *J* = 8.8 Hz, 1H), 4.99 (s, 2H), 4.41 (s, 2H), 4.16 (q, *J* = 7.2 Hz, 2H), 4.00 (q, *J* = 7.2 Hz, 2H), 3.95 (s, 2H), 3.51 (t, *J* = 7.6 Hz, 2H), 2.86 (t, *J* = 7.6 Hz, 2H), 1.23 (t, *J* = 7.2 Hz, 3H), 1.13 (t, *J* = 7.2 Hz, 3H); ^13^C NMR (100 MHz, CDCl_3_) *δ* 168.9, 156.6, 145.7, 145.3, 139.4, 139.3, 138.6, 137.8, 136.5, 132.4, 131.4, 129.2, 129.1, 128.7, 128.53, 128.46, 128.4, 128.2, 127.9, 127.7, 127.5, 127.4, 127.4, 127.3, 111.9, 70.3, 61.5, 61.3, 53.1, 48.4, 48.0, 30.0, 14.2, 14.0; LC/MS (ESI) *m/z* 847.4 [M + H]^+^.

*Ethyl N-(5-((N-(2-ethoxy-2-oxoethyl)-4-methoxyphenyl)sulfonamido)-2-hydroxybenzyl)-N-((4-methoxy-phenyl)sulfonyl)glycinate (****44a****).* Prepared as described in the experimental procedure of **25** from intermediate **43e** to obtain the title compound as a colorless oil (96 mg, 45%); ^1^H NMR (400 MHz, CDCl_3_) *δ* 7.73 (d, *J* = 8.8 Hz, 2H), 7.58 (d, *J* = 8.8 Hz, 2H), 6.93 (d, *J* = 8.8 Hz, 2H), 6.91 (d, *J* = 8.8 Hz, 2H), 6.89-6.86 (m, 2H), 6.66 (d, *J* = 8.4 Hz, 1H), 4.30 (s, 2H), 4.15-4.06 (m, 4H), 3.97 (s, 2H), 3.84 (s, 6H), 3.34 (t, *J* = 7.6 Hz, 2H), 2.78 (t, *J* = 7.6 Hz, 2H), 1.22-1.17 (m, 6H); ^13^C NMR (100 MHz, CDCl_3_) *δ* 169.5, 169.2, 163.2, 163.1, 154.7, 131.9, 131.5, 131.0, 130.6, 130.1, 129.7, 129.1, 125.2, 116.4, 114.3, 114.0, 61.6, 61.5, 55.7, 49.2, 48.6, 31.7, 30.2, 22.7, 14.2, 14.1; LC/MS (ESI) *m/z* 665.3 [M + H]^+^.

*Ethyl N-(5-((N-(2-ethoxy-2-oxoethyl)-4-methylphenyl)sulfonamido)-2-hydroxybenzyl)-N-((4-methyl-phenyl)sulfonyl)glycinate (****44b****).* Prepared as described in the experimental procedure of **25** from intermediate **43f** to obtain the title compound as a colorless oil (122 mg, 77%); ^1^H NMR (400 MHz, CDCl_3_) *δ* 7.70 (d, *J* = 8.4 Hz, 2H), 7.54 (d, *J* = 8.4 Hz, 2H), 7.28 (d, *J* = 8.4 Hz, 2H), 7.25 (d, *J* = 8.4 Hz, 2H), 6.90-6.86 (m, 2H), 6.68 (d, *J* = 8.4 Hz, 1H), 6.51 (brs, 1H, OH), 4.32 (s, 2H), 4.16-4.07 (m, 4H), 3.99 (s, 2H), 3.35 (t, *J* = 7.6 Hz, 2H), 2.83 (t, *J* = 7.6 Hz, 2H), 2.41 (s, 6H), 1.24-1.18 (m, 6H); ^13^C NMR (100 MHz, CDCl_3_) *δ* 169.4, 169.1, 154.7, 143.8, 136.4, 136.1, 132.0, 131.7, 129.8, 129.5, 129.1, 127.9, 127.5, 125.0, 116.5, 61.7, 61.5, 53.1, 49.3, 48.8, 30.4, 21.7, 21.6, 14.2, 14.1; LC/MS (ESI) *m/z* 633.3 [M + H]^+^.

*Ethyl N-([1,1'-biphenyl]-4-ylsulfonyl)-N-(5-(N-(2-ethoxy-2-oxoethyl)-[1,1'-biphenyl]-4-sulfonamido)-2-hydroxyphenethyl)glycinate (****44c****).* Prepared as described in the experimental procedure of **25** from intermediate **43g** to obtain the title compound as a white solid (22 mg, 16%); ^1^H NMR (400 MHz, CDCl_3_) *δ* 7.86 (d, *J* = 8.4 Hz, 2H), 7.74 (d, *J* = 8.0 Hz, 2H), 7.68-7.65 (m, 3H), 7.61 (d, *J* = 8.0 Hz, 2H), 7.58 (d, *J* = 8.0 Hz, 2H), 7.48-7.44 (m, 4H), 7.43-7.37 (m, 3H), 7.04 (d, *J* = 2.4 Hz, 1H), 6.92 (dd, *J* = 8.4, 2.4 Hz, 2H), 6.70 (d, *J* = 8.4 Hz, 1H), 4.37 (s, 2H), 4.15 (q, *J* = 7.2 Hz, 1H), 4.08 (q, *J* = 7.2 Hz, 1H), 4.03 (s, 2H), 3.41 (t, *J* = 7.2 Hz, 2H), 2.91 (t, *J* = 7.2 Hz, 2H), 1.22 (t, *J* = 7.0 Hz, 3H), 1.18 (t, *J* = 7.0 Hz, 3H); ^13^C NMR (100 MHz, CDCl_3_) *δ* 169.3, 169.1, 154.7, 145.9, 145.8, 139.4, 137.9, 137.8, 132.2, 132.0, 129.2, 129.1, 128.8, 128.6, 128.5, 128.1, 127.8, 127.5, 127.5, 125.1, 116.7, 61.7, 61.6, 53.3, 49.5, 49.0, 29.8, 14.2, 14.2; LC/MS (ESI) m/z 757.3 [M + H]^+^.

*tert-Butyl N-(4-(benzyloxy)-3-(2-((N-(2-(tert-butoxy)-2-oxoethyl)-4-methylphenyl)sulfonamido)ethyl)-phenyl)-N-tosylglycinate (****45****).* Prepared as described in the experimental procedure of **24a** from intermediate **42f** and *tert*-butyl 2-bromoacetate to obtain the title compound as a colorless oil (385 mg, 96%); ^1^H NMR (400 MHz, CDCl_3_) *δ* 7.61 (d, *J* = 8.0 Hz, 2H), 7.54 (d, *J* = 8.0 Hz, 2H), 7.40-4.30 (m, 5H), 7.25 (d, *J* = 8.0 Hz, 2H), 7.20 (d, *J* = 8.0 Hz, 2H), 7.07 (dd, *J* = 8.8, 2.8 Hz, 1H), 6.87 (d, *J* = 2.8 Hz, 1H), 6.76 (d, *J* = 8.8 Hz, 1H), 4.99 (s, 2H), 4.22 (s, 2H), 3.80 (s, 2H), 3.39 (t, *J* = 7.6 Hz, 2H), 2.78 (t, *J* = 7.6 Hz, 2H), 2.41 (s, 3H), 2.38 (s, 3H), 1.39 (s, 9H), 1.34 (s, 9H); ^13^C NMR (100 MHz, CDCl_3_) *δ* 168.1, 167.9, 156.4, 143.7, 143.2, 137.4, 136.7, 136.2, 132.7, 131.1, 129.6, 129.5, 129.2, 128.8, 128.2, 127.9, 127.7, 127.4, 127.4, 111.8, 82.2, 82.0, 70.3, 53.7, 49.2, 48.1, 30.2, 28.1, 28.0, 21.7, 21.6 ; LC/MS (ESI) *m/z* 796.5 [M + H + H_2_O]^+^.

*tert-Butyl N-(5-((N-(2-(tert-butoxy)-2-oxoethyl)-4-methylphenyl)sulfonamido)-2-hydroxyphenethyl)-N-tosylglycinate (****46****).* Prepared as described in the experimental procedure of **25** from intermediate **45** to obtain the title compound as a white solid (306 mg, 90%); ^1^H NMR (400 MHz, CDCl_3_) *δ* 7.69 (d, *J* = 8.4 Hz, 2H), 7.52 (d, *J* = 8.4 Hz, 2H), 7.27 (d, *J* = 8.0 Hz, 2H), 7.23 (d, *J* = 8.0 Hz, 2H), 6.90 (d, *J* = 2.4 Hz, 1H), 6.86 (dd, *J* = 8.4, 2.4 Hz, 1H), 6.69 (d, *J* = 8.4 Hz, 1H), 6.58 (brs, 1H, OH), 4.20 (s, 2H), 3.89 (s, 2H), 3.33 (t, *J* = 7.6 Hz, 2H), 2.85 (t, *J* = 7.6 Hz, 2H), 2.40 (s, 6H), 1.39 (s, 9H), 1.39 (s, 9H); ^13^C NMR (100 MHz, CDCl_3_) *δ* 168.4, 168.0, 154.7, 143.7, 143.6, 136.6, 136.1, 132.0, 131.4, 129.7, 129.5, 128.9, 127.9, 127.5, 125.0, 116.4, 82.5, 82.2, 53.8, 50.0, 48.8, 30.5, 28.1, 28.0, 21.63, 21.59; LC/MS (ESI) *m/z* 577.3 [M + H - C_8_H_16_]^+^.

*tert-Butyl N-(4-(2-(tert-butoxy)-2-oxoethoxy)-3-(2-((N-(2-(tert-butoxy)-2-oxoethyl)-4-methyl-phenyl)-sulfonamido)ethyl)phenyl)-N-tosylglycinate (****47****).* Prepared as described in the experimental procedure of **29** from intermediate **46** and *tert*-butyl 2-bromoacetate to obtain the title compound as a colorless oil (93 mg, 94%); ^1^H NMR (400 MHz, CDCl_3_) *δ* 7.68 (d, *J* = 8.0 Hz, 2H), 7.53 (d, *J* = 8.0 Hz, 2H), 7.25 (d, *J* = 8.0 Hz, 4H), 7.08 (dd, *J* = 8.8, 2.4 Hz, 1H), 6.85 (d, *J* = 2.4 Hz, 1H), 6.54 (d, *J* = 8.8 Hz, 1H), 4.44 (s, 2H), 4.21 (s, 2H), 3.90 (s, 2H), 3.44 (t, *J* = 7.6 Hz, 2H), 2.80 (t, *J* = 7.6 Hz, 2H), 2.41 (s, 3H), 2.40 (s, 3H), 1.44 (s, 9H), 1.39 (s, 9H), 1.37 (s, 9H); ^13^C NMR (100 MHz, CDCl_3_) *δ* 168.3, 167.9, 167.5, 155.6, 143.8, 143.2, 137.5, 136.1, 133.2, 131.2, 129.6, 129.5, 129.2, 128.0, 127.9, 127.5, 111.3, 82.5, 82.2, 81.9, 65.7, 53.7, 49.2, 48.1, 30.3, 28.2, 28.1, 28.1, 21.7, 21.6; LC/MS (ESI) *m/z* 635.3 [M + H - C_12_H_24_]^+^.

*2-(Benzyloxy)-5-nitrophenol (****48****).* To a solution of 4-nitrobenzene-1,2-diol (2.0 g, 12.89 mmol) in tetrahydrofuran (3 mL) was added sodium hydride (60% dispersion in mineral oil, 516 mg, 12.89 mmol) at 0 °C. After stirring at 0 °C for 5 minutes, benzyl bromide (2.2 g, 12.89 mmol) was added at 0 °C. The reaction mixture was stirred at room temperature overnight. After the reaction was completed, the crude mixture was added to water and extracted with dichloromethane. The organic layer was dried over anhydrous Na_2_SO_4_ and concentrated under reduced pressure. The residue was purified by flash column chromatograph (0-15% ethyl acetate/hexane) to give the product as a pale yellow solid (2.4 g, 76%); ^1^H NMR (400 MHz, CDCl_3_) *δ* 7.79-7.76 (m, 2H), 7.41-7.37 (m, 5H), 6.94 (d, *J* = 8.4 Hz, 1H), 5.20 (s, 2H); ^13^C NMR (100 MHz, CDCl_3_) *δ* 151.3, 146.2, 142.1, 135.0, 128.9, 128.8, 127.9, 116.6, 111.1, 110.6, 71.5; LC/MS (ESI) *m/z* 244.1 [M - H]^-^.

*2-(2-(2-Methyl-5-nitrophenoxy)ethyl)isoindoline-1,3-dione (****49a****).* To a solution of 2-methyl-5-nitrophenol (500 mg, 3.26 mmol) in *N,N*-dimethylformamide (10 mL) were added potassium carbonate (677 mg, 4.90 mmol) and *N*-(2-bromoethyl)phthalimide (996 mg, 3.92 mmol) at room temperature. The reaction mixture was stirred at room temperature overnight. After the reaction was completed, the crude mixture was added to water and extracted with ethyl acetate. The organic layer was dried over anhydrous Na_2_SO_4_ and concentrated under reduced pressure. The residue was purified by flash column chromatograph (0-50% ethyl acetate/hexane) to give the product as a white solid (126 mg, 12%); ^1^H NMR (400 MHz, CDCl_3_) δ 7.92-7.89 (m, 2H), 7.87-7.84 (m, 2H), 7.74 (dd, *J* = 8.4, 2.0 Hz, 1H), 7.71 (d, *J* = 2.0 Hz, 1H), 7.38 (d, *J* = 8.4 Hz, 1H), 4.36 (t, *J* = 5.6 Hz, 2H), 4.05 (t, *J* = 5.6 Hz, 2H), 2.12 (s, 3H); LC/MS (ESI) *m/z* 327.0 [M + H]^+^.

*2-(2-(2-Fluoro-5-nitrophenoxy)ethyl)isoindoline-1,3-dione (****49b****).* Prepared as described in the experimental procedure of **49a** from 2-fluoro-5-nitrophenol to obtain the title compound as a white solid (178 mg, 17%); ^1^H NMR (400 MHz, DMSO-*d_6_*) δ 8.01-7.99 (m, 1H), 7.89-7.83 (m, 5H), 7.49-7.44 (m, 1H), 4.45 (t, *J* = 5.6 Hz, 2H), 4.01 (t, *J* = 5.6 Hz, 2H); ^13^C NMR (100 MHz, DMSO-*d_6_*) *δ* 167.6, 155.7 (*J*_C,F_ = 254 Hz), 146.3 (*J*_C,F_ = 12 Hz), 144.1, 134.5, 131.5, 123.1, 117.5 (*J*_C,F_ = 9 Hz), 116.8 (*J*_C,F_ = 21 Hz), 110.3 (*J*_C,F_ = 4 Hz), 66.4; LC/MS (ESI) *m/z* 330.0 [M]^-^.

*2-(2-(2-(Benzyloxy)-5-nitrophenoxy)ethyl)isoindoline-1,3-dione (****49c****).* Prepared as described in the experimental procedure of **49a** from intermediate **48** to obtain the title compound as a white solid (467 mg, 55%); ^1^H NMR (400 MHz, CDCl_3_) *δ* 7.85-7.77 (m, 4H), 7.74-7.69 (m, 2H), 7.38-7.35 (m, 5H), 6.90 (d, *J* = 8.8 Hz, 1H), 5.13 (s, 2H), 4.37-4.35 (m, 2H), 4.20-4.19 (m, 2H); ^13^C NMR (100 MHz, CDCl_3_) *δ* 168.2, 154.3, 148.2, 141.6, 135.8, 134.2, 132.2, 128.8, 128.3, 127.1, 123.5, 118.5, 112.5, 109.1, 71.1, 66.2, 37.1; LC/MS (ESI) *m/z* 419.2 [M + H]^+^.

*4-Methoxy-N-(2-(2-methyl-5-nitrophenoxy)ethyl)benzenesulfonamide (****50a****).* To a solution of **49a** (126 mg, 0.39 mmol) in methanol (5 mL) and dichloromethane (3 mL) was added hydrazine monohydrate (58 mg, 1.16 mmol) at room temperature. The reaction mixture was stirred at 60 °C for overnight. After the reaction was completed, the crude mixture was cooled to room temperature, added to sat. NaHCO_3_ and then extracted with dichloromethane. The organic layer was dried over anhydrous Na_2_SO_4,_ and concentrated under reduced pressure to give 2-(2-methyl-5-nitrophenoxy)ethan-1-amine as a pale yellow oil (113 mg, quantitative); ^1^H NMR (400 MHz, CD_3_OD) *δ* 7.75 (dd, *J* = 8.0, 2.4 Hz, 1H), 7.72 (d, *J* = 8.4 Hz, 1H), 7.35 (d, *J* = 8.0 Hz, 1H), 4.16 (t, *J* = 5.2 Hz, 2H), 3.15 (t, *J* = 5.2 Hz, 2H), 2.33 (s, 3H); ^13^C NMR (100 MHz, CD_3_OD) *δ* 158.4, 136.4, 131.8, 126.8, 116.8, 106.6, 70.4, 41.4, 16.6; LC/MS (ESI) *m/z* 197.1 [M + H]^+^.

Prepared as described in the experimental procedure of **41c** from 2-(2-methyl-5-nitrophenoxy)ethan-1-amine and 4-methoxybenzenesulfonyl chloride to obtain the title compound as a white solid (60 mg, 28%); ^1^H NMR (400 MHz, CDCl_3_) *δ* 7.81 (d, *J* = 8.0 Hz, 2H), 7.73 (dd, *J* = 8.4, 2.0 Hz, 1H), 7.46 (d, *J* = 2.0 Hz, 1H), 7.22 (d, *J* = 8.4 Hz, 1H), 6.91 (d, *J* = 8.8 Hz, 2H), 5.37 (s, 1H, NH), 4.03 (t, *J* = 5.2 Hz, 2H), 3.86 (s, 3H), 3.46-3.39 (m, 2H), 2.27 (s, 3H), 3.08-3.03 (m, 2H), 2.64 (t, *J* = 7.2 Hz, 2H), 2.08 (s, 3H); ^13^C NMR (100 MHz, CDCl_3_) *δ* 163.2, 156.4, 147.1, 135.2, 131.6, 130.8, 129.2, 116.3, 114.5, 105.6, 67.0, 55.7, 42.4, 16.7; LC/MS (ESI) *m/z* 367.1 [M + H]^+^.

*N-(2-(2-Fluoro-5-nitrophenoxy)ethyl)-4-methylbenzenesulfonamide (****50b****).* Prepared as described in the experimental procedure of **50a** from intermediate **49b** to obtain the title compound as a pale yellow solid (182 mg, 47% over 2 steps); ^1^H NMR (400 MHz, DMSO-*d_6_*) *δ* 7.91-7.86 (m, 2H), 7.88 (brs, 1H, NH), 7.69 (d, *J* = 8.4 Hz, 2H), 7.53-7.48 (m, 1H), 7.34 (d, *J* = 8.4 Hz, 2H), 4.17 (t, *J* = 5.6 Hz, 2H), 3.18-3.16 (m, 2H), 2.34 (s, 3H); ^13^C NMR (100 MHz, DMSO-*d_6_*) *δ* 155.6 (*J*_C,F_ = 254 Hz), 146.4 (*J*_C,F_ = 12 Hz), 144.1, 142.6, 137.6, 129.5, 126.4, 117.4 (*J*_C,F_ = 8 Hz), 116.8 (*J*_C,F_ = 20 Hz), 110.2 (*J*_C,F_ = 3 Hz), 68.2, 41.6, 20.9; LC/MS (ESI) *m/z* 355.1 [M + H]^+^.

*N-(2-(2-(Benzyloxy)-5-nitrophenoxy)ethyl)-4-methylbenzenesulfonamide (****50c****).* Prepared as described in the experimental procedure of **50a** from intermediate **49c** to obtain 2-(2-(benzyloxy)-5-nitrophenoxy)ethan-1-amine as a pale yellow oil (351 mg, quantitative); ^1^H NMR (400 MHz, CDCl_3_) *δ* 7.85 (dd, *J* = 8.8, 2.8 Hz, 1H), 7.77 (d, *J* = 2.8 Hz, 1H), 7.43-7.31 (m, 5H), 6.94 (d, *J* = 8.8 Hz, 1H), 5.21 (s, 2H), 4.10 (t, *J* = 5.2 Hz, 2H), 3.13 (t, *J* = 5.2 Hz, 2H), 1.69 (brs, 2H, NH); ^13^C NMR (100 MHz, CDCl_3_) *δ* 154.3, 148.8, 141.7, 135.9, 128.9, 128.5, 127.3, 118.0, 112.4, 108.9, 72.0, 71.2, 41.4; LC/MS (ESI) *m/z* 289.2 [M + H]^+^.

Prepared as described in the experimental procedure of **50a** from 2-(2-(benzyloxy)-5-nitrophenoxy)-ethan-1-amine and 4-methylbenzenesulfonyl chloride to obtain the title compound as a pale yellow solid (288 mg, 56%); ^1^H NMR (400 MHz, DMSO-*d_6_*) *δ* 7.90-7.87 (m, 2H), 7.71 (brs, 1H, NH), 7.69 (d, *J* = 8.0 Hz, 2H), 7.48 (d, *J* = 7.6 Hz, 2H), 7.43-7.39 (m, 2H), 3.37-7.34 (m, 1H), 7.32 (d, *J* = 8.0 Hz, 2H), 7.25 (dd, *J* = 9.2, 1.6 Hz, 1H), 5.28 (s, 2H), 4.10 (t, *J* = 5.2 Hz, 2H), 3.15-3.11 (m, 2H), 2.34 (s, 3H); ^13^C NMR (100 MHz, DMSO-*d_6_*) *δ* 154.0, 147.7, 142.6, 140.7, 137.5, 136.1, 129.6, 128.5, 128.1, 127.6, 126.5, 118.1, 113.0, 108.8, 70.3, 67.9, 41.7, 20.9.

*4-Methoxy-N-(2-(5-((4-methoxyphenyl)sulfonamido)-2-methylphenoxy)ethyl)benzenesulfonamide (****51a****).* Prepared as described in the experimental procedure of **23a** from intermediate **50a** to obtain the title compound as a pale yellow solid (45 mg, 51% over two steps); ^1^H NMR (400 MHz, CDCl_3_) *δ* 7.81 (d, *J* = 8.8 Hz, 2H), 7.67 (d, *J* = 8.8 Hz, 2H), 7.03 (s, 1H, NH), 6.95 (d, *J* = 8.8 Hz, 2H), 6.89 (d, *J* = 8.4 Hz, 1H), 6.86 (d, *J* = 8.8 Hz, 2H), 6.56 (s, 1H), 6.47 (d, *J* = 8.4 Hz, 1H), 5.09 (t, *J* = 5.6 Hz, 1H, NH), 3.91-3.84 (m, 2H), 3.82 (s, 3H), 3.80 (s, 3H), 3.33-3.29 (m, 2H), 2.04 (s, 3H); ^13^C NMR (100 MHz, CDCl_3_) *δ* 163.2, 163.2, 156.6, 135.5, 131.5, 131.1, 130.7, 129.6, 129.3, 124.0, 114.5, 114.3, 105.8, 66.6, 55.8, 55.7, 42.6, 15.8; LC/MS (ESI) *m/z* 507.2 [M + H]^+^.

*N-(4-Fluoro-3-(2-((4-methylphenyl)sulfonamido)ethoxy)phenyl)-4-methylbenzenesulfonamide (****51b****).* Prepared as described in the experimental procedure of **23a** from intermediate **50b** to obtain the title compound as a pale yellow solid (203 mg, 87% over 2 steps); ^1^H NMR (400 MHz, DMSO-*d_6_*) *δ* 10.12 (brs, 1H, NH), 7.85 (t, *J* = 5.6 Hz, 1H, NH), 7.69 (d, *J* = 8.0 Hz, 2H), 7.61 (d, *J* = 8.4 Hz, 2H), 7.36-7.32 (m, 4H), 7.04 (dd, *J* = 11.2, 8.8 Hz, 1H), 6.78 (dd, *J* = 7.6, 2.4 Hz, 1H), 6.59-6.55 (m, 1H), 3.89 (t, *J* = 5.6 Hz, 2H), 3.11-3.07 (m, 2H), 2.35 (s, 3H), 2.32 (s, 3H); ^13^C NMR (100 MHz) (DMSO-d_6_) δ ^13^C NMR (100 MHz, DMSO-*d_6_*) *δ* 148.6 (*J*_C,F_ = 239 Hz), 145.9 (*J*_C,F_ = 12 Hz), 143.3, 142.6, 137.5, 136.3, 134.3 (*J*_C,F_ = 3 Hz), 129.6, 129.6, 126.7, 126.5, 116.2 (*J*_C,F_ = 19 Hz), 113.0 (*J*_C,F_ = 7 Hz), 107.8, 67.6, 41.7, 20.9; LC/MS (ESI) *m/z* 479.2 [M + H]^+^.

*N-(4-(Benzyloxy)-3-(2-((4-methylphenyl)sulfonamido)ethoxy)phenyl)-4-methylbenzenesulfonamide (****51c****).* Prepared as described in the experimental procedure of **23a** from intermediate **50c** to obtain the title compound as a pale yellow solid (317 mg, 89% over two steps); ^1^H NMR (400 MHz, DMSO-*d_6_*) *δ* 9.88 (brs, 1H, NH), 7.81 (brs, 1H, NH), 7691 (d, *J* = 8.4 Hz, 2H), 7.58 (d, *J* = 8.0 Hz, 2H), 7.39-7.30 (m, 9H), 6.86 (d, *J* = 8.8 Hz, 1H), 6.68 (d, *J* = 2.4 Hz, 1H), 6.53 (dd, *J* = 8.8, 2.4 Hz, 1H), 4.98 (s, 2H), 3.84 (t, *J* = 5.6 Hz, 1H, NH), 3.07-3.03 (m, 2H), 2.36 (s, 3H), 2.32 (s, 3H); ^13^C NMR (100 MHz, DMSO-*d_6_*) *δ* 148.2, 145.3, 143.1, 142.7, 137.5, 137.1, 136.6, 131.3, 129.6, 129.5, 128.3, 127.7, 127.6, 126.7, 126.5, 115.2, 114.1, 108.5, 70.4, 67.6, 41.8, 20.9; LC/MS (ESI) *m/z* 567.3 [M + H]^+^.

*Ethyl N-(2-(5-((N-(2-ethoxy-2-oxoethyl)-4-methoxyphenyl)sulfonamido)-2-methylphenoxy)ethyl)-N-((4-methoxyphenyl)sulfonyl)glycinate (****52a****).* Prepared as described in the experimental procedure of **24a** from intermediate **51a** to obtain the title compound as a colorless oil (40 mg, 66%); ^1^H NMR (400 MHz, CDCl_3_) *δ* 7.78 (d, *J* = 8.8 Hz, 2H), 7.62 (d, *J* = 8.8 Hz, 2H), 6.99-6.92 (m, 5H), 6.67 (d, *J* = 2.0 Hz, 1H), 6.62 (dd, *J* = 8.0, 2.0 Hz, 1H), 4.34 (s, 2H), 4.24 (s, 2H), 4.14 (q, *J* = 7.2 Hz, 2H), 4.05-3.99 (m, 4H), 3.86 (s, 3H), 3.85 (s, 3H), 3.63 (t, *J* = 5.2 Hz, 2H), 2.10 (s, 3H), 1.22 (t, *J* = 6.8 Hz, 3H), 1.15 (t, *J* = 7.2 Hz, 3H); ^13^C NMR (100 MHz, CDCl_3_) *δ* 169.2, 169.0, 163.2, 163.2, 156.5, 138.7, 131.5, 130.8, 130.7, 130.2, 129.6, 126.9, 120.7, 114.3, 114.0, 112.0, 67.7, 61.6, 61.4, 55.8, 55.7, 53.0, 49.9, 47.6, 16.2, 14.2, 14.1; LC/MS (ESI) *m/z* 679.2 [M + H]^+^.

*Ethyl N-(2-(5-((N-(2-ethoxy-2-oxoethyl)-4-methylphenyl)sulfonamido)-2-fluorophenoxy)ethyl)-N-tosyl-glycinate (****52b****).* Prepared as described in the experimental procedure of **24a** from intermediate **51b** to obtain the title compound as a colorless oil (183 mg, 90%); ^1^H NMR (400 MHz, CDCl_3_) *δ* 7.73 (d, *J* = 8.4 Hz, 2H), 7.56 (d, *J* = 8.4 Hz, 2H), 7.31-7.27 (m, 4H), 7.04 (dd, *J* = 10.8, 8.8 Hz, 1H), 6.83 (dd, *J* = 7.6, 2.4 Hz, 1H), 6.72-6.68 (m, 1H), 4.35 (s, 2H), 4.25 (s, 2H), 4.16 (q, *J* = 7.2 Hz, 2H), 4.10 (t, *J* = 5.2 Hz, 2H), 4.04 (q, *J* = 7.2 Hz, 2H), 3.65 (t, *J* = 5.2 Hz, 2H), 2.43 (s, 3H), 2.42 (s, 3H), 1.24 (t, *J* = 7.2 Hz, 3H), 1.17 (t, *J* = 7.2 Hz, 3H); ^13^C NMR (100 MHz, CDCl_3_) *δ* 169.1, 168.8, 152.1 (*J*_C,F_ = 247 Hz), 146.5 (*J*_C,F_ = 11 Hz), 144.2, 143.8, 136.8, 136.1 (*J*_C,F_ = 4 Hz), 135.8, 129.8, 129.6, 128.0, 127.5, 122.0 (*J*_C,F_ = 7 Hz), 116.2 (*J*_C,F_ = 19 Hz), 116.1 (*J*_C,F_ = 2 Hz), 69.3, 61.7, 61.4, 53.0, 50.1, 47.6, 21.7, 21.6, 14.2, 14.1; LC/MS (ESI) *m/z* 651.3 [M + H]^+^.

*Ethyl N-(4-(benzyloxy)-3-(2-((N-(2-ethoxy-2-oxoethyl)-4-methylphenyl) sulfonamido)ethoxy)-phenyl)-N-tosylglycinate (****52c****).* Prepared as described in the experimental procedure of **24a** from intermediate **51c** to obtain the title compound as a colorless oil (171 mg, 75%); ^1^H NMR (400 MHz, CDCl_3_) *δ* 7.70 (d, *J* = 8.0 Hz, 2H), 7.55 (d, *J* = 8.0 Hz, 2H), 7.40-7.31 (m, 5H), 7.26 (d, *J* = 8.0 Hz, 2H), 7.25 (d, *J* = 8.0 Hz, 2H), 6.78 (d, *J* = 8.8 Hz, 1H), 6.73 (dd, *J* = 8.8, 2.0 Hz, 1H), 6.67 (d, *J* = 2.0 Hz, 1H), 5.02 (s, 2H), 4.34 (s, 2H), 4.26 (s, 2H), 4.14 (q, *J* = 7.2 Hz, 2H), 4.03 (t, *J* = 5.2 Hz, 2H), 3.94 (q, *J* = 7.2 Hz, 2H), 3.62 (t, *J* = 5.2 Hz, 2H), 2.42 (s, 3H), 2.40 (s, 3H), 1.22 (t, *J* = 7.2 Hz, 3H), 1.09 (t, *J* = 7.2 Hz, 3H); ^13^C NMR (100 MHz, CDCl_3_) *δ* 169.2, 169.0, 148.6, 148.2, 143.9, 143.6, 136.9, 136.6, 136.0, 132.8, 129.7, 129.5, 128.7, 128.1, 128.0, 127.6, 127.4, 122.3, 114.6, 113.2, 71.0, 69.2, 61.5, 61.2, 53.1, 50.0, 47.5, 21.7, 21.6, 14.2, 14.1; LC/MS (ESI) *m/z* 739.3 [M + H]^+^.

*Ethyl N-(2-(5-((N-(2-ethoxy-2-oxoethyl)-4-methylphenyl)sulfonamido)-2-hydroxyphenoxy)ethyl)-N-tosylglycinate (****53****).* Prepared as described in the experimental procedure of **25** from intermediate **52c** to obtain the title compound as a white solid (137 mg, 91%); ^1^H NMR (400 MHz, CDCl_3_) *δ* 7.75 (d, *J* = 8.4 Hz, 2H), 7.53 (d, *J* = 8.4 Hz, 2H), 7.30 (d, *J* = 8.4 Hz, 2H), 7.24 (d, *J* = 8.4 Hz, 2H), 6.84 (d, *J* = 2.0 Hz, 1H), 6.73 (d, *J* = 8.8 Hz, 1H), 6.64 (brs, 1H, OH), 6.47 (dd, *J* = 8.8, 2.0 Hz, 1H), 4.32 (s, 2H), 4.16-4.05 (m, 8H), 3.62 (t, *J* = 4.8 Hz, 2H), 2.41 (s, 3H), 2.41 (s, 3H), 1.22 (t, *J* = 7.2 Hz, 3H), 1.17 (t, *J* = 7.2 Hz, 3H); ^13^C NMR (100 MHz, CDCl_3_) *δ* 169.3, 169.0, 146.6, 145.5, 144.1, 143.8, 136.2, 136.0, 131.5, 129.9, 129.4, 128.0, 127.6, 122.2, 115.0, 114.6, 66.6, 61.9, 61.5, 53.2, 49.5, 47.8, 21.7, 14.2, 14.1; LC/MS (ESI) *m/z* 649.3 [M + H]^+^.

*1-(Benzyloxy)-2-iodo-4-nitrobenzene (****54****).* To a solution of 4-nitrophenol (2.0 g, 14.38 mmol) in methanol (10 mL) and water (60 mL) were added potassium iodide (1.6 g, 9.49 mmol) and potassium iodate (1.0 g, 4.74 mmol) at room temperature. After stirring at 0 °C for 10 minutes, con. HCl (1.2 mL) in water (10 mL) was slowly added at room temperature for 1 hour. The reaction mixture was stirred at room temperature overnight. After the reaction was completed, the crude mixture was cooled to room temperature, diluted with water, and extracted with dichloromethane. The organic layer was dried over anhydrous Na_2_SO_4_ and concentrated under reduced pressure to give the product, 2-iodo-4-nitrophenol**,** as a pale yellow solid. The resulting crude compound was used for the next reaction without further purification.

Prepared as described in the experimental procedure of **19** from iodo-4-nitrophenol to obtain the title compound as a pale yellow solid (604 mg, 12% over two steps); ^1^H NMR (400 MHz, DMSO-*d_6_*) *δ* 8.58 (d, *J* = 2.8 Hz, 1H), 8.28 (dd, *J* = 9.2, 2.8 Hz, 1H), 7.51 (d, *J* = 7.2 Hz, 2H), 7.43 (dd, *J* = 7.4, 7.4 Hz, 2H), 7.39-7.34 (m, 1H), 7.28 (d, *J* = 9.2 Hz, 1H), 5.36 (s, 2H); ^13^C NMR (100 MHz, DMSO-*d_6_*) *δ* 162.1, 141.5, 135.7, 134.1, 128.6, 128.1, 127.4, 125.8, 112.3, 86.8, 71.1.

*(E)-N-(3-(2-(Benzyloxy)-5-nitrophenyl)allyl)-4-methylbenzenesulfonamide (****55****).* To a solution of **54** (294mg, 0.83 mmol) in acetonitrile (4 mL) were added *N*-​allyl-​*p*-​toluenesulfonamide (210 mg, 0.99 mmol), palladium (II) acetate (11 mg, 0.050 mmol), tri(o-tolyl)phosphine (30 mg, 0.099 mmol) and triethylamine (117 mg, 1.16 mmol) at room temperature. The reaction mixture was stirred at 80 °C overnight. After the reaction was completed, the crude mixture was added to water and extracted with dichloromethane. The organic layer was dried over anhydrous Na_2_SO_4_ and concentrated under reduced pressure. The residue was purified by flash column chromatograph (0-20% ethyl acetate/hexane) to give the product as a pale yellow solid (162 mg, 45%); ^1^H NMR (400 MHz, CDCl_3_) *δ* 8.10 (s, 1H), 8.08 (d, *J* = 9.2 Hz, 1H), 7.77 (d, *J* = 8.0 Hz, 2H), 7.44-7.36 (m, 5H), 7.29 (d, *J* = 8.0 Hz, 2H), 6.94 (d, *J* = 9.2 Hz, 1H), 6.80 (d, *J* = 15.6 Hz, 1H), 6.16-6.09 (m, 1H), 5.19 (s, 2H), 4.66 (t, *J* = 6.0 Hz, 1H, NH), 3.81-3.78 (m, 2H), 2.40 (s, 3H); ^13^C NMR (100 MHz, CDCl_3_) *δ* 160.4, 143.9, 141.7, 137.4, 135.5, 129.9, 129.0, 128.7, 128.2, 127.5, 127.4, 126.6, 126.0, 124.8, 122.7, 112.1, 71.2, 45.7, 21.6; LC/MS (ESI) *m/z* 437.1 [M - H]^-^.

*(E)-N-(4-(Benzyloxy)-3-(3-((4-methylphenyl)sulfonamido)prop-1-en-1-yl)phenyl)-4-methylbenzene-sulfonamide (****56****).* Prepared as described in the experimental procedure of **23a** from intermediate **55** to obtain the title compound as a pale yellow solid (113 mg, 48% over 2 steps); ^1^H NMR (400 MHz, DMSO-*d_6_*) *δ* 9.85 (brs, 1H, NH), 7.77 (t, *J* = 6.0 Hz, 1H, NH), 7.67 (d, *J* = 8.0 Hz, 2H), 7.57 (d, *J* = 8.4 Hz, 2H), 7.41-7.37 (m, 4H), 7.35-7.31 (m, 5H), 7.03 (d, *J* = 2.4 Hz, 1H), 6.92 (d, *J* = 8.8 Hz, 1H), 6.85 (dd, *J* = 8.8, 2.4 Hz, 1H), 6.63 (d, *J* = 16.0 Hz, 1H), 5.91-5.84 (m, 1H), 5.03 (s, 2H), 3.54-3.51 (m, 2H), 2.33 (s, 6H); ^13^C NMR (100 MHz, DMSO-*d_6_*) *δ* 152.4, 143.1, 142.6, 137.8, 136.9, 136.6, 130.6, 129.9, 129.6, 128.4, 127.8, 127.4, 126.9, 126.7, 126.5, 125.6, 125.4, 122.0, 119.9, 113.4, 69.7, 44.8, 20.9; LC/MS (ESI) *m/z* 563.3 [M + H]^+^.

*Ethyl (E)-N-(4-(benzyloxy)-3-(3-((N-(2-ethoxy-2-oxoethyl)-4-methylphenyl) sulfonamido)prop-1-en-1-yl)phenyl)-N-tosylglycinate (****57****).* Prepared as described in the experimental procedure of **24a** from intermediate **56** to obtain the title compound as a pale yellow oil (136 mg, 97%); ^1^H NMR (400 MHz, CDCl_3_) *δ* 7.70 (d, *J* = 8.0 Hz, 2H), 7.52 (d, *J* = 8.4 Hz, 2H), 7.34-7.33 (m, 5H), 7.26-7.22 (m, 4H), 7.07 (d, *J* = 2.8 Hz, 1H), 7.03 (dd, *J* = 8.8, 2.4 Hz, 1H), 6.77 (d, *J* = 8.8 Hz, 1H), 6.69 (d, *J* = 16.0 Hz, 1H), 5.88-5.81 (m, 1H), 5.00 (s, 2H), 4.30 (s, 2H), 4.13-4.06 (m, 4H), 3.99-3.96 (m, 4H), 2.40 (s, 3H), 2.38 (s, 3H), 1.17 (t, *J* = 7.2 Hz, 3H), 1.09 (t, *J* = 7.2 Hz, 3H); ^13^C NMR (100 MHz, CDCl_3_) *δ* 168.9, 168.8, 155.6, 144.0, 143.6, 137.1, 136.6, 136.2, 132.8, 130.1, 129.7, 129.5, 128.8, 128.7, 128.2, 128.0, 127.8, 127.5, 127.4, 126.3, 125.3, 112.8, 70.6, 61.5, 61.3, 53.0, 50.6, 47.1, 21.6, 21.6, 14.2, 14.1; LC/MS (ESI) *m/z* 735.3 [M + H]^+^.

*Ethyl N-(3-(5-((N-(2-ethoxy-2-oxoethyl)-4-methylphenyl)sulfonamido)-2-hydroxyphenyl)propyl)-N-tosylglycinate (****58****).* Prepared as described in the experimental procedure of **25** from intermediate **57** to obtain the title compound as a pale yellow solid (68 mg, 57%); ^1^H NMR (400 MHz, CDCl_3_) *δ* 7.69 (d, *J* = 8.0 Hz, 2H), 7.53 (d, *J* = 8.0 Hz, 2H), 7.29 (d, *J* = 8.0 Hz, 2H), 7.23 (d, *J* = 8.0 Hz, 2H), 6.89 (d, *J* = 2.0 Hz, 1H), 6.85 (dd, *J* = 8.4, 2.4 Hz, 1H), 6.66 (d, *J* = 8.4 Hz, 1H), 6.38 (brs, 1H, OH), 4.32 (s, 2H), 4.15-4.09 (m, 4H), 3.96 (s, 2H), 3.23 (t, *J* = 6.4 Hz, 2H), 2.57 (t, *J* = 7.6 Hz, 2H), 2.40 (s, 6H), 1.73-1.70 (m, 2H), 1.22-1.18 (m, 6H); ^13^C NMR (100 MHz, CDCl_3_) *δ* 169.8, 169.1, 154.2, 143.7, 136.4, 136.2, 132.0, 131.2, 129.8, 129.5, 128.4, 128.1, 128.0, 127.5, 116.2, 61.8, 61.5, 53.2, 49.0, 48.9, 28.3, 27.3, 21.7, 14.2, 14.1; LC/MS (ESI) *m/z* 647.3 [M + H]^+^.

**3. Table S1 original data of activity in FP assay for final compounds**

| **Compd** | **Structure** | **IC_50_ (μM)^a^** | **Curve** |
| --- | --- | --- | --- |
| **7a** | **** | **1.91±0.50** | 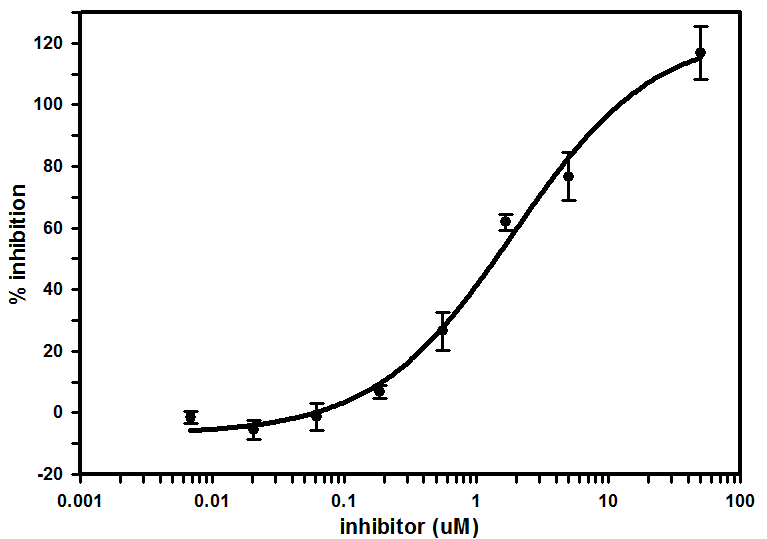 |
| **7d** | **** | **10.03±3.45** | 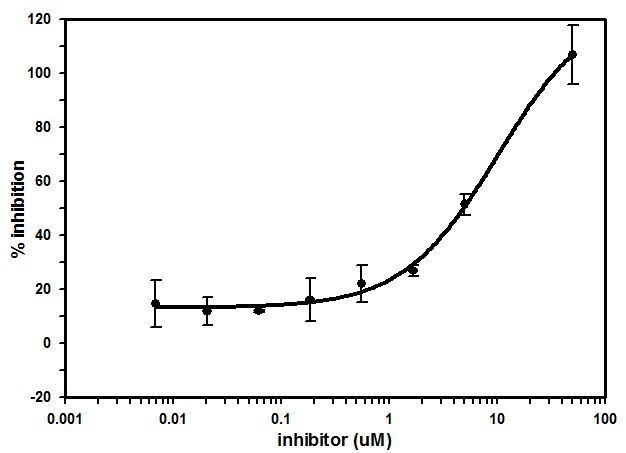 |
| **7e** | **** | **1.54±0.27** | 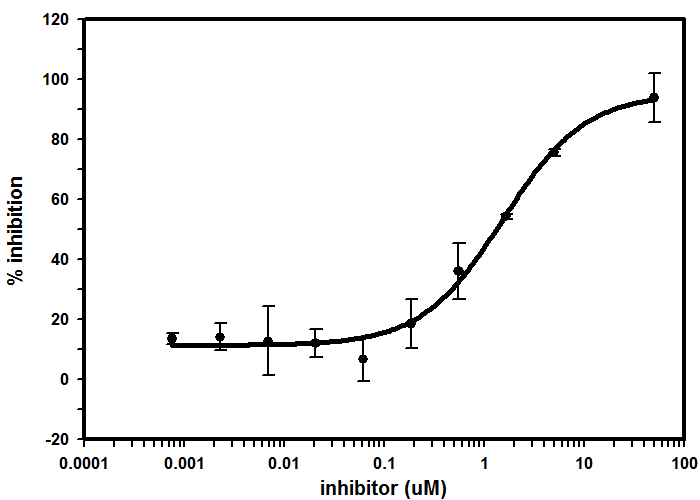 |
| **10** | **** | **1.08±0.40** | 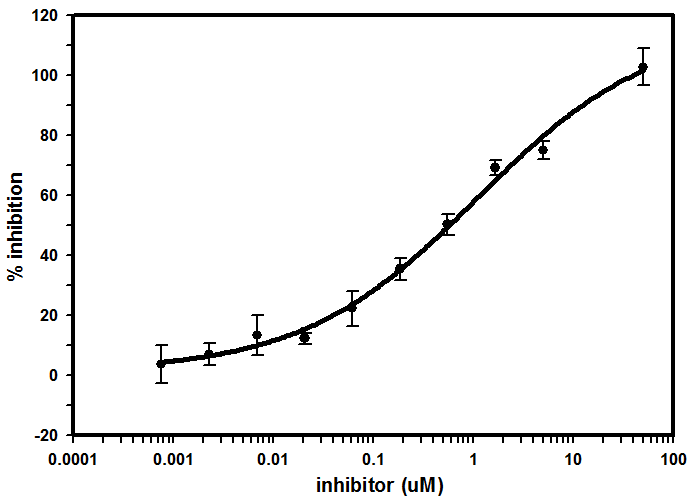 |
| **12a** | **** | **7.68±2.73** | 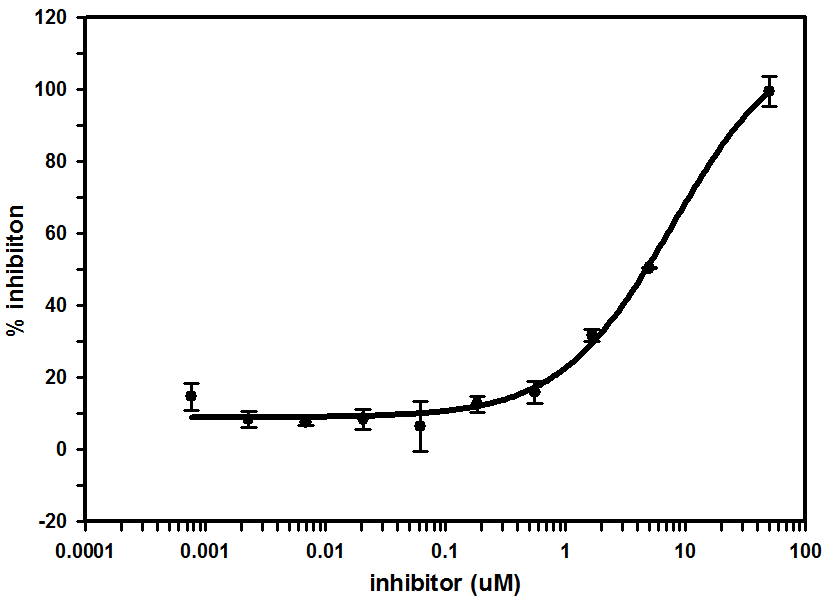 |
| **12b** | **** | **5.22±1.19** | 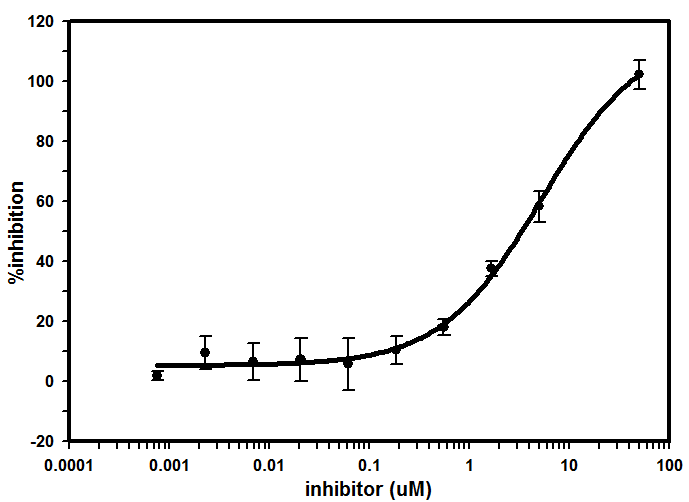 |
| **12d** | **** | **6.13±1.61** | 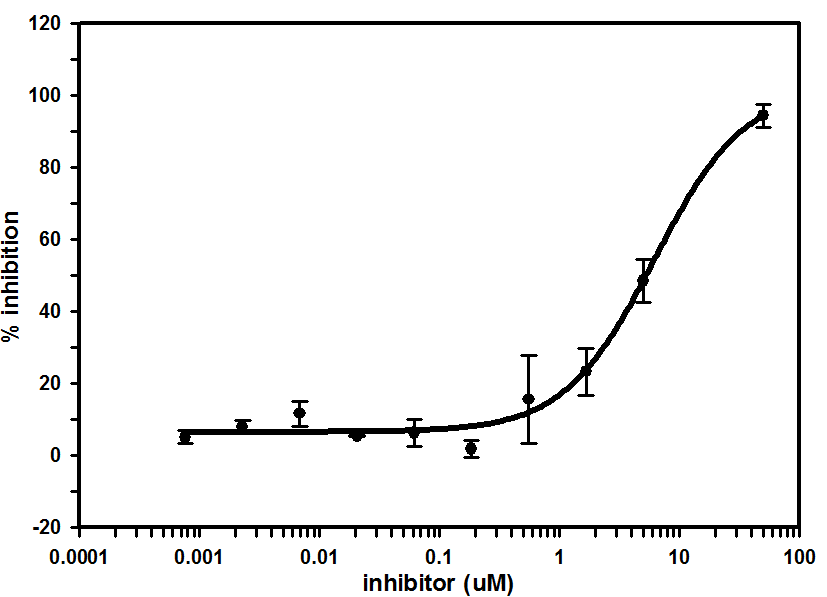 |
| **13a** | **** | **0.44±0.13** | 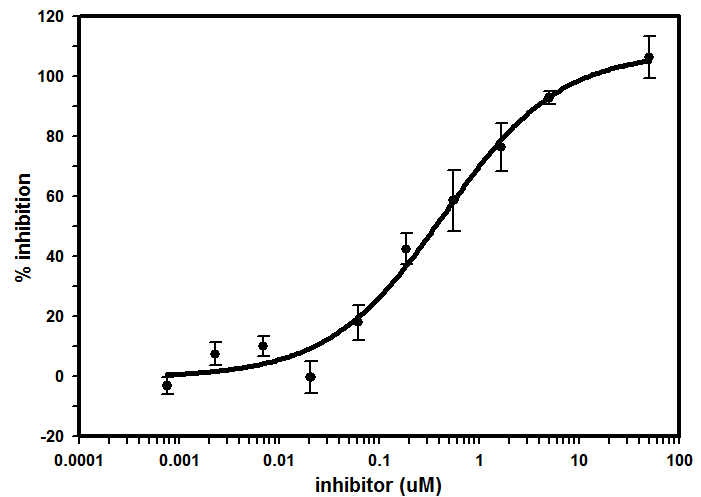 |
| **13b** | **** | **0.18±0.03** | 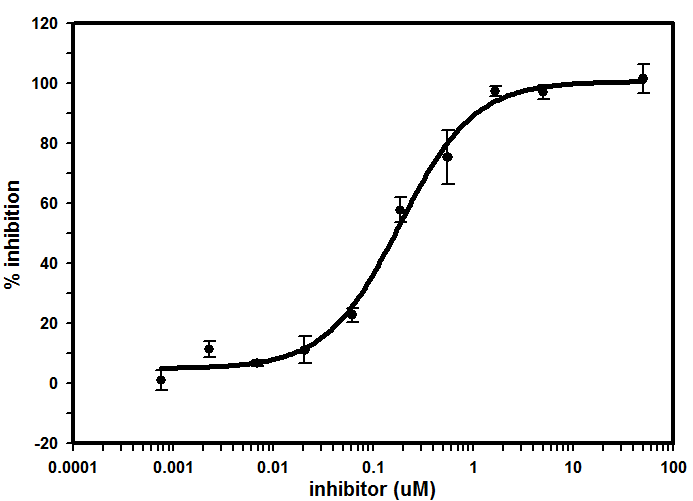 |
| **13c** | **** | **1.13±0.27** | 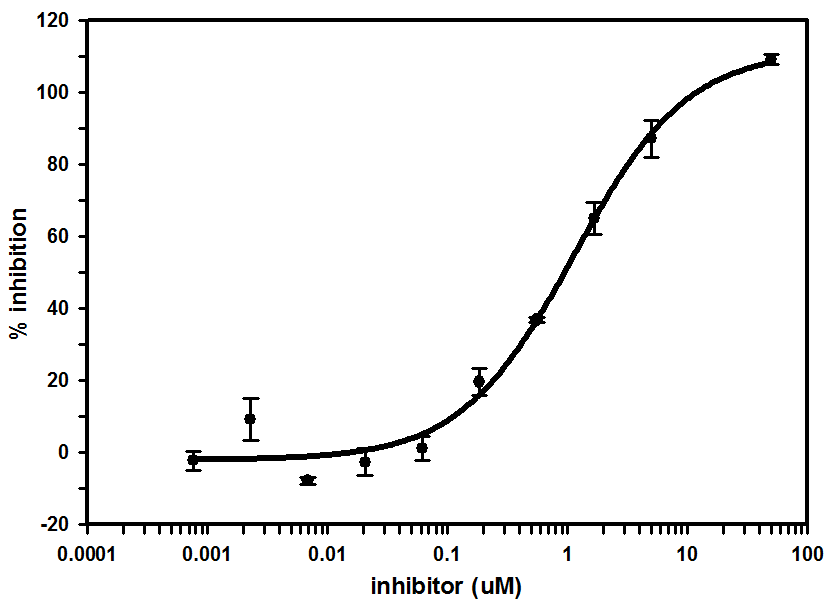 |
| **15a** | **** | **4.29±1.39** | 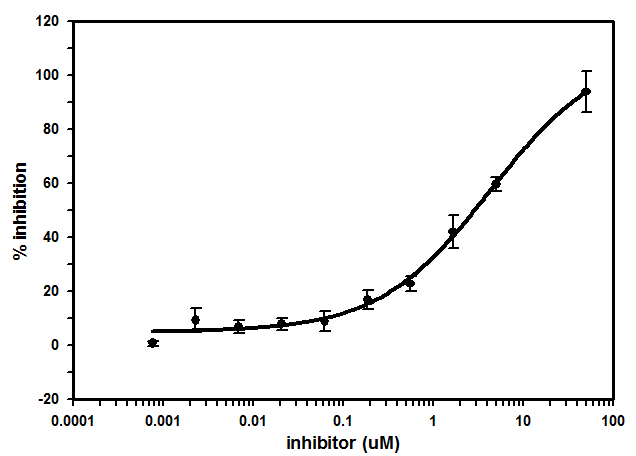 |
| **15b** | **** | **10.85±7.05** | 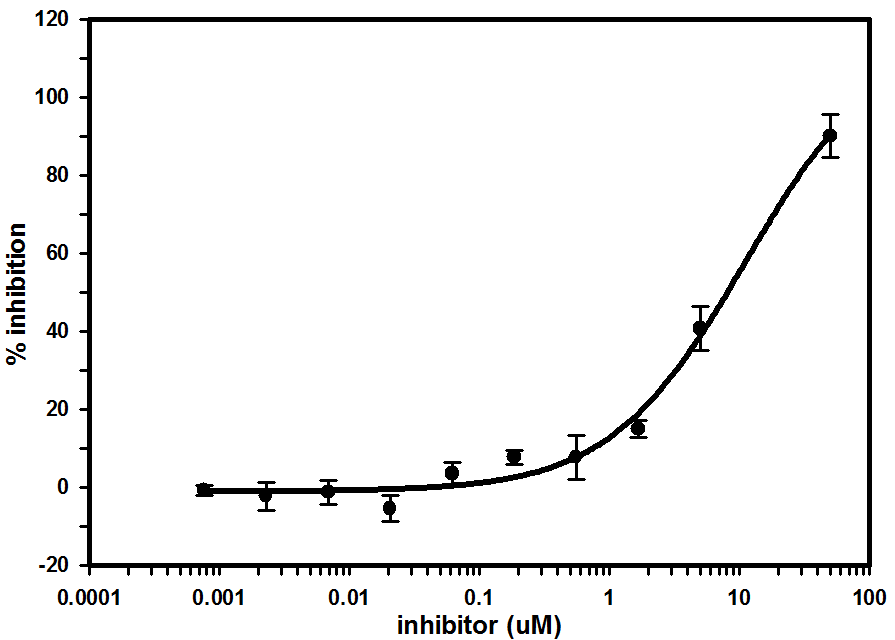 |
| **16** | **** | **1.39±0.15** | 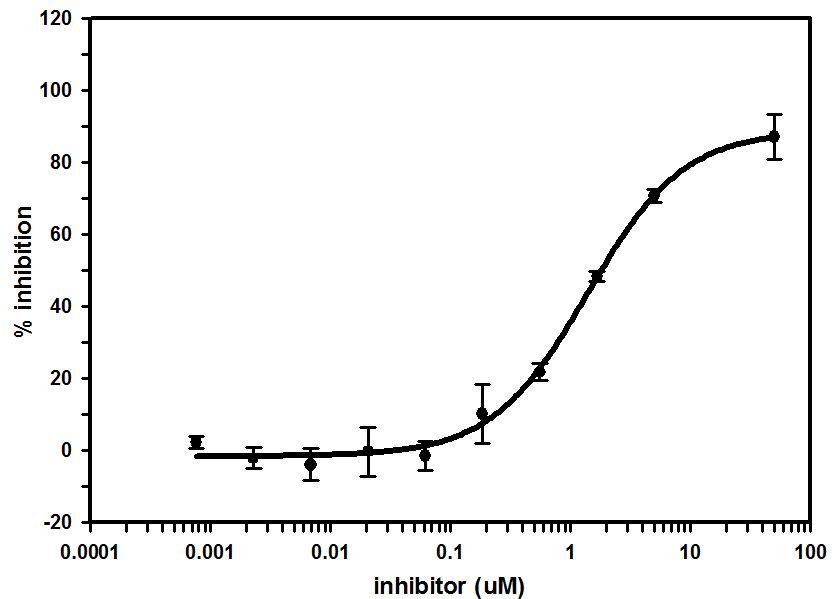 |
| **17** | **** | **0.96±0.28** | 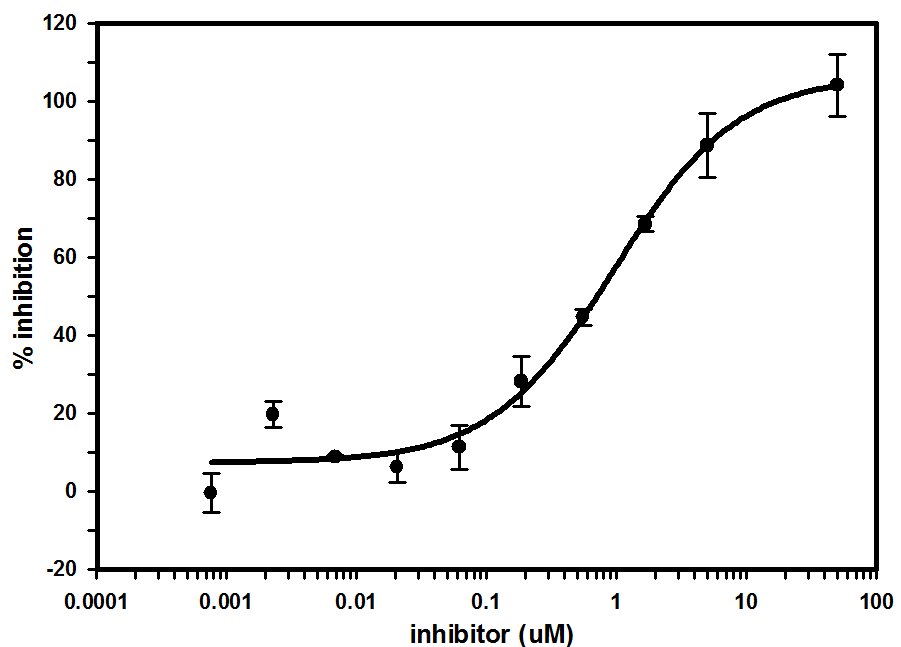 |

^a^Inhibitory potency was determined by FP assay, and all experiments were performed in triplicate. IC_50_ values are reported as an average of three replicates ± standard error of the mean (SEM).

**4. ^1^H and ^13^C NMR spectra of final compounds**

*^1^H-NMR of compound* ***7a***

***
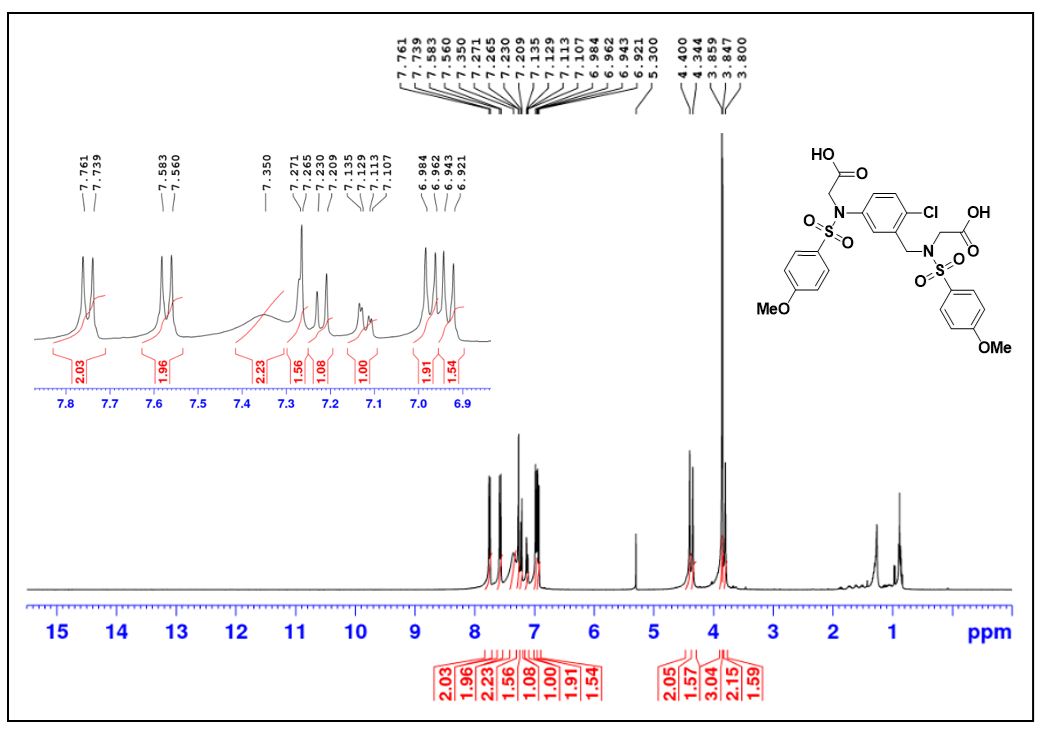
***

*^13^C-NMR of compound* ***7a***

***
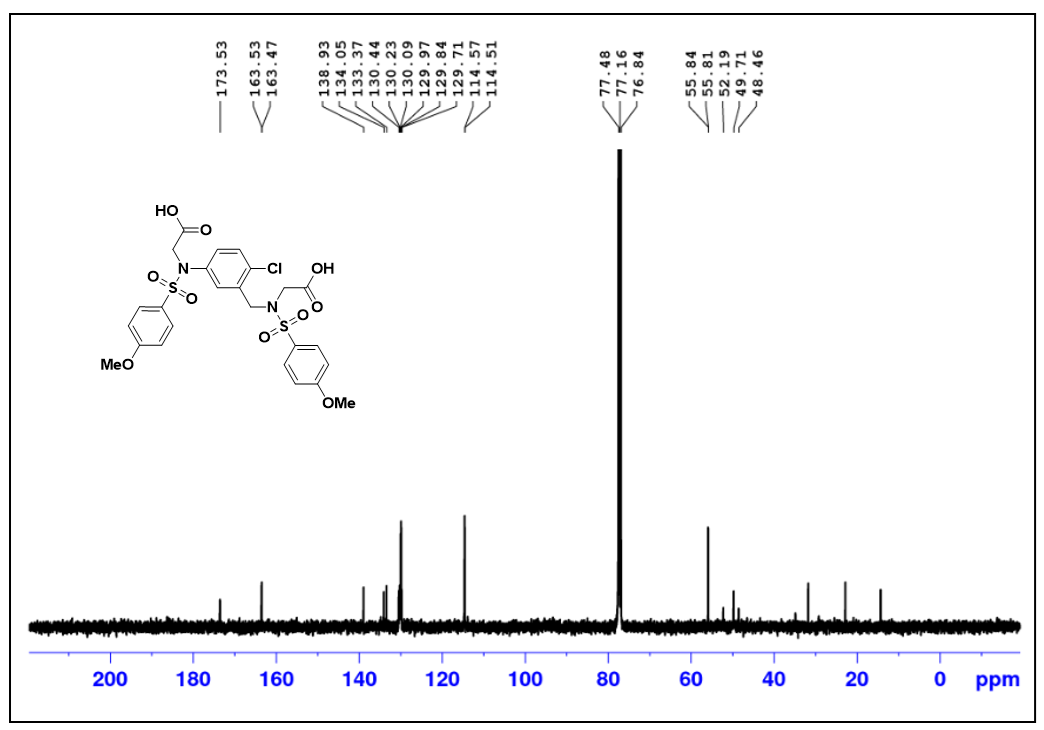
***

*^1^H-NMR of compound* ***7b***

***
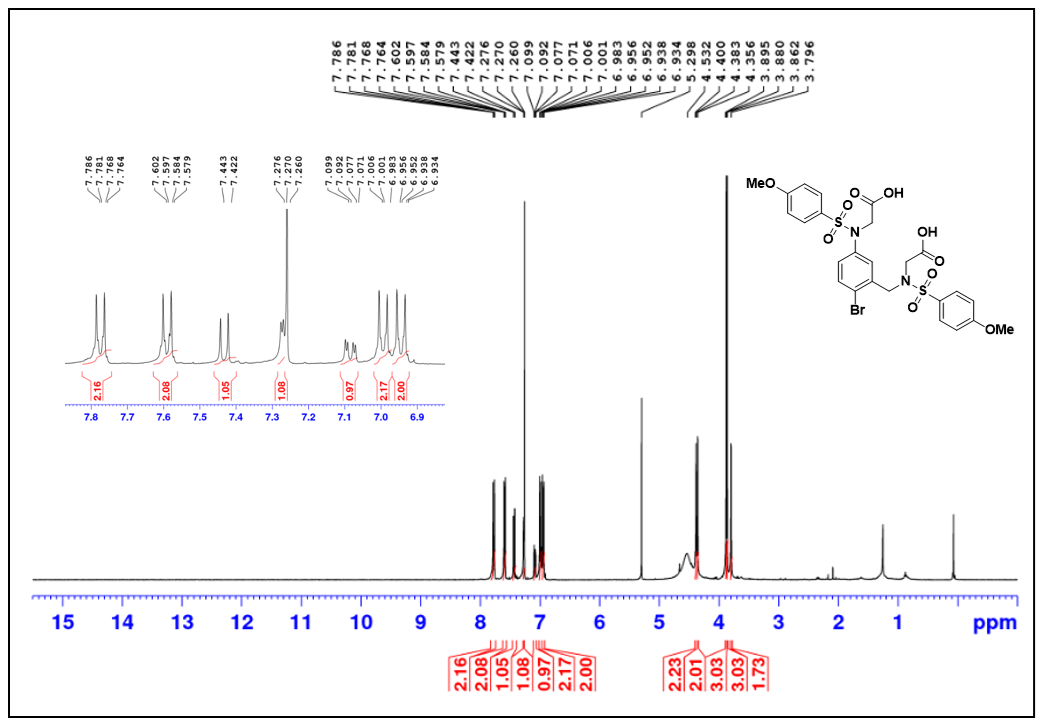
***

*^13^C-NMR of compound* ***7b***

***
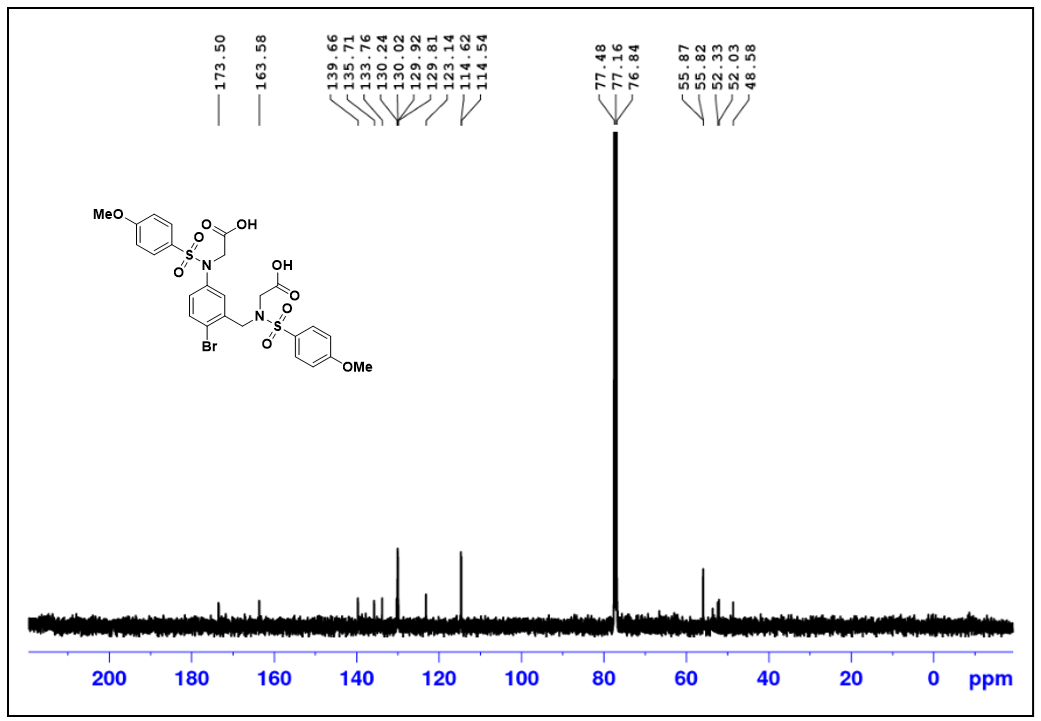
***

*^1^H-NMR of compound* ***7c***

***
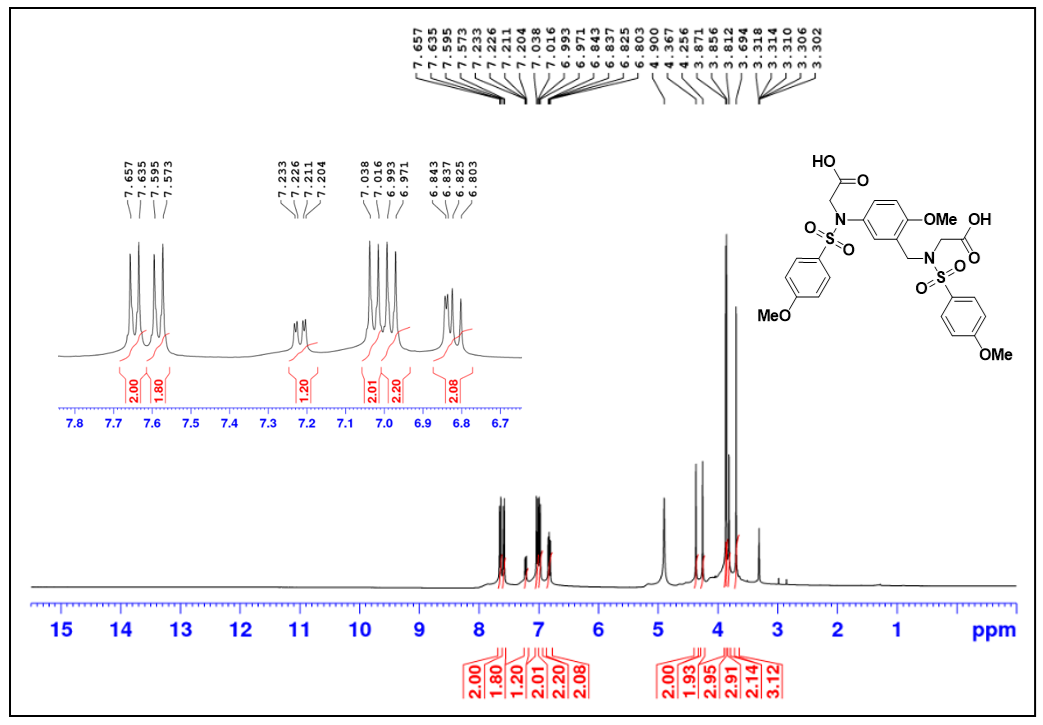
***

*^13^C-NMR of compound* ***7c***

***
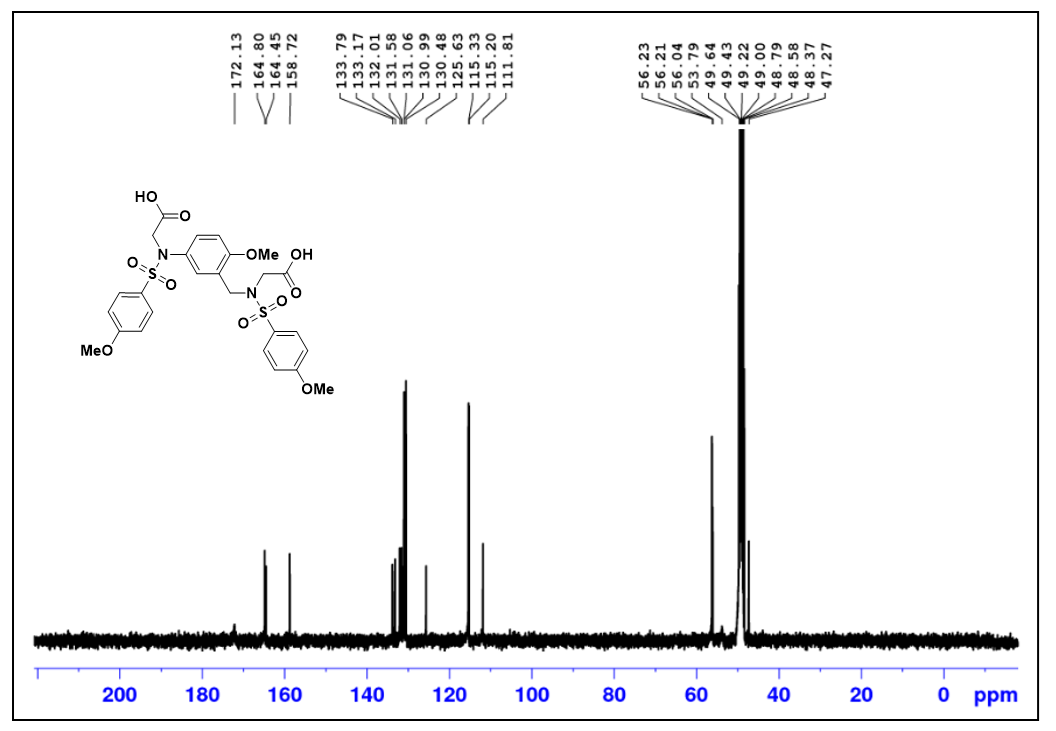
***

*^1^H-NMR of compound* ***7d***

***
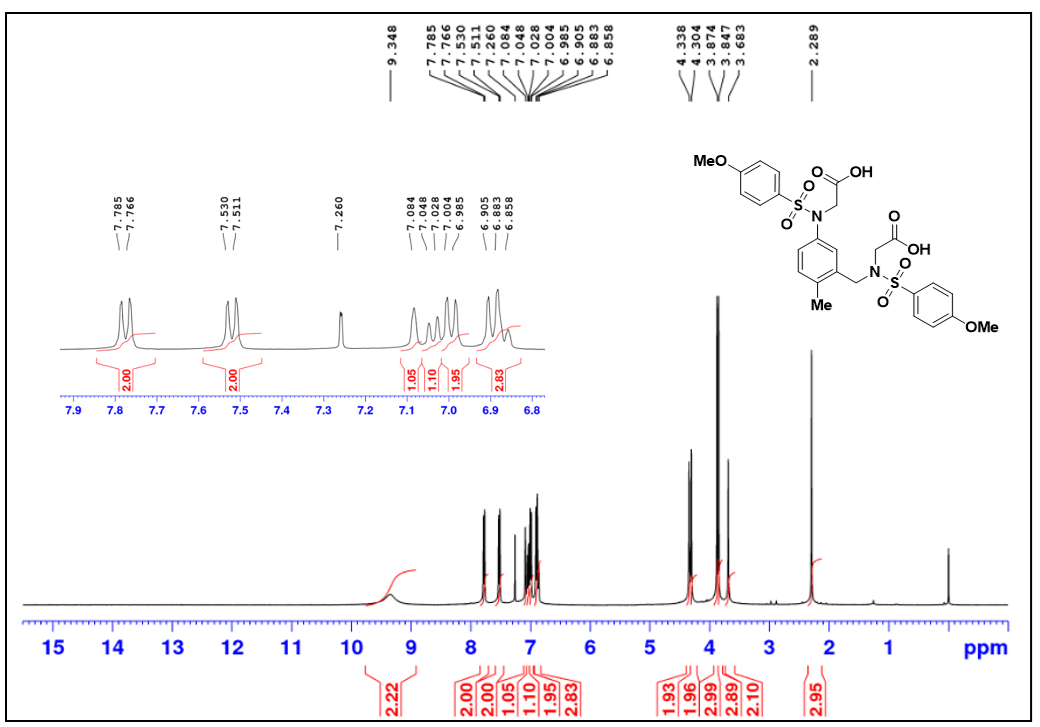
***

*^13^C-NMR of compound* ***7d***

***
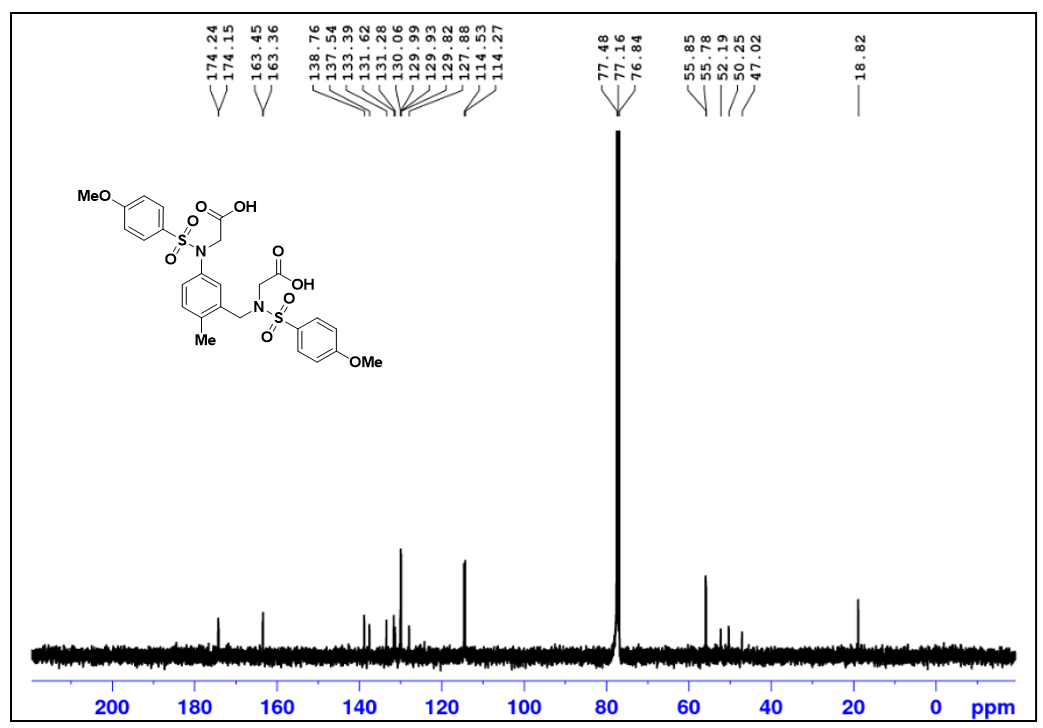
***

*^1^H-NMR of compound* ***7e***

***
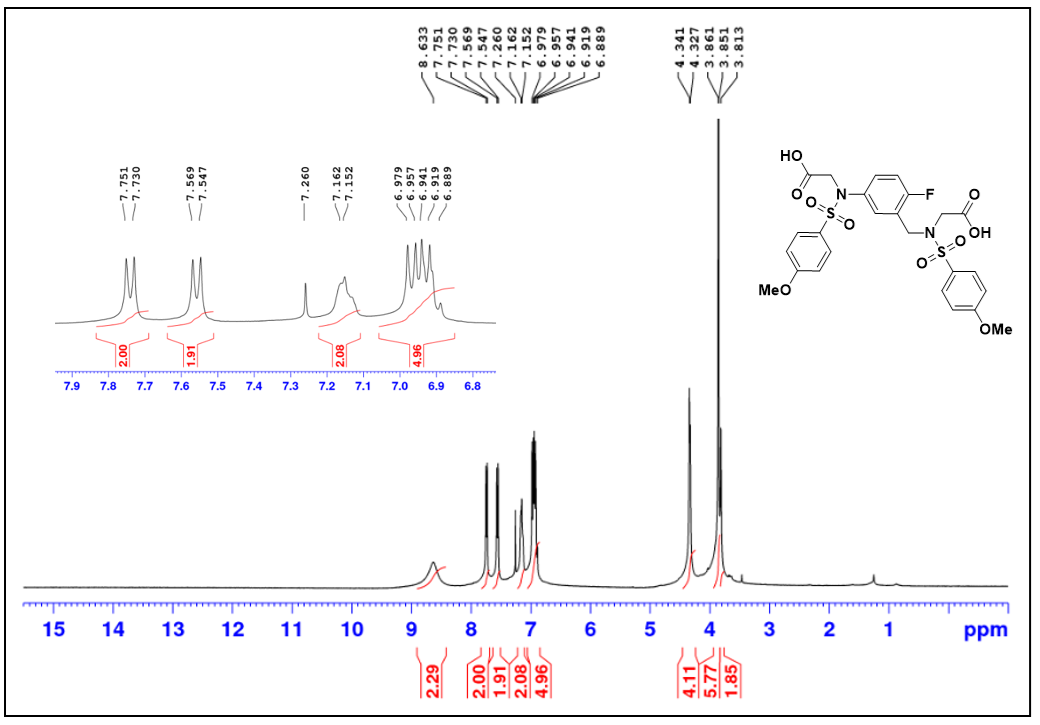
***

*^13^C-NMR of compound* ***7e***

***
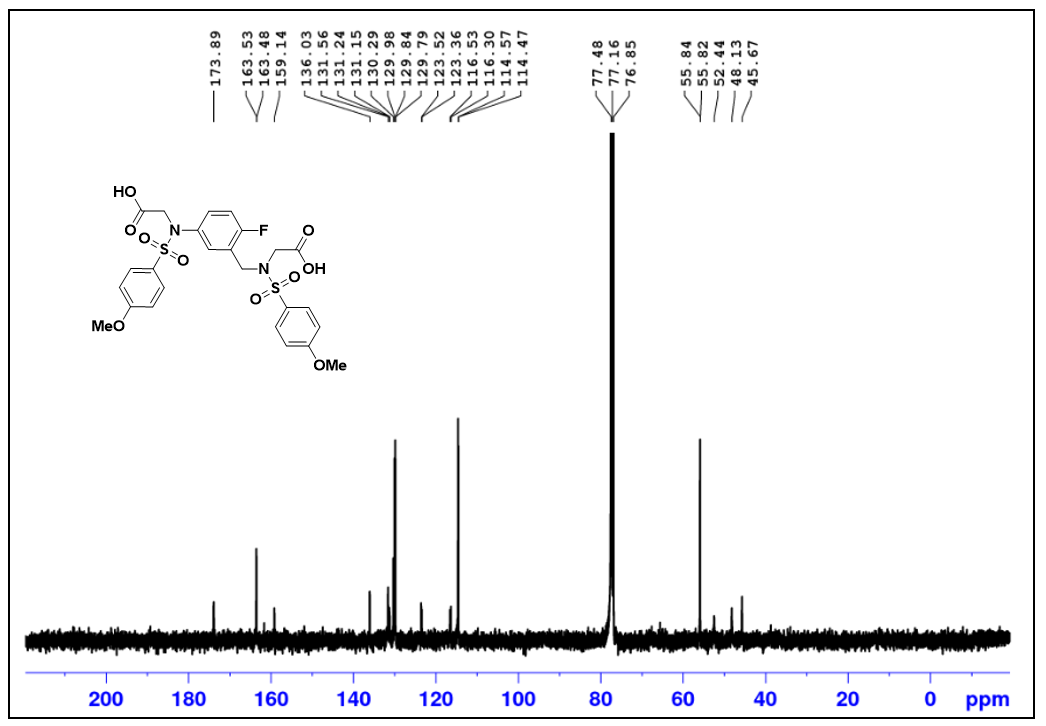
***

*^1^H-NMR of compound* ***7f***

***
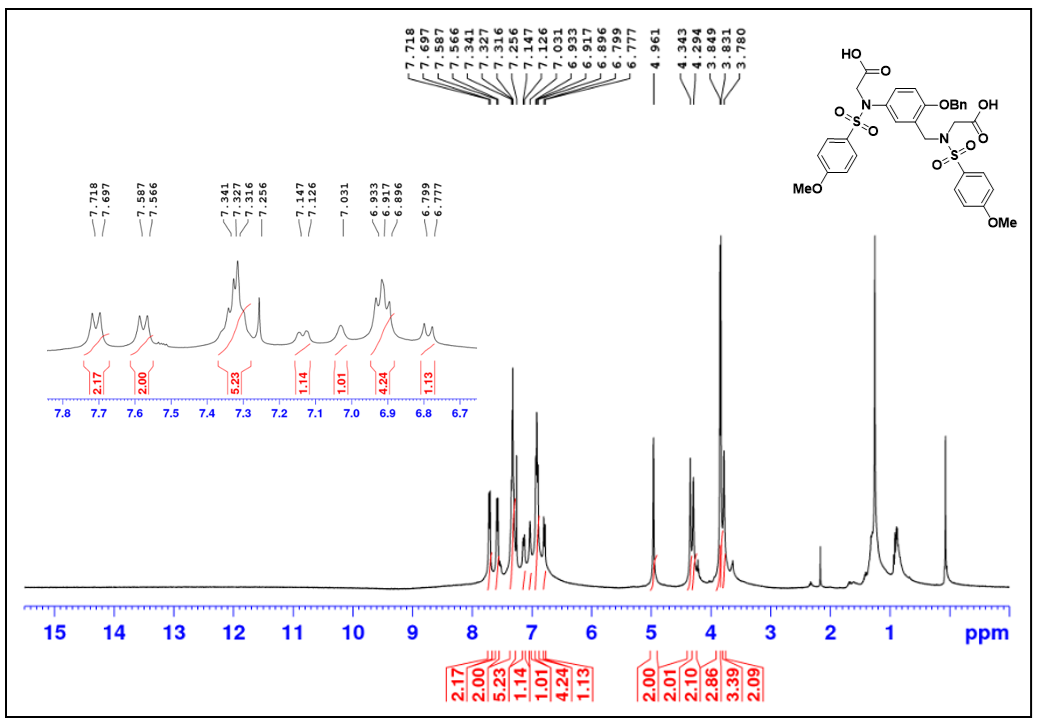
***

*^13^C-NMR of compound* ***7f***

***
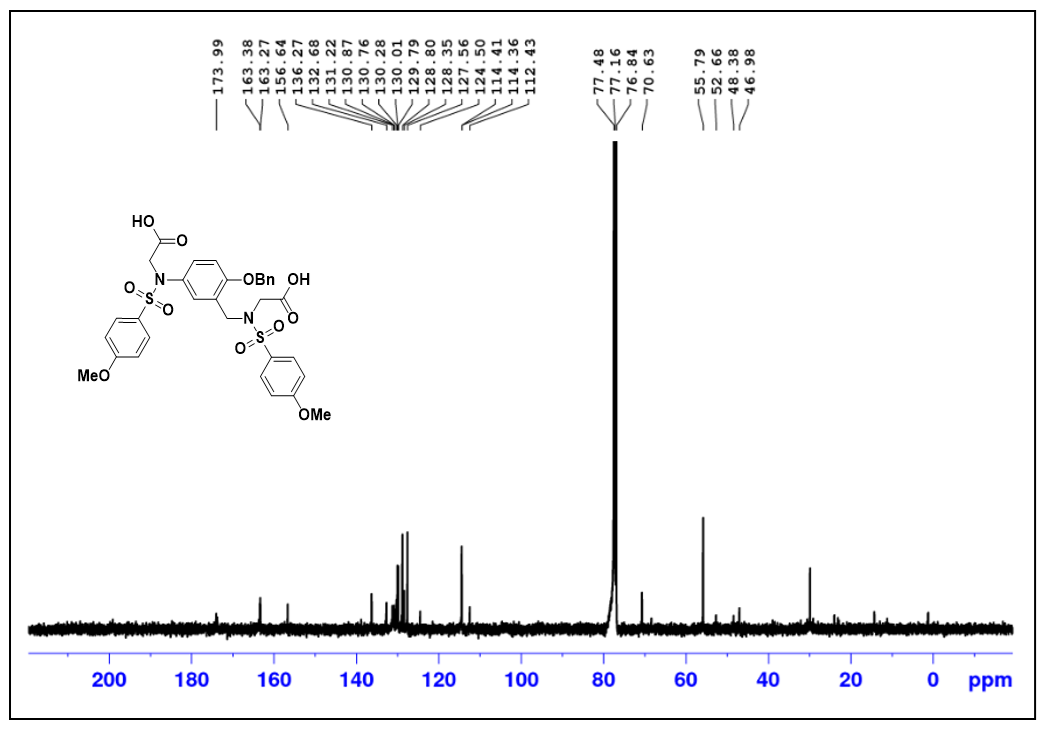
***

*^1^H-NMR of compound* ***8***

***
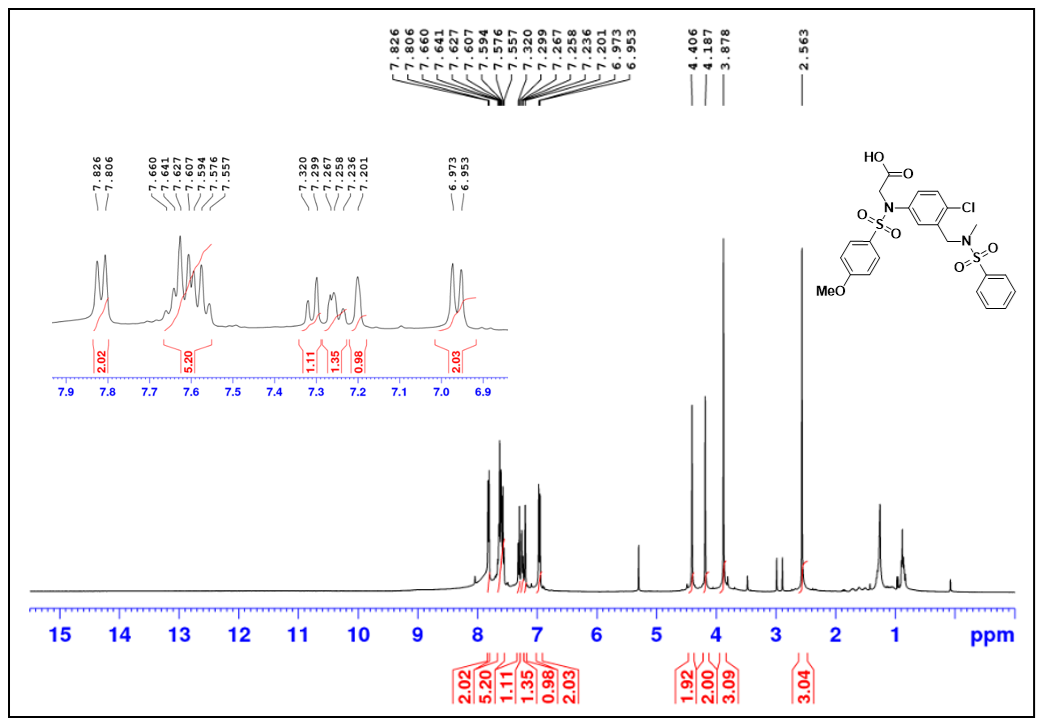
***

*^13^C-NMR of compound* ***8***

***
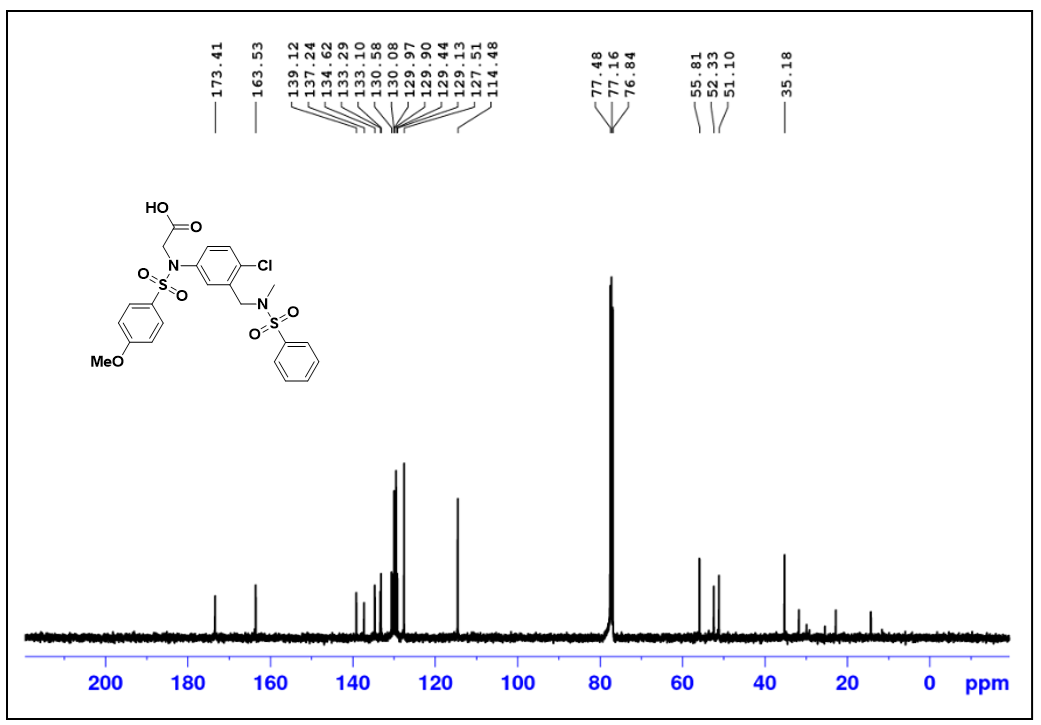
***

*^1^H-NMR of compound* ***9***

***
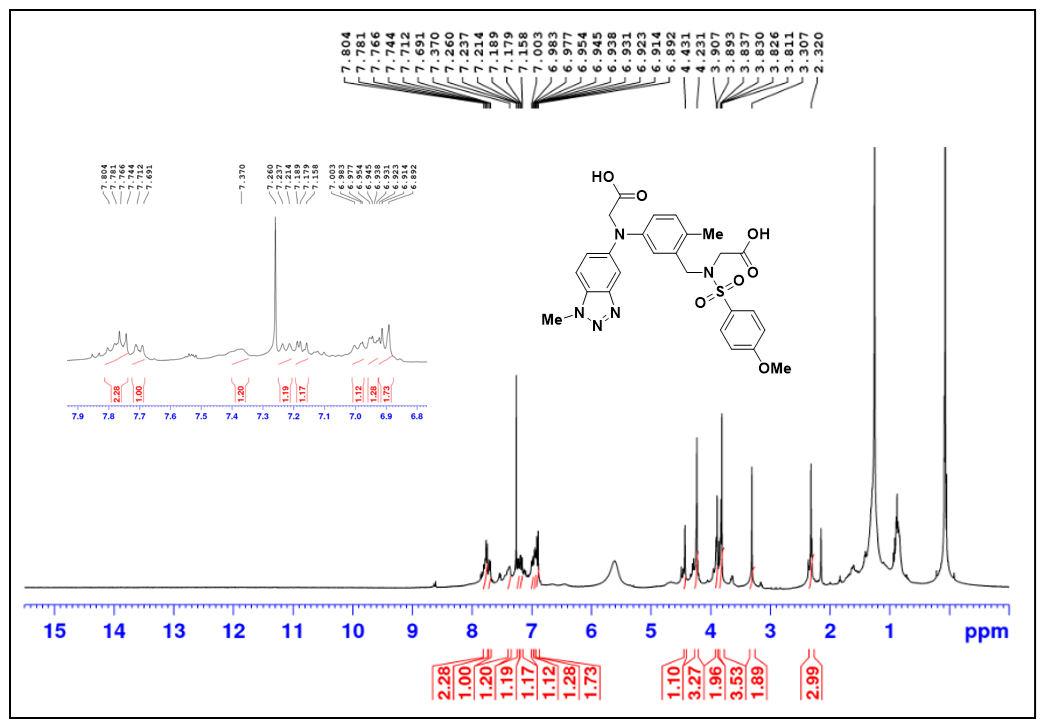
***

*^13^C-NMR of compound* ***9***

***
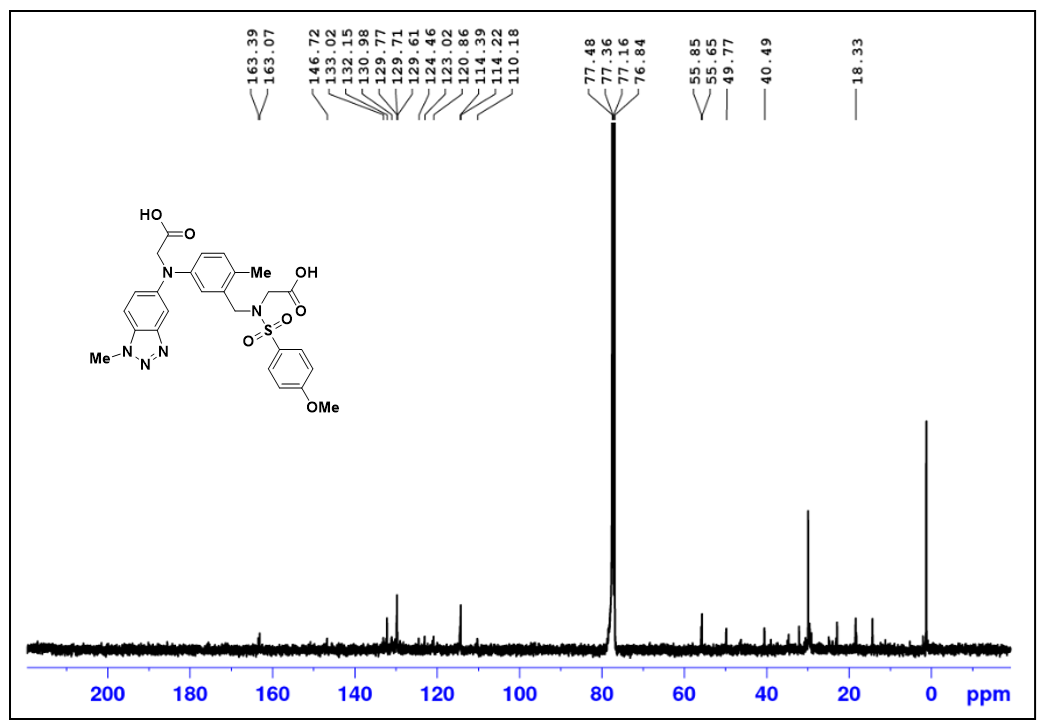
***

*^1^H-NMR of compound* ***10***

***
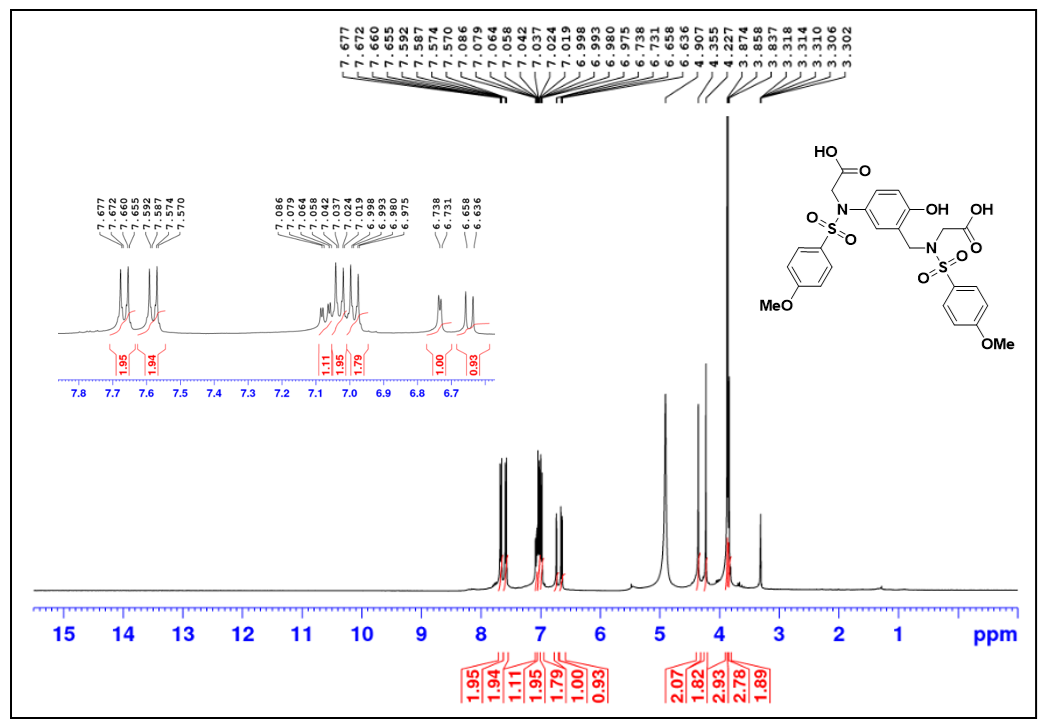
***

*^13^C-NMR of compound* ***10***

***
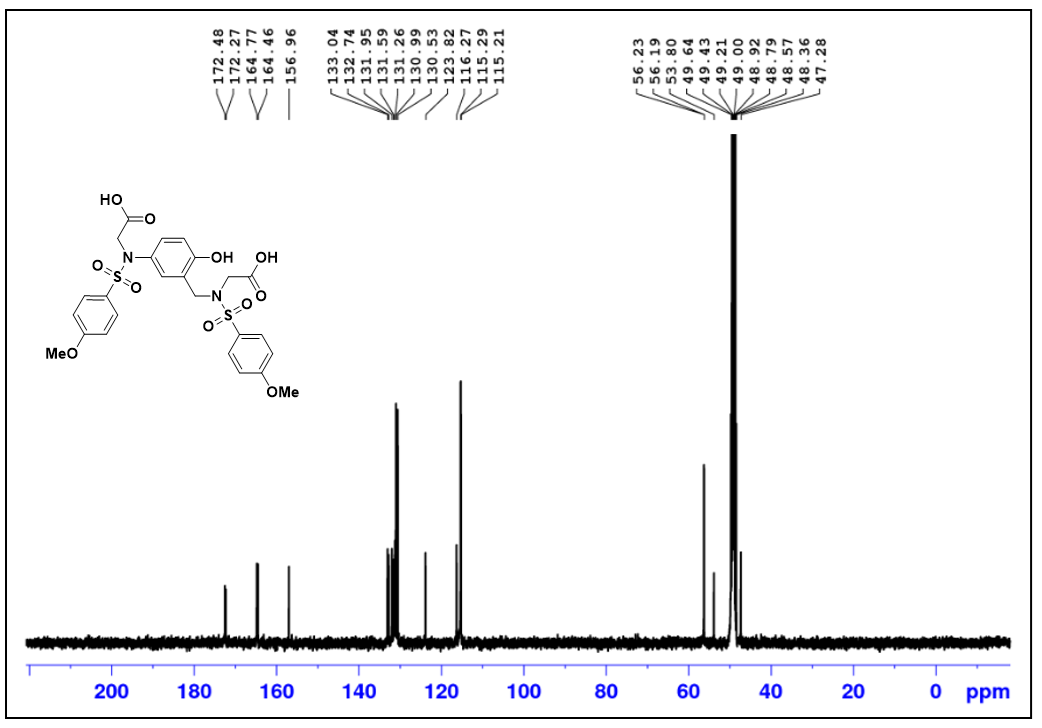
***

*^1^H-NMR of compound* ***11a***

***
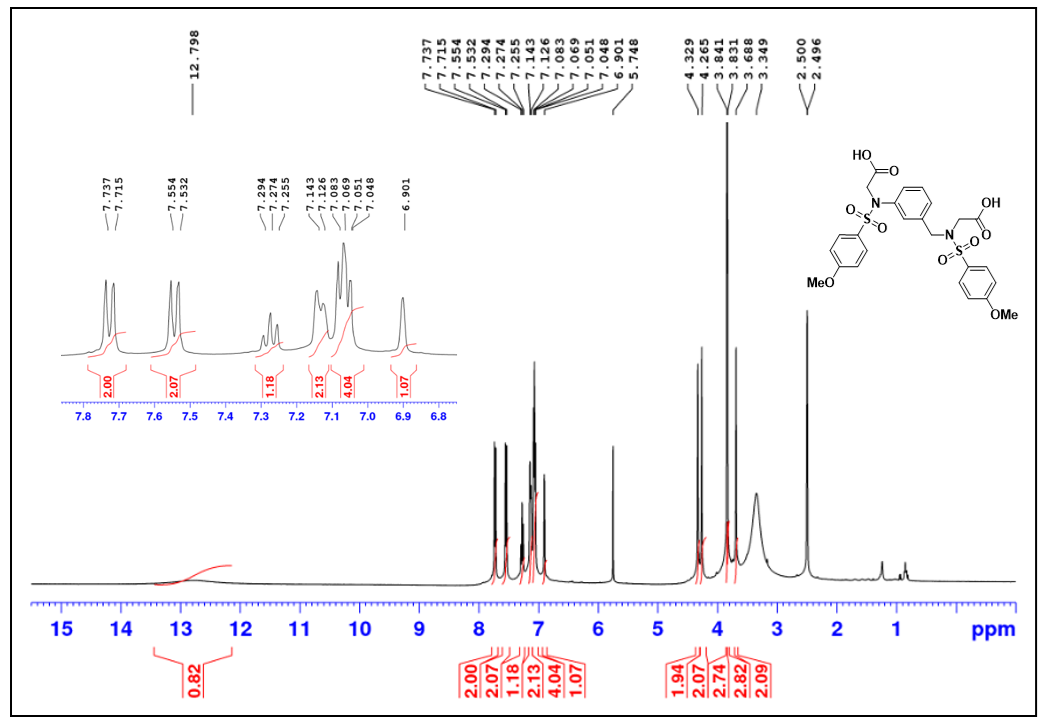
***

*^13^C-NMR of compound* ***11a***

***
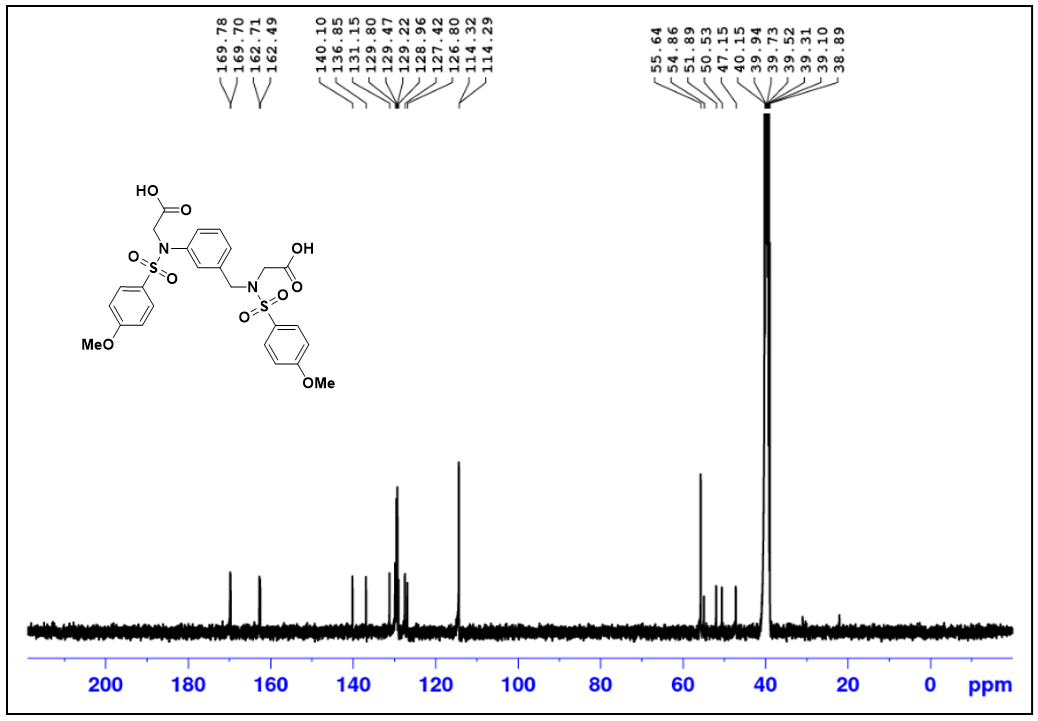
***

*^1^H-NMR of compound* ***11b***

***
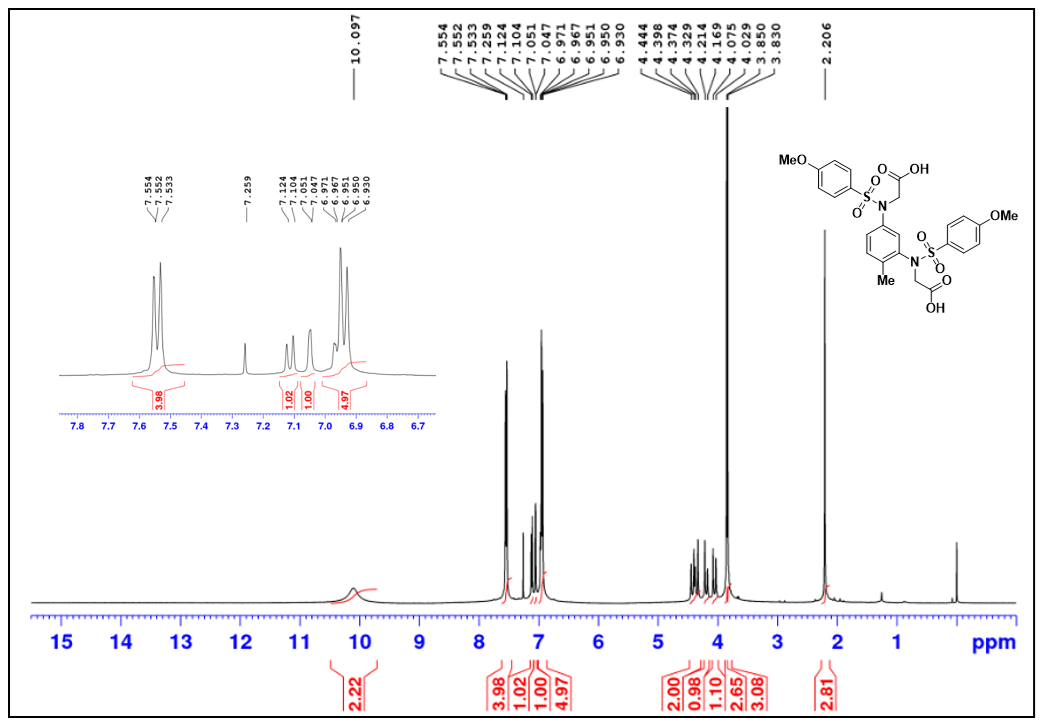
***

*^13^C-NMR of compound* ***11b***

***
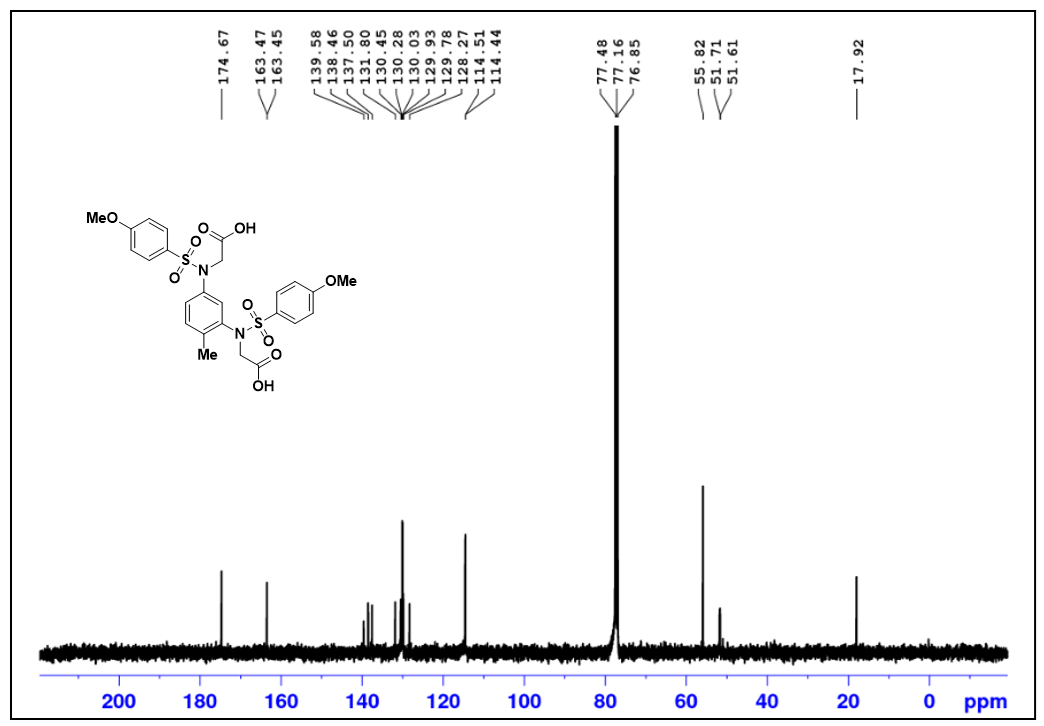
***

*^1^H-NMR of compound* ***12a***

***
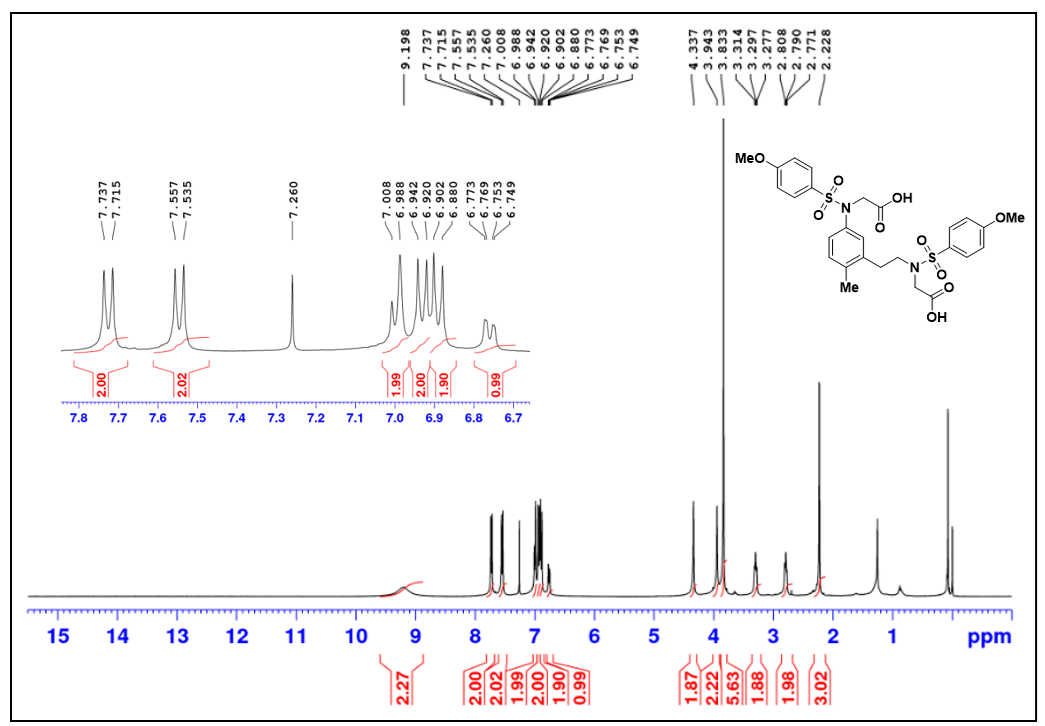
***

*^13^C-NMR of compound* ***12a***

***
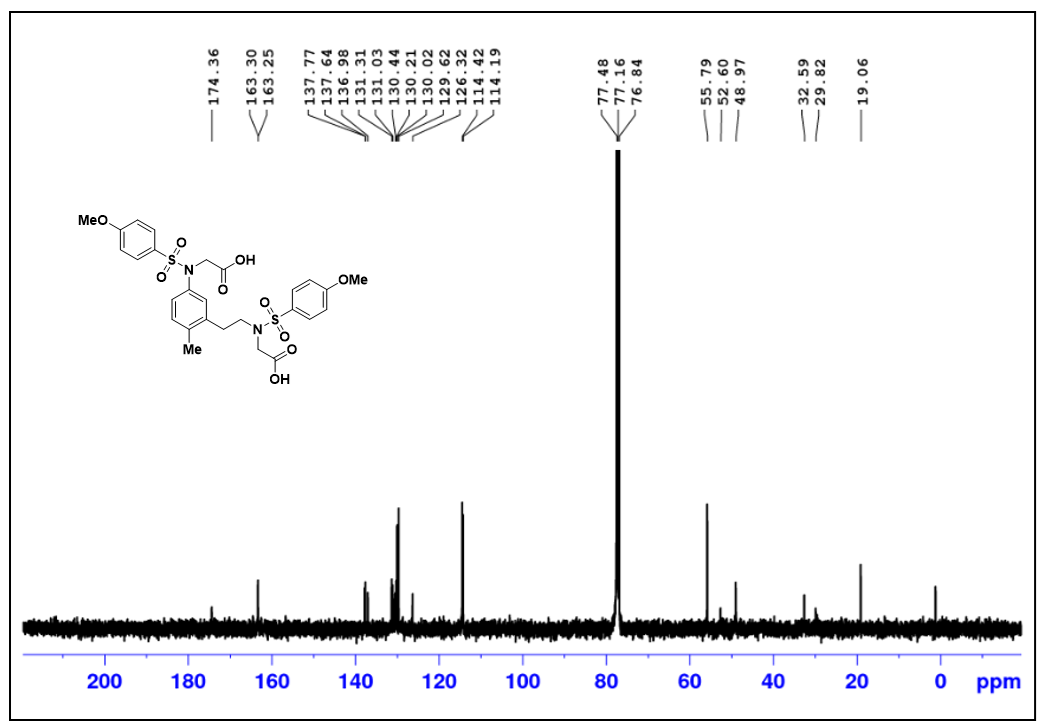
***

*^1^H-NMR of compound* ***12b***

***
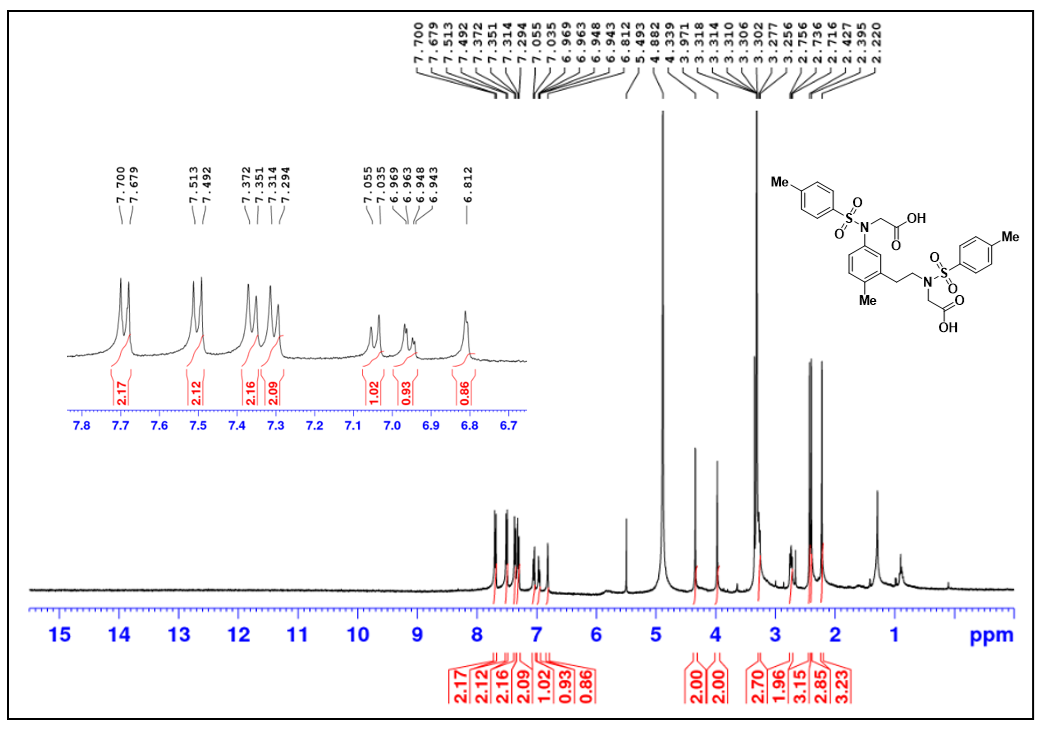
***

*^13^C-NMR of compound* ***12b***

***
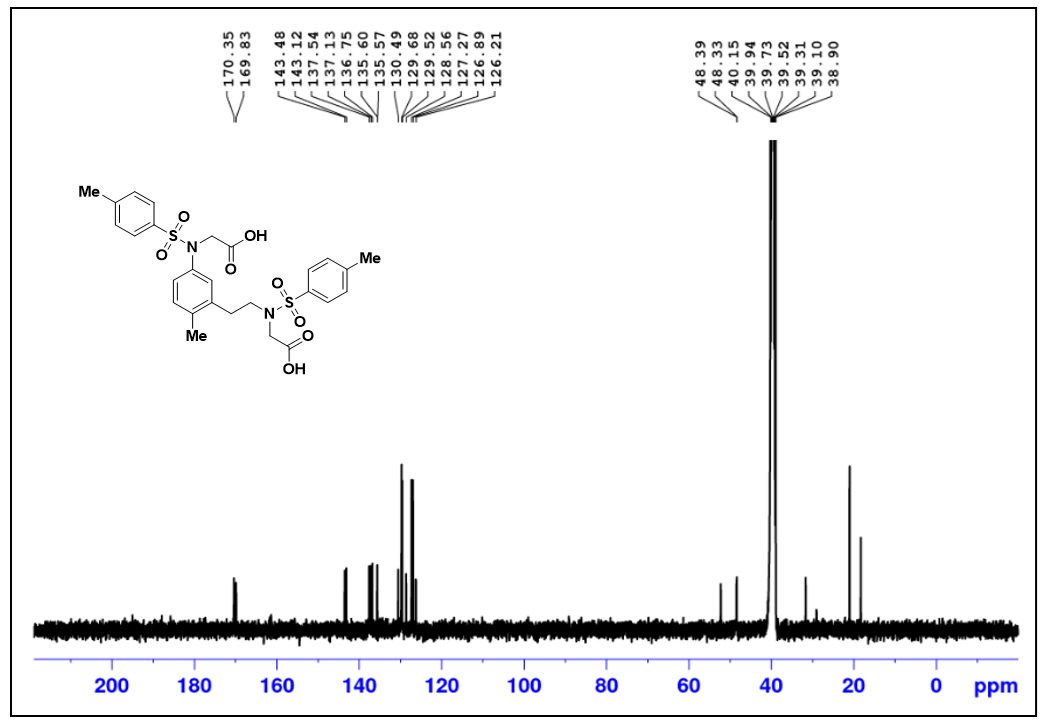
***

*^1^H-NMR of compound* ***12c***

***
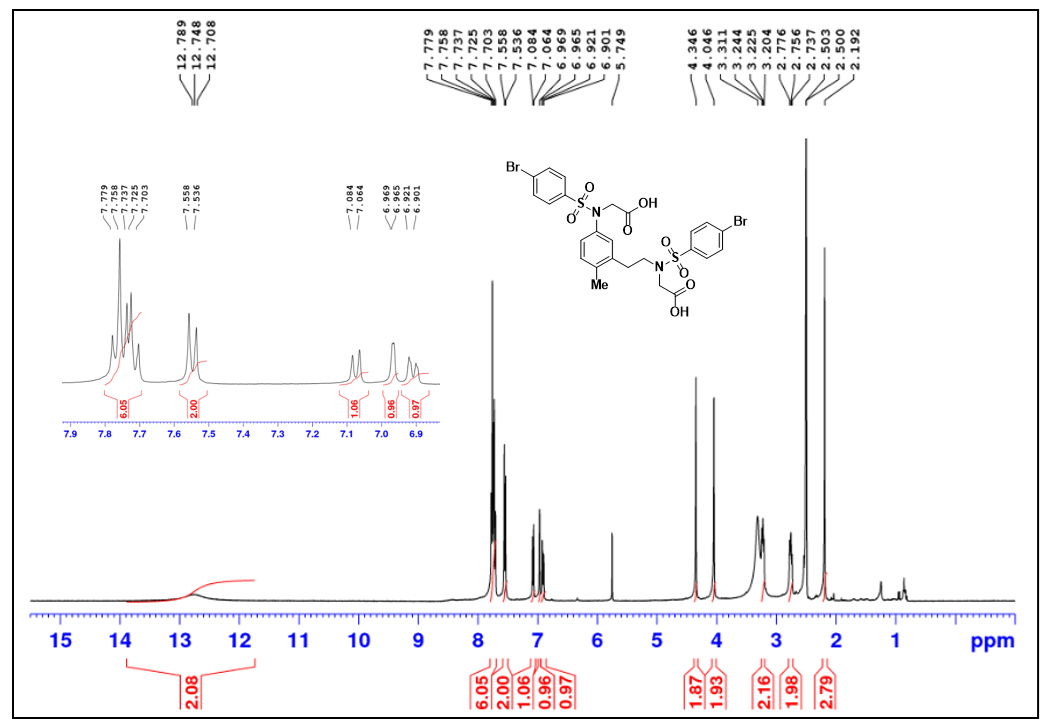
***

*^13^C-NMR of compound* ***12c***

***
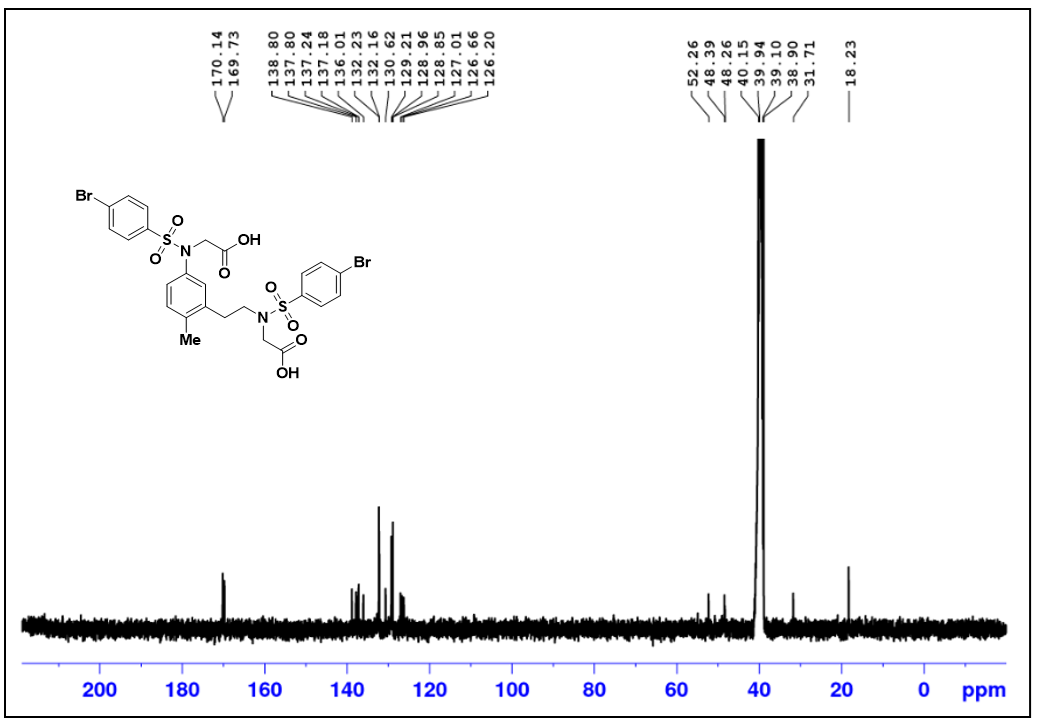
***

*^1^H-NMR of compound* ***12d***

***
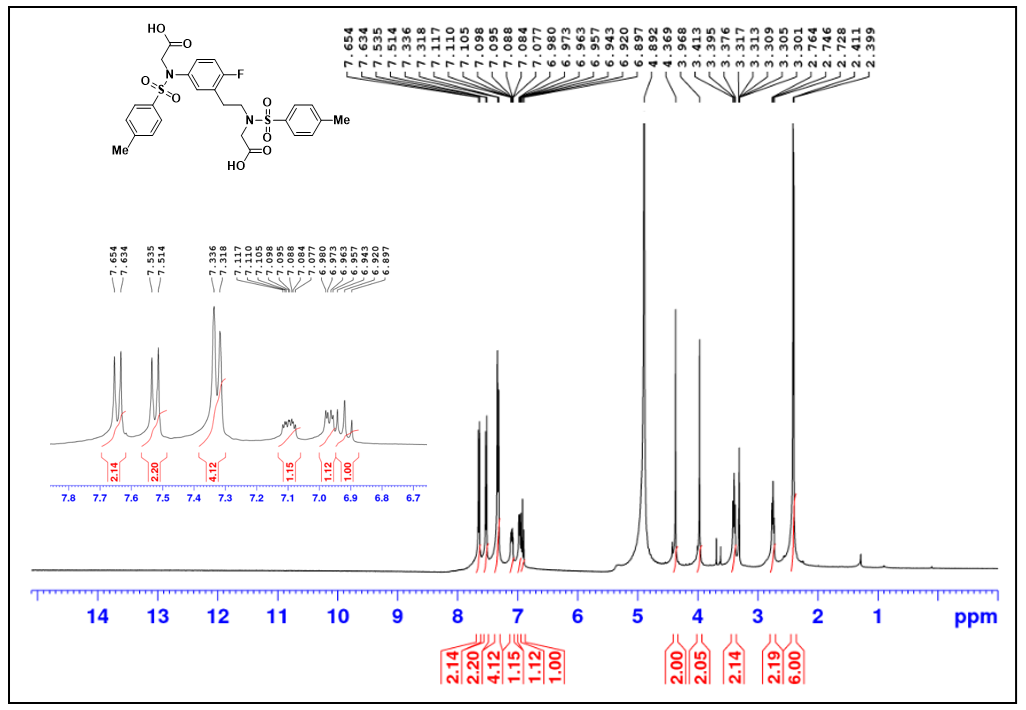
***

*^13^C-NMR of compound* ***12d***

***
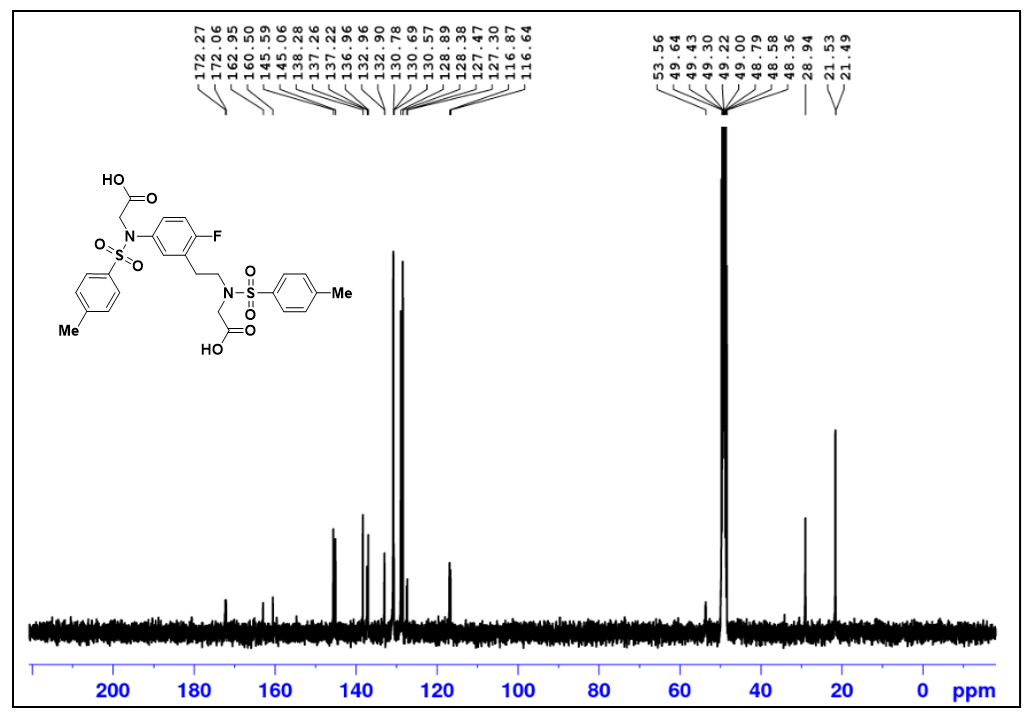
***

*^1^H-NMR of compound* ***13a***

***
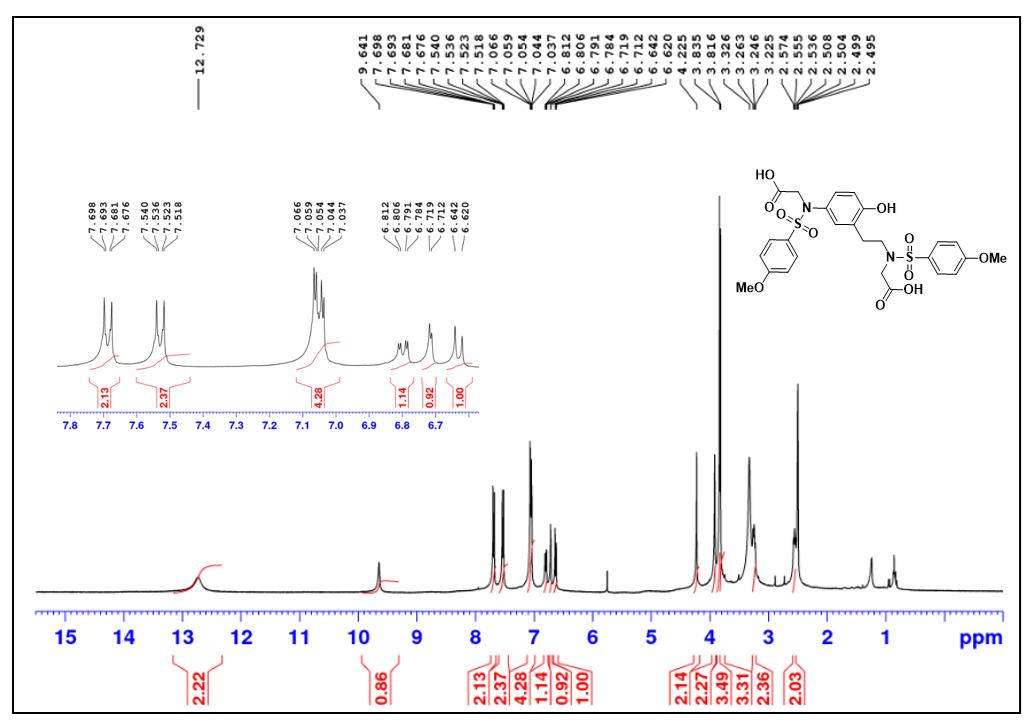
***

*^13^C-NMR of compound* ***13a***

***
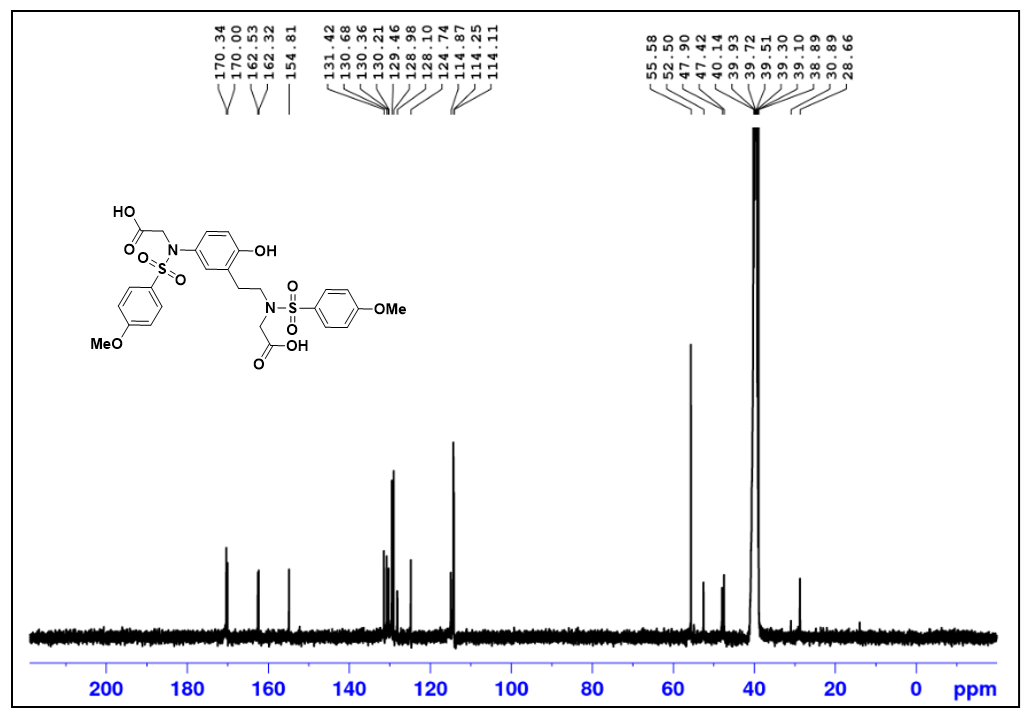
***

*^1^H-NMR of compound* ***13b***

***
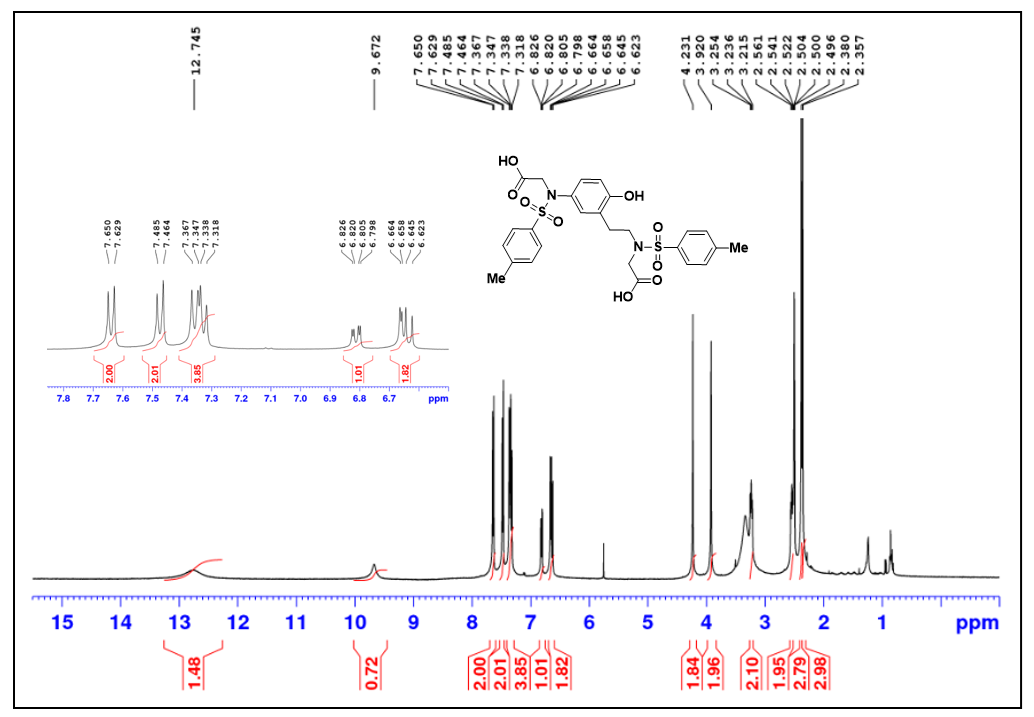
***

*^13^C-NMR of compound* ***13b***

***
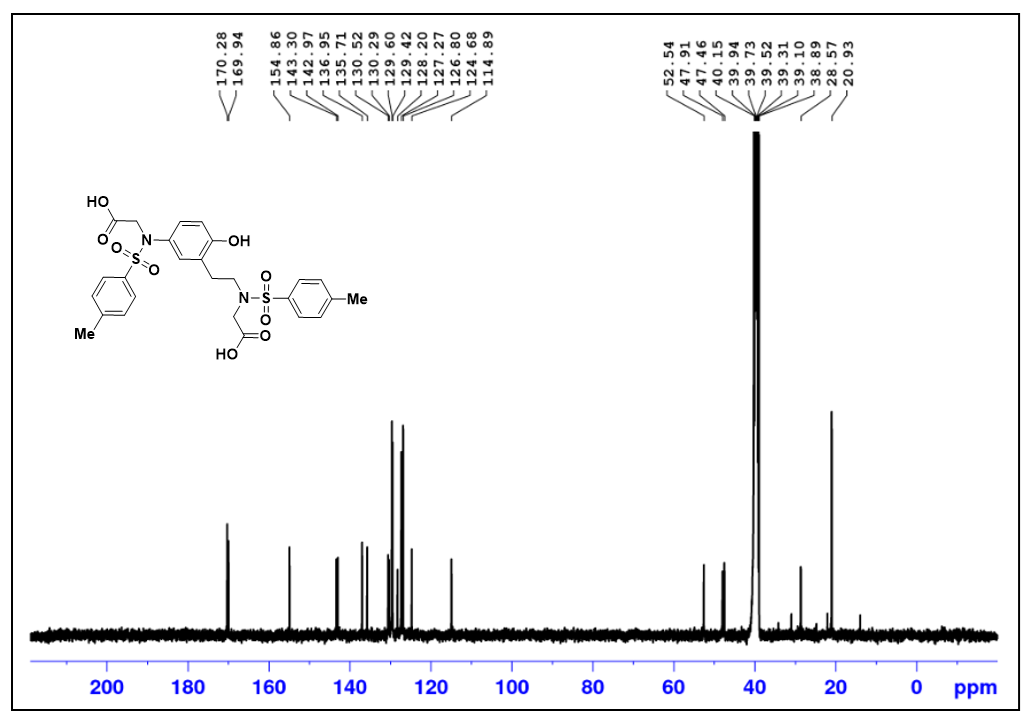
***

*^1^H-NMR of compound* ***13c***

***
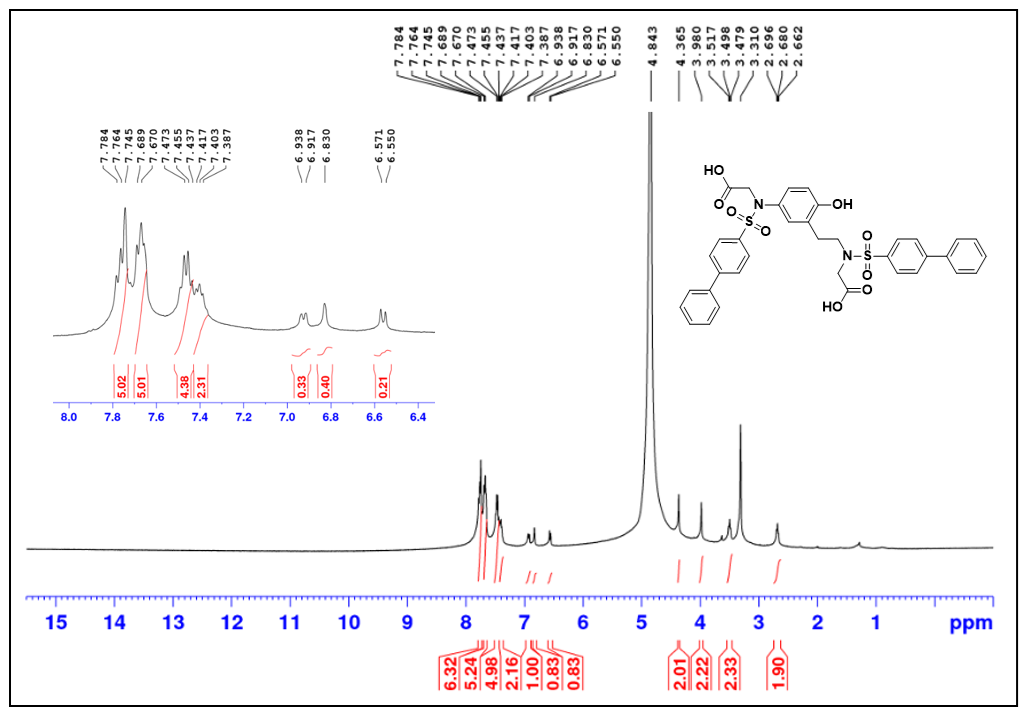
***

*^13^C-NMR of compound* ***13c***

***
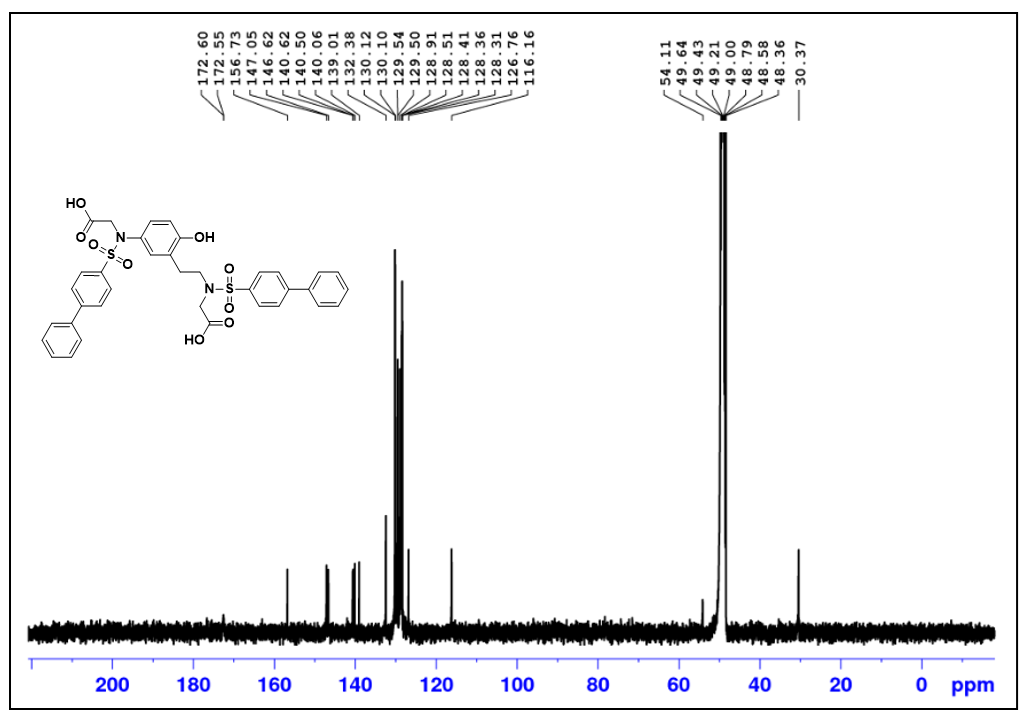
***

*^1^H-NMR of compound* ***14***

***
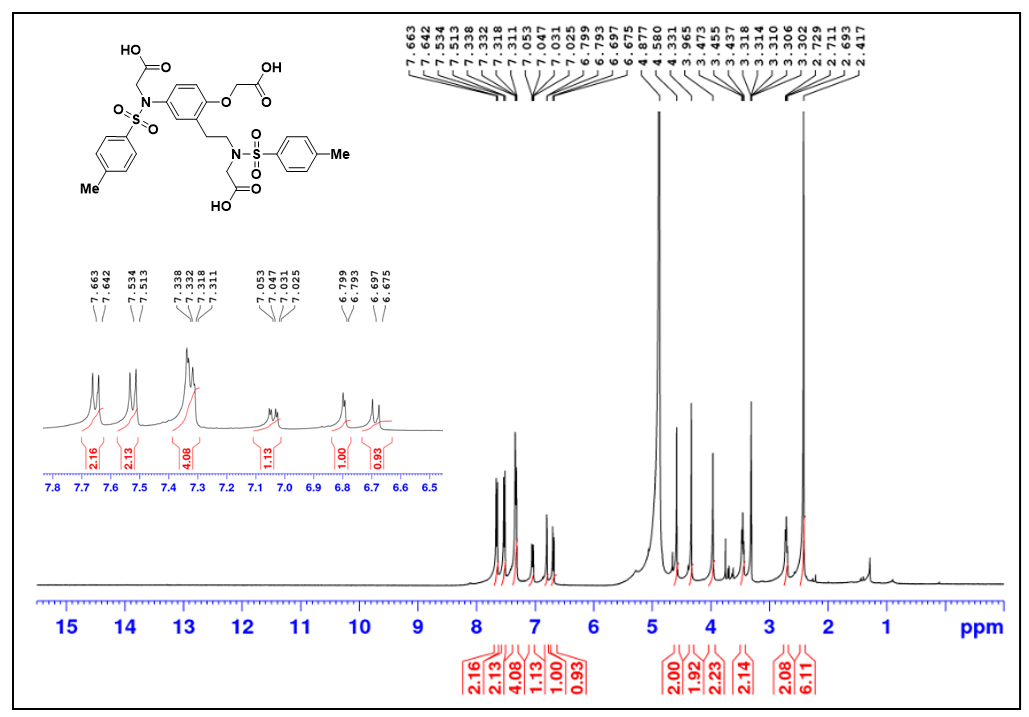
***

*^13^C-NMR of compound* ***14***

***
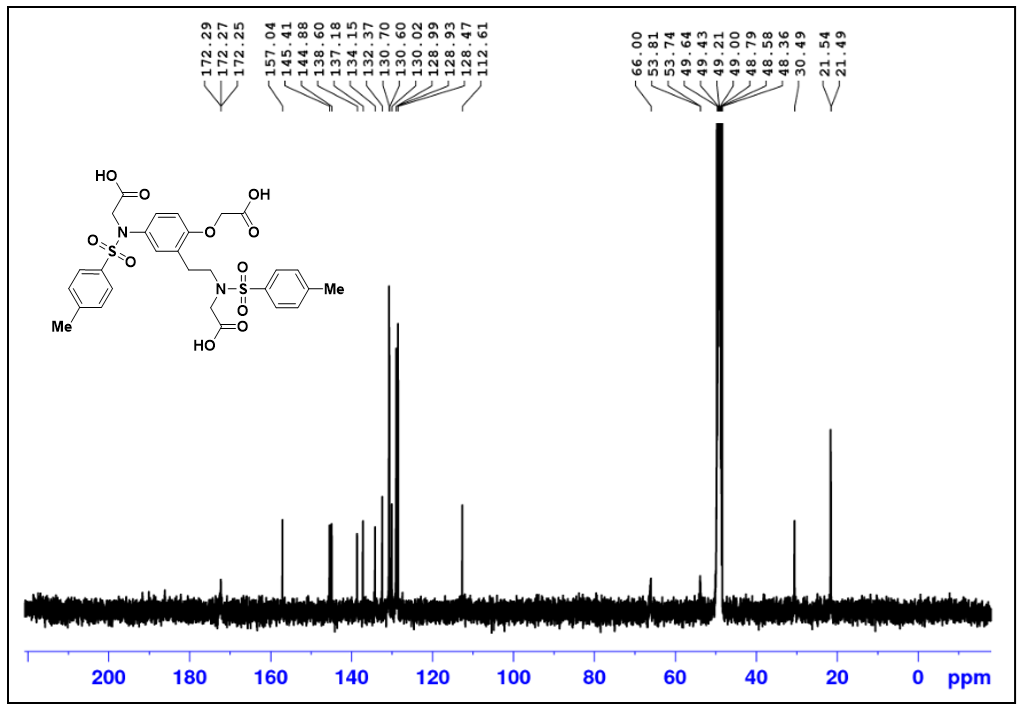
***

*^1^H-NMR of compound* ***15a***

***
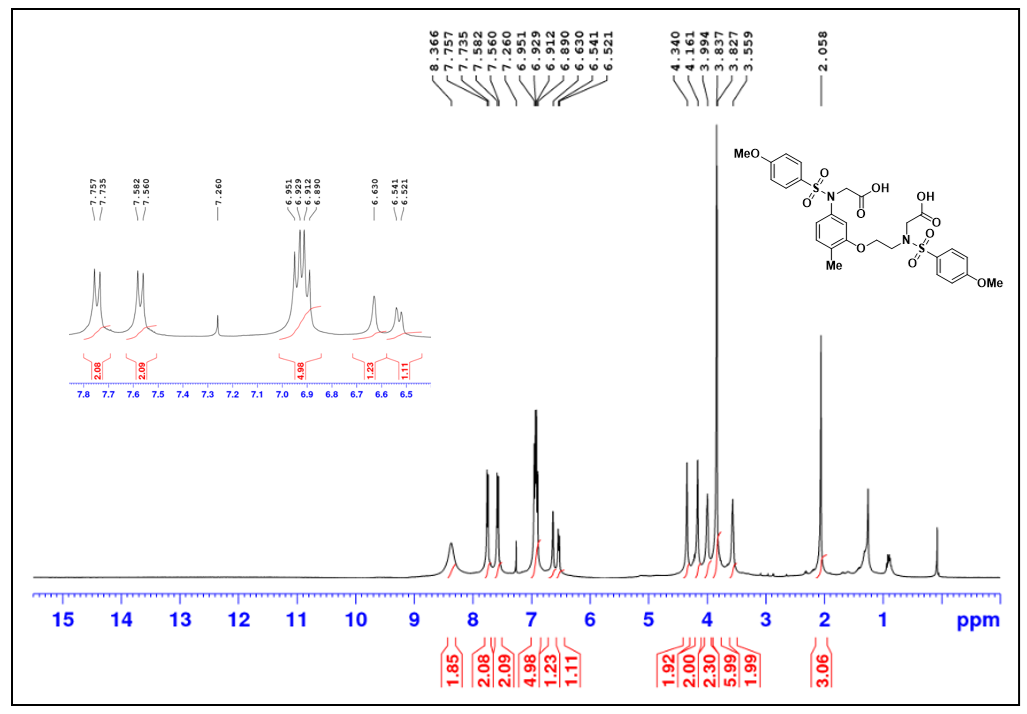
***

*^13^C-NMR of compound* ***15a***

***
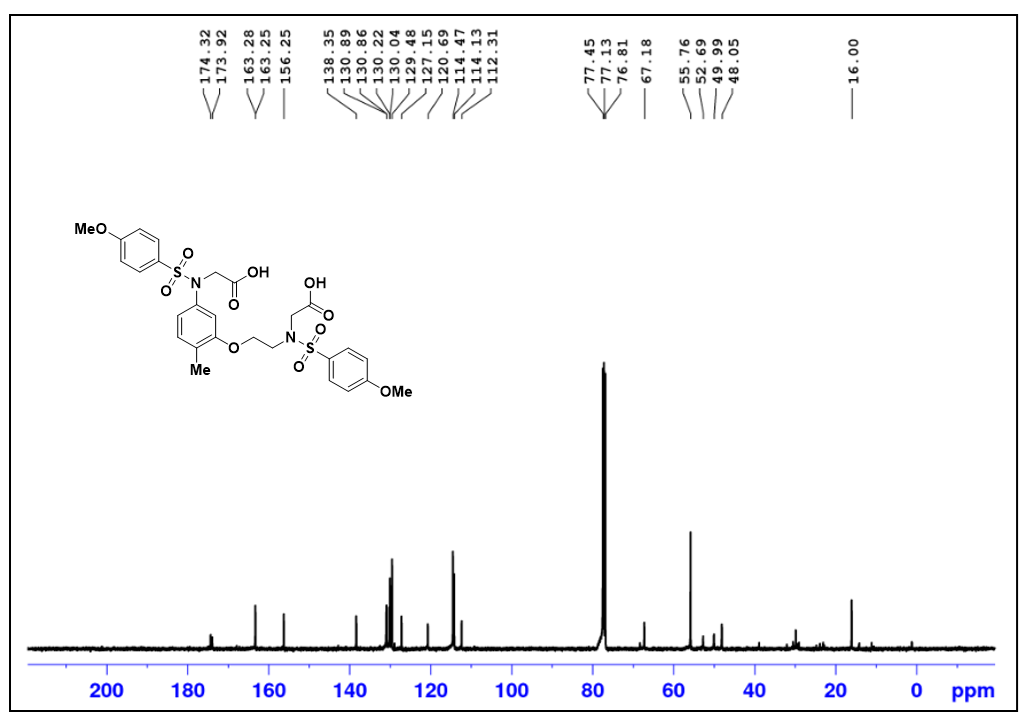
***

*^1^H-NMR of compound* ***15b***

***
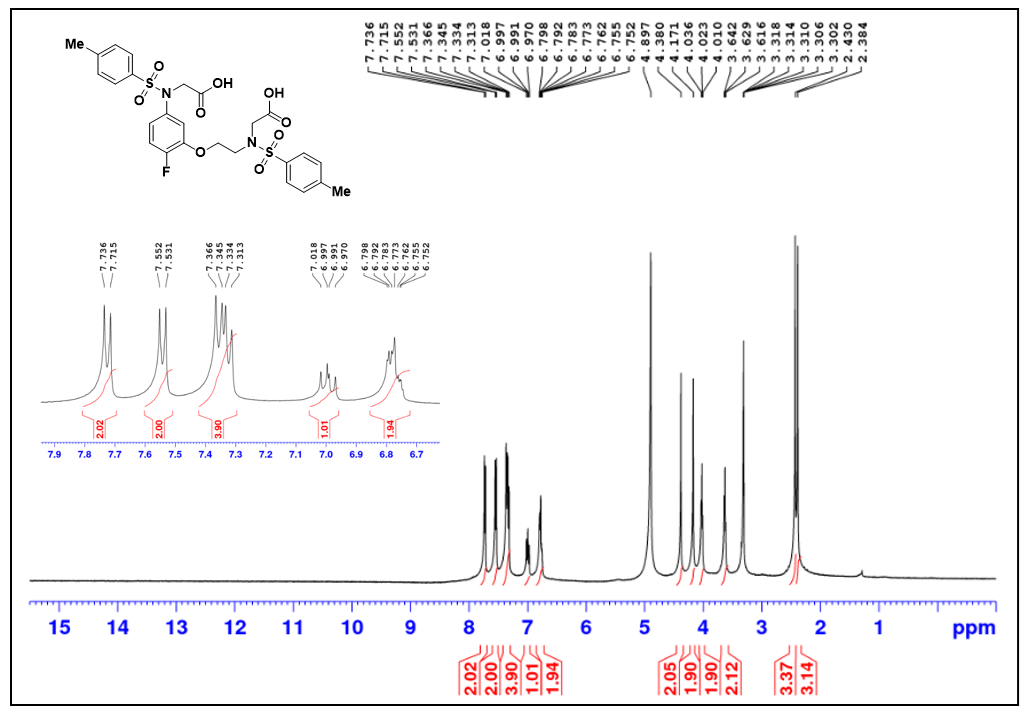
***

*^13^C-NMR of compound* ***15b***

***
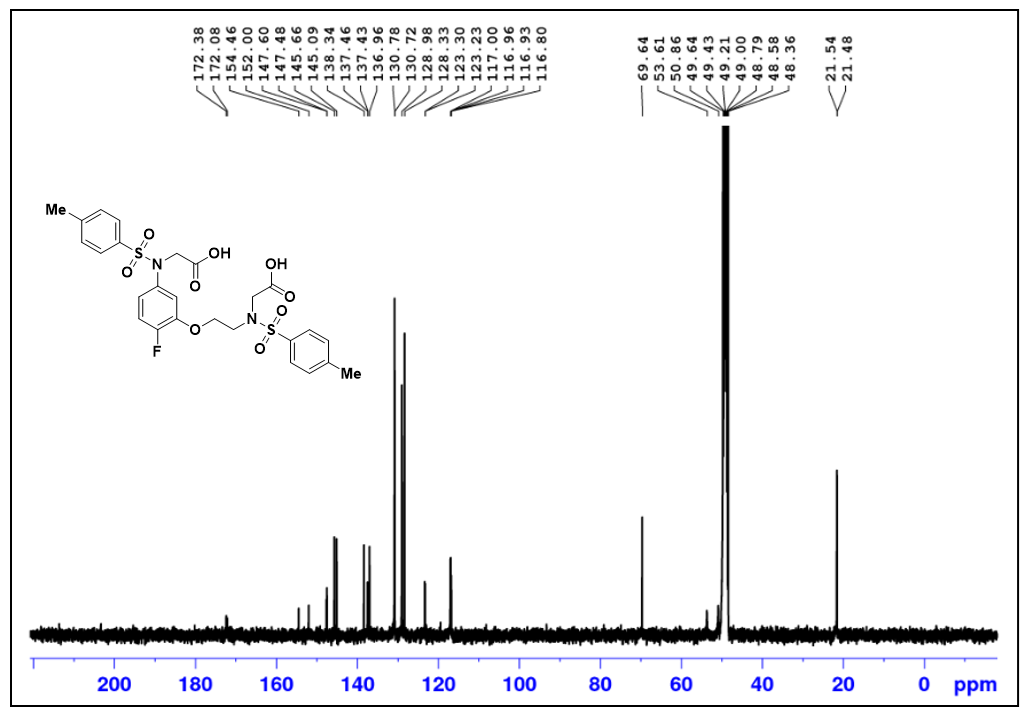
***

*^1^H-NMR of compound* ***16***

***
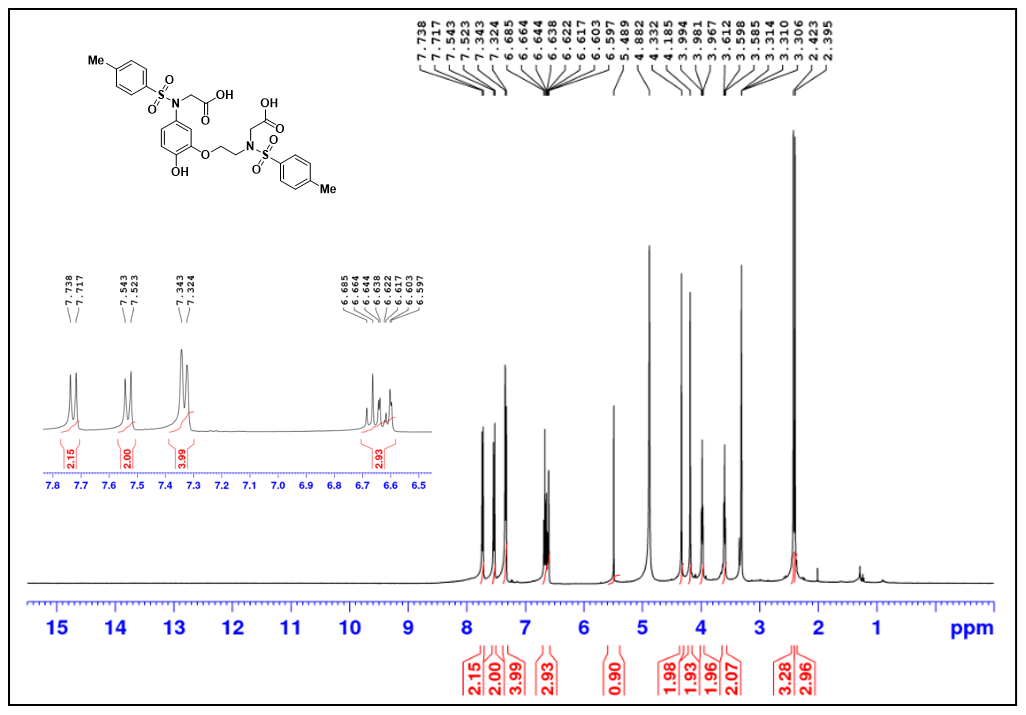
***

*^13^C-NMR of compound* ***16***

***
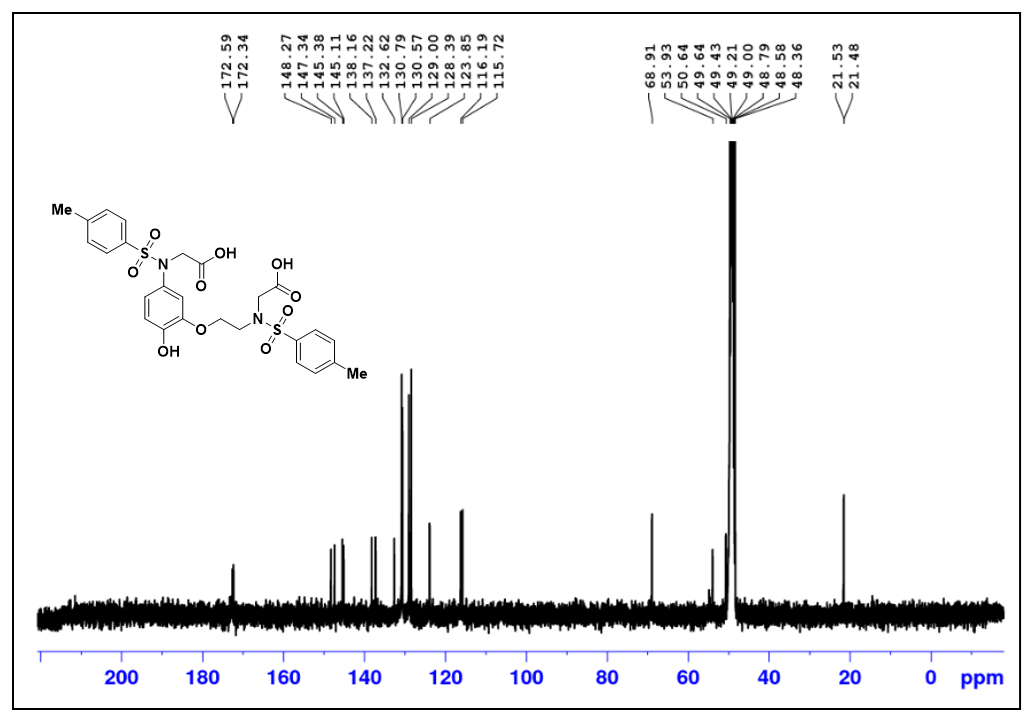
***

*^1^H-NMR of compound* ***17***

***
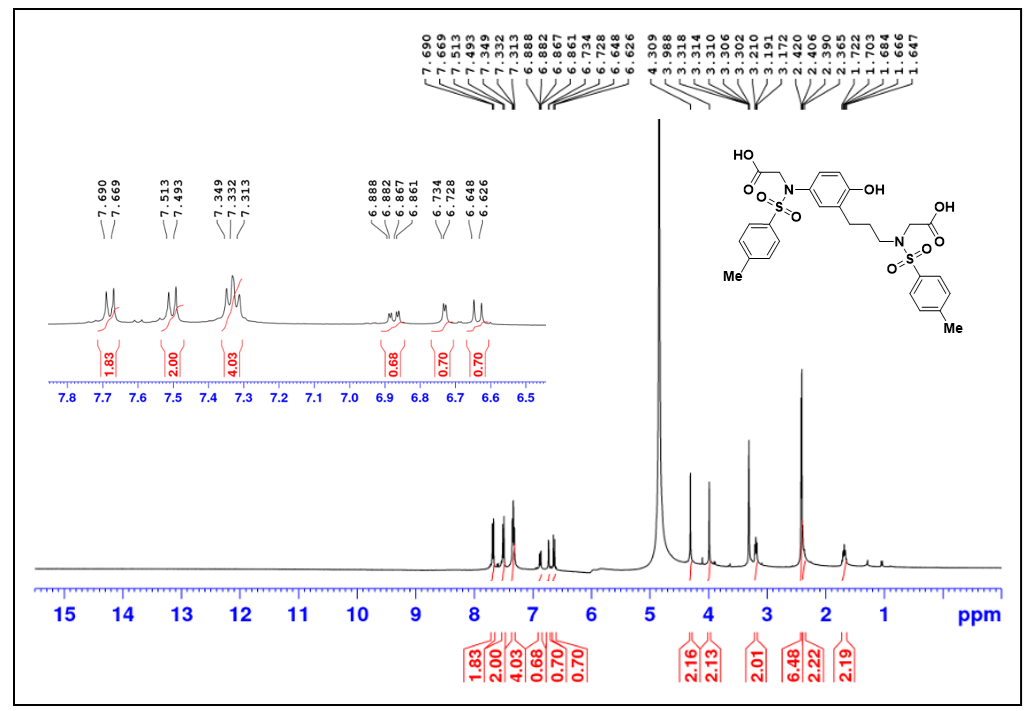
***

*^13^C-NMR of compound* ***17***

***
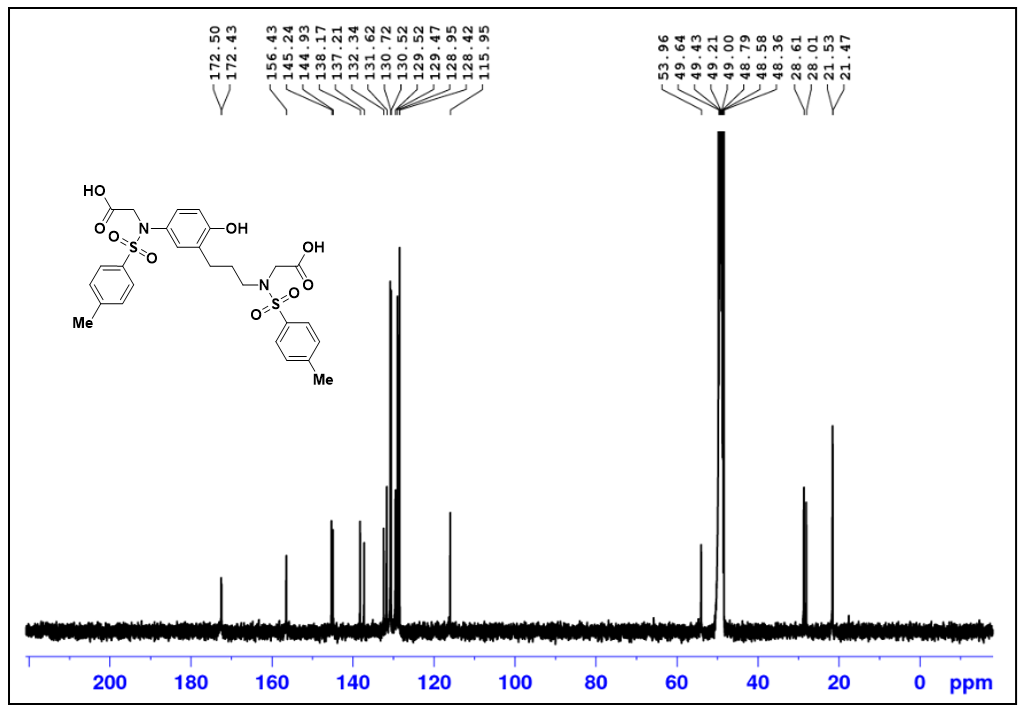
***

**5. UPLC analysis and purity spectra of final compounds**

To determine the purities of final compounds, UPLC analysis was performed using a Waters ACQUITY UPLC^TM^ system equipped with an ACQUITY UPLC HSS T3 column (100Å, 1.8 μm, 3 mm x 150 mm). A gradient elution was carried out from 10% water with 0.1% trifluoroacetic acid to 90% acetonitrile with 0.1% trifluoroacetic acid over 5.0 minutes at a flow rate of 0.6 mL/min. UV detection was performed at 254 or 280 nm.

*UPLC analysis of compound* ***7a***

***
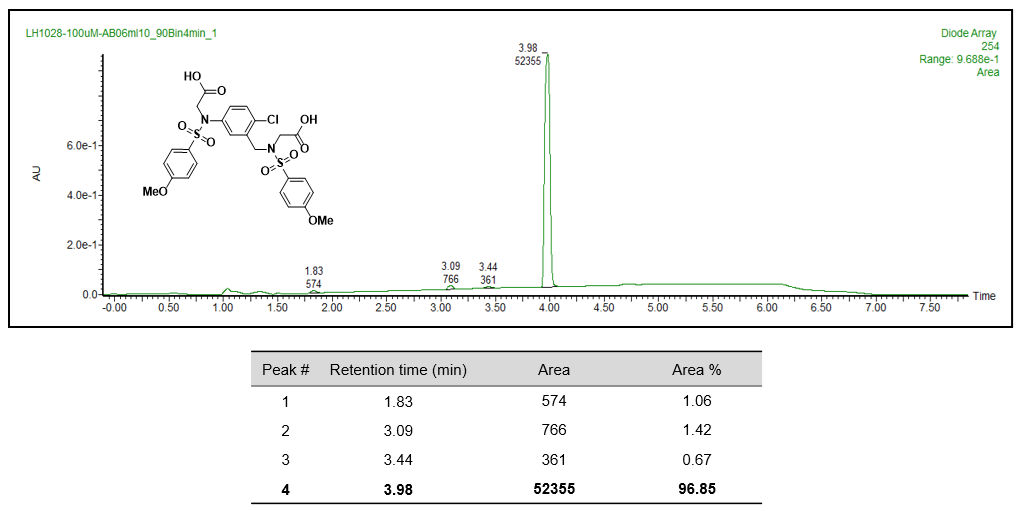
***

*UPLC analysis of compound* ***7b***

***
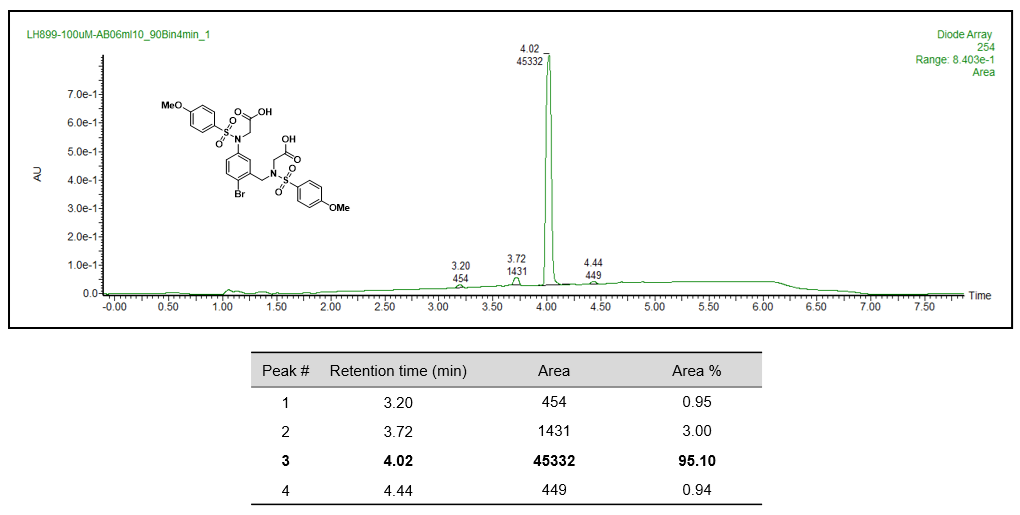
***

*UPLC analysis of compound* ***7c***

***
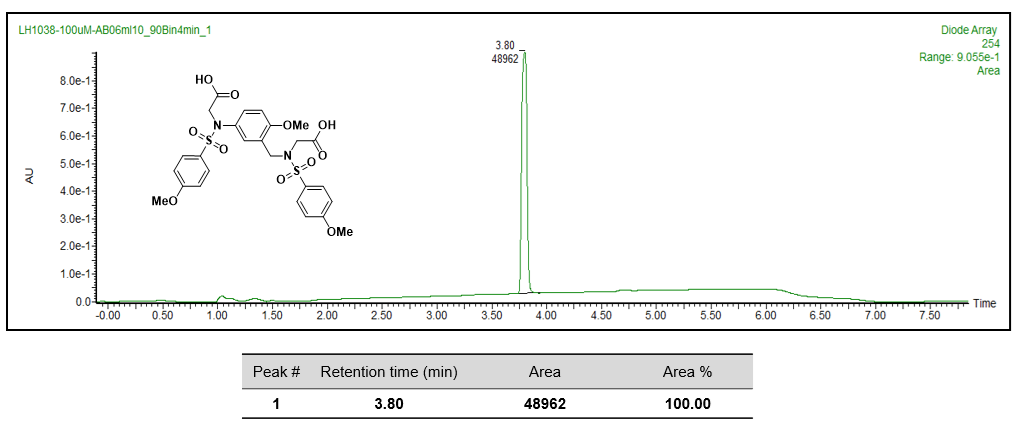
***

*UPLC analysis of compound* ***7d***

***
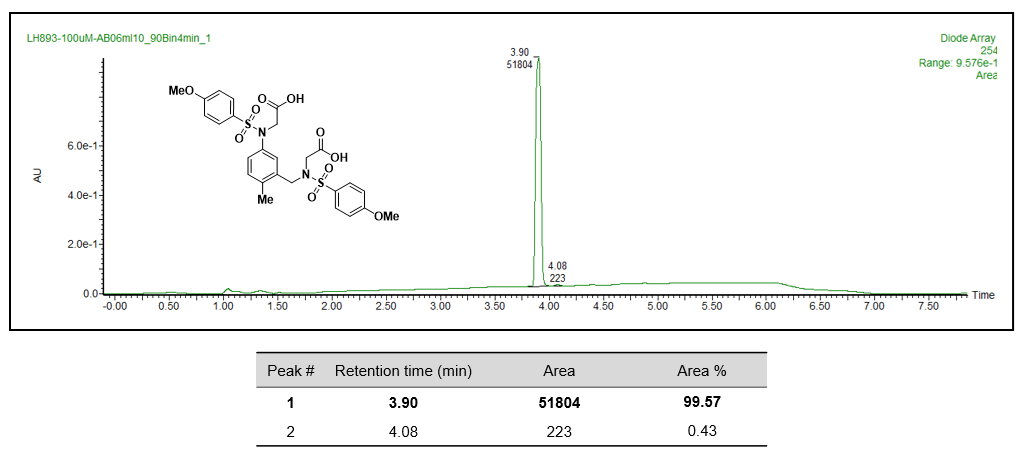
***

*UPLC analysis of compound* ***7e***

***
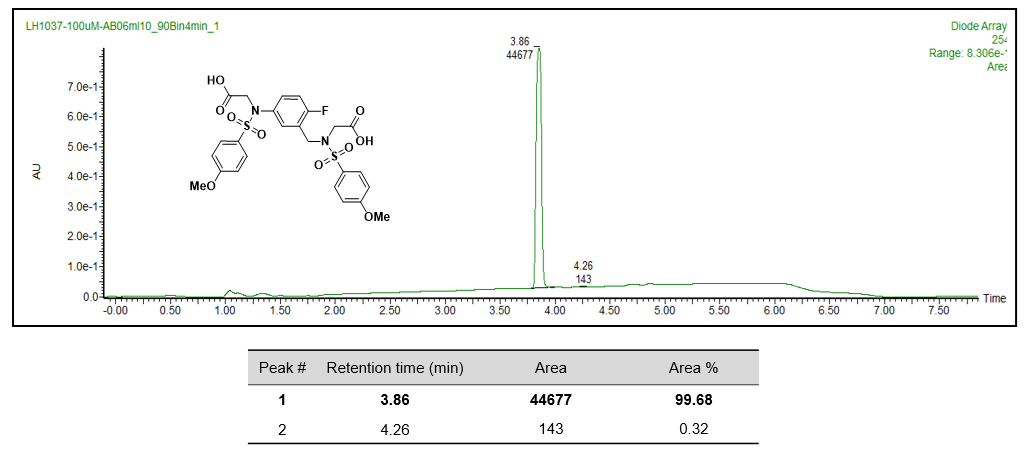
***

*UPLC analysis of compound* ***7f***

***
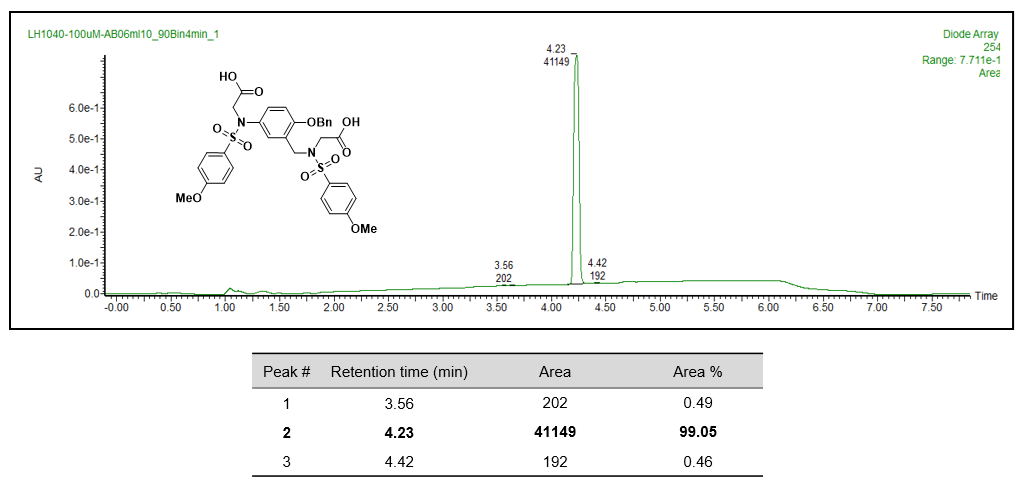
***

*UPLC analysis of compound* ***8***

***
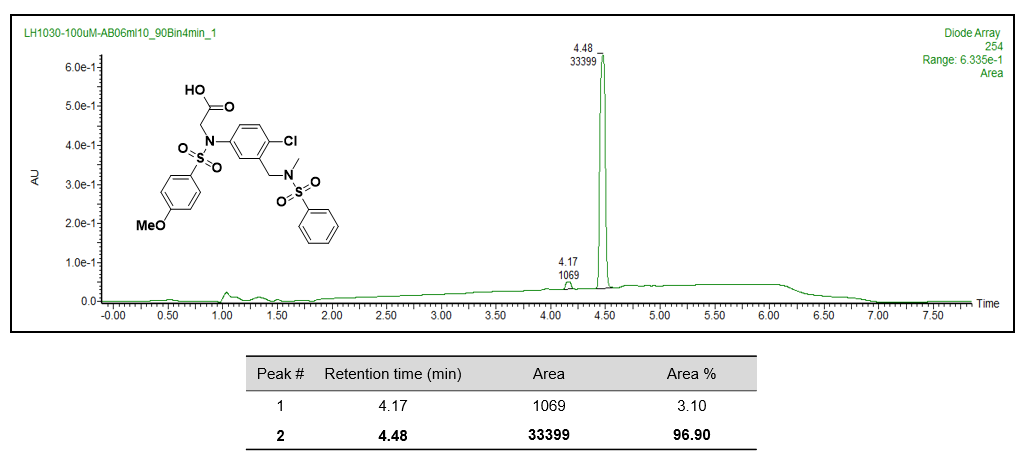
***

*UPLC analysis of compound* ***9***

***
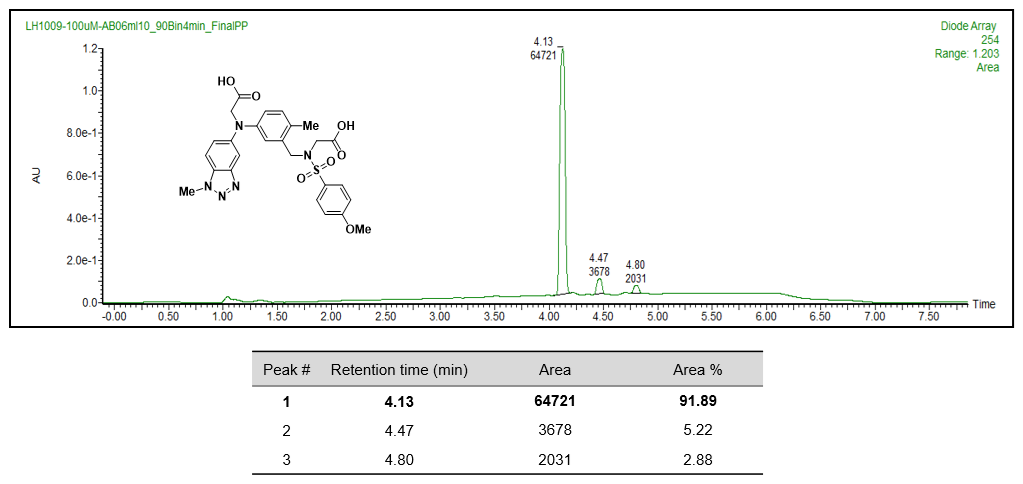
***

*UPLC analysis of compound* ***10***

***
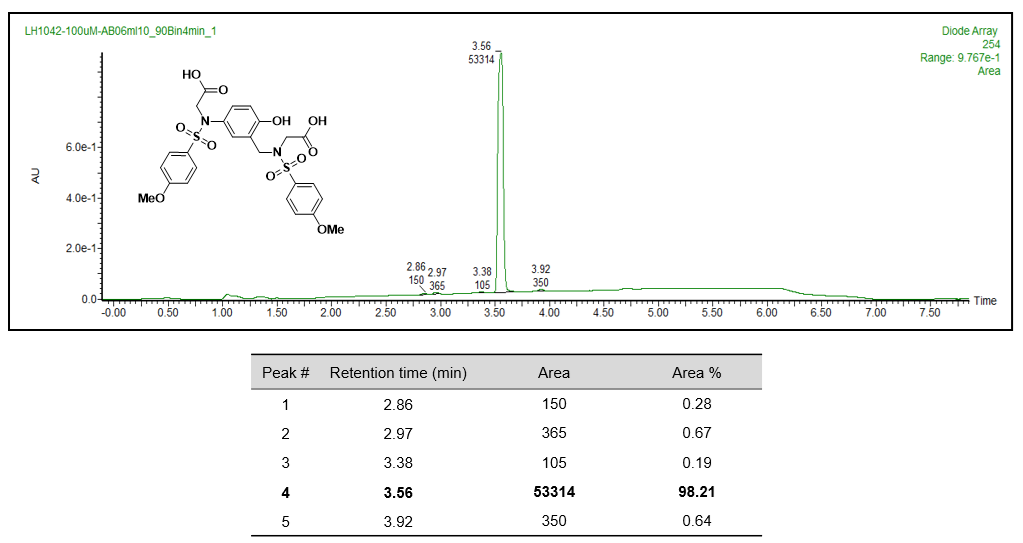
***

*UPLC analysis of compound* ***11a***

***
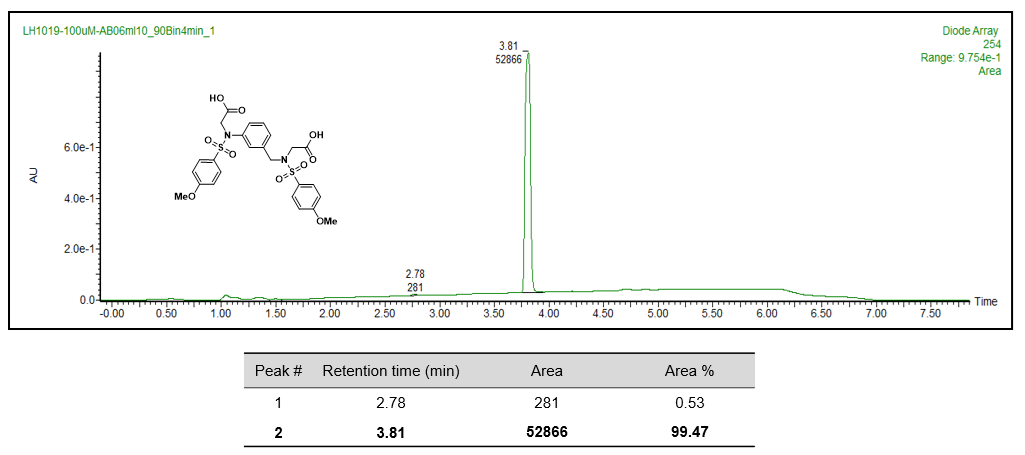
***

*UPLC analysis of compound* ***11b***

***
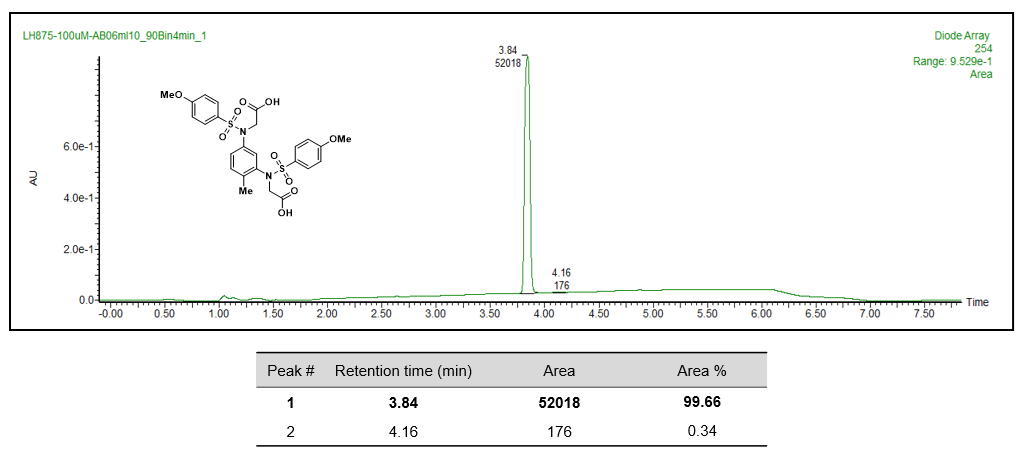
***

*UPLC analysis of compound* ***12a***

***
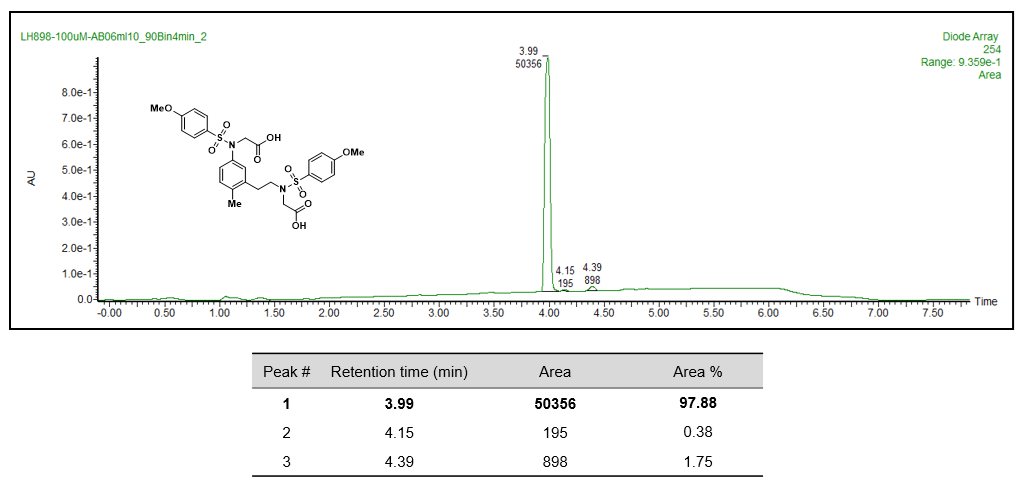
***

*UPLC analysis of compound* ***12b***

***
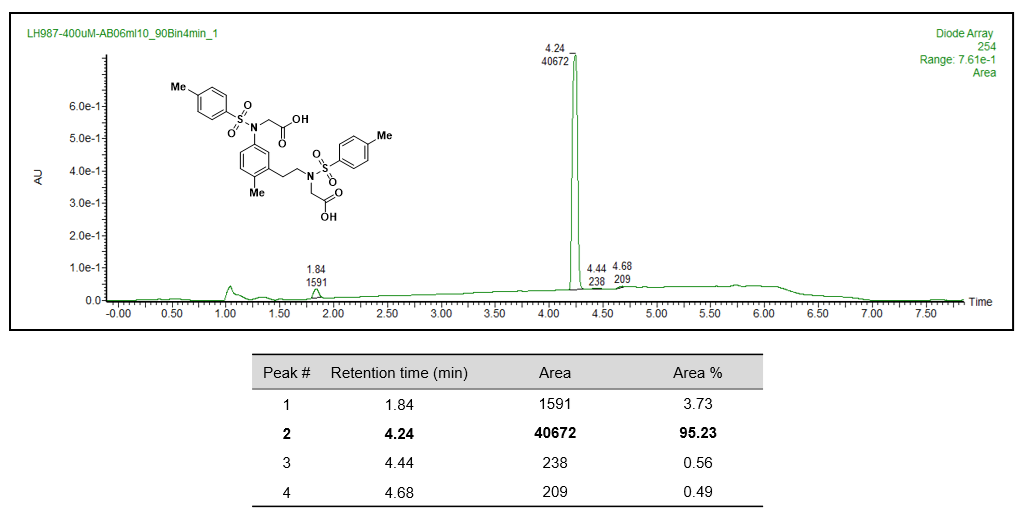
***

*UPLC analysis of compound* ***12c***

***
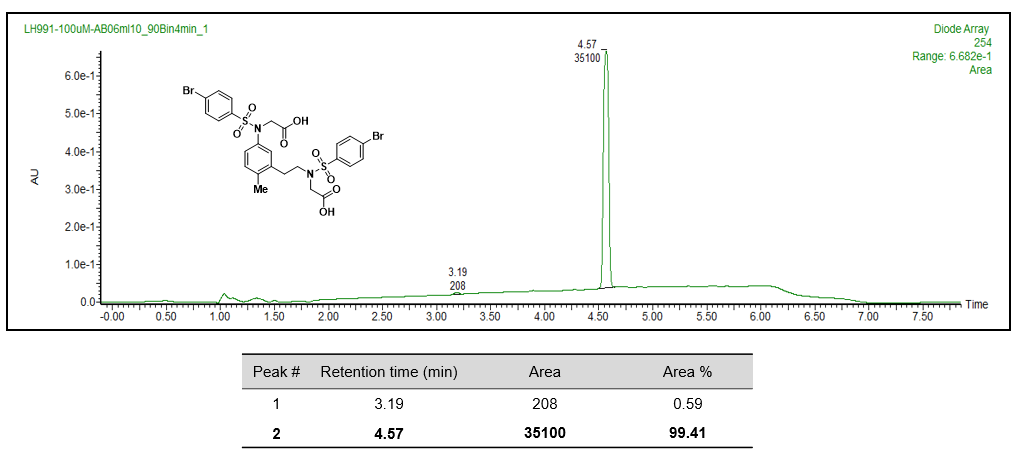
***

*UPLC analysis of compound* ***12d***

***
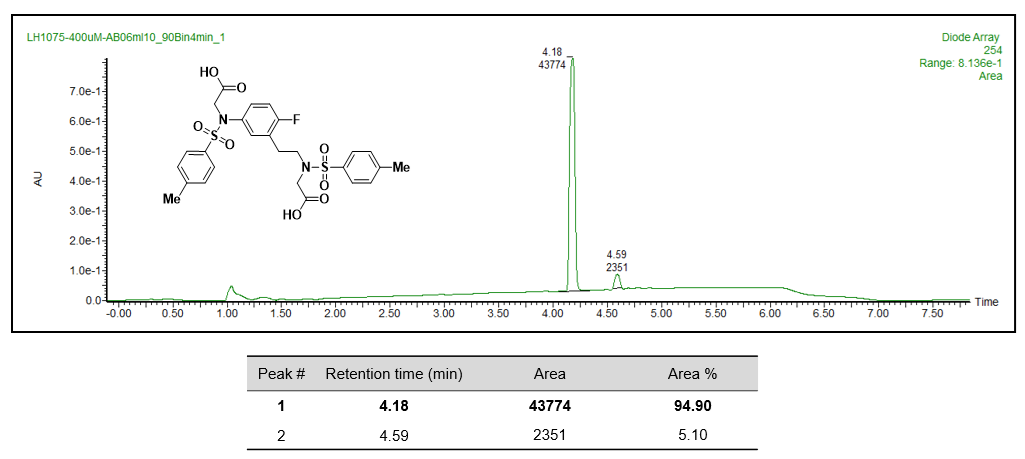
***

*UPLC analysis of compound* ***13a***

***
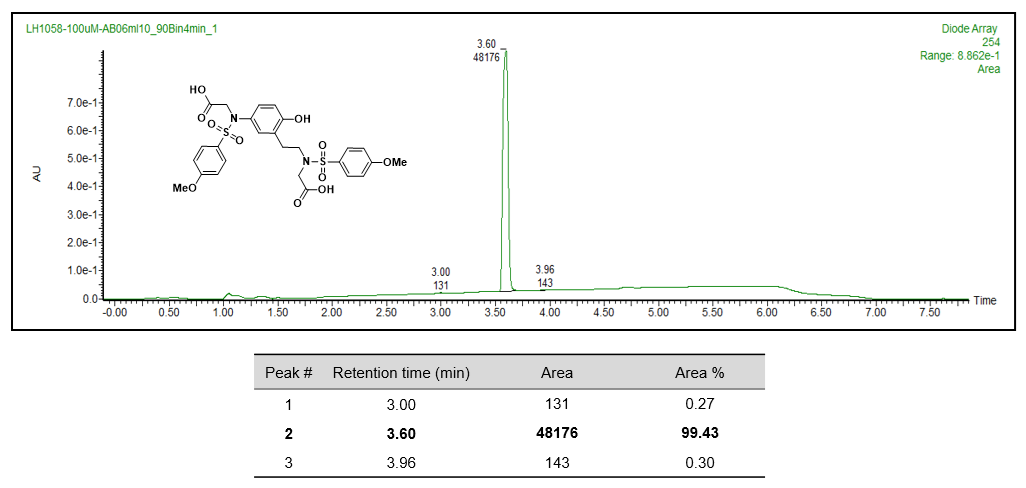
***

*UPLC analysis of compound* ***13b***

***
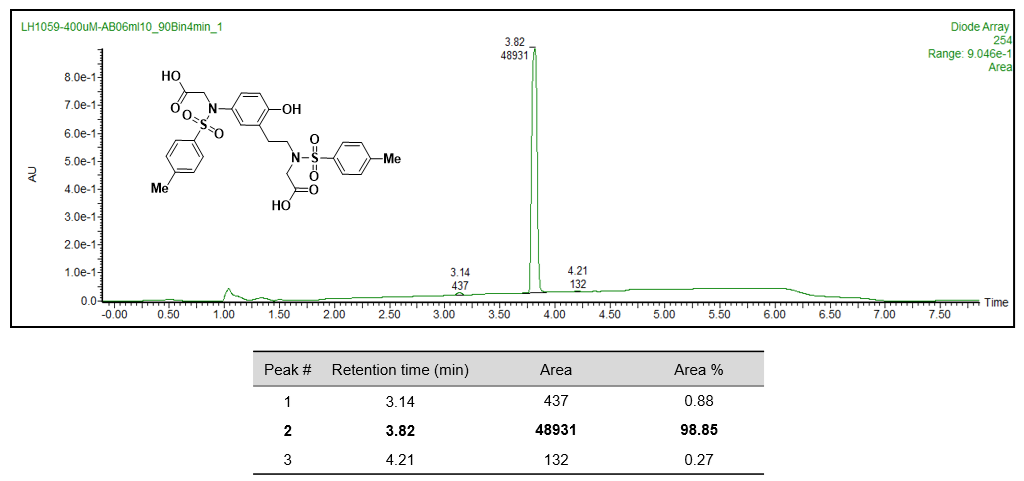
***

*UPLC analysis of compound* ***13c***

***
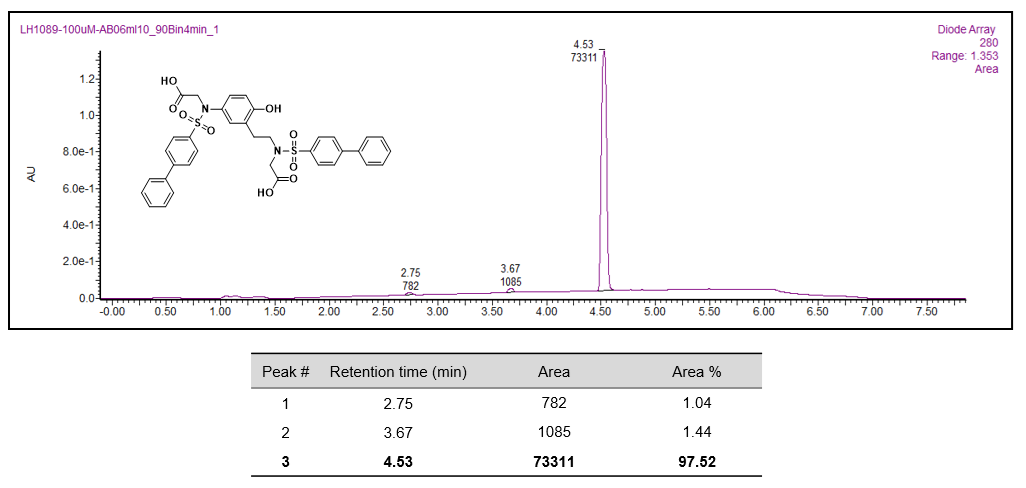
***

*UPLC analysis of compound* ***14***

***
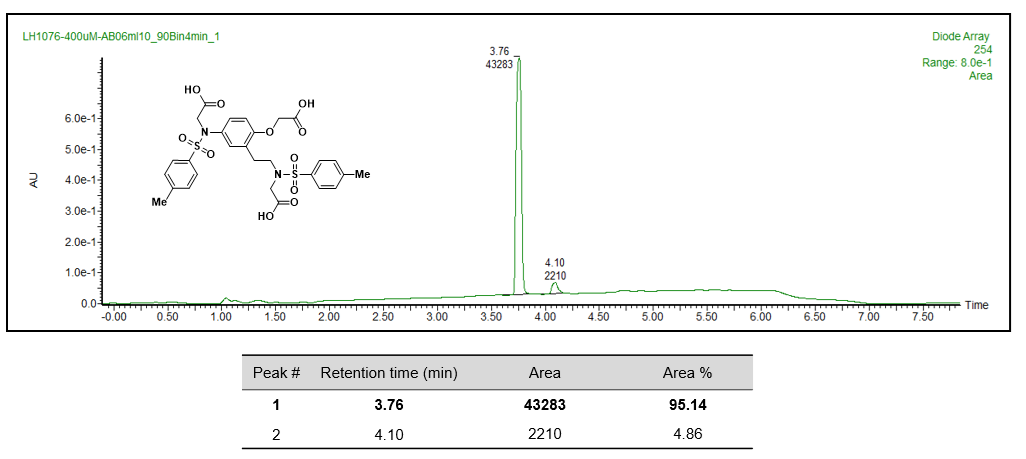
***

*UPLC analysis of compound* ***15a***

***
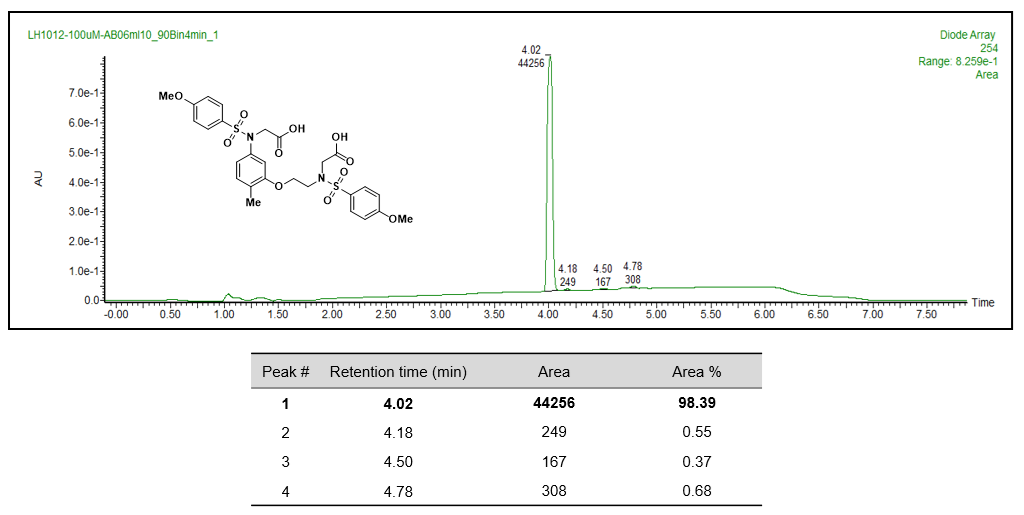
***

*UPLC analysis of compound* ***15b***

***
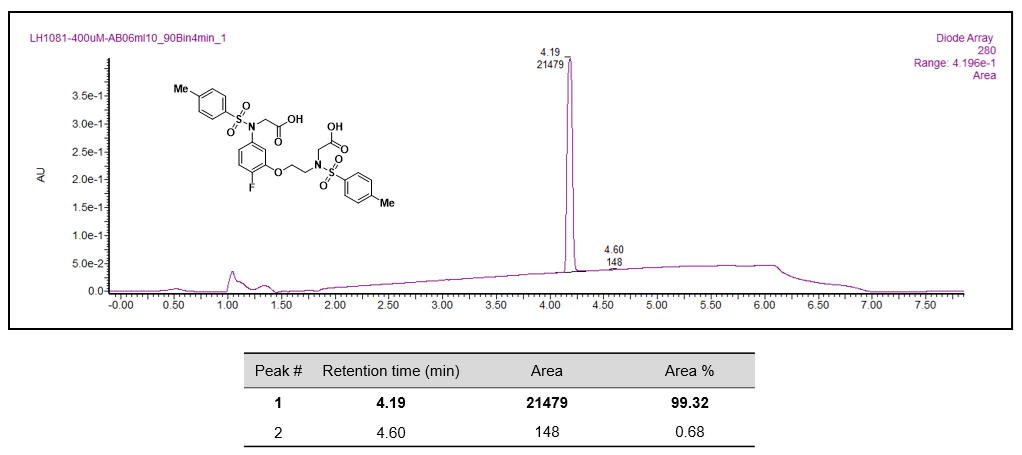
***

*UPLC analysis of compound* ***16***

***
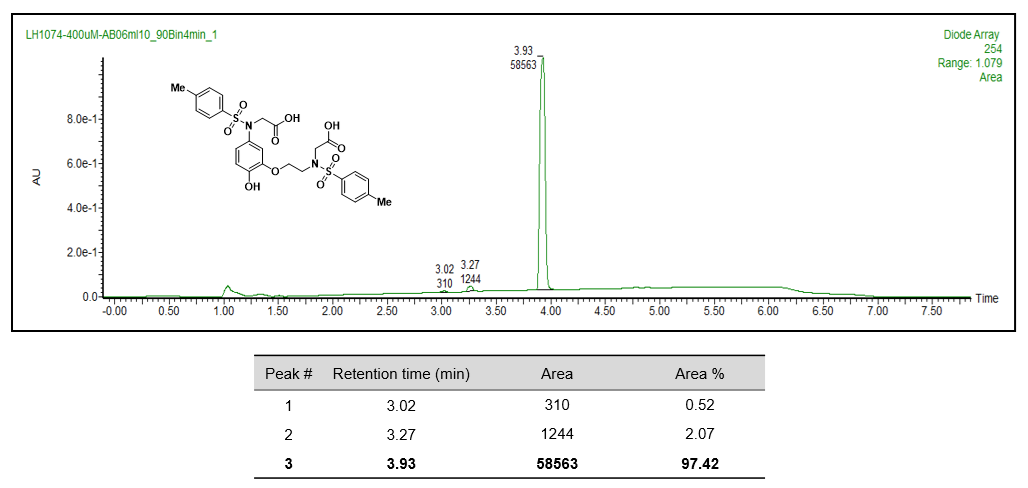
***

*UPLC analysis of compound* ***17***


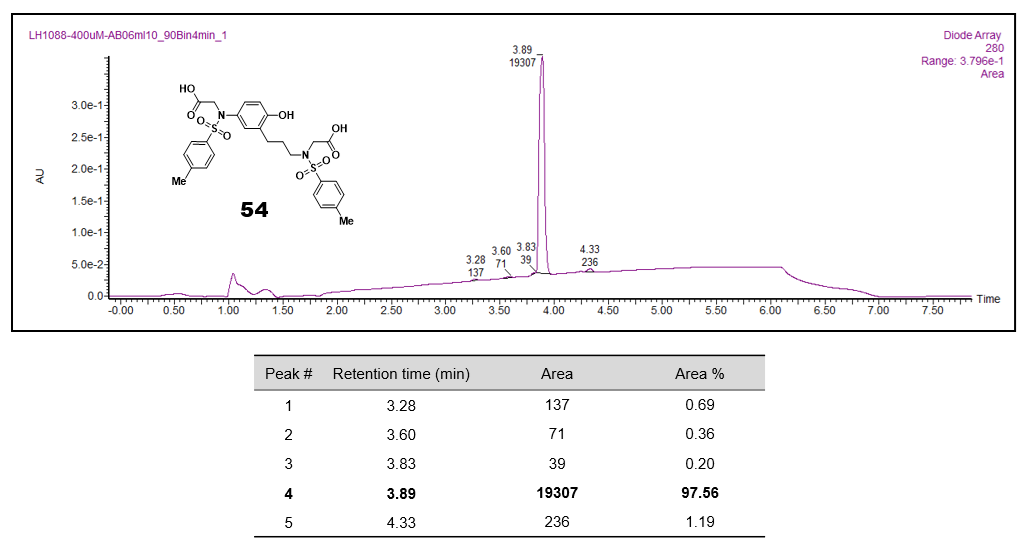

Supplement: Keap1Nrf2_MS3 SP_v9_122822025_anonymous.docx [file IENZ_A_2622777_SM4032.docx]
